# Zircon evidence for incorporation of terrigenous sediments into the magma source of continental basalts

**DOI:** 10.1038/s41598-017-18549-7

**Published:** 2018-01-09

**Authors:** Zheng Xu, Yong-Fei Zheng, Zi-Fu Zhao

**Affiliations:** CAS Key Laboratory of Crust-Mantle Materials and Environments, School of Earth and Space Sciences, University of Science and Technology of China, Hefei, 230026 China

## Abstract

Crustal components may be incorporated into continental basalts by either shallow contamination or deep mixing. While the former proceeds at crustal depths with common preservation of refractory minerals, the latter occurs at mantle depths with rare survival of relict minerals. Discrimination between the two mechanisms has great bearing to subcontinental mantle geochemistry. Here we report the occurrence of relict zircons in Cenozoic continental basalts from eastern China. A combined study of zircon U-Pb ages and geochemistry indicates that detrital zircons were carried by terrigenous sediments into a subcontinental subduction zone, where the zircon were transferred by fluids into the magma sources of continental basalts. The basalts were sampled from three petrotectonic units with distinct differences in their magmatic and metamorphic ages, making the crustal contamination discernible. The terrigenous sediments were carried by the subducting oceanic crust into the asthenospheric mantle, producing both soluble and insoluble materials at the slab-mantle interface. These materials were served as metasomatic agents to react with the overlying mantle wedge peridotite, generating a kind of ultramafic metasomatites that contain the relict zircons. Therefore, the occurrence of relict zircons in continental basalts indicates that this refractory mineral can survive extreme temperature-pressure conditions in the asthenospheric mantle.

## Introduction

Zircon is a refractory mineral in crustal rocks. Because of its chemically resistant property, zircon can survive from high-grade metamorphism and even partial melting^1,2^. The primary zircon of magmatic and peritectic origins is common not only in magmatic rocks of felsic composition but also in some mafic intrusives such as gabbro and pyroxenite. However, zircon rarely occurs in mafic volcanics such as basalts because the crystallization of zircon from silicate melts requires the oversaturation of both ZrO_2_ and SiO_2_^3,4^. This is not justified in basaltic melts that were derived from partial melting of the depleted MORB mantle, the ordinary asthenospheric mantle. Nevertheless, zircon megacrysts with U-Pb ages consistent with those of host rocks do occur in some alkali basalts. There are different interpretations for their origin, including xenocrysts from metasomatized lithospheric mantle^5^, and crystallized from the primitive magma of host basalts^6^, fractionated alkali basaltic magma^7,8^, or felsic melts derived from incipient melting of amphibole-rich mantle domains^9^. In addition, some basalts contain zircons with variably older U-Pb ages, which were interpreted as xenocrysts captured from metasomatic lithospheric mantle^10^ or felsic wallrocks^11^ during ascent of basaltic magmas.

On the other hand, old zircons in basalts can be produced through recycling of crustal components during the metasomatic formation of mantle sources at different depths^12,13^. Such basalts were derived from partial melting of ultramafic metasomatites in the ordinary asthenospheric mantle. This involves both chemical and physical transports of crustal materials from the subducting slab to the mantle, which is illustrated by the occurrence of relict zircons in Cenozoic continental basalts from east-central China. As such, survival of the detrital zircons becomes possible not only during partial melting of the subducting crustal rocks at the slab-mantle interface in the subcontinental subduction channel but also during partial melting of the metasomatic mantle domains above the subducted oceanic slab.

The target basalts were sampled from three petrotectonic units with significant differences in crustal composition, making the shallow contamination discernible. The three petrotectonic units are tectonically located in the southeastern edge of the North China Craton, the northeastern edge of the South China Block, and the Dabie-Sulu orogenic belt. The Dabie-Sulu orogenic belt was built by the Triassic subduction of the South China Block beneath the North China Craton^14^. Nevertheless, there is no considerable difference in basalt composition, exhibiting not only variable depletions in radiogenic Sr-Nd isotopes relative to bulk silicate Earth but also oceanic island basalts-like trace element distribution patterns on the primitive mantle-normalized spidergram^15–17^.

## Sample and Results

**Figure 1:**
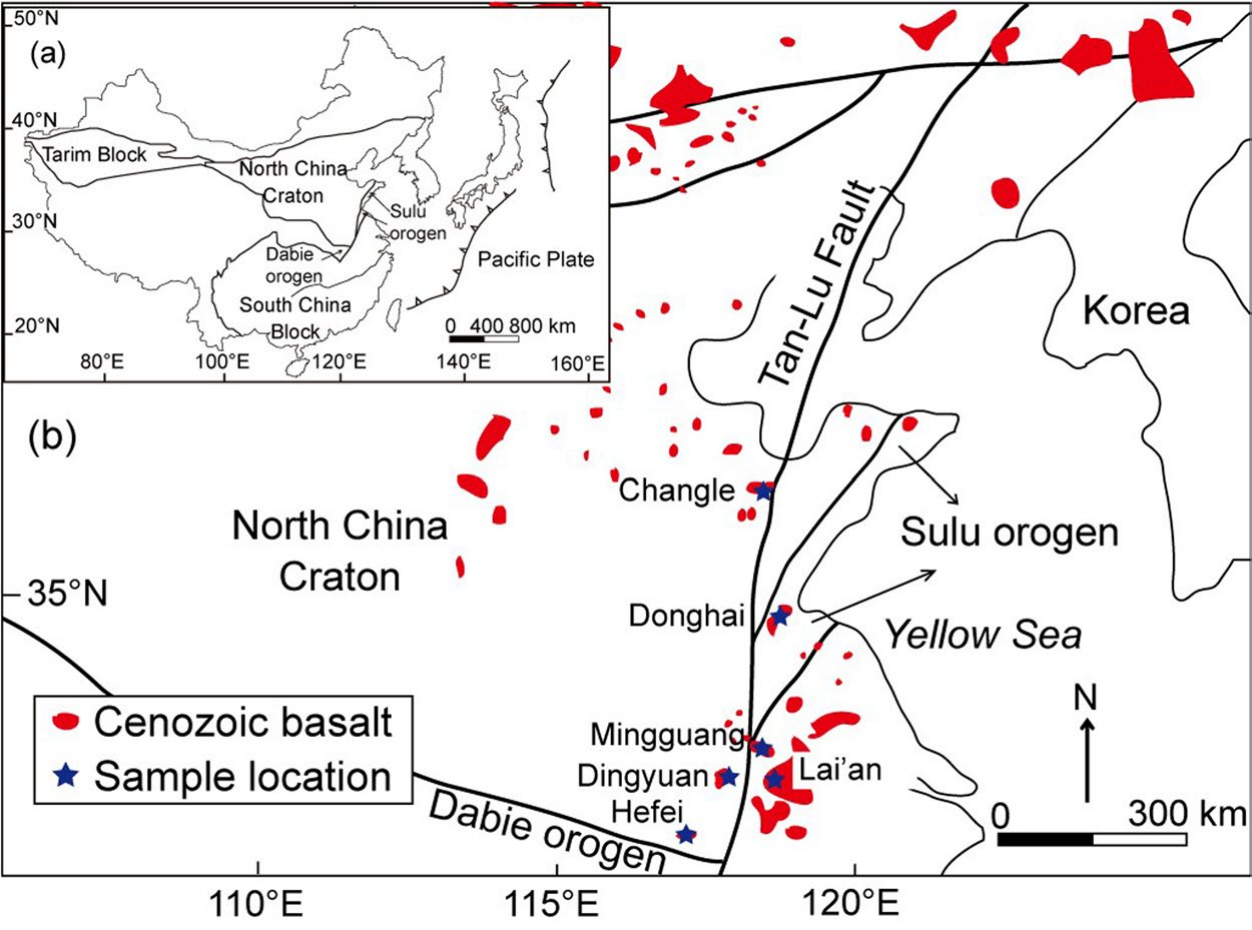
Sketch map of geology in east-central China showing distribution of Cenozoic continental basalts, and locations of the samples used in this study. This map was draw by Adobe Illustrator CC ver. 21.1.0 (https://www.adobe.com/products/illustrator.html). The base maps are drawn according to the map of the People’s Republic of China whereas the sample locations are based on the author’s collection.

Thirty basalt samples were selected for this study. They were collected from east-central China, close to the Tan-Lu Fault (Fig. 1). Geographically, they are located at Donghai in northern Jiangsu (Subei), at Dingyuan, Mingguang and Lai’an in eastern Anhui (Wandong), at Hefei in central Anhui, and at Changle in western Shandong (Luxi). Although these localities are geographically close to the Dabie-Sulu orogenic belt that is characterized by the occurrence of Triassic ultrahigh-pressure (UHP) metamorphic rocks, only the basalts at Donghai are tectonically located inside the Sulu orogen. The other localities of basalts fall in petrotectonic units outside the Dabie-Sulu orogenic belt. The basalts at Changle, Hefei and Dingyuan basalts are located in the southeastern edge of the North China Craton, northwest to the Dabie-Sulu orogenic belt; those at Mingguang and Lai’an are located in the northeastern edge of the South China Block, south to the Dabie-Sulu orogenic belt^18^.

All these basalts are generally porphyritic in texture. Phenocryst are olivine ± clinopyroxene ± plagioclase, matrix contains all these three minerals in fine grains. Olivine phenocrysts are subhedral to anhedral and usually show fractures. Plagioclase exhibits as tabular with small olivine and pyroxene grains filling between them. They usually have polysynthetic twinning. Clinopyroxene phenocrysts are subhedral. Compositional zoning is present in some olivine and clinopyroxene phenocrysts. Zircon is rare in the basalts and hardly observed in thin sections under a microscope. Nevertheless, mineral separation yields considerable amounts of zircon grains for U-Pb dating and geochemical analyses. The all basalt samples from the Sulu orogen and part of samples from the North China Craton and the South China Block show slight low-temperature alteration after eruption, resulting in transformation of some olivines to iddingsites. Petrography and geochemistry of the target basalts have been investigated in detail^15–17^.

**Figure 2:**
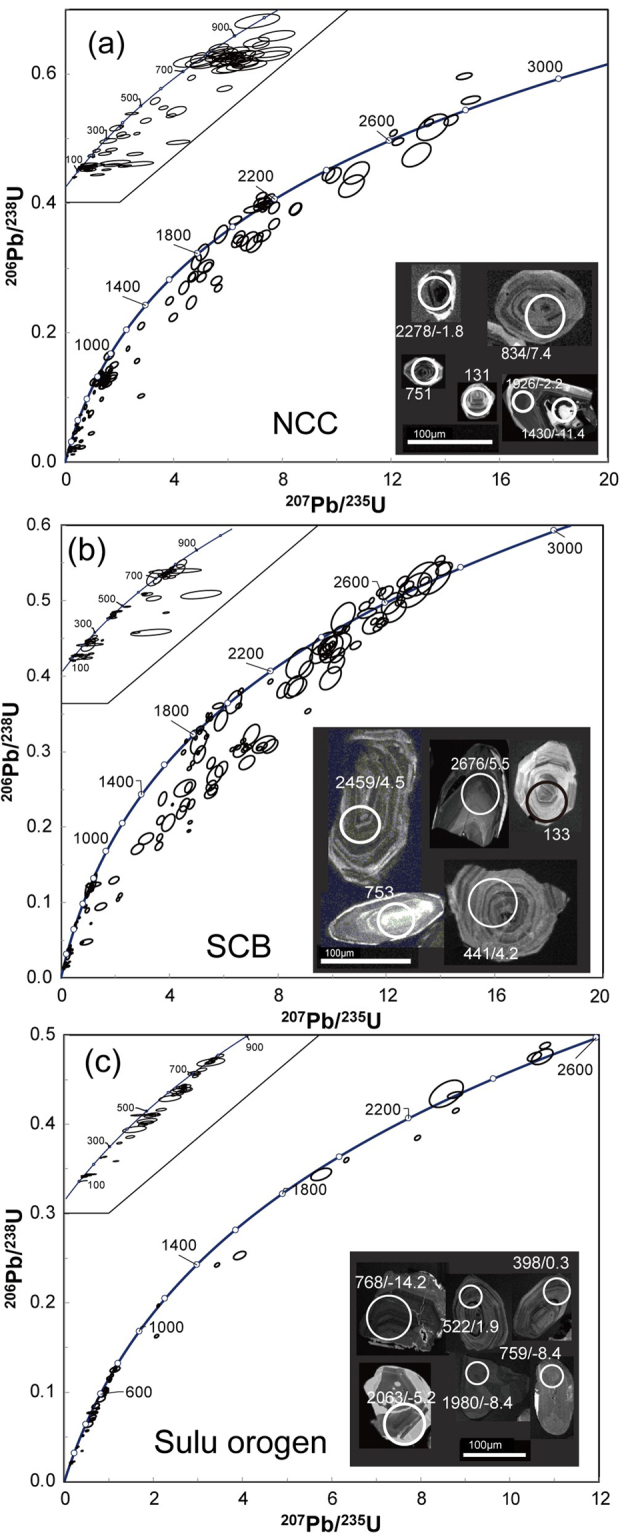
U-Pb ages and selected CL images for zircons from zircons from Cenozoic continental basalts in east-central China. Ages in concordia diagrams are in Ma. Data for each spot in CL images are in the form of U-Pb age (Ma)/ε_Hf_(t). Samples locality: (**a**) the North China Craton (NCC), (**b**) the South China Block (SCB), (**c**) the Sulu orogen.

Most zircon grains from the basalts are subhedral or rounded, without euhedral ones. The internal structural of these zircons are complicated in the CL images (Fig. 2). Many zircons show clear oscillatory or sector zoning, and many zircons exhibit weekly, planar or no zoning. Many zircons show core-(mantle)-rim structures, and most of them exhibit the relict core of oscillatory or sector zoning with the overgrown rims of weekly, planar or no zoning. However, some zircons show the relict cores of weekly, planar or no zoning with overgrown rims of oscillatory or sector zoning.

**Figure 3:**
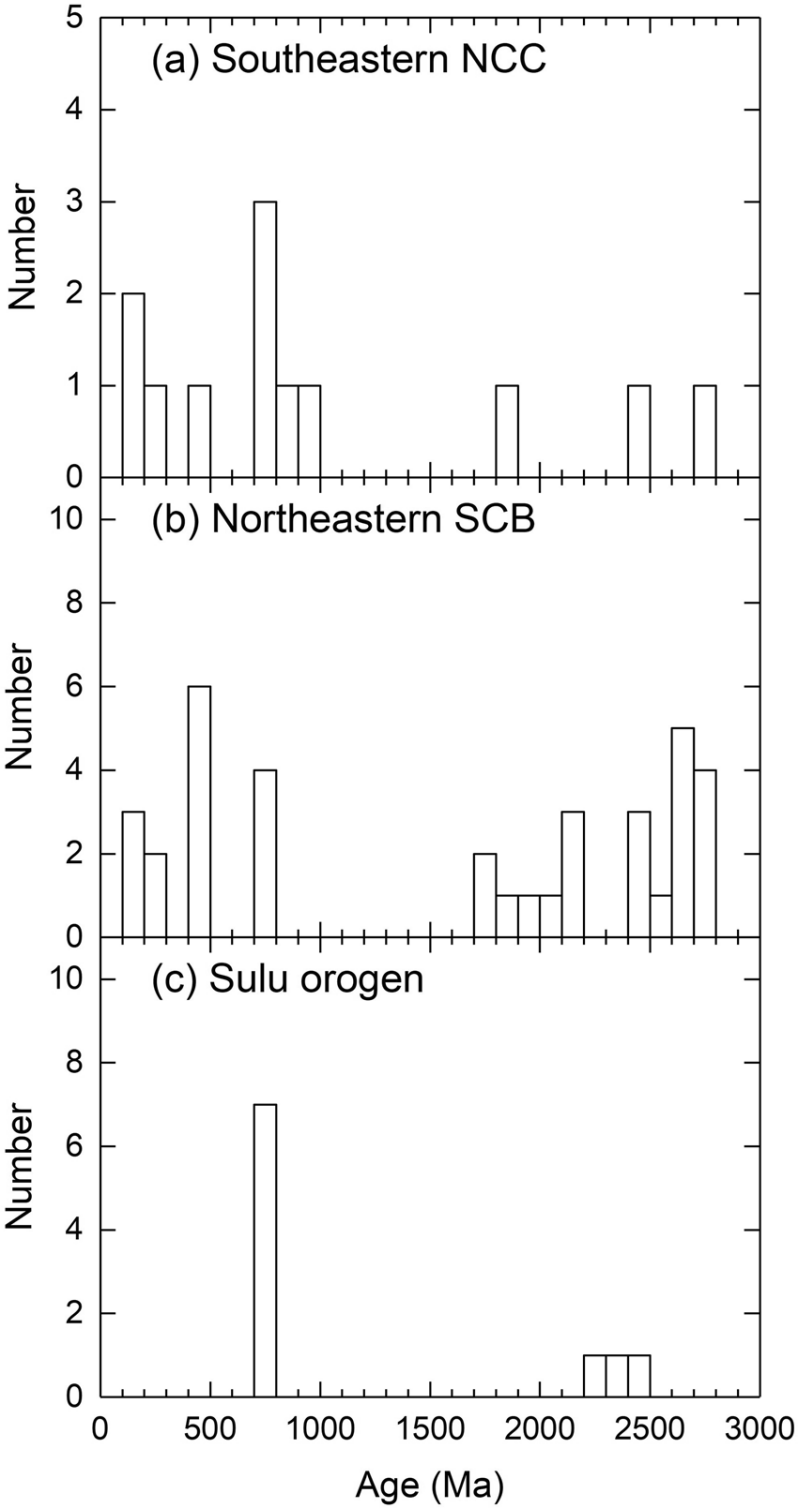
Histograms of concordant U-Pb ages for zircons from Cenozoic continental basalts in east-central China. Samples locality: (**a**) the North China Craton (NCC), (**b**) the South China Block (SCB), (**c**) the Sulu orogen. Data are listed in Tables S1 and S2 in Supplementary data.

**Figure 4:**
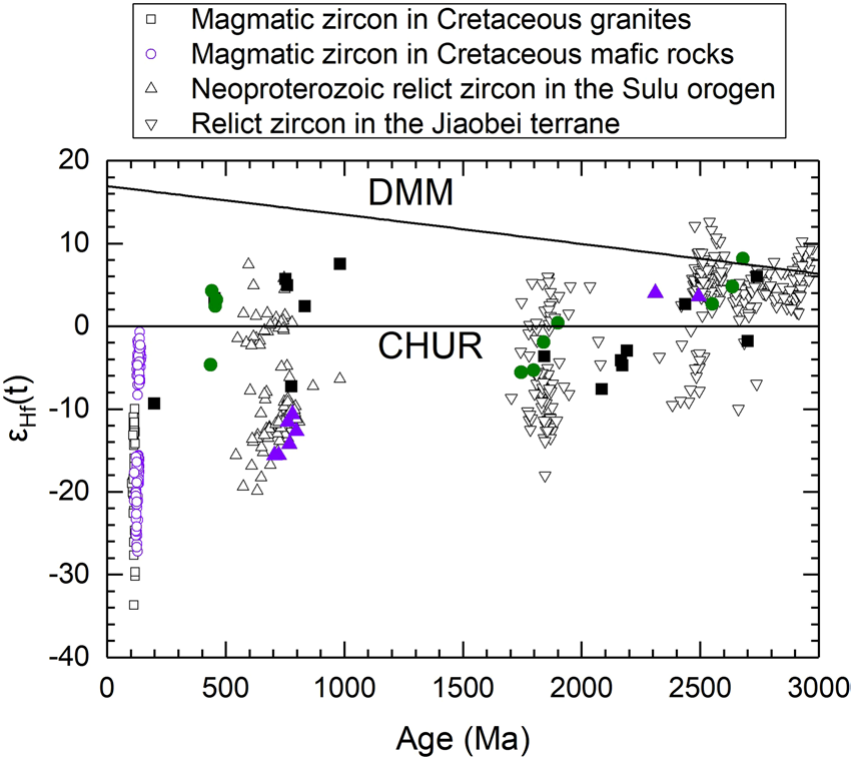
ε_Hf_(t) vs. U-Pb ages diagram for zircons with concordant U-Pb ages from the Cenozoic basalts, the Jiaobei terrane, the Sulu orogen and the Cretaceous igneous rocks. Filled square, circle and triangle symbols denote the zircons in basalts from the North China Craton, the South China Block and the Sulu orogen, respectively. Data source: the Jiaobei terrane^38–44^; the Sulu orogen^44–48,50,66,67^; the Cretaceous granites^63,64,68^; the Cretaceous mafic rocks^54,64^.

**Figure 5:**
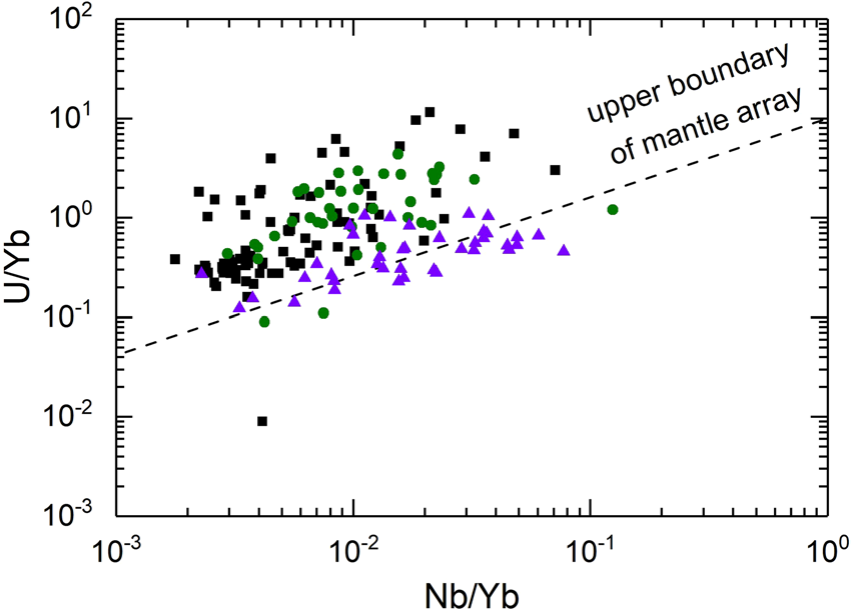
The plot of U/Yb vs. Nb/Yb ratios for zircons from Cenozoic continental basalts in east-central China. Data are listed in Table S4. Square, circle and triangle symbols denote the zircons in basalts from North China Craton, the South China Block and the Sulu orogen, respectively.

Although the host basalts are located in three petrotectonic settings, zircons from each tectonic setting do not show considerable differences in zircon U-Pb ages, trace elements and Lu-Hf isotopes (Table 1). Zircons exhibit a large range in apparent U-Pb ages from 101 to 3015 Ma (Fig. 1, and Tables S1 and S2 in Supplementary Information), with two prominent peaks around 800 and 2500 Ma. Although only a few zircons have concordant U-Pb ages of 106 to 2786 Ma, they are mainly clustered in the middle Neoproterozoic, Triassic and Early Cretaceous, respectively (Fig. 3). The all zircons show a large variation in Th/U ratios from 0.01 to 4.74. Zircons from the basalts show ε_Hf_(t) values of −18.9 to 9.9 (Table S3 in Supplementary Information). Zircon Hf model ages are 883 to 3280 Ma. Zircons with concordant U-Pb ages show variable ε_Hf_(t) values from −18.9 to 9.4 (Fig. 4), and their Hf model ages range from 883 to 3280 Ma. Zircons with U-Pb ages younger and older than 1000 Ma show similar ε_Hf_(t) values, with a positive correlation between ε_Hf_(t) values and U-Pb ages. On the other hand, zircons with U-Pb ages younger than 1000 Ma show a large variation in ^176^Lu/^177^Hf ratios relative to those of zircons older than 1000 Ma (0.000301 to 0.004743 vs. 0.000060 to 0.003006). Moreover, Th/U ratios are correlated neither with ^176^Lu/^177^Hf nor with ^176^Hf/^177^Hf ratios. Zircons exhibit variable HREE enrichments with highly variable (Yb/La)_n_ ratios from 1.6 to 19750 and (Lu/Gd)_n_ ratios from 2.4 to 107 and highly variable Eu anomalies with Eu/Eu* ratios from 0.01 to 0.64 and Ce anomalies with Ce/Ce* ratios from 0.29 to 83.3 (Table S4 in Supplementary Information). They show a roughly positive correlation between U/Yb and Nb/Yb ratios (Fig. 5).

**Table 1:** Summary of zircon U–Pb ages and geochemistry for Cenozoic continental basalts from eastern China.

Geochemical variable	NCC	SCB	Sulu
Th (ppm)	11–8966	0.4–4908	16–1741
U (ppm)	24–8942	27–3555	23–3315
Th/U	0.03–3.42	0.01–4.74	0.04–2.57
(Yb/La)_n_	1.6–7551	4.3–10122	3.7–19750
(Lu/Gd)_n_	2.4–107	6.1–46.2	3.0–41.9
Apparent U-Pb age (Ma)	101–3015	106–2841	119–2563
Concordant U-Pb age (Ma)	122–2786	106–2740	478–2493
ε_Hf_(t)	−15.9–9.4	−18.6–9.9	−18.9–8.4
T_DM_ (Ma)	932–3061	883–3280	1007–3010

Abbreviations: NCC, the North China Craton; SCB, the South China Block; Sulu, the Sulu orogen.

## Discussion

It is known that all these continental basalts from east-central China have Cenozoic eruption ages of 6 to 38 Ma^15–17^. In contrast, the zircons from the basalts show highly variable U-Pb ages from 101 to 2865 Ma (Tables S1 and S2). This age difference indicates that these zircons did not crystallize from basaltic magmas. Furthermore, the majority of basalt zircons plot above the upper boundary of the mantle array in the U/Yb vs. Nb/Yb diagram (Fig. 4), inconsistent with their direct growth from mantle-derived magmas^19^. Instead, they would originate either from the continental crust through shallow contamination during magma emplacement or from the mantle sources that were generated by deep metasomatism before basaltic magmatism. As such, they are either xenocrysts from the continental crust or inherited from crustal components in the magma sources of continental basalts.

The concordant zircon U-Pb ages for the Cenozoic continental basalts are mainly clustered in the middle Neoproterozoic, Triassic and Early Cretaceous ages (Fig. 2), respectively, in general agreement with those for magmatic and metamorphic zircons from the Dabie-Sulu orogenic belt^2,20,21^. In particular, the middle Neoproterozoic U-Pb ages of 700 to 800 Ma are prominent. Such an age range is characteristic in the South China Block (including the Dabie-Sulu orogenic belt in its northern margin) but absent in the North China Craton^22^. This precludes the possibility that these zircons would be of xenocrystic origin through the crustal contamination during the ascent of basaltic magmas through the continental crust of the North China Craton. Furthermore, the Triassic U-Pb ages of 200 to 245 Ma are consistent with those for zircons from UHP metamorphic rocks in the Dabie-Sulu orogenic belt but absent in crustal rocks from either the North China Craton or the South China Block outside the Dabie-Sulu orogenic belt^2,20,21^. Therefore, the old zircons in the target basalts are not the xenocrysts from the local continental crust.

It is known that zircon only occurs as a tiny accessory mineral in very small amounts in crustal rocks; even silica-saturated granitic rocks contain very little zircons. If the crustal contamination could be responsible for the origin of old zircons in basalts, there would be concurrent changes in the whole-rock major and trace element compositions of the resulted basalts. In this regard, the effect of crustal contamination on zircon addition to continental basalts can be tested by using a simple calculation to see whether there is a problem in mass balance. This is primarily gauged with the amount of crustal rocks in basaltic magmas in view of melt-mobile incompatible elements relative to the amount of xenocrystic zircons in erupted basalts. Supposing that 1 gram zircon is captured by an ascending basaltic magma, several hundreds to thousands grams of the crustal rocks are necessarily incorporated into the basaltic magma at the same time. Consequently, the finally erupted basalt must show a considerable increase in typical crustal components such as SiO_2_ and LILE (e.g., Rb, K and Pb), which are melt-mobile during assimilation. However, these features are absent in our basalts, arguing against the effect of crustal contamination^15–17^.

An alternative interpretation for the origin of old zircons in the Cenozoic continental basalts is the source inheritance from the metasomatic agents of terrigenous origin. This mechanism has been used to explain the occurrence of old zircons in metasomatic pyroxenites veins within peridotite xenoliths carried by continental basalts in North China^12^ and in volcanic arc rocks above the Caribbean subduction zone in Cuba^13^. If these zircons are inherited from crustal components, they must survive during metamorphic dehydration and partial melting at UHP conditions^23,24^. The survival of detrital zircons during subduction zone metamorphism is also indicated by the occurrence of inherited zircons with Precambrian relict cores and Triassic metamorphic rims in UHP metamorphic rocks from the Dabie-Sulu orogenic belt in east-central China^25^, where the crustal rocks were subducted to subarc depths for the Triassic UHP metamorphism^2,20,21^.

Furthermore, detrital zircons may survive during partial melting at ultrahigh-temperature (UHT) conditions of 900 to 1100 °C^26–28^. Despite small amounts, relict magmatic zircons do not show significant disturbance of not only U-Pb and Lu-Hf isotope systems but also trace element composition in UHT metamorphic rocks from the Bohemian Massif in central Europe^28^ and the Napier Complex in Antarctica^26,27^. In fact, a subset of detrital zircons were identified in the enriched MORB from the Macquarie Island ophiolite^29^. In addition, old zircons have been recovered from mafic igneous rocks from Indian mid-ocean ridges^30,31^. These observations indicate that zircons do have survived at temperatures for the generation of enriched MORB, which are derived from decompressional melting of the asthenospheric mantle at temperatures of 1300–1400 °C. In this case, the old zircons may be inherited from the mantle sources of their host rocks rather than captured from an unknown continent crust fragment.

It is known from the study of zircons in metamorphic veins inside UHP metamorphic rocks that tinny zircon grains can be physically transported from subducting crustal rocks by metamorphic fluids^32,33^. Both newly grown and relict zircon domains occur in orogenic peridotites from the Dabie-Sulu orogenic belt^23,34–37^. Such observations indicate that both the chemical transport of dissolved Zr and the physical transport of crustal zircon by metasomatic agents (metamorphic fluids, anatectic melts, or both) have taken place at the slab-mantle interface in subcontinental subduction channels^14^. During partial melting of the metasomatic mantle domains, these tinny zircon grains may not be completely dissolved into basaltic melts, and some of them may survive and be physically transported by the basaltic melts to the surface.

The occurrence of relict zircons in the Cenozoic continental basalts indicates that subduction zone fluids would have served as metasomatic agents to transport the detrital zircons from the orogenic crust via the mantle wedge to the basaltic melts. This involves two-stage processes in mass transfer at the slab-mantle interface in the subcontinental subduction channel. The first stage is analogue to the occurrence of Precambrian zircons in metasomatic pyroxenite veins inside peridotite xenoliths entrained by Cenozoic continental basalts^12^. The second stage is analogue to the occurrence of inherited zircons in intra-arc mafic igneous rocks above the Caribbean subduction zone^13^. In order to document this possibility, we have made a detailed discussion in Supplementary Information on the occurrence of magmatic zircon in mafic igneous rocks and the survival of detrital zircons at deep subduction zones. The results indicate that the old zircons were capable of survival at the lithosphere-asthenosphere boundary. As such, they were inherited from their mantle sources that contain the tinny zircon grains of crustal origin from the recycling of subducted crustal rocks in the subcontinental subduction channel.

It is known that zircon U-Pb ages for high-grade gneisses from the Jiaobei terrane in the southeastern margin of the North China Craton exhibit three episodes of magmatic events around 1.8 Ga, 2.5 Ga and 2.7 Ga^38–44^. This is consistent with the concordant U-Pb age peaks at 2.5 Ga and 2.7 Ga for Paleoproterozoic to Archean zircons from the Cenozoic continental basalts (Tables S1 and S2). Furthermore, they show ^176^Lu/^177^Hf and ^176^Hf/^177^Hf ratios of 0.000002 to 0.003050 and 0.280405 to 0.282943, respectively, which are similar to those of grains or relic cores for zircon with U-Pb ages older than 1700 Ma in the basalts (Table S4). Thus, the Jiaobei terrane could serve as a source of the oldest detrital zircons. Moreover, the old magmatic zircons with U-Pb ages around 2.5 and 2.7 Ga from the basalts have positive ε_Hf_(t) values (Fig. 4 and Table S4), which are consistent with positive ε_Hf_(t) values for zircons from the Jiaobei terrane^44^. Thus the ancient crustal rocks from the Jiaobei terrane could be the source of the Paleoproterozoic to Archean zircons in the Cenozoic continental basalts. In other words, the Paleoproterozoic to Archean zircons in the basalts would be sourced from weathering of the continental crust in the Jiaobei terrane.

The Neoproterozoic relict zircons show concordant U-Pb ages of 705 to 983 Ma (Tables S1 and S2). These ages cover the U-Pb age range of 740 to 800 Ma for UHP metaigneous protoliths in the Sulu orogen^2,22,45–49^. These relict zircons show Th/U ratios of 0.15 to 1.56, which resemble Th/U ratios of 0.19 to 4.49 for the relict zircon cores of Neoproterozoic U-Pb ages in the UHP metaigneous rocks from the Sulu orogen. Furthermore, the ^176^Hf/^177^Hf ratios of Neoproterozoic relict zircons in the Cenozoic continental basalts are similar to those of most relict cores in the UHP metaigneous rocks^45–48,50,51^, giving similar ε_Hf_(t) values (Fig. 4). In this regard, the Neoproterozoic relict zircons in the basalts were possibly originated from the UHP metamorphic rocks in the Sulu orogen.

On the other hand, some of the studied Cenozoic continental basalts are spatially distributed along the southeastern edge of the North China Craton. It is likely that the subducting Paleotethyan oceanic slab could have influenced the overlying cratonic mantle wedge. In this case, zircons could come from the terrigenous sediments that were weathered from the continental crust in the northeastern margin of the South China Block and they would be carried by the subducting Paleotethyan oceanic crust into the mantle sources of Cenozoic continental basalts. As a consequence, the Neoproterozoic magmatic zircons in the Cenozoic basalts could be inherited from the seafloor sediment overlying the subducting Paleotethyan oceanic crust. However, the mantle source of Early Cretaceous mafic igneous rocks in the southeastern margin of the North China Craton has been documented as the metasomatic product through reaction of the ancient cratonic mantle with felsic melts derived from partial melting of the subducting continental crust of the South China Block^52–55^. Because the South China Block was subducted subsequent to the subduction of the Paleotethyan oceanic crust in the Late Paleozoic, the Early Cretaceous mafic igneous rocks should contain the geochemical signature from the interaction between the cratonic mantle and the subducting Paleotethyan oceanic crust. However, no such a signature has been found so far in these rocks. In this regard, the influence of the subducted Paleotethyan oceanic crust on the mantle sources of Cenozoic continental basalts is insignificant. Therefore, the Neoproterozoic relict zircons in the Cenozoic basalts would be not originated from the seafloor sediment overlying the subducting Paleotethyan oceanic crust in east-central China.

The metamorphic zircons of Triassic U-Pb ages are common in crustal rocks from the Dabie-Sulu orogenic belt^2,45–51,56^. They are the product of metamorphic growth during the subduction of the South China Block beneath the North China Craton. These zircons exhibit similar ^176^Hf/^177^Hf ratios to the Triassic zircons in the Cenozoic continental basalts. The majority of these metamorphic zircons show very low Th/U ratios of <0.1^2,^ ^45–51^, consistent with the low Th/U ratios for the majority of Triassic zircons in the basalts (Tables S1 and S2). Therefore, the UHP metamorphic rocks from the Sulu orogen were the source of Triassic zircons in the basalts.

A few zircons from the Cenozoic continental basalts show concordant U-Pb ages of 101 to 131 Ma (Tables S1 and S2). Such young ages are common in postcollisional magmatic rocks from the Sulu orogen^52,53,57–65^. Zircons in Mesozoic gabbros with U-Pb ages around 130 Ma always show Th/U ratios of >1.0, with Th and U contents mainly between 50 to 300 ppm^52,53,57,62,64^. These are consistent with those for zircons with U-Pb ages around 130 Ma in the Cenozoic continental basalts, which exhibit Th/U ratios generally higher than 1.0 with Th and U contents mainly between 100 to 300 ppm (Tables S1 and S2). In this regard, it is possible that the Early Cretaceous magmatic rocks in the Sulu orogen would have provided the detrital zircons to the mantle source of Cenozoic basalts.

**Figure 6:**
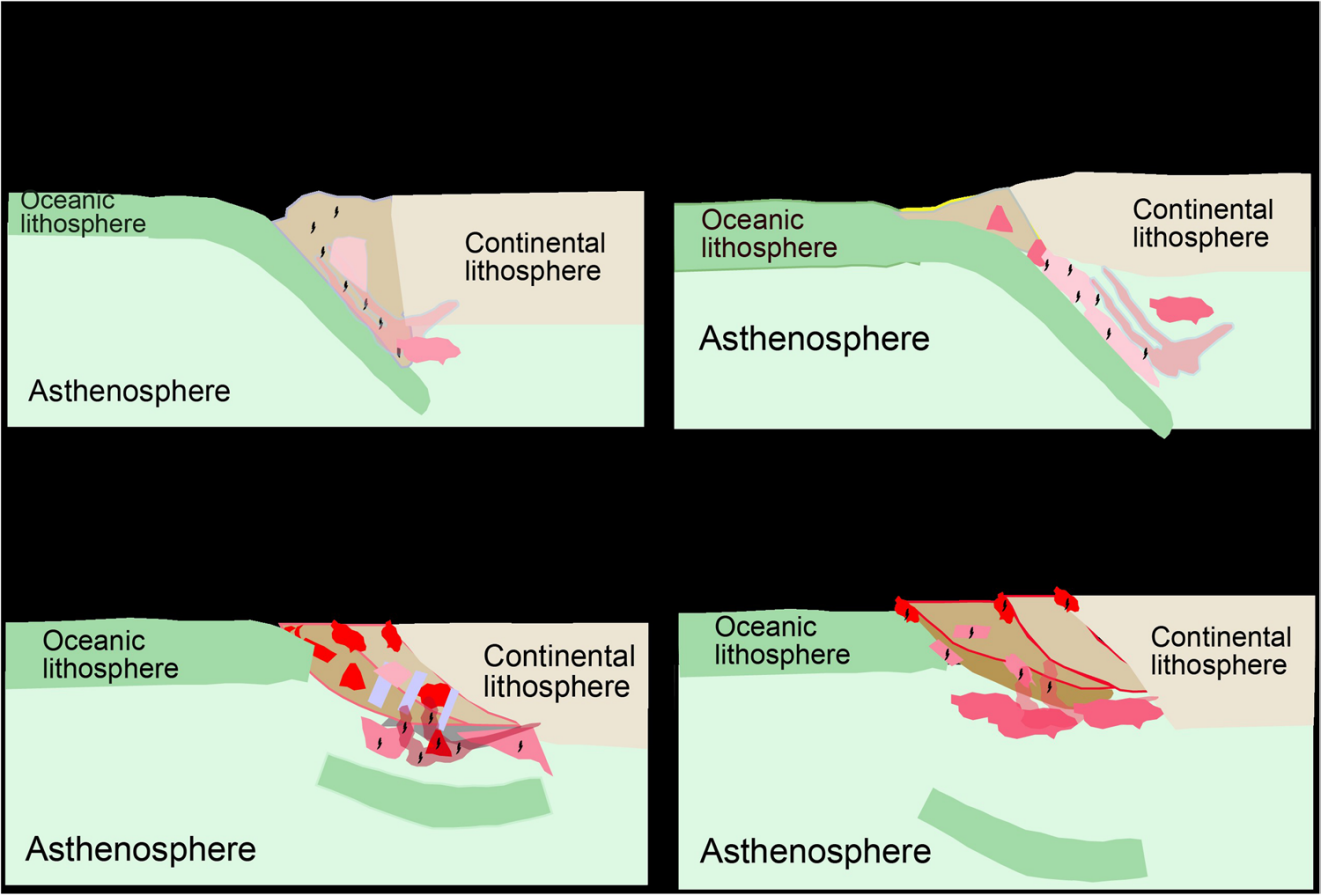
Schematic cartoon showing the origin of relict zircons from Cenozoic continental basalts in east-central China. (**a**) Zircon-bearing detrital sediments were eroded from the continental crust in the Sulu orogen and its adjacent region in the Jurassic. They were transported to the trench overlying the Paleo-Pacific subduction zone, forming the seafloor sediment on the subducting Paleo-Pacific slab. Meanwhile, the lower continental crust offscrapped by subducting Paleo-Pacific oceanic slab brought zircons into the subduction zone. (**b**) Old zircons were carried into mantle metasomatites by felsic melts derived from partial melting of the subducted detrital sediments. (**c**) Thinning and destruction of cratonic lithosphere in the Early Cretaceous leads to the melt-peridotite reaction at the slab-mantle interface in the subcontinental subduction channel, causing growth of new zircons in the mantle metasomatites. (**d**) The mantle metasomatites underwent partial melting due to the extension of continental lithosphere in the Late Cretaceous to Cenozoic, producing basaltic melts. All old and new zircons survived from the partial melting and transported together with the basaltic melts. Relict zircon-bearing basaltic magmas erupted on the surface, giving rise to the continental basalts.

As discussed above, the crustal rocks exposed in the Dabie-Sulu orogenic belt may be the source of detrital zircons in the Cenozoic continental basalts. This is consistent with Mesozoic subduction of the Paleo-Pacific slab beneath the continental edge of eastern China^65^. As such, the crustal rocks would be eroded from the continental margin in the Sulu orogen, delivering the terrigenous sediments into the trench adjacent to the continent margin (Fig. 6a). After hydraulic sorting, tinny zircon grains were retained in the trench and transported into the subcontinental subduction channel. They were transferred into metasomatic mantle domains by hydrous felsic melts derived from partial melting of the subducting terrigenous sediments and their underlying basaltic crust (Fig. 6b). It is possible that the detrital sediment would be only deposited in a limited area close to the Sulu orogen during subduction of the paleo-Pacific slab in the Early Mesozoic.

On the other hand, some basalt zircons show U-Pb ages of younger than Triassic (Tables S1 and S2). This suggests zircon growth after the collisional orogeny in the Triassic. The mantle source of Cenozoic continental basalts in east-central China was generated by reaction of the depleted MORB mantle peridotite with felsic melts derived from partial melting of subducted crustal rocks in the Early Cretaceous^15–17^. In this regard, these youngest relict zircons were not provided by the terrigenous sediments. Instead, they were generated during the melt-peridotite reaction at the slab-mantle interface in the subcontinental subduction channel (Fig. 6c). In this regard, their U-Pb ages record the time of metasomatic reaction during subduction of the Paleo-Pacific slab. The metasomatic agents (aqueous solutions and hydrous melts) released from the subducting Paleo-Pacific crust led to the growth of peritectic zircon in the mantle sources. Finally, the both detrital and peritectic zircons would have partially survived during partial melting of the metasomatic mantle domains in the Cenozoic and erupted together with the basaltic magmas to the surface (Fig. 6d). Rapid ascent of the basaltic magmas allows zircons to survive in the SiO_2_-undersaturated magmas and eventually occur in the Cenozoic continental basalts.

## Electronic supplementary material

Supplementary information

Table S1 to S7
