# Author Correction: Zircon evidence for incorporation of terrigenous sediments into the magma source of continental basalts

**DOI:** 10.1038/s41598-018-35991-3

**Published:** 2018-11-27

**Authors:** Zheng Xu, Yong-Fei Zheng, Zi-Fu Zhao

**Affiliations:** CAS Key Laboratory of Crust-Mantle Materials and Environments, School of Earth and Space Sciences, University of Science and Technology of China, Hefei, 230026 China

Correction to: *Scientific Reports* 10.1038/s41598-017-18549-7, published online 09 January 2018

This Article contains an error in Figure 6 where the white background has been erroneously changed to black, which obscures multiple labels. The correct Figure 6 appears below as Figure 1.

**Figure 1:**
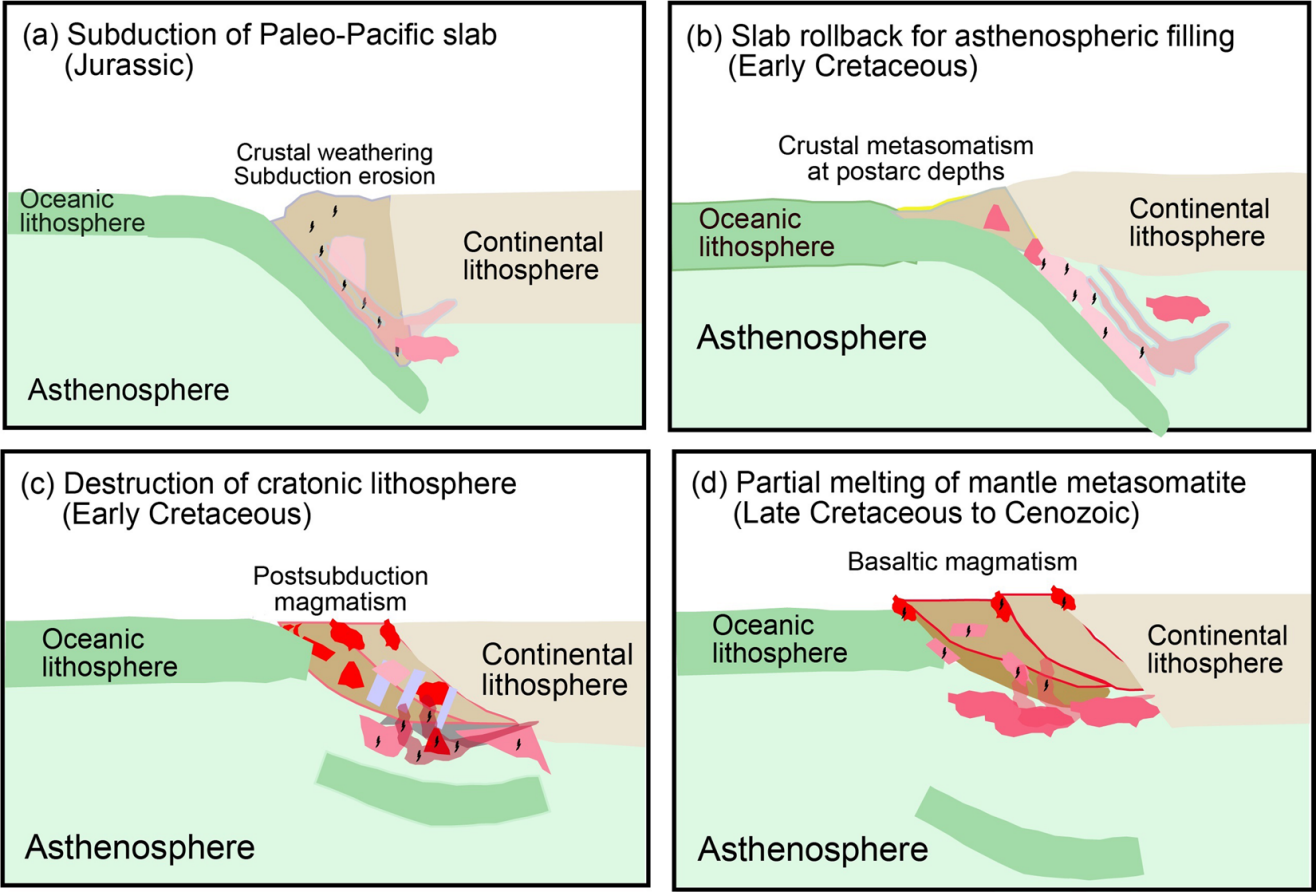
Schematic cartoon showing the origin of relict zircons from Cenozoic continental basalts in east-central China. (**a**) Zircon-bearing detrital sediments were eroded from the continental crust in the Sulu orogen and its adjacent region in the Jurassic. They were transported to the trench overlying the Paleo-Pacific subduction zone, forming the seafloor sediment on the subducting Paleo-Pacific slab. Meanwhile, the lower continental crust offscrapped by subducting Paleo-Pacific oceanic slab brought zircons into the subduction zone. (**b**) Old zircons were carried into mantle metasomatites by felsic melts derived from partial melting of the subducted detrital sediments. (**c**) Thinning and destruction of cratonic lithosphere in the Early Cretaceous leads to the melt-peridotite reaction at the slab-mantle interface in the subcontinental subduction channel, causing growth of new zircons in the mantle metasomatites. (**d**) The mantle metasomatites underwent partial melting due to the extension of continental lithosphere in the Late Cretaceous to Cenozoic, producing basaltic melts. All old and new zircons survived from the partial melting and transported together with the basaltic melts. Relict zircon-bearing basaltic magmas erupted on the surface, giving rise to the continental basalts.

