# Supplementary material for: Zircon evidence for incorporation of terrigenous sediments into the magma source of continental basalts
Source: Sci Rep. 2018 Jan 9;8:178. doi: 10.1038/s41598-017-18549-7 (PMC5760614; doi:10.1038/s41598-017-18549-7)
Supplement: Supplementary file 1 — Supplementary information [file 41598_2017_18549_MOESM1_ESM.doc]

**Zircon evidence for incorporation of terrigenous sediments into the magma source of continental basalts**

Zheng Xu[[1]](#footnote-2)*, Yong-Fei Zheng, Zi-Fu Zhao

CAS Key Laboratory of Crust-Mantle Materials and Environments,

School of Earth and Space Sciences, University of Science and Technology of China,

Hefei 230026, China

**Supplementary Information**

**1. Methods**

Zircon grains were separated by the conventional magnetic and density techniques, and they were selected by handpicking under a binocular microscope. Specific attention was paid to the zircon separation from these basalt samples during the laboratory processing. Then the zircon grains were embedded in epoxy resin and polished to expose the crystals for analyses. Transmitted and reflected light micrographs and cathodoluminescence (CL) images were obtained for the polished zircon grains before U–Pb and Lu–Hf isotope analyses in order to characterize their internal structure and external morphology (mineragraphy) and to select potential analytical spots. The CL images were made on a HITACHI S3000-N Scanning electron microscope at Beijing SHRIMP Center. The working conditions for CL imaging were at 20 kV and 15 nA.

**1.1 SHRIMP U-Pb dating**

Zircon SHRIMP U-Pb dating was performed on SHRIMP II at Beijing SHRIMP Center in Chinese Academy of Geological Sciences. Zircon grains was mounted together with RSES reference zircons SL 13 (with a U-Pb age of 572 Ma, Williams et al., 1996) and TEM (with a U-Pb age of 417 Ma, Black et al., 2003). SL3 was used to calibrate the U, Th and Pb contents whereas TEM was used to correct inter-element fractionation. O- 2 focused 25-30 µm diameter beam under the conditions of 4.5-6.5 nA, 10kV was used in measurement. Each measurement included five scans through Zr, Pb, U and Th isotopes, which were analyzed on a single electronic multiplier by cyclic stepping of magnetic field. Every two zircon sample analyses were intercepted with an analysis of the references, allowing assessment of Pb^+^/U^+^ discrimination. The reproducibility of standard Pb/U ratios was around ±3% (1σ). Data were processed by the PRAWN data reduction software (Williams et al., 1996). Ages were calculated using the constants recommended by Steiger and Jäger (1977). Common Pb was corrected by the approach outlined by Compston et al. (1992), and initial Pb compositions were estimated by the approach of Cumming and Richards (1975).

**1.2 LA-(MC)-ICPMS U-Pb dating, trace elements and Lu-Hf isotopes analyses**

Zircon LA-(MC)-ICPMS analyses for U-Pb ages, trace elements and Lu-Hf isotopes were conducted at State Key Laboratory of Lithospheric Evolution in Institute of Geology and Geophysics, Chinese Academy of Sciences (CAS), Beijing. The detail analytical protocols have been presented by Wu et al. (2006) and Xie et al. (2008). The whole equipment includes a GeoLas Plus laser ablation system which contains a COMPex 102 excimer laser generator with 193 nm wavelength and a laser optical system with a laser beam homogenizing system, a Neptune multiple-collector inductively coupled plasma mass spectrometer (MC-ICPMS) and an Agilent 7500a quadrupole inductively coupled plasma mass spectrometer (Q-ICPMS). Each analysis was performed with beam diameters of 30 to 50 µm, a repetition rate of 8 Hz and a laser power of 15 J/cm^2^. Reference materials are 91500, NIST610, GJ-1, MON-1 and BCR-2. These reference materials were intercepted at the beginning of every twelve analyses, whereas 91500 was intercepted at the end of first six sample spots in these twelve sample spots. References 91500 and GJ-1 were used to calibrate the ^207^Pb/^206^Pb, ^206^Pb/^238^U, ^207^U/^235^U and ^208^Pb/^232^Th ratios, and reference 91500 was used to calibrate Lu-Hf isotopes while the rest references were used to calibrate trace elements.

Zircon LA-(MC)-ICPMS analyses for U-Pb ages, trace elements and Lu-Hf isotopes were also performed for some samples at State Key Laboratory of Continental Dynamics in Northwest University, Xi’an. The detail analytical protocols have been presented by Liu et al. (2007) and Yuan et al. (2008). For in suit U-Pb dating and trace elements analyses, the whole equipment includes a GeoLas 200M laser ablation system which contains a COMPex 102 excimer laser generator with 193 nm wavelength and a laser optical system with a laser beam homogenizing system, a Agilent7500a ICP-MS. Each analysis was conducted with beam diameters of 30 to 50 µm, a repetition rate of 10 Hz and a laser power of 15 J/cm^2^. A Nu Plasma HR MC-ICP-MS connected by GeoLas 2005 laser ablation system which consists COMPex 102 excimer laser generator with 193 nm wavelength and a laser optical system with a laser beam homogenizing system was used for Lu-Hf isotope analyses. Each analysis was conducted with beam diameters of 30 to 50 µm, a 10 Hz repetition rate and a laser power of 15 J/cm^2^. References materials of 91500 and NIST610 with/without Temora-1 were intercepted at the start of every twenty-four sample spots whereas 91500 was analyzed before each six sample spots. References 91500 and Temora-1 were used to monitor the U-Pb dating whereas NISR610 and 91500 were used to calibrate the trace element and Lu-Hf isotope compositions, respectively.

Off-line background and analysis signal selection and integration and U-Pb age and trace element data calibration were performed by software GLIATTER (Macquarie University). U-Pb ages of 1065.4±0.6 Ma for 91500 and 416.50±0.22 Ma for Temora are recommended by Wiedenbeck et al. (1995) and Black et al. (2004), respectively. Common Pb correction was processed by Excel macro ComPbCorr#3_151 (Anderson, 2002), with the assumption that lead loss at a certain age could lead to the observed ^206^Pb/^238^U, ^207^Pb/^235^U and ^208^Pb/^232^Th for a discordant zircon. Trace elements were calibrated using an internal standard ^29^Si and external standard NIST 610. Analytical uncertainties for trace elements are less than ±10% (2σ).

^176^Lu was calculated by measuring uninterfaced ^175^Lu and using a ^176^Lu/^175^Lu ratio of 0.02655 reported in Blichert-Toft et al. (1997), which was used to calibrate the isobaric interface of ^176^Lu on ^176^Hf. ^176^Yb was calculated by measuring uninterfaced ^173^Yb and using a ^176^Yb/^173^Yb of 0.78696 reported in Thirlwall and Anczkiewicz (2004), which was used to calibrate the isobaric interface of ^176^Yb on ^176^Hf. Moreover, β_Yb31_ defined by β_Yb31_ = ln[(^173^Yb/^171^Yb)_mean_/(^173^Yb/^171^Yb)_true_]/ln(M_173Yb_/M_171Yb_) (Iizuka and Hirata, 2005) was used to calibrate ^176^Yb interface on ^176^Hf. Reference zircon 91500 with a recommended ^176^Hf/^177^Hf ratios of 0.282306 (Woodhead et al., 2004) was used as the external standard. All the Lu-Hf isotope compositions are reported with 2σ uncertainties.

Initial ^176^Hf/^177^Hf were calculated using the measured ^176^Lu/^177^Hf ratios and U-Pb ages with a decay constant of 1.865×10^-11^ yr^-1^ for ^176^Lu (Scherer et al., 2001). The ε_Hf_(t) value, which is defined by ε_Hf_(t) = 10000×[(^176^Hf/^177^Hf)_sample_/(^176^Hf/^177^Hf)_CHUR_], was calculated using current ^176^Hf/^177^Hf = 0.282772 and ^176^Lu/^177^Hf = 0.0332 for chondrite^50^. Two-stage Hf model age (T_DM2_) were calculated with the assumption of average crustal ^176^Hf/^177^Hf = 0.015 (Griffin et al., 2002). The ε_Hf_(t) and T_DM2_ are reported with 2σ uncertainties which are calculated based on uncertainties in measured ^176^Hf/^177^Hf, ^176^Hf/^177^Hf and ^176^Lu/^177^Hf ratios.

**2. Sediment and lower continental crust components in the mantle sources of basalts**

Studied basalts are all OIB-type intracontinental basalts with depleted to weekly enriched Sr-Nd isotope compositions (Zhang et al., 2009; Wang et al., 2011; Xu et al., 2012). In the ^206^Pb/^204^Pb vs. Nb/La diagram (Fig. S1), some basalts show the descent of Nb/La with the slightly elevation of ^206^Pb/^204^Pb. This could be attributed to the evolvement of sediment component into the mantle sources. Terrigenous sediment that is mainly from the continental crust show elevated U/Pb comparing to depleted MORB mantle (Plank and Langmuir, 1998; Plank, 2014). Then it will obtain high ^206^Pb/^204^Pb. On the other hand, sediment is characterized by HFSE depletion (Plank and Langmuir, 1998; Plank, 2014) and therefore low Nb/La. As a result, the mixing of felsic melt derived from partial melting of terrigenous sediment and depleted MORB mantle will lead to the descent of Nb/La as well as elevation of ^206^Pb/^204^Pb.

Some basalts from NCC and SCB also construct positive trends between ^206^Pb/^204^Pb and Nb/La (Fig. S1). This could be the result of involvement of lower continental crust (LCC) component in their mantle sources. LCC has lower U/Pb than depleted to mantle (Rudnick and Gao, 2014). As a consequent, it will yield lower ^206^Pb/^204^Pb. Furthermore, LCC also shows lower Nb/La (Rudnick and Gao, 2014). Therefore, mixing with melts derived from partial melting of LCC would lead the coherent descent of ^206^Pb/^204^Pb and Nb/La. Basalts from Sulu orogen, however, do not show such positive correlations, thus LCC component may be absent in their mantle sources.

**
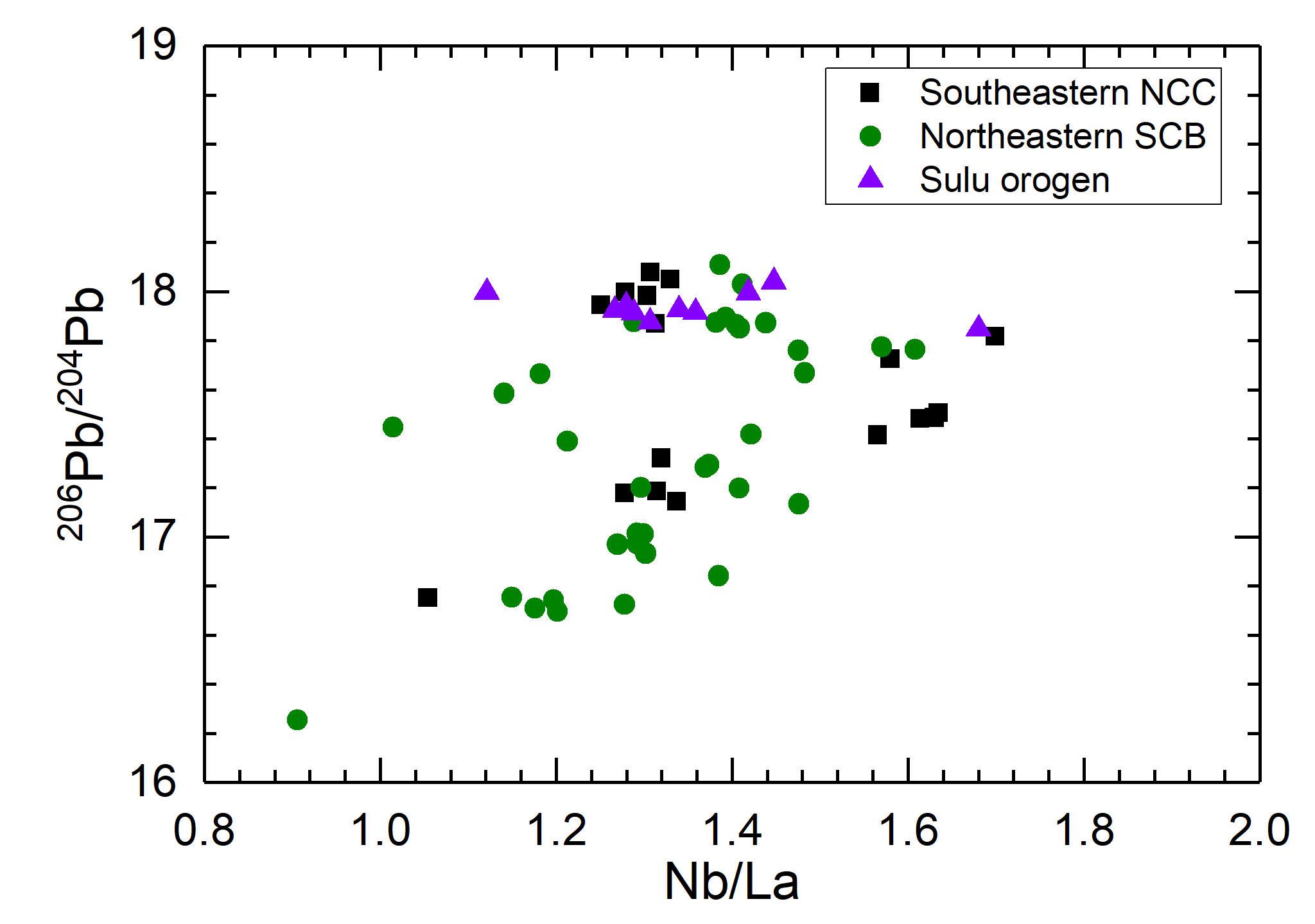
**

Figure S1. ^206^Pb/^204^Pb vs. Nb/La diagram for basalts from studied area. Data are from Table S5.

**3. The occurrence of magmatic zircon in mafic igneous rock**

As an accessory phase, zircon is common in felsic igneous rocks such as granites and rhyolites but less common in mafic igneous rocks such as basalts and gabbros. It is known for a long time that Zr saturation contents in silicate melts at a given temperature is negatively correlated with major element composition (Waterson and Harrison, 1983; Boehnke et al., 2013), which is defined as a combined variable M = (2Ca+K+Na)/(Si×Al). As a result, the felsic melts of high SiO_2_ and Al_2_O_3_ are susceptible to Zr saturation and thus to zircon growth. In contrast, zircon growth from mafic melts is usually considered impossible because the Zr saturation is expected to not achievable in the mafic melts of low SiO_2_ and Al_2_O_3_. According to the revised experiments of Boehnke et al. (2013) for Zr solubility in silicate melts, several hundreds of ppm Zr are needed for zircon growth from felsic melts whereas several thousands of ppm Zr are required for the Zr saturation in mafic melts. Because natural mafic igneous rocks commonly have Zr contents of tens to few hundreds ppm that are much lower than those expected for the Zr saturation, zircon is theoretically not able to either grow from or be stable in natural mafic magmas.

However, a number of field-based geochemical studies do have reported the occurrence of magmatic zircons in mafic igneous rocks such as basalts, gabbros and dolerites (e.g., Grimes et al., 2009; Lissenberg et al., 2009; Portner et al., 2011; Huang et al., 2012; Yang et al., 2012a, 2012b; Zhang et al., 2012; Zhao et al., 2005, 2012; Ding et al., 2013; Li et al., 2013; Dai et al., 2011, 2012, 2014, 2015a, 2015b; Torsvik et al., 2013; Stepanova et al., 2014; Shakerardakani et al., 2015; Cheng et al., 2016). For example, Portner et al. (2011) identified a subset of detrital zircons from the enriched (E-MORB) Macquarie Island ophiolite with elevated U/Yb that overlapped the continental field of Grimes et al. (2007), and, citing complementary enriched mantle-like ɛ_Hf_(t) values, interpreted the zircons as originating from parent melts with variably enriched mantle sources. In particular, old zircons has been recovered from mafic igneous rocks from mid-ocean ridges (Torsvik et al., 2013; Cheng et al., 2016). Such observations indicate that zircons do have survived at temperatures for the generation of MORB, which are derived from decompressional melting of the asthenospheric mantle at temperatures of 1300-1400°C. Furthermore, the old zircons may be inherited from their source rather than captured from an unknown continent crust fragment. On the other hand, arc volcanics above the Caribbean subduction zone in Cuba also contain old zircons that cannot be explained as xenocrysts from the continental crust but from the recycled crustal component in the mantle source (Rojas-Agramonte et al., 2016).


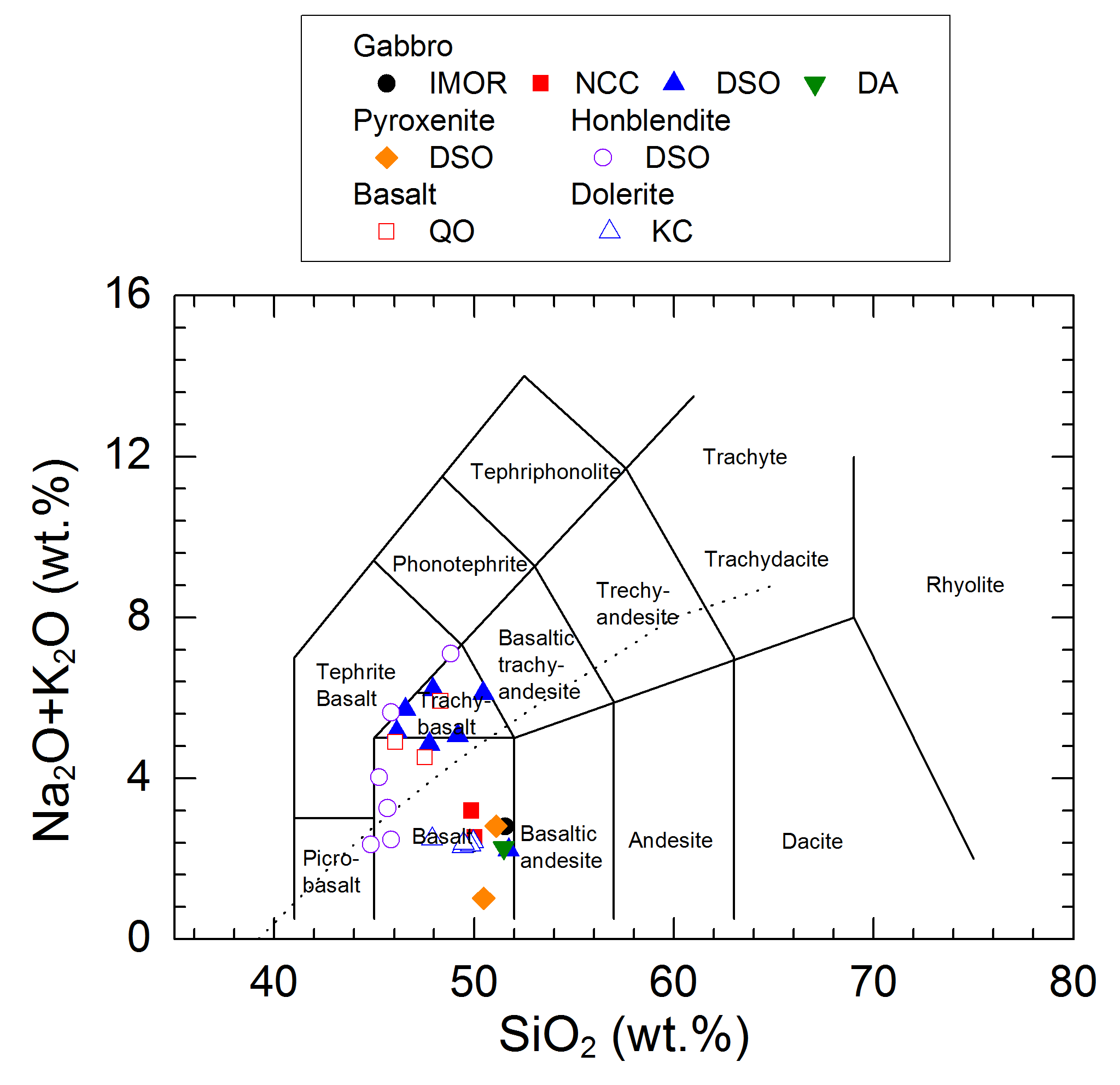


Figure S2. TAS diagram of magmatic zircon-bearing mafic igneous rocks. Data are after Table S5. The framework is after Le Maitre (2002). Abbreviations: IMOR: Indian Mid-Ocean Ridge; NCC: the North China Craton; DSOB: the Dabie-Sulu orogenic belt, China; QO: the Qinling orogen, China; DA: the Dorud–Azna, Iran; KC: the Karelian Craton.

We have made a survey of whole-rock geochemistry for these magmatic zircon-bearing mafic igneous rocks. It appears that these rocks occur not only in convergent plate margins such as collisional orogens but also in divergent plate margins such as Indian mid-ocean ridge. Lithochemically, they include not only alkaline and sub-alkaline rocks but also K_2_O-rich and Na_2_O-rich rocks (Fig. S2 and Table S5). They show MORB-like, arc-like and OIB-like trace element patterns in the primitive mantle-normalized spidergram (Figs. S2 and S3). Therefore, the primary magmatic zircon-bearing mafic igneous rocks are associated neither with specific tectonic settings nor with particular lithochemical compositions. It is notable that the Zr contents of these mafic rocks vary from 25 to 325 ppm (Fig. S5 and Table S5), mostly lower than 100 ppm. Such Zr contents are much lower than those expected for the Zr saturation in mafic magmas. In terms of the zircon U-Pb geochronology and geological occurrences of these mafic igneous rocks, it appears that the magmatic zircon did crystallize from the low Zr mafic melts. Such a natural occurrence demonstrates that zircon did grow from and stabilize in the mafic silicate magmas. As such, the both Zr saturation concentration and zircon stability in mafic melts may be dictated not only by some major elements that are include in the combined variable M but also by other major and trace elements that are not evaluated so far for magmatic zirconology.

**4. Survival of detrital zircons at deep subduction zones**

It is intriguing how the detrital zircons in terrigenous materials would have survived during the following two melting events: (1) dehydration melting of the terrigenous materials at the slab-mantle interface during the westward subduction of Pacific slab in the Mesozoic, and (2) decompressional melting of the metasomatic mantle domains in the Cenozoic. Studies of experimental petrology have revealed that zircon can occur as a residual phase during partial melting of continental rocks (crystalline basement and cover sediment) at 2.5 to 4.5 GPa (e.g., Auzanneau et al., 2006; Hermann and Spandler, 2008). Thus, it is possible for zircon to survive during partial melting of the subducted terrigenous materials and to be transferred into the mantle sources by felsic melts via the melt-peridotite reaction (Zhang et al., 2009; Zheng and Hermann, 2014). Furthemore, the subducted crust may partially melt at temperatures of ≤870-900^o^C (Qian and Hermann, 2013; Mann and Schmidt, 2015; Poli, 2015), which are lower than the closure temperature of 900-1000°C for Pb diffusion in crystalline zircon (Lee et al., 1997; Cherniak and Watson, 2000). As such, the zircon U-Pb radiometric system may not be reset during the partial melting of terrigneous materials at the slab-mantle interface in the oceanic subduction channel.

On the other hand, the detrital zircon could be not stable in mafic melts and thus would be dissolved into these melts. This can be evaluated by using the three-dimensional model of Watson (1996). In terms of this model, the rate of zircon dissolution is related to zircon grain radius, temperature and the difference between saturated and actual concentrations of Zr in silicate melts. Assuming a grain size of zircon is 100 μm, it takes 200 kyr to dissolve the bulk zircon grain at the peridotite solidus when Zr is nearly saturated (e.g., C_saturation_ – C_melt_ = 0.01 ppm) in a mafic melt. According to the average composition of basaltic rocks (e.g., trachybasalt, tholeiite) reported in the studying area (Zou et al., 2000; Chen et al., 2007, 2009; Zhang et al., 2009; Wang et al., 2011; Zeng et al., 2011; Xu et al., 2012) and assuming that partial melting of peridotite mantle started at 1280°C, calculated Zr-saturation concentrations at the peridotite solidus using the revised formula of Boehnke et al. (2013) are ~4000 and ~370 ppm, respectively, for basanitic and other basaltic melts. By using the Zr concentration of studied basalts, the calculated results suggest that only several to tens of years are enough for the complete dissolution of zircon. Anhydrous ultramafic rocks have higher solidi than hydrous ones at 2.5 to 4.5 GPa (Walter, 1998; Herzberg et al., 2000; Hirschmann et al., 2003; Keshav et al., 2004; Tuff et al., 2005; Davis et al., 2011). Peridotite with high H_2_O (> 0.3%) or H_2_O+CO_2_ would melt at lower temperatures than 1100°C at 3.0 GPa (Wallace and Green, 1991; Green and Falloon, 1998; Conceição and Green, 2004; Tumiati et al., 2013). As documented by Xu et al. (2014), the mantle source of Cenozoic continental basalts used in this study is characterized by only 290 ppm (0.029 wt.%) water. In this regard, the solidus of this hydrous source may be higher than 1100°C if it would have a peridotite lithology. However, olivine-poor ultramafic rocks such as pyroxenite have been suggested as the mantle source of Cenozoic continental basalts in east-central China (Zhang et al., 2009; Wang et al., 2011; Xu et al., 2012). In this regard, the temperature of partial melting in the mantle source may be lower than 1100°C (e.g., Hirschmann and Stolper, 1996; Yaxley, 2000). Although the mantle source has been considered as the pyroxene-rich lithology (Zhang et al., 2009; Wang et al., 2011; Xu et al., 2012), the solidus of this fertile source may be still higher than 1000°C as implicated by the difference of ~100°C between hydrous peridotite and Si-poor pyroxenite (Walter, 1998; Herzberg et al., 2000; Hirschmann et al., 2003; Keshav et al., 2004).

We have made estimates of temperatures not only for dehydration melting of the subducting slab beneath the mantle wedge to produce metasomatic agents but also partial melting of the metasomatic mantle domains for basaltic magmatism. The former is based on the phenocryst H_2_O/Ce thermometer of Plank et al. (2009) and Cooper et al. (2012), yielding temperatures of about 940 to 1140°C in terms of the limited analyses on the compositions of clinopyroxene and matrix. The latter is based on the whole-rock thermometers of Albarede (1992) and Herzberg and Asimow (2008), giving temperatures of mainly about 1050 to 1280°C (Table S6) except few samples with higher temperature due to their high MgO contents of >10 wt.%. These temperatures are considerably lower than those of 1300 to 1400°C for decompressional melting of the asthenospheric mantle to produce MORB. As such, the old zircons are able to survive not only during dehydration melting of the subducting crust in the Mesozoic but also during partial melting of the metasomatic mantle domains in the Cenozoic. The calculated Zr-saturation concentrations at this estimated pyroxenite solidus are ~2250 and ~210 ppm, respectively, for basanitic and other basaltic melts. In this case, only several to tens of years are required for the dissolution of zircon. It is possible that the mafic melts would buoyantly rise soon after the mantle melting to undergo considerable cooling. As a consequence, one part of the relict zircon grains would have survived from the dissolution and become stable when the temperature of mafic magmas decreased to 800-850 °C though the other part of the relict zircon grains would be partially to completely dissolved into the mafic magmas.

Because the Zr saturation is much hard to reach in basaltic melts, there is no newly grown zircon from the basalts. This is consistent with our observations from the Cenozoic continental basalts in east-central China. Thus, the common occurrence of old zircons in the continental basalts indicates their survival during partial melting of the metasomatic mantle domains. Nevertheless, it is possible that, when the mantle melting started, the initial melt drops would be very small (e.g., <500 μm) and left the melting zones immediately after their generation (Bea et al., 2001). These melt drops may contain zircons separated from melted rocks. Zircons in these melt drops would be dissolved because of the unsaturation of Si and Zr. However, the melt drops may reach the saturation of Si and Zr before the complete dissolution of zircon because of the limited amount of melt. In this regard, zircons would partially survive in the melt drops. On the other hand, zircons have been found in phlogopite that was the product of crustal metasomatism in wehrlite (Zhang et al., 2005). Thus it is reasonable to assume that the old zircons would be enclosed by metasomatic minerals such as orthopyroxene, clinopyroxene or garnet when they crystallized during the melt-peridotite reaction, preventing the relict zircons to contact with the melt (Bea et al., 2001). This makes the survival of relict zircons during the crustal metasomatism at the slab-mantle interface in the Mesozoic. At last these old zircons were released into basaltic melts after dissolution of host minerals during the mantle melting in the Cenozoic.


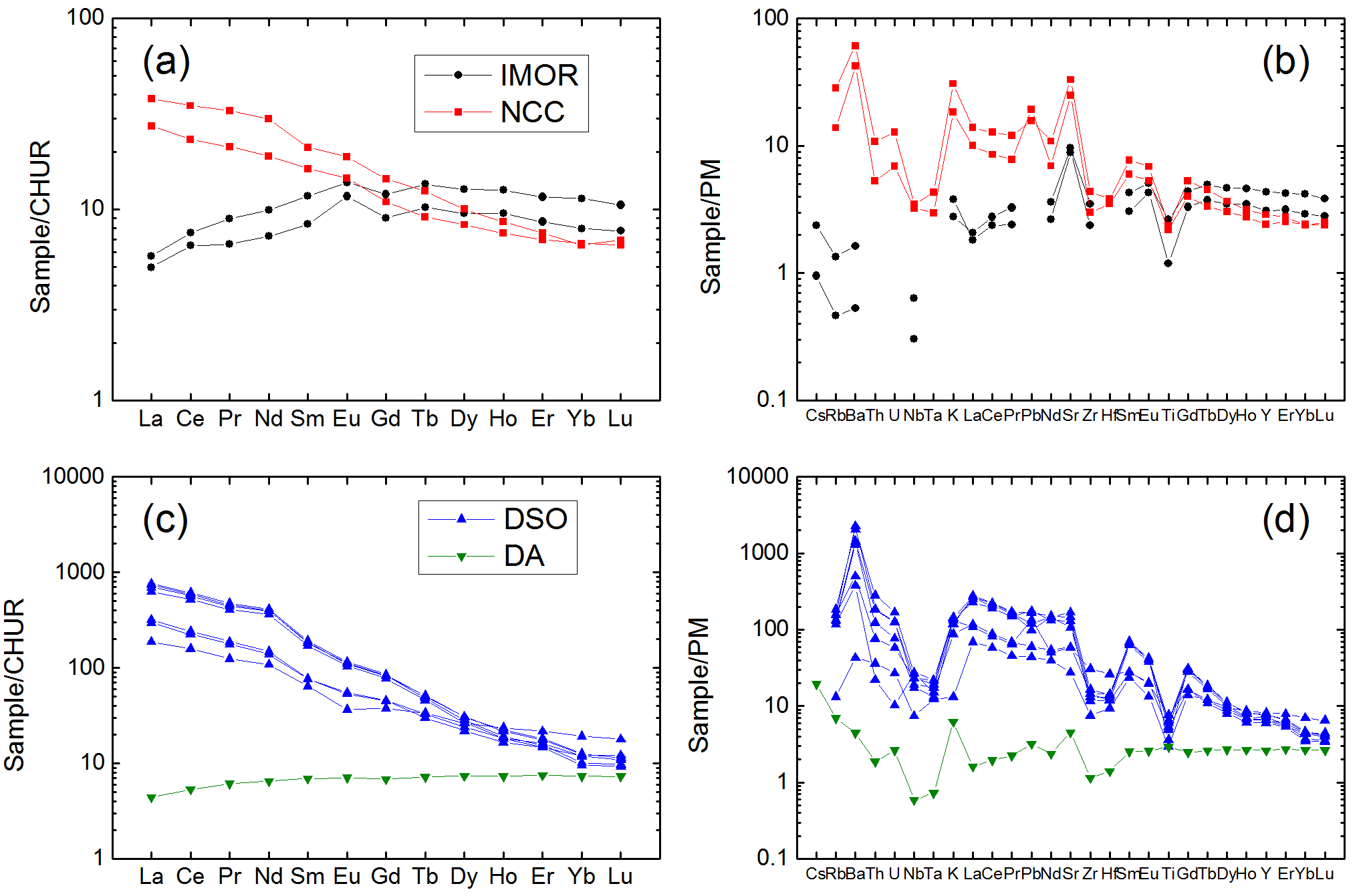


Figure S3. REE and trace element patterns of magmatic zircon-bearing gabbros.

CHUR and PM values are after McDonough and Sun (1995).


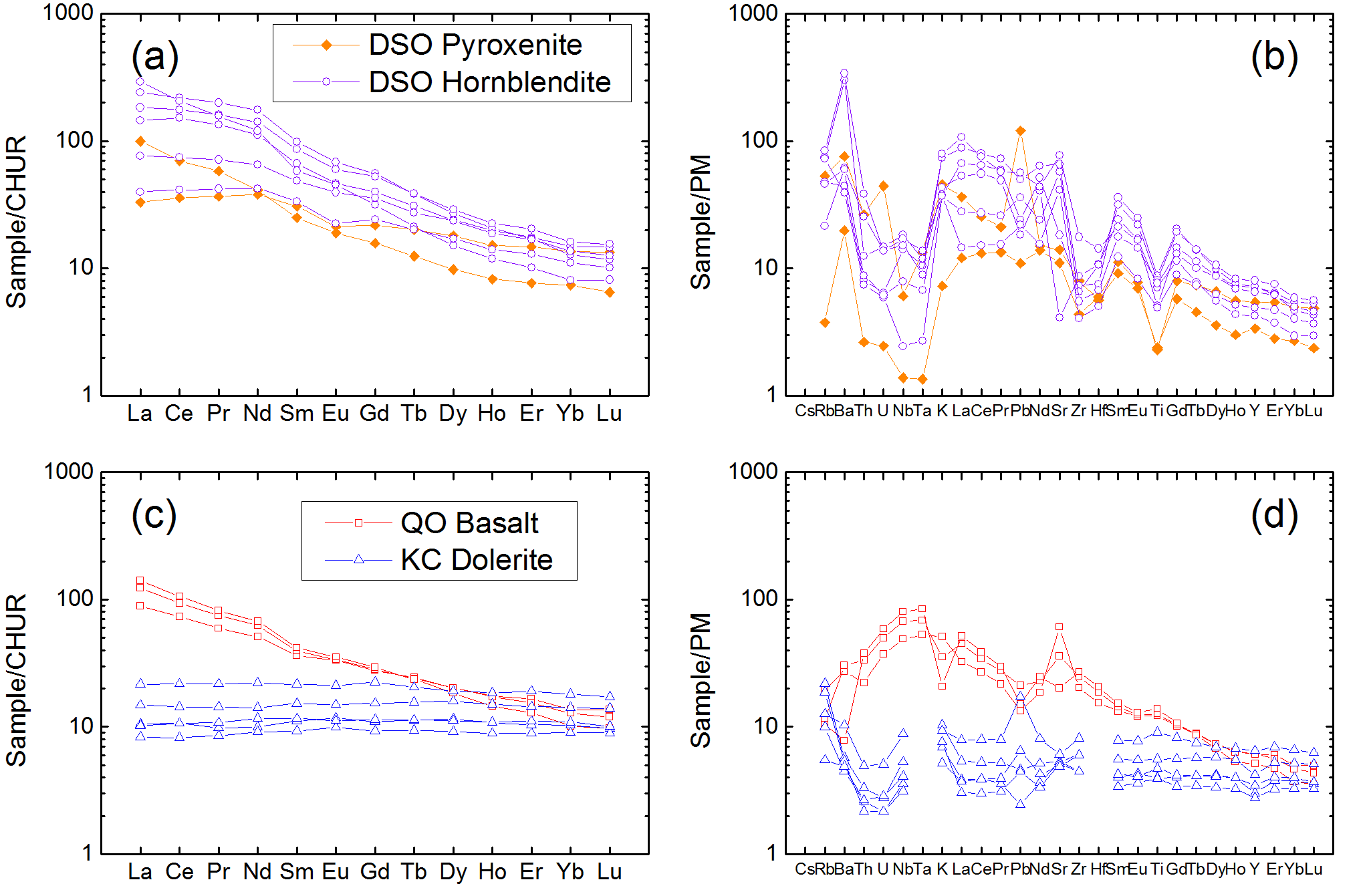


Figure S4. REE and trace element patterns of magmatic zircon-bearing pyroxenites, hornblendites, basalts and dolerites.

CHUR and PM values are after McDonough and Sun (1995).


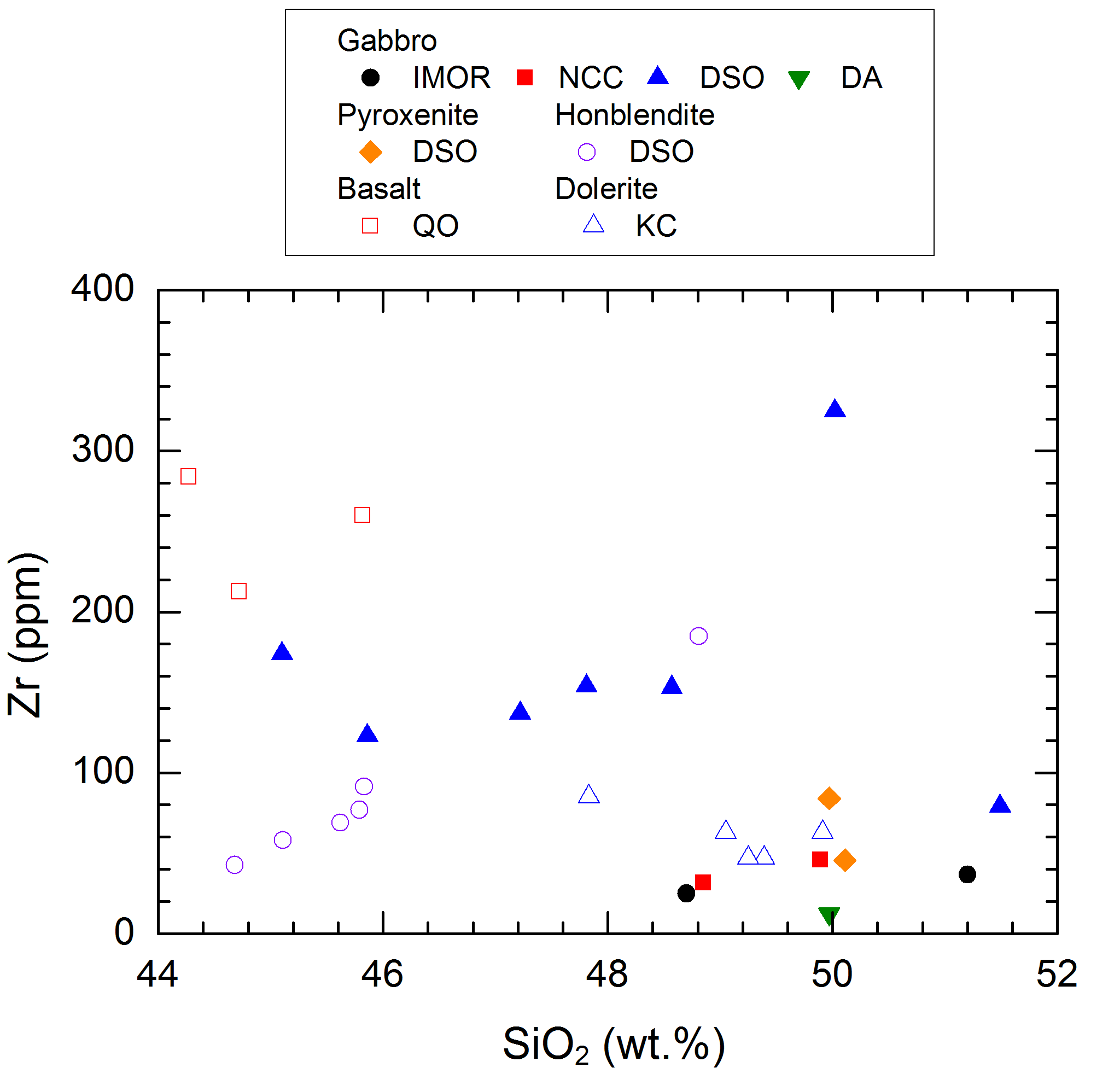


Figure S5. Diagram of whole-rock Zr vs. SiO_2_ contents for magmatic zircon-bearing mafic igneous rocks.

Many zircons from the Cenozoic continental basalts in east-central China exhibit the concordantly old U-Pb ages. This suggests that the zircon U-Pb chronometric system was not reset during the crustal and mantle melting events. Since the radiogenic Pb diffusion in zircon has been found to be much more rapid than U (Cherniak et al., 1997), diffusion is a key factor that affects the U-Pb chronometric system of zircon. Assuming that the mantle melting would take place at 1100°C as discussed above. The maximum age of 1 ka is estimated for the preservation of Pb profile on 10 µm scale which is close to the minimum diameter of zircons with concordant U-Pb ages (Cherniak and Watson, 2003). Thus, the duration of less than 1 ka can be estimated from the beginning of partial melting to the temperature of melt production below the closure temperature of Pb diffusion in zircon. Only in this case, the U-Pb chronometric system could be retained without resetting. In other words, the petrogenesis of Cenozoic continental basalts involves rapid partial melting of the metasomatic mantle domains and subsequent cooling of basaltic magmas.

**5. Metamorphic effects on the REE**

There are different REE distribution patterns for the Cenozoic continental basalts in east-central China (Fig. S6). In the diagram of Ce/Ce* vs. (Yb/La)_n_, all zircons can be categorized into two groups (Fig. S7a).


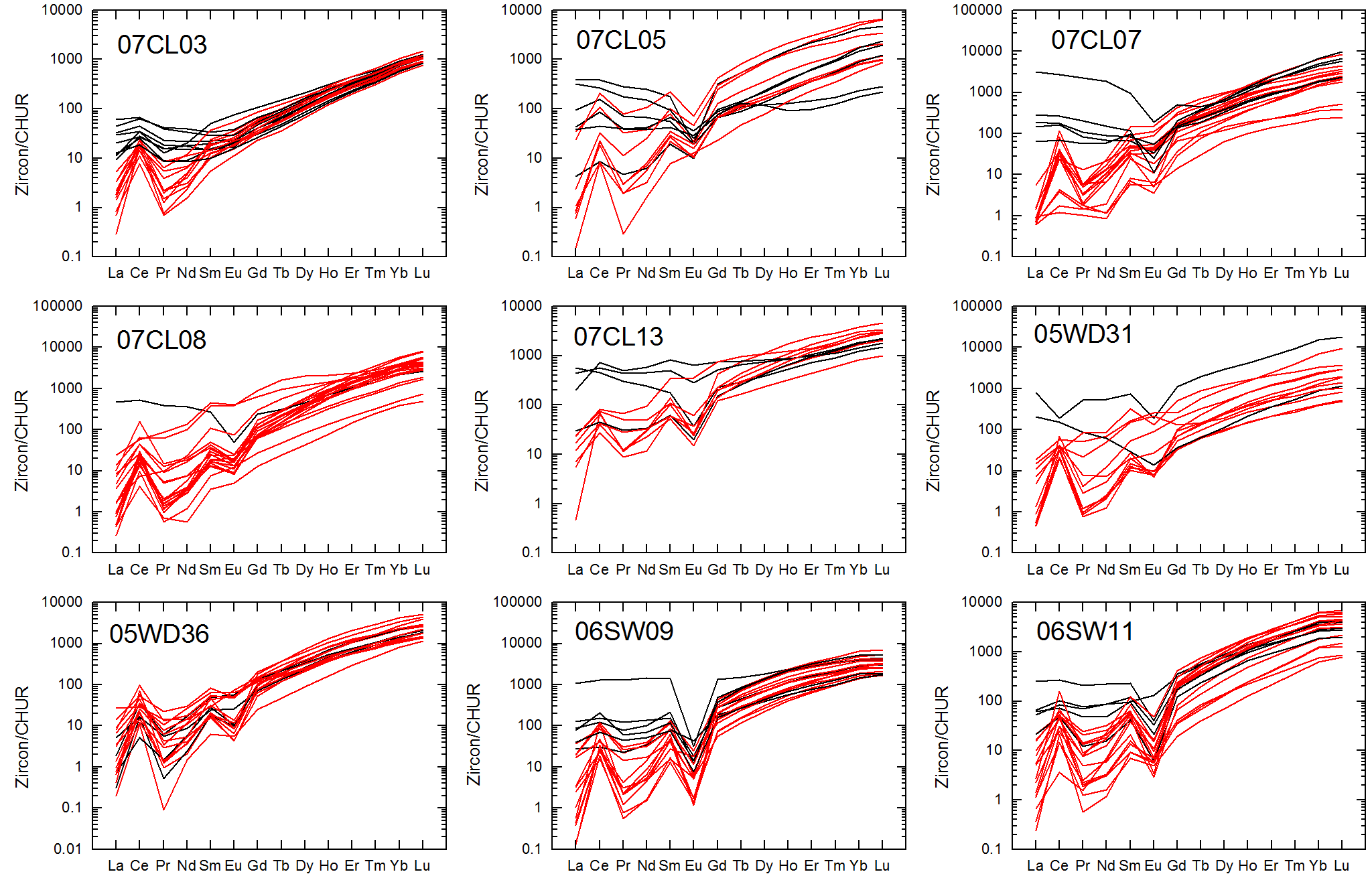


Figure S6. Rare earth element distribution patterns for zircons from Cenozoic continental basalts in east-central China.

Black and red lines denote the Groups I and II zircons, respectively. Chondrite REE values are after McDonough and Sun (1989).


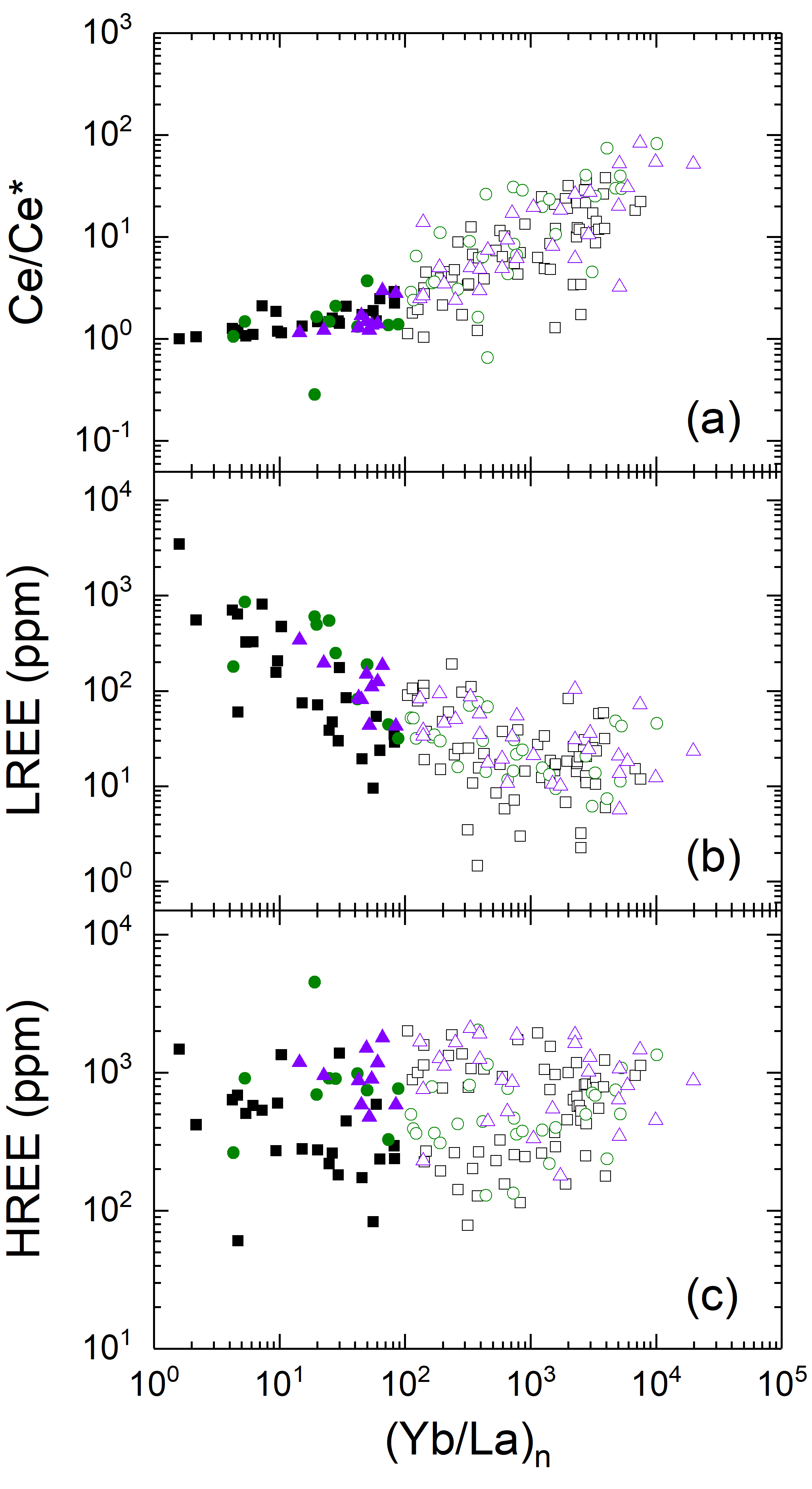


**Figure S7.** Ce/Ce*, LREE and HREE vs. (Yb/La)_n_ diagrams for zircons from Cenozoic continental basalts in east-central China. Data are listed in Table S4. Filled and open symbols denote the Groups I and II zircons, respectively. Square, circle and triangle symbols denote the zircons in basalts from the North China Craton, the South China Block and the Sulu orogen, respectively.

Group I zircons have (Yb/La)_n_ and Ce/Ce* ratios lower than 106 and 3.71, respectively. They show a large variation in (Yb/La)_n_ ratios from 1.6 to 84.5 but a small variation in Ce/Ce* from 0.29 to 3.71 (mostly between 1.00 to 3.00). In contrast, Group II zircons have (Yb/La)_n_ higher than 100 and a large variation in Ce/Ce* from 0.65 to 83.25 (mostly larger than 4.00) with profound positive Ce anomalies. Both groups show positively correlated trends between (Yb/La)_n_ and Ce/Ce* ratios (Fig. 7a). Group I zircons have variable LREE contents from 10 to 3455 ppm, consistently higher than those of 1 to 190 ppm for Group II zircons at the same (Yb/La)n ratios (Fig. S7b). Nevertheless, there is no considerable difference in their HREE contents between Group I and II zircons (60 to 4521 ppm vs. 78 to 2095 ppm) (Fig. S7c). There are also no considerable differences in zircon U-Pb ages and Th/U ratios between the two groups of zircons. Group I and II zircons exhibit similar ranges of U-Pb ages in 129 to 2676 Ma and 101 to 2805 Ma, respectively. Group I zircons have Th/U ratios of 0.05 to 2.23, similar to those of 0.03 to 3.07 for Group II zircons.

The LREE abundances of Group I zircons are higher than those of Group II zircons (Fig. S7b). This could be attributed to the substitution of Zr^4+^ or Zr^4+^ plus Si^4+^ by LREE^3+^ and some particular elements such as P, Nb and Ta (Eskova, 1959; Spear, 1980) or the occurrence of inclusions of some accessory minerals such as monazite, apatite and epidote. However, the latter is not the case for our zircons because these two groups of zircons do not show the differences in some particular elements such as Th and P (Fig. S8), which are very comparable in the above accessory minerals. On the other hand, these zircons may have suffered interaction with fluids and then experienced recrystallization. Thus the property of fluids is a key to the trace element composition of zircons.

The zircons LREE abundances are well negatively correlated with their (Yb/La)_n_ ratios whereas their HREE abundances do not display positive correlations with their (Yb/La)_n_ ratios (Fig. S7). Thus, the increase of (Yb/La)_n_ can be ascribed to the decrease of LREE rather than the increase of HREE. Zircons from samples 06SW09 and 06SW11 show positive correlations between P and LREE (Fig. S8a). This can be explained either by the coupled substitution in that P^5+^ plus LREE^3+^ substitute the Si^4+^ and Zr^4+^ in zircon lattice (Speer, 1982) or by the occurrence of P-bearing LREE-enriched minerals such as monazite (CePO_4_) and apatite [Ca_5_(PO_4_)_3_OH] inclusions in zircon. There are also positive correlations between LREE and Nb+Ta for the zircons (Fig. S8b), consistent with the substitution of Zr^4+^ by LREE^3+^ and Nb^5+^ + Ta^5+^ (Eskova, 1959). On the other hand, LREE contents are positively correlated with Th contents (Fig. S8c). This favors the presence of monazite, apatite and epidote inclusions in zircons, which may exert controls on the enrichment in LREE, because Th^4+^ does not substitute Zr^4+^ together with LREE^3+^ (Frondel, 1953) but is enriched in monazite, apatite and epidote. Epidote does not favor P, so that the occurrence of monazite and apatite inclusions in zircon leads to the enrichment in LREE together with P and Th. If the high LREE contents for Group I zircons compared to Group II zircons are the results of accessory mineral inclusions, they shall also show high P or Th compared to Group II zircons. However, that is not the case for zircons in the Cenozoic basalts (Fig. S8). Therefore, higher LREE contents for Group I zircons relative to Group II zircons may not be attributed to the accessory mineral inclusions. On the other hand, the LREE contents of metasomatic agents may have played a role on the difference in LREE contents between the two groups. For example, HFSE such as Nb, Ta and Hf are less soluble in aqueous fluids and hydrous melts but become soluble in supercritical fluids (e.g., Kogiso et al., 1997; Stalder et al., 1998; Green and Adam, 2003; Kessel et al., 2005). As such, the difference in the property of metasomatic agents may lead to the difference in HFSE contents between metamorphically grown and recrystallized zircons (Xia et al., 2010). However, the general similarity in HFSE contents between Groups I and II zircons excludes the possibility that these zircons were altered by the different properties of metasomatic agents.


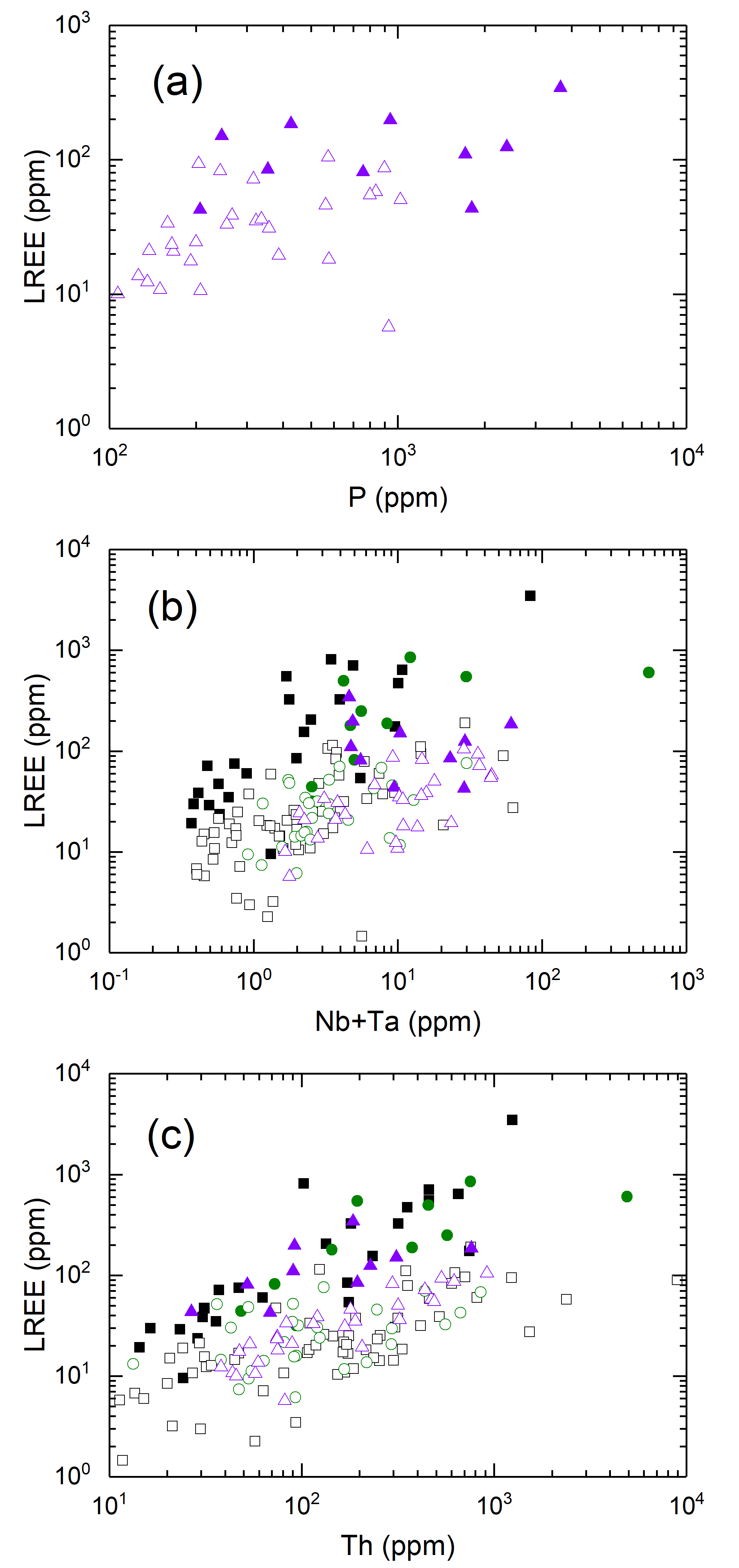


Figure S8. Trace element diagrams for zircons from Cenozoic continental basalts in east-central China. Filled and open symbols denote the Groups I and II zircons, respectively. Square, circle and triangle symbols denote the zircons in basalts from the North China Craton, the South China Block and the Sulu orogen, respectively.

Tetravalent Ce (Ce^4+^) has a cation radius of 0.970 Å and thus is smaller than Ce^3+^ (1.143 Å). Ce^4+^ preferentially substitutes Zr^4+^ in zircon lattice relative to other LREE^3+^ (Schulz et al., 2006). Thus, an oxidized environment would increase Ce^4+^/Ce^3+^ ratio and then increase the content of Ce in zircon to result in a positive Ce anomaly. Likewise, Eu^3+^ (1.066 Å) is smaller than Eu^2+^ (1.250 Å) and is preferentially substitutes Zr^4+^ in zircon lattice relative to other LREE^3+^ (Schulz et al., 2006). A reduced environment would decrease Eu^3+^/Eu^2+^ ratio and then increase the content of Eu in zircon to result in a negative Eu anomaly. If the redox conditions control the Ce and Eu anomalies, Ce/Ce* would show a positive correlation with Eu/Eu*. However, Ce/Ce* ratios are not correlated with Eu/Eu* ratios for the zircons from the Cenozoic continental basalts (Fig. S9). This suggests that the degrees of positive Ce anomalies and/or negative Eu anomalies are controlled by factors rather than the redox state. It is known that Eu^2+^ can substitute Ca^2+^ in plagioclase. Thus fluid/melt in equilibrium with plagioclase would be depleted in Eu. As a result, zircons crystallized from this melt/fluid would exhibit negative Eu anomalies. In this regard, the decoupling between Ce and Eu anomalies could be explained by the presence of plagioclase during zircon crystallization.


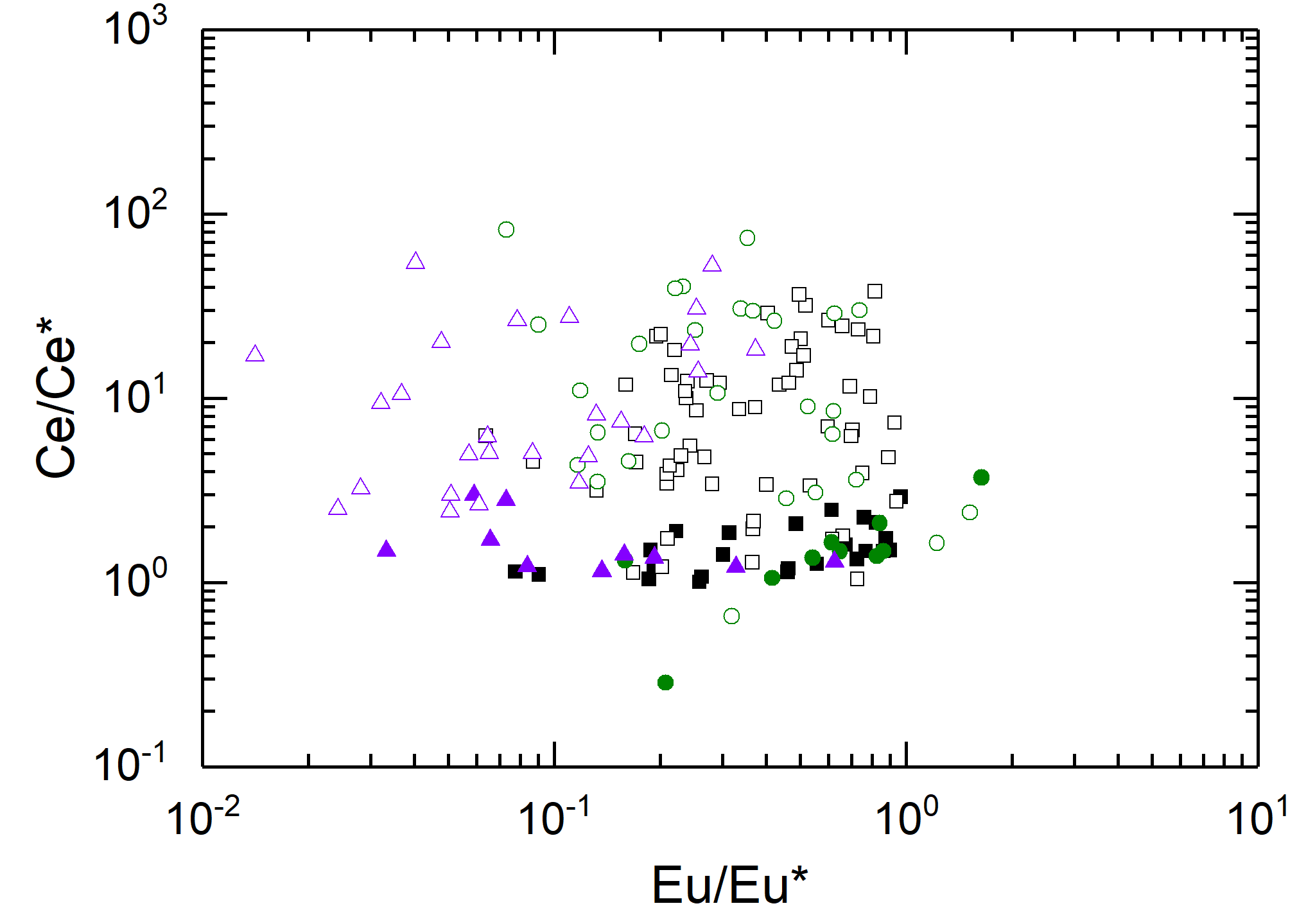


Figure S9. The plot of Ce/Ce* vs. Eu/Eu* ratios for zircons from Cenozoic continental basalts in east-central China. Filled and open symbols denote the Groups I and II zircons, respectively. Square, circle and triangle symbols denote the zircons in basalts from the North China Craton, the South China Block and the Sulu orogen, respectively.

Hoskin et al. (2005) utilized (Yb/La)_n_ and Ce/Ce* ratios to discriminate between magmatic and hydrothermal zircons. They considered zircons with high (Yb/La)_n_ and Ce/Ce* ratios as magmatic origin but zircons with low (Yb/La)_n_ and Ce/Ce* as hydrothermal origin. In our case, however, the both groups contain magmatic zircons which show oscillatory zoning, concordant U-Pb ages and high Th/U ratios (>0.1). In addition, magmatic zircons from kimberlites in Monastery Mine, South Africa and granites in New England, Australia exhibit low (Yb/La)_n_ and Ce/Ce* ratios (Belousova et al., 1998). In this regard, (Yb/La)_n_ and Ce/Ce* ratios may not effectively discriminate between magmatic and hydrothermal zircons. The low (Yb/La)_n_ and Ce/Ce* for magmatic zircons may suggest their crystallization from particular LREE enriched, low fO_2_ melts.

For many Group II zircons that experienced metamorphic recrystallization, it is possible that they were interacted with small amounts of metamorphic fluids. Because the metamorphic fluids have much higher LREE contents than magmatic zircon, LREE in the metamorphically recrystallized zircons would be controlled by the composition of metamorphic fluids. On the other hand, if the oxygen fugacity of metamorphic fluids is low, their Ce^4+^/Ce^3+^ ratios would be low. As a result, positive Ce anomalies in the metamorphically recrystallized zircons would be less significant than those in unaltered magmatic zircons. In this regard, the interaction between magmatic zircon and metamorphic fluids would lead to an increase of LREE but decreases of (Yb/La)_n_ and Ce/Ce* ratios in the metasomatized zircons. When the amount of metamorphic fluids is large, the LREE abundance of metamorphically recrystallized zircons would be governed by the composition of metamorphic fluids. Although (Yb/La)_n_ would still decrease during the interaction between zircon and metamorphic fluids, zircon Ce^4+^/Ce^3+^ ratios would show a small variation. Then zircon Ce/Ce* ratios also show a small variation. Both two groups of zircons show dispersive but similar HFSE contents, suggesting that their HFSE may be controlled by zircon itself because they are enriched in zircon (Hoskin and Schaltegger, 2003).

**References**

Albarede, F., 1992. How deep do common basaltic magmas form and differentiate? Journal of Geophysical Research 97, 10997–11009.

Andersen, T., 2002. Correction of common lead in U–Pb analyses that do not report ^204^Pb. Chemical Geology 192, 59-79.

Auzanneau, E., Vielzeuf, D., Schmidt, M.W., 2006. Experimental evidence of decompression melting during exhumation of subducted continental crust. Contributions to Mineralogy and Petrology 152, 125-148.

Bea, F., Fershtater, G.B., Montero, P., Whitehouse, M., Levin, V.Y., Scarrow, J.H., Austrheim, H., Pushkariev, E.V., 2001. Recycling of continental crust into the mantle as revealed by Kytlym dunite zircons, Ural Mts, Russia. Terra Nova 13, 407-412.

Black, L.P., Kamo, S.L., Allen, C.M., Aleinikoff, J.N., Davis, D.W., Korsch, R.J., Foudoulis, C., 2003. TEMORA 1: a new zircon standard for Phanerozoic U–Pb geochronology. Chemical Geology 200, 155-170.

Black, L.P., Kamo, S.L., Allen, C.M., Davis, D.W., Aleinikoff, J.N., Valley, J.W., Mundil, R., Campbell, I.H., Korsch, R.J., Williams, I.S., Foudoulis, C., 2004. Improved ^206^Pb/^238^U microprobe geochronology by the monitoring of a trace-element-related matrix effect; SHRIMP, ID–TIMS, ELA–ICP–MS and oxygen isotope documentation for a series of zircon standards. Chemical Geology 205, 115-140.

Blichert-Toft, J., Albarède, F., 1997. The Lu-Hf isotope geochemistry of chondrites and the evolution of the mantle-crust system. Earth and Planetary Science Letters 148, 243-258.

Boehnke, P., Watson, E.B., Trail, D., Harrison, T.M., Schmitt, A.K., 2013. Zircon saturation re-revisited. Chemical Geology 351, 324-334.

Chen, Y., Zhang, Y.-X., Graham, D., Su, S.-G., Deng, J.-F., 2007. Geochemistry of Cenozoic basalts and mantle xenoliths in Northeast China. Lithos 96, 108-126.

Chen, L.H., Zeng, G., Jiang, S.Y., Hofmann, A.W., Xu, X.S., Pan, M.B., 2009. Sources of Anfengshan basalts: Subducted lower crust in the Sulu UHP belt, China. Earth and Planetary Science Letters 286, 426-435.

Cheng, H., Zhou, H., Yang, Q., Zhang, L., Ji, F., Dick, H., 2016. Jurassic zircons from the Southwest Indian Ridge. Scientific Reports 6, 26260; doi:10.1038/srep26260.

Cherniak, D.J., Hanchar, J.M., Watson, E.B., 1997. Diffusion of tetravalent cations in zircon. Contributions to Mineralogy and Petrology 127, 383-390.

Cherniak, D.J., Watson, E.B., 2000. Pb diffusion in zircon. Chemical Geology 172, 5-24.

Cherniak, D.J., Watson, E.B., 2003. Diffusion in zircon. Reviews in Mineralogy and Geochemistry 53, 113-143.

Compston, W., Williams, I.S., Kirschvink, J.L., Zhang, Z.-C., Guogan, M.A., 1992. Zircon U-Pb ages for the Early Cambrian time-scale. Journal of the Geological Society 149, 171-184.

Conceição, R.V., Green, D.H., 2004. Derivation of potassic (shoshonitic) magmas by decompression melting of phlogopite+pargasite lherzolite. Lithos 72, 209-229.

Cooper, L.B., Ruscitto, D.M., Plank, T., Wallace P.J., Syracuse, E.M., Manning, C.E., 2012. Global variations in H_2_O/Ce: 1. Slab surface temperatures beneath volcanic arcs. Geochemistry Geophysics Geosystems 13, Q03024; doi:10.1029/2011GC003902.

Cumming, G.L., Richards, J.R., 1975. Ore lead isotope ratios in a continuously changing earth. Earth and Planetary Science Letters 28, 155-171.

Dai, L.-Q., Zhao, Z.-F., Zheng, Y.-F., Li, Q., Yang, Y., Dai, M., 2011. Zircon Hf-O isotope evidence for crust–mantle interaction during continental deep subduction. Earth and Planetary Science Letters 308, 224-244.

Dai, L.-Q., Zhao, Z.-F., Zheng, Y.-F., Zhang, J., 2012. The nature of orogenic lithospheric mantle: Geochemical constraints from postcollisional mafic–ultramafic rocks in the Dabie orogen. Chemical Geology 334, 99-121.

Dai, L.-Q., Zhao, Z.-F., Zheng, Y.-F., 2014. Geochemical insights into the role of metasomatic hornblendite in generating alkali basalts. Geochemistry, Geophysics, Geosystems 15, 3762-3779.

Dai, L.-Q., Zhao, Z.-F., Zheng, Y.-F., 2015a. Tectonic development from oceanic subduction to continental collision: Geochemical evidence from postcollisional mafic rocks in the Hong'an–Dabie orogens. Gondwana Research 27, 1236-1254.

Dai, L.-Q., Zhao, Z.-F., Zheng, Y.-F., Zhang, J., 2015b. Source and magma mixing processes in continental subduction factory: Geochemical evidence from postcollisional mafic igneous rocks in the Dabie orogen. Geochemistry, Geophysics, Geosystems 16, 659-680.

Davis, F.A., Hirschmann, M.M., Humayun, M., 2011. The composition of the incipient partial melt of garnet peridotite at 3 GPa and the origin of OIB. Earth and Planetary Science Letters 308, 380-390.

Eskova, E.M.. 1959. Geochemistry of Nb and Ta in the nepheline syenite massifs of the Vishnevyie Mountains. Geokhimiya 2, 130-139.

Green, D.H., Falloon, T.J., 1998. Pyrolite: A Ringwood concept and its current expression. In: I. Jackson (ed.), The Earth’s Mantle. Cambridge University Press, Cambridge, pp. 311–378.

Griffin, W.L., Wang, X., Jackson, S.E., Pearson, N.J., O'Reilly, S.Y., Xu, X., Zhou, X., 2002. Zircon chemistry and magma mixing, SE China: In-situ analysis of Hf isotopes, Tonglu and Pingtan igneous complexes. Lithos 61, 237-269.

Grimes, C.B., John, B.E., Kelemen, P.B., Mazdab, F., Wooden, J.L., Cheadle, M.J., Hanghoj, K., Schwartz, J.J., 2007. The trace element chemistry of zircons from oceanic crust: a method for distinguishing detrital zircon provenance. Geology 35, 643–646.

Grimes, C.B., John, B.E., Cheadle, M.J., Mazdab, F.K., Wooden, J.L., Swapp, S., Schwartz, J.J., 2009. On the occurrence, trace element geochemistry, and crystallization history of zircon from in situ ocean lithosphere. Contributions to Mineralogy and Petrology 158, 757–783.

Hermann, J., Spandler, C.J., 2008. Sediment melts at sub-arc depths: an experimental study. Journal of Petrology 49, 717-740.

Herzberg, C., Raterron, P., Zhang, J., 2000. New experimental observations on the anhydrous solidus for peridotite KLB-1. Geochemistry Geophysics Geosystems 1, 1051.

Herzberg, C., Asimow, P.D., 2008. Petrology of some oceanic island basalts: PRIMELT2.XLS software for primary magma calculation. Geochemistry Geophysics Geosystems 9, Q09001.

Hirschmann, M.M., Stolper, E.M., 1996. A possible role for garnet pyroxenite in the origin of the “garnet signature” in MORB. Contributions to Mineralogy and Petrology 124, 185–208.

Hirschmann, M.M., Kogiso, T., Baker, M.B., Stolper, E.M., 2003. Alkalic magmas generated by partial melting of garnet pyroxenite. Geology 31, 481-484.

Huang, X.-L., Zhong, J.-W., Xu, Y.-G., 2012. Two tales of the continental lithospheric mantle prior to the destruction of the North China Craton: Insights from Early Cretaceous mafic intrusions in western Shandong, East China. Geochimica et Cosmochimica Acta 96, 193-214.

Iizuka, T., Hirata, T., 2005. Improvements of precision and accuracy in in situ Hf isotope microanalysis of zircon using the laser ablation-MC-ICPMS technique. Chemical Geology 220, 121-137.

Iwamori, H., McKenzie, D., Takahashi, E., 1995. Melt generation by isentropic mantle upwelling. Earth and Planetary Science Letters 134, 253-266.

Keshav, S., Gudfinnsson, G.H., Sen, G., Fei, Y.-W., 2004. High-pressure melting experiments on garnet clinopyroxenite and the alkalic to tholefitic transition in ocean-island basalts. Earth and Planetary Science Letters 223, 365-379.

Le Maitre, R.W., 2002. Igneous Rocks A Classification and Glossary of Terms, 2nd ed. Cambridge University Press, Cambridge.

Lee, J.K.W., Williams, I.S., Ellis, D.J., 1997. Pb, U and Th diffusion in natural zircon. Nature 390, 159-162.

Li, X.-W., Mo, X.-X., Yu, X.-H., Ding, Y., Huang, X.-F., Wei, P., He, W.-Y., 2013. Geochronological, geochemical and Sr–Nd–Hf isotopic constraints on the origin of the Cretaceous intraplate volcanism in West Qinling, Central China: Implications for asthenosphere–lithosphere interaction. Lithos 177, 381-401.

Lissenberg, C.J., Rioux, M., Shimizu, N., Bowring, S.A., Mével, C., 2009. Zircon dating of oceanic crustal accretion. Science 323, 1048-1050.

Liu, X., Gao, S., Diwu, C., Yuan, H., Hu, Z., 2007. Simultaneous in-situ determination of U-Pb age and trace elements in zircon by LA-ICP-MS in 20 μm spot size. Chinese Science Bulletin 52, 1257-1264.

McDonough, W.F., Sun, S.-S., 1995. The Composition of the Earth. Chemical Geology 120, 223-253.

Mann, U., Schmidt, M.W., 2015. Melting of pelitic sediments at subarc depths: 1. Flux vs. fluid-absent melting and a parameterization of melt productivity. Chemical Geology 404, 150-167.

Ping, X., Zheng, J., Tang, H., Xiong, Q., Su, Y., 2015. Paleoproterozoic multistage evolution of the lower crust beneath the southern North China Craton. Precambrian Research 269, 162-182.

Plank, T., 2014. The Chemical Composition of Subducting Sediments, in: Turekian, H.D.H.K. (Ed.), Treatise on Geochemistry (Second Edition). Elsevier, Oxford, pp. 607-629.

Plank, T., Langmuir, C.H., 1998. The chemical composition of subducting sediment and its consequences for the crust and mantle. Chemical Geology 145, 325-394.

Plank, T., Cooper, L.B., Manning, C.E., 2009. Emerging geothermometers for estimating slab surface temperatures. Nature Geoscience 2, 611-615.

Poli, S., 2015. Carbon mobilized at shallow depths in subduction zones by carbonatitic liquids. Nature Geoscience 8, 633-636.

Portner, R.A., Daczko, N.R., Murphy, M.J., Pearson, N.J., 2011. Enriching mantle melts within a dying mid-ocean spreading ridge: insights from Hf-isotope and trace element patterns in detrital oceanic zircon. Lithos 126, 355–368.

Qian, Q., Hermann, J., 2013. Partial melting of lower crust at 10–15 kbar: constraints on adakite and TTG formation. Contributions to Mineralogy and Petrology 165, 1195-1224.

Rojas-Agramonte, Y., Garcia-Casco, A., Kemp, A., Kröner, A., Proenza, J.A., Lázaro, C., Liu, D., 2016. Recycling and transport of continental material through the mantle wedge above subduction zones: A Caribbean example. Earth and Planetary Science Letters 436, 93-107.

Rudnick, R.L., Gao, S., 2014. Composition of the continental crust. Treatise on Geochemistry 4, 1-51.

Scherer, E., Münker, C., Mezger, K., 2001. Calibration of the Lutetium-Hafnium Clock. Science 293, 683-687.

Shakerardakani, F., Neubauer, F., Masoudi, F., Mehrabi, B., Liu, X., Dong, Y., Mohajjel, M., Monfaredi, B., Friedl, G., 2015. Panafrican basement and Mesozoic gabbro in the Zagros orogenic belt in the Dorud–Azna region (NW Iran): Laser-ablation ICP–MS zircon ages and geochemistry. Tectonophysics 647–648, 146-171.

Speer, J. A., 1980. Zircon. Rev Mineral Geochem 5, 67-112.

Steiger, R.H., Jäger, E., 1977. Subcommission on geochronology: Convention on the use of decay constants in geo- and cosmochronology. Earth and Planetary Science Letters 36, 359-362..

Stepanova, A.V., Samsonov, A.V., Salnikova, E.B., Puchtel, I.S., Larionova, Y.O., Larionov, A.N., Stepanov, V.S., Shapovalov, Y.B., Egorova, S.V., 2014. Palaeoproterozoic Continental MORB-type Tholeiites in the Karelian Craton: Petrology, Geochronology, and Tectonic Setting. Journal of Petrology 55, 1719-1751.

Tang, Y.-J., Zhang, H.-F., Ying, J.-F., 2006. Asthenosphere–lithospheric mantle interaction in an extensional regime: Implication from the geochemistry of Cenozoic basalts from Taihang Mountains, North China Craton. Chemical Geology 233, 309-327.

Thirlwall, M.F., Anczkiewicz, R., 2004. Multidynamic isotope ratio analysis using MC–ICP–MS and the causes of secular drift in Hf, Nd and Pb isotope ratios. International Journal of Mass Spectrometry 235, 59-81.

Torsvik, T.H., Amundsen, H., Hartz, E.H., Corfu, F., Kusznir, N., Gaina, C., Doubrovine, P.V., Steinberger, B., Ashwal, L.D., Jamtveit, B., 2013. A Precambrian microcontinent in the Indian Ocean. Nature Geoscience 6, 223-227.

Tuff, J., Takahashi, E., Gibson, S.A., 2005. Experimental constraints on the role of garnet pyroxenite in the genesis of high-Fe mantle plume derived melts. Journal of Petrology 46, 2023-2058.

Tumiati, S., Fumagalli, P., Tiraboschi, C., Poli, S., 2013. An experimental study on COH-bearing peridotite up to 3.2 GPa and implications for crust–mantle recycling. Journal of Petrology 54, 453-479.

Wallace, M., Green, D.H., 1991. The effect of bulk rock composition on the stability of amphibole in the upper mantle: Implications for solidus positions and mantle metasomatism. Mineralogy and Petrology 44, 1-19.

Walter, M.J., 1998. Melting of garnet peridotite and the origin of komatiite and depleted lithosphere. Journal of Petrology 39, 29-60.

Wang, Y., Zhao, Z.-F., Zheng, Y.-F., Zhang, J.-J., 2011. Geochemical constraints on the nature of mantle source for Cenozoic continental basalts in east-central China. Lithos 125, 940-955.

Watson, E.B., 1996. Dissolution, growth and survival of zircons during crustal fusion: kinetic principles, geological models and implications for isotopic inheritance. Transactions of the Royal Society of Edinburgh: Earth Sciences 87, 43-56.

Wiedenbeck, M., AllÉ, P., Corfu, F., Griffin, W.L., Meier, M., Oberli, F., Quadt, A.V., Roddick, J.C., Spiegel, W., 1995. Three natural zircon standards for U-Th-Pb, Lu-Hf, trace element and REE analyses. Geostandards Newsletter 19, 1-23.

Williams, I.S., Buick, I.S., Cartwright, I., 1996. An extended episode of early Mesoproterozoic metamorphic fluid flow in the Reynolds Range, central Australia. Journal of Metamorphic Geology 14, 29-47.

Woodhead, J., Hergt, J., Shelley, M., Eggins, S., Kemp, R., 2004. Zircon Hf-isotope analysis with an excimer laser, depth profiling, ablation of complex geometries, and concomitant age estimation. Chemical Geology 209, 121-135.

Wu, F.-Y., Yang, Y.-H., Xie, L.-W., Yang, J.-H., Xu, P., 2006. Hf isotopic compositions of the standard zircons and baddeleyites used in U–Pb geochronology. Chemical Geology 234, 105-126.

Xia, Q.-X., Zheng, Y.-F., Hu, Z., 2010. Trace elements in zircon and coexisting minerals from low-T/UHP metagranite in the Dabie orogen: Implications for action of supercritical fluid during continental subduction-zone metamorphism. Lithos 114, 385-412.

Xie, L.-W., Zhang, Y.-B., Zhang, H.-H., Sun, J.-F., Wu, F.-Y., 2008. In situ simultaneous determination of trace elements, U-Pb and Lu-Hf isotopes in zircon and baddeleyite. Chinese Science Bulletin 53, 1565-1573.

Xu, Z., Zhao, Z.-F., Zheng, Y.-F., 2012. Slab–mantle interaction for thinning of cratonic lithospheric mantle in North China: Geochemical evidence from Cenozoic continental basalts in central Shandong. Lithos 146–147, 202-217.

Xu, Z., Zheng, Y.-F., Zhao, Z.-F., Gong, B., 2014. The hydrous properties of subcontinental lithospheric mantle: Constraints from water content and hydrogen isotope composition of phenocrysts from Cenozoic continental basalt in North China. Geochimica et Cosmochimica Acta 143, 285-302.

Yang, Q.-L., Zhao, Z.-F., Zheng, Y.-F., 2012a. Modification of subcontinental lithospheric mantle above continental subduction zone: Constraints from geochemistry of Mesozoic gabbroic rocks in southeastern North China. Lithos 146–147, 164-182.

Yang, Q.-L., Zhao, Z.-F., Zheng, Y.-F., 2012b. Slab–mantle interaction in continental subduction channel: Geochemical evidence from Mesozoic gabbroic intrusives in southeastern North China. Lithos 155, 442-460.

Yaxley, G.M., 2000. Experimental study of the phase and melting relations of homogeneous basalt1peridotite mixtures and implications for the petrogenesis of flood basalts. Contributions to Mineralogy and Petrology 139, 326–338.

Yuan, H.-L., Gao, S., Dai, M.-N., Zong, C.-L., Günther, D., Fontaine, G.H., Liu, X.-M., Diwu, C., 2008. Simultaneous determinations of U–Pb age, Hf isotopes and trace element compositions of zircon by excimer laser-ablation quadrupole and multiple-collector ICP-MS. Chemical Geology 247, 100-118.

Zeng, G., Chen, L.-H., Hofmann, A.W., Jiang, S.-Y., Xu, X.-S., 2011. Crust recycling in the sources of two parallel volcanic chains in Shandong, North China. Earth and Planetary Science Letters 302, 359-368.

Zhang, J.-J., Zheng, Y.-F., Zhao, Z.-F., 2009. Geochemical evidence for interaction between oceanic crust and lithospheric mantle in the origin of Cenozoic continental basalts in east-central China. Lithos 110, 305-326.

Zhang, J., Zhao, Z.-F., Zheng, Y.-F., Dai, M.-N., 2010. Postcollisional magmatism: Geochemical constraints on the petrogenesis of Mesozoic granitoids in the Sulu orogen, China. Lithos 119, 512-536.

Zhang, J., Zhao, Z.-F., Zheng, Y.-F., Liu, X.-M., Xie, L.-W., 2012. Zircon Hf–O isotope and whole-rock geochemical constraints on origin of postcollisional mafic to felsic dykes in the Sulu orogen. Lithos 136–139, 225-245.

Zhao, Z.-F., Zheng, Y.-F., Wei, C.-S., Wu, Y.-B., Chen, F., Jahn, B.-m., 2005. Zircon U–Pb age, element and C–O isotope geochemistry of post-collisional mafic-ultramafic rocks from the Dabie orogen in east-central China. Lithos 83, 1-28.

Zhao, Z.-F., Zheng, Y.-F., Zhang, J., Dai, L.-Q., Li, Q., Liu, X., 2012. Syn-exhumation magmatism during continental collision: Evidence from alkaline intrusives of Triassic age in the Sulu orogen. Chemical Geology 328, 70-88.

Zheng, Y.-F., Hermann, J., 2014. Geochemistry of continental subduction-zone fluids. Earth, Planets and Space 66, 93; doi:10.1186/1880-5981-66-93.

Zou, H.-B., Zindler, A., Xu, X.-S., Qi, Q., 2000. Major, trace element, and Nd, Sr and Pb isotope studies of Cenozoic basalts in SE China: mantle sources, regional variations, and tectonic significance. Chemical Geology 171, 33-47.

1. ^*^ Corresponding author. E-mail address: zxu85@ustc.edu.cn [↑](#footnote-ref-2)
