# Supplementary material for: Zircon evidence for incorporation of terrigenous sediments into the magma source of continental basalts
Source: Sci Rep. 2018 Jan 9;8:178. doi: 10.1038/s41598-017-18549-7 (PMC5760614; doi:10.1038/s41598-017-18549-7)
Supplement: Supplementary file 2 — Table S1 to S7 [file 41598_2017_18549_MOESM2_ESM.doc]

**Zircon evidence for incorporation of terrigenous sediments into the magma source of continental basalts**

Zheng Xu[[1]](#footnote-2)*, Yong-Fei Zheng, Zi-Fu Zhao

CAS Key Laboratory of Crust-Mantle Materials and Environments,

School of Earth and Space Sciences, University of Science and Technology of China,

Hefei 230026, China

**Supplementary data**

Table S1. Zircon SHRIMP U-Pb data for Cenozoic continental basalts from east-central China

| Spot | Th (ppm) | U (ppm) | Th/U | *Pb^a^ (ppm) | ^207^Pb/^206^Pb | 1σ | ^207^Pb/^235^U | 1σ | ^206^Pb/^238^U | 1σ | t_207/206_ (Ma) | 1σ | t_207/235_ (Ma) | 1σ | t_206/238_ (Ma) | 1σ | concord^b^ (%) |
| --- | --- | --- | --- | --- | --- | --- | --- | --- | --- | --- | --- | --- | --- | --- | --- | --- | --- |
| 05HF04 |  |  |  |  |  |  |  |  |  |  |  |  |  |  |  |  |  |
| 1.1 | 612 | 523 | 1.17 | 8.9 | 0.0484 | 0.0061 | 0.1270 | 0.0169 | 0.0190 | 0.0007 | 118 | 295 | 121 | 17 | 122 | 4 | 0.8 |
| 2.1 | 241 | 440 | 0.55 | 22.4 | 0.0526 | 0.0041 | 0.4260 | 0.0354 | 0.0587 | 0.0018 | 313 | 175 | 360 | 35 | 367 | 11 | 1.9 |
| 3.1 | 293 | 234 | 1.25 | 4.5 | 0.0487 | 0.0048 | 0.1380 | 0.0139 | 0.0205 | 0.0010 | 135 | 230 | 131 | 14 | 131 | 6 | 0.0 |
| 4.1 | 622 | 945 | 0.66 | 42.2 | 0.0570 | 0.0067 | 0.3600 | 0.0461 | 0.0458 | 0.0016 | 492 | 258 | 312 | 46 | 289 | 10 | -7.4 |
| 5.1 | 291 | 729 | 0.40 | 54.3 | 0.0643 | 0.0016 | 0.7569 | 0.0273 | 0.0854 | 0.0022 | 751 | 53 | 572 | 27 | 528 | 13 | -7.7 |
|  |  |  |  |  |  |  |  |  |  |  |  |  |  |  |  |  |  |
| 05HF15 |  |  |  |  |  |  |  |  |  |  |  |  |  |  |  |  |  |
| 1.1 | 113 | 498 | 0.23 | 191.0 | 0.1741 | 0.0024 | 10.7300 | 0.3112 | 0.4470 | 0.0116 | 2597 | 23 | 2500 | 275 | 2380 | 51 | 3.9 |
| 2.1 | 363 | 327 | 1.11 | 10.4 | 0.0527 | 0.0037 | 0.2630 | 0.0208 | 0.0362 | 0.0011 | 316 | 161 | 237 | 21 | 229 | 7 | -3.4 |
| 3.1 | 565 | 315 | 1.79 | 5.4 | 0.0488 | 0.0025 | 0.1255 | 0.0073 | 0.0187 | 0.0008 | 138 | 120 | 120 | 7 | 119 | 5 | -0.8 |
| 4.1 | 131 | 1044 | 0.13 | 68.3 | 0.0902 | 0.0037 | 0.9380 | 0.0544 | 0.0754 | 0.0031 | 1430 | 78 | 672 | 54 | 469 | 18 | -30.2 |
| 4.2 | 47 | 1000 | 0.05 | 232.0 | 0.1180 | 0.0009 | 4.3800 | 0.1183 | 0.2693 | 0.0067 | 1926 | 14 | 1709 | 113 | 1537 | 35 | 12.7 |
| 5.1 | 144 | 436 | 0.33 | 194.0 | 0.1897 | 0.0021 | 13.5500 | 0.3794 | 0.5180 | 0.0135 | 2740 | 18 | 2719 | 327 | 2692 | 57 | 0.8 |
| 5.2 | 81 | 368 | 0.22 | 135.0 | 0.1792 | 0.0023 | 10.5500 | 0.3060 | 0.4270 | 0.0111 | 2645 | 22 | 2484 | 271 | 2293 | 50 | 6.5 |
| 6.1 | 447 | 180 | 2.49 | 3.1 | 0.0507 | 0.0031 | 0.1310 | 0.0088 | 0.0187 | 0.0010 | 227 | 141 | 125 | 9 | 120 | 6 | -4.0 |
| 7.1 | 300 | 152 | 1.97 | 2.6 | 0.0496 | 0.0042 | 0.1260 | 0.0108 | 0.0184 | 0.0007 | 174 | 197 | 120 | 11 | 118 | 5 | -1.7 |
| 8.1 | 126 | 482 | 0.26 | 140.0 | 0.1496 | 0.0012 | 6.9700 | 0.1882 | 0.3380 | 0.0088 | 2341 | 14 | 2108 | 175 | 1877 | 42 | 11.1 |
| 9.1 | 136 | 207 | 0.66 | 60.9 | 0.1440 | 0.0017 | 6.7900 | 0.2037 | 0.3420 | 0.0096 | 2276 | 21 | 2084 | 188 | 1896 | 45 | 9.2 |
| 10.1 | 186 | 365 | 0.51 | 149.0 | 0.1977 | 0.0013 | 12.9500 | 0.3497 | 0.4750 | 0.0124 | 2808 | 11 | 2676 | 304 | 2506 | 54 | 4.9 |
|  |  |  |  |  |  |  |  |  |  |  |  |  |  |  |  |  |  |
| 05HF18 |  |  |  |  |  |  |  |  |  |  |  |  |  |  |  |  |  |
| 1.1 | 624 | 342 | 1.82 | 6.0 | 0.0574 | 0.0031 | 0.1620 | 0.0102 | 0.0204 | 0.0007 | 507 | 120 | 152 | 10 | 130 | 4 | -14.5 |
| 2.1 | 137 | 155 | 0.88 | 7.0 | 0.0649 | 0.0032 | 0.4710 | 0.0269 | 0.0526 | 0.0016 | 771 | 100 | 392 | 27 | 331 | 10 | -15.6 |
| 3.1 | 56 | 294 | 0.19 | 89.0 | 0.1183 | 0.0013 | 5.7500 | 0.1610 | 0.3525 | 0.0092 | 1931 | 19 | 1939 | 152 | 1947 | 44 | -0.4 |
| 4.1 | 2308 | 674 | 3.42 | 10.6 | 0.0559 | 0.0024 | 0.1418 | 0.0072 | 0.0184 | 0.0005 | 450 | 95 | 135 | 7 | 118 | 3 | -12.6 |
| 5.1 | 34 | 279 | 0.12 | 7.6 | 0.0502 | 0.0040 | 0.2170 | 0.0185 | 0.0314 | 0.0010 | 202 | 180 | 199 | 19 | 200 | 6 | 0.5 |
|  |  |  |  |  |  |  |  |  |  |  |  |  |  |  |  |  |  |

| Table S1 (continued) | | | | | | | | | | | | | | | | | |
| --- | --- | --- | --- | --- | --- | --- | --- | --- | --- | --- | --- | --- | --- | --- | --- | --- | --- |
| Spot | Th (ppm) | U (ppm) | Th/U | *Pb^a^ (ppm) | ^207^Pb/^206^Pb | 1σ | ^207^Pb/^235^U | 1σ | ^206^Pb/^238^U | 1σ | t_207/206_ (Ma) | 1σ | t_207/235_ (Ma) | 1σ | t_206/238_ (Ma) | 1σ | concord^b^ (%) |
| 6.1 | 78 | 228 | 0.34 | 12.9 | 0.0743 | 0.0026 | 0.6770 | 0.0305 | 0.0660 | 0.0018 | 1050 | 71 | 525 | 30 | 412 | 11 | -21.5 |
| 7.1 | 257 | 193 | 1.33 | 21.3 | 0.0635 | 0.0020 | 1.1220 | 0.0460 | 0.1281 | 0.0035 | 726 | 66 | 764 | 46 | 777 | 20 | 1.7 |
| 8.1 | 205 | 884 | 0.23 | 235.0 | 0.1131 | 0.0007 | 4.8300 | 0.1256 | 0.3099 | 0.0078 | 1850 | 11 | 1790 | 120 | 1740 | 38 | 3.4 |
| 9.1 | 117 | 137 | 0.85 | 2.5 | 0.0640 | 0.0173 | 0.1810 | 0.0489 | 0.0206 | 0.0008 | 729 | 580 | 169 | 48 | 132 | 5 | -21.9 |
| 10.1 | 244 | 295 | 0.83 | 82.2 | 0.1127 | 0.0012 | 5.0500 | 0.1465 | 0.3248 | 0.0084 | 1844 | 20 | 1828 | 139 | 1813 | 42 | 0.9 |
| 11.1 | 290 | 173 | 1.68 | 3.5 | 0.0600 | 0.0120 | 0.1900 | 0.0399 | 0.0230 | 0.0008 | 602 | 440 | 177 | 40 | 146 | 5 | -17.5 |
|  |  |  |  |  |  |  |  |  |  |  |  |  |  |  |  |  |  |
| 05NS04 |  |  |  |  |  |  |  |  |  |  |  |  |  |  |  |  |  |
| 1.1 | 267 | 731 | 0.37 | 149.0 | 0.1146 | 0.0010 | 3.7500 | 0.1013 | 0.2373 | 0.0062 | 1874 | 16 | 1582 | 98 | 1373 | 32 | 18.5 |
| 2.1 | 237 | 192 | 1.24 | 3.4 | 0.0493 | 0.0045 | 0.1300 | 0.0130 | 0.0191 | 0.0009 | 161 | 213 | 124 | 13 | 122 | 6 | -1.6 |
| 5.1 | 1006 | 664 | 1.52 | 169.0 | 0.1252 | 0.0009 | 5.1200 | 0.1382 | 0.2966 | 0.0077 | 2032 | 13 | 1839 | 131 | 1675 | 38 | 10.5 |
| 6.1 | 134 | 740 | 0.18 | 84.9 | 0.0688 | 0.0007 | 1.2636 | 0.0354 | 0.1333 | 0.0035 | 891 | 21 | 830 | 35 | 807 | 20 | -2.8 |
| 6.2 | 27 | 123 | 0.22 | 13.3 | 0.0703 | 0.0023 | 1.2130 | 0.0582 | 0.1252 | 0.0045 | 936 | 66 | 807 | 57 | 761 | 26 | -5.7 |
| 7.1 | 1327 | 875 | 1.52 | 84.7 | 0.0649 | 0.0009 | 1.0070 | 0.0292 | 0.1125 | 0.0028 | 772 | 29 | 707 | 29 | 688 | 16 | -2.7 |
| 8.1 | 200 | 159 | 1.26 | 2.7 | 0.0848 | 0.0144 | 0.2280 | 0.0388 | 0.0195 | 0.0006 | 1311 | 330 | 209 | 39 | 125 | 4 | -40.2 |
| 9.1 | 111 | 182 | 0.61 | 57.3 | 0.1263 | 0.0015 | 6.3680 | 0.1910 | 0.3658 | 0.0099 | 2047 | 21 | 2028 | 178 | 2010 | 47 | 0.9 |
| 10.1 | 558 | 271 | 2.06 | 4.5 | 0.0483 | 0.0092 | 0.1280 | 0.0243 | 0.0192 | 0.0006 | 114 | 448 | 122 | 24 | 123 | 4 | 0.8 |
| 11.1 | 301 | 191 | 1.58 | 3.2 | 0.0575 | 0.0054 | 0.1530 | 0.0153 | 0.0193 | 0.0006 | 509 | 207 | 145 | 15 | 123 | 4 | -15.2 |
|  |  |  |  |  |  |  |  |  |  |  |  |  |  |  |  |  |  |
| 05WD01 |  |  |  |  |  |  |  |  |  |  |  |  |  |  |  |  |  |
| 1.1 | 85 | 200 | 0.42 | 85.8 | 0.1818 | 0.0010 | 12.5100 | 0.1126 | 0.4991 | 0.0036 | 2669 | 9 | 2643 | 108 | 2610 | 16 | 1.0 |
| 2.1 | 47 | 83 | 0.57 | 38.6 | 0.1890 | 0.0018 | 14.1300 | 0.1978 | 0.5423 | 0.0060 | 2733 | 15 | 2758 | 183 | 2793 | 24 | -0.9 |
| 3.1 | 57 | 98 | 0.59 | 43.9 | 0.1781 | 0.0016 | 12.7400 | 0.1656 | 0.5187 | 0.0047 | 2636 | 15 | 2661 | 156 | 2694 | 20 | -0.9 |
| 4.1 | 65 | 147 | 0.44 | 62.3 | 0.1650 | 0.0012 | 11.2000 | 0.1232 | 0.4923 | 0.0040 | 2508 | 12 | 2540 | 118 | 2581 | 17 | -1.3 |
| 4.2 | 155 | 213 | 0.73 | 93.6 | 0.1674 | 0.0009 | 11.8000 | 0.1062 | 0.5114 | 0.0037 | 2531 | 9 | 2589 | 102 | 2663 | 16 | -2.2 |
| 5.1 | 31 | 117 | 0.27 | 6.9 | 0.0572 | 0.0018 | 0.5410 | 0.0179 | 0.0686 | 0.0008 | 498 | 68 | 439 | 18 | 428 | 5 | -2.5 |
| 6.1 | 32 | 95 | 0.33 | 5.5 | 0.0561 | 0.0020 | 0.5230 | 0.0194 | 0.0676 | 0.0010 | 456 | 78 | 427 | 19 | 422 | 6 | -1.2 |
| 7.1 | 21 | 503 | 0.04 | 41.7 | 0.0665 | 0.0011 | 0.8764 | 0.0158 | 0.0956 | 0.0007 | 821 | 35 | 639 | 16 | 589 | 4 | -7.8 |
|  | | | | | | | | | | | | | | | | | |

| Table S1 (continued) | | | | | | | | | | | | | | | | | |
| --- | --- | --- | --- | --- | --- | --- | --- | --- | --- | --- | --- | --- | --- | --- | --- | --- | --- |
| Spot | Th (ppm) | U (ppm) | Th/U | *Pb^a^ (ppm) | ^207^Pb/^206^Pb | 1σ | ^207^Pb/^235^U | 1σ | ^206^Pb/^238^U | 1σ | t_207/206_ (Ma) | 1σ | t_207/235_ (Ma) | 1σ | t_206/238_ (Ma) | 1σ | concord^b^ (%) |
| 8.1 | 403 | 316 | 1.27 | 26.2 | 0.0656 | 0.0016 | 0.8684 | 0.0217 | 0.0960 | 0.0008 | 795 | 50 | 635 | 22 | 591 | 5 | -6.9 |
| 9.1 | 175 | 175 | 1.00 | 79.2 | 0.1741 | 0.0012 | 12.6000 | 0.1386 | 0.5249 | 0.0046 | 2597 | 11 | 2650 | 132 | 2720 | 19 | -2.0 |
|  |  |  |  |  |  |  |  |  |  |  |  |  |  |  |  |  |  |
| 06SW05 |  |  |  |  |  |  |  |  |  |  |  |  |  |  |  |  |  |
| 1.1 | 234 | 206 | 1.14 | 84.1 | 0.1608 | 0.0013 | 10.5500 | 0.1055 | 0.4758 | 0.0031 | 2464 | 13 | 2484 | 102 | 2509 | 14 | -0.8 |
| 2.1 | 912 | 1670 | 0.55 | 596.0 | 0.1533 | 0.0004 | 8.7834 | 0.0492 | 0.4156 | 0.0020 | 2383 | 5 | 2316 | 49 | 2240 | 9 | 2.9 |
| 3.1 | 189 | 574 | 0.33 | 241.0 | 0.1602 | 0.0007 | 10.7850 | 0.0766 | 0.4882 | 0.0027 | 2458 | 8 | 2505 | 75 | 2563 | 12 | -1.9 |
| 4.1 | 832 | 1223 | 0.68 | 378.0 | 0.1274 | 0.0004 | 6.3230 | 0.0379 | 0.3599 | 0.0018 | 2063 | 5 | 2022 | 38 | 1982 | 9 | 2.0 |
| 5.1 | 1536 | 1867 | 0.82 | 694.0 | 0.1469 | 0.0016 | 8.7657 | 0.1140 | 0.4327 | 0.0024 | 2310 | 19 | 2314 | 110 | 2318 | 11 | -0.2 |
| 6.1 | 82 | 1835 | 0.04 | 29.5 | 0.0492 | 0.0008 | 0.1266 | 0.0022 | 0.0187 | 0.0001 | 156 | 37 | 121 | 2 | 119 | 1 | -1.7 |
| 7.1 | 16 | 23 | 0.71 | 8.6 | 0.1433 | 0.0026 | 8.5800 | 0.2488 | 0.4342 | 0.0100 | 2268 | 31 | 2294 | 226 | 2325 | 44 | -1.1 |
| 8.1 | 1302 | 1745 | 0.75 | 53.6 | 0.0827 | 0.0031 | 0.3930 | 0.0145 | 0.0345 | 0.0002 | 1262 | 72 | 337 | 15 | 218 | 2 | -35.3 |
| 9.1 | 1741 | 3315 | 0.52 | 64.0 | 0.0534 | 0.0011 | 0.1647 | 0.0035 | 0.0224 | 0.0002 | 345 | 45 | 155 | 4 | 143 | 1 | -7.7 |
| 10.1 | 245 | 1348 | 0.18 | 446.0 | 0.1495 | 0.0004 | 7.9300 | 0.0452 | 0.3848 | 0.0020 | 2340 | 4 | 2223 | 45 | 2099 | 9 | 5.3 |
|  |  |  |  |  |  |  |  |  |  |  |  |  |  |  |  |  |  |
| 06SW07 |  |  |  |  |  |  |  |  |  |  |  |  |  |  |  |  |  |
| 2.1 | 205 | 197 | 1.04 | 3.6 | 0.0513 | 0.0051 | 0.1410 | 0.0141 | 0.0200 | 0.0003 | 254 | 230 | 134 | 14 | 127 | 2 | -5.2 |
| 3.1 | 175 | 218 | 0.80 | 3.4 | 0.0675 | 0.0064 | 0.2080 | 0.0200 | 0.0224 | 0.0003 | 852 | 200 | 192 | 20 | 143 | 2 | -25.5 |
| 4.1 | 702 | 36 | 19.77 | 146.0 | 0.1022 | 0.0010 | 3.4200 | 0.0376 | 0.2427 | 0.0014 | 1665 | 18 | 1509 | 37 | 1401 | 7 | 10.3 |
| 6.1 | 134 | 109 | 1.23 | 2.6 | 0.0575 | 0.0063 | 0.1760 | 0.0194 | 0.0223 | 0.0003 | 509 | 240 | 165 | 19 | 142 | 2 | -13.9 |
| 7.1 | 165 | 161 | 1.02 | 3.1 | 0.0602 | 0.0058 | 0.1760 | 0.0173 | 0.0212 | 0.0003 | 612 | 210 | 165 | 17 | 135 | 2 | -18.2 |
| 8.1 | 155 | 151 | 1.03 | 2.7 | 0.0555 | 0.0034 | 0.1530 | 0.0095 | 0.0200 | 0.0003 | 431 | 140 | 145 | 10 | 128 | 2 | -11.7 |
| 9.1 | 350 | 900 | 0.39 | 59.3 | 0.0775 | 0.0006 | 2.1090 | 0.0209 | 0.1973 | 0.0013 | 1135 | 15 | 1152 | 21 | 1161 | 7 | -1.5 |
| 10.1 | 245 | 220 | 1.15 | 4.0 | 0.0475 | 9.1000 | 0.1380 | 9.2000 | 0.0211 | 1.1000 | 75 | 220 | 131 | 13 | 135 | 2 | 2.6 |
|  |  |  |  |  |  |  |  |  |  |  |  |  |  |  |  |  |  |
| 06SW21a |  |  |  |  |  |  |  |  |  |  |  |  |  |  |  |  |  |
| 1.1 | 104 | 261 | 0.40 | 71.6 | 0.1221 | 0.0008 | 5.3790 | 0.0495 | 0.3194 | 0.0022 | 1988 | 11 | 1882 | 49 | 1787 | 11 | 5.6 |
| 2.1 | 64 | 128 | 0.50 | 7.9 | 0.0551 | 0.0022 | 0.5380 | 0.0215 | 0.0709 | 0.0008 | 415 | 87 | 437 | 22 | 441 | 5 | 0.9 |
|  |  |  |  |  |  |  |  |  |  |  |  |  |  |  |  |  |  |

| Table S1 (continued) | | | | | | | | | | | | | | | | | |
| --- | --- | --- | --- | --- | --- | --- | --- | --- | --- | --- | --- | --- | --- | --- | --- | --- | --- |
| Spot | Th (ppm) | U (ppm) | Th/U | *Pb^a^ (ppm) | ^207^Pb/^206^Pb | 1σ | ^207^Pb/^235^U | 1σ | ^206^Pb/^238^U | 1σ | t_207/206_ (Ma) | 1σ | t_207/235_ (Ma) | 1σ | t_206/238_ (Ma) | 1σ | concord^b^ (%) |
| 3.1 | 148 | 337 | 0.44 | 82.6 | 0.1236 | 0.0010 | 4.8586 | 0.0486 | 0.2850 | 0.0018 | 2009 | 14 | 1795 | 48 | 1616 | 9 | 11.9 |
| 4.1 | 102 | 174 | 0.59 | 11.2 | 0.0571 | 0.0023 | 0.5840 | 0.0245 | 0.0742 | 0.0007 | 496 | 90 | 467 | 25 | 461 | 4 | -1.3 |
| 5.1 | 105 | 224 | 0.47 | 7.7 | 0.0523 | 0.0019 | 0.2850 | 0.0106 | 0.0395 | 0.0004 | 300 | 82 | 255 | 11 | 250 | 2 | -2.0 |
| 6.1 | 92 | 239 | 0.39 | 57.1 | 0.1237 | 0.0009 | 4.7400 | 0.0474 | 0.2779 | 0.0020 | 2010 | 13 | 1774 | 47 | 1581 | 10 | 13.3 |
| 6.2 | 482 | 1654 | 0.29 | 327.0 | 0.1296 | 0.0005 | 4.1080 | 0.0255 | 0.2299 | 0.0012 | 2092 | 6 | 1656 | 26 | 1334 | 6 | 26.3 |
| 7.1 | 157 | 289 | 0.54 | 18.0 | 0.1103 | 0.0020 | 1.0990 | 0.0220 | 0.0723 | 0.0006 | 1804 | 33 | 753 | 22 | 450 | 4 | -40.2 |
| 8.1 | 372 | 304 | 1.22 | 5.4 | 0.0489 | 0.0024 | 0.1371 | 0.0067 | 0.0203 | 0.0002 | 142 | 113 | 130 | 7 | 130 | 1 | 0.0 |
| 9.1 | 168 | 246 | 0.68 | 15.7 | 0.0590 | 0.0020 | 0.6020 | 0.0205 | 0.0740 | 0.0006 | 567 | 72 | 479 | 21 | 460 | 4 | -4.0 |
| 10.1 | 121 | 203 | 0.60 | 12.9 | 0.0557 | 0.0025 | 0.5620 | 0.0259 | 0.0732 | 0.0006 | 439 | 100 | 453 | 26 | 456 | 4 | 0.7 |
|  |  |  |  |  |  |  |  |  |  |  |  |  |  |  |  |  |  |
| 06SW21b |  |  |  |  |  |  |  |  |  |  |  |  |  |  |  |  |  |
| 2.1 | 779 | 2123 | 0.37 | 912.0 | 0.1663 | 0.0003 | 11.4570 | 0.0630 | 0.4998 | 0.0026 | 2520 | 3 | 2561 | 62 | 2613 | 11 | -1.6 |
| 3.1 | 104 | 297 | 0.35 | 87.9 | 0.1164 | 0.0006 | 5.5160 | 0.0469 | 0.3438 | 0.0023 | 1901 | 10 | 1903 | 47 | 1905 | 11 | -0.1 |
| 4.1 | 89 | 244 | 0.37 | 68.6 | 0.1140 | 0.0009 | 5.1330 | 0.0513 | 0.3265 | 0.0023 | 1864 | 14 | 1842 | 51 | 1821 | 11 | 1.2 |
| 4.2 | 34 | 752 | 0.05 | 205.0 | 0.1135 | 0.0004 | 4.9760 | 0.0323 | 0.3178 | 0.0018 | 1857 | 6 | 1815 | 32 | 1779 | 9 | 2.3 |
| 5.1 | 21 | 1117 | 0.02 | 334.0 | 0.1126 | 0.0003 | 5.4050 | 0.0346 | 0.3483 | 0.0020 | 1841 | 6 | 1886 | 35 | 1926 | 10 | -2.4 |
| 6.1 | 603 | 1679 | 0.36 | 101.0 | 0.0553 | 0.0006 | 0.5330 | 0.0064 | 0.0699 | 0.0004 | 423 | 24 | 434 | 6 | 436 | 2 | 0.5 |
| 7.1 | 55 | 457 | 0.12 | 137.0 | 0.1146 | 0.0005 | 5.5050 | 0.0413 | 0.3484 | 0.0021 | 1873 | 8 | 1901 | 41 | 1927 | 10 | -1.5 |
| 8.1 | 97 | 371 | 0.26 | 118.0 | 0.1166 | 0.0026 | 5.8000 | 0.1334 | 0.3611 | 0.0025 | 1904 | 40 | 1946 | 127 | 1988 | 12 | -2.2 |
| 9.1 | 106 | 997 | 0.11 | 293.0 | 0.1148 | 0.0004 | 5.4190 | 0.0352 | 0.3422 | 0.0019 | 1877 | 6 | 1888 | 35 | 1897 | 9 | -0.6 |
|  |  |  |  |  |  |  |  |  |  |  |  |  |  |  |  |  |  |
| 06SW25 |  |  |  |  |  |  |  |  |  |  |  |  |  |  |  |  |  |
| 1.1 | 150 | 145 | 1.04 | 15.6 | 0.0648 | 0.0023 | 1.0710 | 0.0396 | 0.1199 | 0.0010 | 767 | 76 | 739 | 39 | 730 | 6 | -1.2 |
| 2.1 | 128 | 20 | 6.23 | 7.4 | 0.0575 | 0.0039 | 0.5230 | 0.0350 | 0.0660 | 0.0007 | 509 | 147 | 427 | 35 | 412 | 4 | -3.5 |
| 3.1 | 141 | 78 | 1.80 | 36.4 | 0.1094 | 0.0009 | 4.5150 | 0.0542 | 0.2992 | 0.0024 | 1790 | 16 | 1734 | 54 | 1687 | 12 | 3.2 |
| 4.1 | 221 | 196 | 1.13 | 4.2 | 0.0499 | 0.0036 | 0.1432 | 0.0103 | 0.0208 | 0.0003 | 190 | 168 | 136 | 10 | 133 | 2 | -2.2 |
| 5.1 | 235 | 302 | 0.78 | 24.6 | 0.0654 | 0.0018 | 1.0950 | 0.0307 | 0.1215 | 0.0009 | 787 | 57 | 751 | 31 | 739 | 5 | -1.6 |
| 6.1 | 162 | 172 | 0.94 | 17.3 | 0.0671 | 0.0017 | 1.1400 | 0.0296 | 0.1233 | 0.0010 | 840 | 52 | 773 | 30 | 749 | 6 | -3.1 |
|  | | | | | | | | | | | | | | | | | |

| Table S1 (continued) | | | | | | | | | | | | | | | | | |
| --- | --- | --- | --- | --- | --- | --- | --- | --- | --- | --- | --- | --- | --- | --- | --- | --- | --- |
| Spot | Th (ppm) | U (ppm) | Th/U | *Pb^a^ (ppm) | ^207^Pb/^206^Pb | 1σ | ^207^Pb/^235^U | 1σ | ^206^Pb/^238^U | 1σ | t_207/206_ (Ma) | 1σ | t_207/235_ (Ma) | 1σ | t_206/238_ (Ma) | 1σ | concord^b^ (%) |
| 7.1 | 101 | 88 | 1.15 | 1.8 | 0.0651 | 0.0028 | 0.1790 | 0.0077 | 0.0200 | 0.0005 | 777 | 91 | 167 | 8 | 127 | 3 | -24.0 |
| 8.1 | 270 | 322 | 0.84 | 18.7 | 0.0622 | 0.0022 | 0.6850 | 0.0247 | 0.0798 | 0.0006 | 682 | 75 | 530 | 25 | 495 | 4 | -6.6 |
| 9.1 | 197 | 307 | 0.64 | 64.6 | 0.1264 | 0.0008 | 6.6220 | 0.0636 | 0.3800 | 0.0027 | 2048 | 11 | 2062 | 63 | 2076 | 13 | -0.7 |
| 10.1 | 185 | 204 | 0.91 | 18.9 | 0.0653 | 0.0015 | 1.0630 | 0.0255 | 0.1181 | 0.0009 | 784 | 48 | 735 | 26 | 720 | 5 | -2.0 |
|  |  |  |  |  |  |  |  |  |  |  |  |  |  |  |  |  |  |
| 06SW28 |  |  |  |  |  |  |  |  |  |  |  |  |  |  |  |  |  |
| 1.1 | 155 | 467 | 0.33 | 158.0 | 0.1425 | 0.0005 | 7.7260 | 0.0525 | 0.3932 | 0.0023 | 2258 | 6 | 2200 | 52 | 2138 | 10 | 2.6 |
| 2.1 | 59 | 282 | 0.21 | 81.9 | 0.1125 | 0.0017 | 5.1250 | 0.0820 | 0.3305 | 0.0024 | 1840 | 26 | 1840 | 80 | 1841 | 12 | 0.0 |
| 3.1 | 304 | 901 | 0.34 | 243.0 | 0.1068 | 0.0004 | 4.6160 | 0.0323 | 0.3135 | 0.0018 | 1745 | 8 | 1752 | 32 | 1758 | 9 | -0.4 |
| 4.1 | 154 | 544 | 0.28 | 169.0 | 0.1318 | 0.0009 | 6.5540 | 0.0610 | 0.3608 | 0.0022 | 2122 | 12 | 2053 | 60 | 1986 | 11 | 3.4 |
| 06SW48 |  |  |  |  |  |  |  |  |  |  |  |  |  |  |  |  |  |
| 1.1 | 361 | 408 | 0.88 | 17.0 | 0.0539 | 0.0015 | 0.3580 | 0.0104 | 0.0481 | 0.0004 | 367 | 62 | 311 | 10 | 303 | 2 | -2.6 |
| 2.1 | 130 | 122 | 1.06 | 42.5 | 0.1312 | 0.0024 | 7.3200 | 0.1464 | 0.4043 | 0.0035 | 2115 | 31 | 2151 | 139 | 2189 | 16 | -1.7 |
| 3.1 | 42 | 79 | 0.54 | 27.0 | 0.1339 | 0.0032 | 7.3700 | 0.1916 | 0.3989 | 0.0040 | 2173 | 41 | 2157 | 178 | 2164 | 19 | 0.7 |
| 4.1 | 91 | 192 | 0.47 | 64.4 | 0.1357 | 0.0008 | 7.3180 | 0.0710 | 0.3911 | 0.0030 | 1914 | 11 | 2151 | 70 | 2128 | 14 | -11.0 |
| 5.1 | 83 | 115 | 0.72 | 40.7 | 0.1275 | 0.0017 | 7.2300 | 0.1157 | 0.4113 | 0.0035 | 2064 | 23 | 2140 | 111 | 2221 | 16 | -3.6 |
| 6.1 | 62 | 107 | 0.57 | 37.2 | 0.1334 | 0.0011 | 7.4080 | 0.0889 | 0.4028 | 0.0036 | 2143 | 15 | 2162 | 86 | 2182 | 17 | -0.9 |
| 7.1 | 34 | 71 | 0.48 | 24.4 | 0.1292 | 0.0019 | 7.1500 | 0.1359 | 0.4014 | 0.0044 | 2087 | 27 | 2130 | 129 | 2176 | 20 | -2.0 |
| 8.1 | 161 | 282 | 0.57 | 98.2 | 0.1372 | 0.0017 | 7.6500 | 0.1071 | 0.4042 | 0.0028 | 2192 | 20 | 2191 | 103 | 2188 | 13 | 0.0 |
| 9.1 | 51 | 111 | 0.46 | 37.7 | 0.1306 | 0.0016 | 7.1200 | 0.1068 | 0.3954 | 0.0037 | 2105 | 21 | 2127 | 103 | 2148 | 17 | -1.0 |
| 10.1 | 66 | 121 | 0.55 | 40.6 | 0.1290 | 0.0044 | 6.9600 | 0.2436 | 0.3911 | 0.0035 | 2085 | 60 | 2106 | 221 | 2128 | 16 | -1.0 |

^a^ *Pb means radiogenic Pb.

^b^ concord denotes the concordance between three U-Pb apparent ages, which are calculated following 100×(t_206/238_/t_207/235_-1) and 100×(t_207/206_/t_207/235_-1) for zircons younger and older than 1000 Ma, respectively.

Table S2. Zircon LA-ICPMS U-Pb data for Cenozoic continental basalts from east-central China

| Spot | U (ppm) | Th (ppm) | Th/U | *Pb^a^ (ppm) | ^207^Pb/^206^Pb | 1σ | ^207^Pb/^235^U | 1σ | ^206^Pb/^238^U | 1σ | t_207/206_ (Ma) | 1σ | t_207/235_ (Ma) | 1σ | t_206/238_ (Ma) | 1σ | concord^b^ (%) |
| --- | --- | --- | --- | --- | --- | --- | --- | --- | --- | --- | --- | --- | --- | --- | --- | --- | --- |
| 07CL03 |  |  |  |  |  |  |  |  |  |  |  |  |  |  |  |  |  |
| 1 | 37 | 34 | 0.91 | 6.4 | 0.0730 | 0.0047 | 1.2963 | 0.0804 | 0.1289 | 0.0029 | 1013 | 131 | 844 | 36 | 781 | 17 | -7.4 |
| 2 | 50 | 29 | 0.57 | 8.3 | 0.0722 | 0.0032 | 1.3065 | 0.0547 | 0.1312 | 0.0025 | 992 | 90 | 849 | 24 | 795 | 14 | -6.3 |
| 3 | 39 | 31 | 0.79 | 6.7 | 0.0776 | 0.0055 | 1.3809 | 0.0935 | 0.1291 | 0.0032 | 1136 | 140 | 881 | 40 | 783 | 18 | -11.1 |
| 4 | 24 | 11 | 0.47 | 4.1 | 0.0961 | 0.0065 | 1.7207 | 0.1102 | 0.1299 | 0.0033 | 1550 | 126 | 1016 | 41 | 787 | 19 | -22.6 |
| 5 | 31 | 16 | 0.53 | 5.2 | 0.0855 | 0.0056 | 1.5323 | 0.0958 | 0.1301 | 0.0032 | 1326 | 126 | 943 | 38 | 788 | 18 | -16.5 |
| 6 | 43 | 23 | 0.54 | 7.2 | 0.0724 | 0.0048 | 1.3241 | 0.0844 | 0.1327 | 0.0031 | 997 | 135 | 856 | 37 | 803 | 18 | -6.2 |
| 7 | 50 | 31 | 0.63 | 8.3 | 0.0780 | 0.0037 | 1.3960 | 0.0618 | 0.1298 | 0.0025 | 1146 | 93 | 887 | 26 | 787 | 14 | -11.3 |
| 8 | 50 | 32 | 0.64 | 8.6 | 0.0824 | 0.0043 | 1.4923 | 0.0743 | 0.1314 | 0.0028 | 1254 | 103 | 927 | 30 | 796 | 16 | -14.2 |
| 9 | 1 | 35 | 28.17 | 8.5 | 0.0724 | 0.0029 | -6.1174 | 0.3436 | -0.6127 | 0.0277 | 997 | 82 |  |  |  |  |  |
| 10 | 36 | 20 | 0.56 | 5.8 | 0.0782 | 0.0044 | 1.3894 | 0.0737 | 0.1288 | 0.0028 | 1153 | 111 | 884 | 31 | 781 | 16 | -11.7 |
| 11 | 44 | 24 | 0.55 | 7.0 | 0.0810 | 0.0041 | 1.4035 | 0.0667 | 0.1257 | 0.0026 | 1220 | 99 | 890 | 28 | 764 | 15 | -14.3 |
| 12 | 60 | 47 | 0.78 | 10.2 | 0.0903 | 0.0046 | 1.5161 | 0.0728 | 0.1218 | 0.0026 | 1431 | 97 | 937 | 29 | 741 | 15 | -20.9 |
| 13 | 36 | 36 | 1.00 | 6.9 | 0.0941 | 0.0065 | 1.7358 | 0.1137 | 0.1338 | 0.0035 | 1511 | 129 | 1022 | 42 | 809 | 20 | -20.8 |
| 14 | 63 | 47 | 0.75 | 10.5 | 0.0818 | 0.0042 | 1.4119 | 0.0687 | 0.1252 | 0.0026 | 1241 | 101 | 894 | 29 | 760 | 15 | -14.9 |
| 15 | 50 | 37 | 0.75 | 8.1 | 0.0917 | 0.0071 | 1.5275 | 0.1136 | 0.1208 | 0.0034 | 1461 | 148 | 942 | 46 | 735 | 20 | -21.9 |
| 16 | 37 | 21 | 0.56 | 5.7 | 0.0858 | 0.0051 | 1.3941 | 0.0783 | 0.1178 | 0.0027 | 1334 | 114 | 886 | 33 | 718 | 15 | -19.0 |
| 17 | 35 | 30 | 0.86 | 6.0 | 0.0892 | 0.0049 | 1.5172 | 0.0787 | 0.1234 | 0.0027 | 1409 | 105 | 937 | 32 | 750 | 15 | -20.0 |
| 18 | 47 | 27 | 0.58 | 7.0 | 0.0845 | 0.0056 | 1.3648 | 0.0865 | 0.1171 | 0.0028 | 1305 | 129 | 874 | 37 | 714 | 16 | -18.3 |
| 19 | 47 | 31 | 0.67 | 7.5 | 0.0777 | 0.0045 | 1.3272 | 0.0734 | 0.1238 | 0.0027 | 1140 | 115 | 858 | 32 | 753 | 15 | -12.3 |
| 20 | 28 | 14 | 0.52 | 4.5 | 0.0838 | 0.0047 | 1.4619 | 0.0778 | 0.1265 | 0.0027 | 1289 | 109 | 915 | 32 | 768 | 16 | -16.1 |
|  |  |  |  |  |  |  |  |  |  |  |  |  |  |  |  |  |  |
| 07CL05 |  |  |  |  |  |  |  |  |  |  |  |  |  |  |  |  |  |
| 1 | 1079 | 1234 | 1.14 | 14.3 | 0.0581 | 0.0025 | 0.0726 | 0.0030 | 0.0091 | 0.0002 | 534 | 96 | 71 | 3 | 58 | 1 | -18.3 |
| 2 | 272 | 107 | 0.39 | 133.9 | 0.1422 | 0.0029 | 7.7004 | 0.1305 | 0.3927 | 0.0060 | 2254 | 36 | 2197 | 125 | 2135 | 39 | 2.6 |
| 3 | 464 | 24 | 0.05 | 149.8 | 0.1187 | 0.0024 | 4.6822 | 0.0754 | 0.2860 | 0.0043 | 1937 | 36 | 1764 | 74 | 1621 | 28 | 9.8 |
| 4 | 29 | 15 | 0.53 | 5.4 | 0.0862 | 0.0051 | 1.7827 | 0.1007 | 0.1500 | 0.0034 | 1343 | 114 | 1039 | 97 | 901 | 22 | -13.3 |

| Table S2 (continued) | | | | | | | | | | | | | | | | | |
| --- | --- | --- | --- | --- | --- | --- | --- | --- | --- | --- | --- | --- | --- | --- | --- | --- | --- |
| Spot | U (ppm) | Th (ppm) | Th/U | *Pb^a^ (ppm) | ^207^Pb/^206^Pb | 1σ | ^207^Pb/^235^U | 1σ | ^206^Pb/^238^U | 1σ | t_207/206_ (Ma) | 1σ | t_207/235_ (Ma) | 1σ | t_206/238_ (Ma) | 1σ | concord^b^ (%) |
| 5 | 333 | 234 | 0.70 | 198.3 | 0.1583 | 0.0031 | 9.7060 | 0.1489 | 0.4448 | 0.0066 | 2437 | 33 | 2407 | 141 | 2372 | 43 | 1.2 |
| 6 | 407 | 173 | 0.43 | 203.4 | 0.1575 | 0.0034 | 8.5024 | 0.1494 | 0.3914 | 0.0061 | 2429 | 36 | 2286 | 141 | 2130 | 39 | 6.3 |
| 7 | 534 | 355 | 0.67 | 276.5 | 0.1569 | 0.0032 | 8.4696 | 0.1412 | 0.3915 | 0.0060 | 2423 | 35 | 2283 | 134 | 2130 | 38 | 6.1 |
| 8 | 942 | 758 | 0.80 | 67.2 | 0.0823 | 0.0026 | 0.6026 | 0.0174 | 0.0531 | 0.0009 | 1252 | 63 | 479 | 18 | 334 | 6 | -30.3 |
| 9 | 264 | 63 | 0.24 | 109.4 | 0.1387 | 0.0035 | 6.5379 | 0.1413 | 0.3419 | 0.0056 | 2211 | 43 | 2051 | 134 | 1896 | 36 | 7.8 |
| 10 | 290 | 63 | 0.22 | 97.5 | 0.1280 | 0.0033 | 5.0317 | 0.1123 | 0.2851 | 0.0047 | 2071 | 45 | 1825 | 108 | 1617 | 30 | 13.5 |
| 11 | 462 | 217 | 0.47 | 208.8 | 0.1518 | 0.0038 | 7.3244 | 0.1574 | 0.3499 | 0.0058 | 2367 | 42 | 2152 | 148 | 1934 | 37 | 10.0 |
| 12 | 263 | 110 | 0.42 | 45.4 | 0.0846 | 0.0027 | 1.6476 | 0.0469 | 0.1412 | 0.0023 | 1307 | 61 | 989 | 47 | 851 | 15 | -14.0 |
| 13 | 514 | 318 | 0.62 | 249.3 | 0.1517 | 0.0032 | 7.7064 | 0.1342 | 0.3685 | 0.0057 | 2365 | 36 | 2197 | 128 | 2022 | 36 | 7.6 |
| 14 | 597 | 301 | 0.50 | 267.5 | 0.1486 | 0.0030 | 7.1229 | 0.1131 | 0.3477 | 0.0052 | 2330 | 34 | 2127 | 109 | 1923 | 33 | 9.5 |
|  |  |  |  |  |  |  |  |  |  |  |  |  |  |  |  |  |  |
| 07CL07 |  |  |  |  |  |  |  |  |  |  |  |  |  |  |  |  |  |
| 1 | 194 | 306 | 1.58 | 6.0 | 0.0688 | 0.0106 | 0.1877 | 0.0282 | 0.0198 | 0.0007 | 893 | 318 | 175 | 28 | 126 | 5 | -27.7 |
| 2 | 4492 | 1532 | 0.34 | 302.0 | 0.0572 | 0.0015 | 0.4741 | 0.0107 | 0.0601 | 0.0009 | 500 | 57 | 394 | 11 | 376 | 6 | -4.5 |
| 3 | 151 | 257 | 1.70 | 4.8 | 0.0763 | 0.0148 | 0.2114 | 0.0398 | 0.0201 | 0.0011 | 1102 | 389 | 195 | 40 | 128 | 7 | -34.1 |
| 4 | 202 | 93 | 0.46 | 76.1 | 0.1237 | 0.0048 | 5.1817 | 0.1869 | 0.3038 | 0.0063 | 2011 | 68 | 1850 | 174 | 1710 | 40 | 8.7 |
| 5 | 83 | 110 | 1.32 | 3.7 | 0.1896 | 0.0314 | 0.6032 | 0.0921 | 0.0231 | 0.0016 | 2739 | 273 | 479 | 89 | 147 | 10 | -69.3 |
| 6 | 365 | 602 | 1.65 | 18.9 | 0.0722 | 0.0083 | 0.3321 | 0.0366 | 0.0334 | 0.0011 | 993 | 232 | 291 | 36 | 211 | 7 | -27.4 |
| 7 | 1608 | 747 | 0.46 | 345.9 | 0.0949 | 0.0020 | 2.2985 | 0.0408 | 0.1757 | 0.0027 | 1526 | 40 | 1212 | 41 | 1044 | 17 | 25.9 |
| 8 | 384 | 57 | 0.15 | 108.0 | 0.1115 | 0.0030 | 3.8298 | 0.0910 | 0.2493 | 0.0041 | 1823 | 49 | 1599 | 88 | 1435 | 27 | 14.0 |
| 9 | 79 | 146 | 1.83 | 2.7 | 0.0652 | 0.0143 | 0.2046 | 0.0441 | 0.0228 | 0.0010 | 781 | 461 | 189 | 44 | 145 | 6 | -23.3 |
| 10 | 435 | 12 | 0.03 | 24.7 | 0.0706 | 0.0055 | 0.4900 | 0.0363 | 0.0503 | 0.0013 | 946 | 158 | 405 | 36 | 317 | 8 | -21.8 |
| 11 | 567 | 21 | 0.04 | 191.2 | 0.1264 | 0.0029 | 5.2552 | 0.1006 | 0.3015 | 0.0048 | 2049 | 40 | 1862 | 97 | 1699 | 31 | 10.1 |
| 12 | 201 | 416 | 2.08 | 13.9 | 0.1551 | 0.0116 | 0.9511 | 0.0660 | 0.0445 | 0.0014 | 2402 | 127 | 679 | 65 | 281 | 9 | -58.7 |
| 13 | 134 | 248 | 1.85 | 4.9 | 0.1310 | 0.0174 | 0.3951 | 0.0497 | 0.0219 | 0.0010 | 2111 | 233 | 338 | 49 | 140 | 6 | -58.7 |
| 14 | 90 | 119 | 1.32 | 2.9 | 0.0894 | 0.0137 | 0.2709 | 0.0404 | 0.0220 | 0.0008 | 1413 | 293 | 243 | 40 | 140 | 5 | -42.4 |
| 15 | 91 | 131 | 1.44 | 3.6 | 0.1255 | 0.0263 | 0.4210 | 0.0844 | 0.0243 | 0.0015 | 2036 | 370 | 357 | 82 | 155 | 10 | -56.6 |
| 16 | 108 | 134 | 1.24 | 2.7 | 0.0737 | 0.0151 | 0.1762 | 0.0351 | 0.0174 | 0.0008 | 1032 | 413 | 165 | 35 | 111 | 5 | -32.7 |

| Table S2 (continued) | | | | | | | | | | | | | | | | | |
| --- | --- | --- | --- | --- | --- | --- | --- | --- | --- | --- | --- | --- | --- | --- | --- | --- | --- |
| Spot | U (ppm) | Th (ppm) | Th/U | *Pb^a^ (ppm) | ^207^Pb/^206^Pb | 1σ | ^207^Pb/^235^U | 1σ | ^206^Pb/^238^U | 1σ | t_207/206_ (Ma) | 1σ | t_207/235_ (Ma) | 1σ | t_206/238_ (Ma) | 1σ | concord^b^ (%) |
| 17 | 406 | 462 | 1.14 | 121.4 | 0.0961 | 0.0029 | 2.7882 | 0.0773 | 0.2104 | 0.0036 | 1550 | 57 | 1352 | 76 | 1231 | 23 | 14.6 |
| 18 | 2557 | 1246 | 0.49 | 323.4 | 0.0711 | 0.0017 | 1.0638 | 0.0225 | 0.1085 | 0.0017 | 961 | 50 | 736 | 23 | 664 | 11 | -9.7 |
| 19 | 8942 | 8966 | 1.00 | 346.4 | 0.0567 | 0.0018 | 0.2354 | 0.0070 | 0.0301 | 0.0005 | 479 | 71 | 215 | 7 | 191 | 3 | -10.9 |
| 20 | 267 | 181 | 0.68 | 15.1 | 0.1592 | 0.0242 | 0.2704 | 0.0384 | 0.0123 | 0.0007 | 2447 | 257 | 243 | 38 | 79 | 5 | -67.5 |
|  |  |  |  |  |  |  |  |  |  |  |  |  |  |  |  |  |  |
| 07CL08 |  |  |  |  |  |  |  |  |  |  |  |  |  |  |  |  |  |
| 1 | 68 | 45 | 0.66 | 10.4 | 0.0669 | 0.0053 | 1.1435 | 0.0880 | 0.1240 | 0.0034 | 834 | 165 | 774 | 86 | 753 | 22 | -2.7 |
| 2 | 24 | 14 | 0.56 | 3.6 | 0.0631 | 0.0070 | 1.0874 | 0.1172 | 0.1250 | 0.0040 | 712 | 234 | 747 | 113 | 759 | 26 | 1.6 |
| 3 | 369 | 172 | 0.47 | 204.4 | 0.1615 | 0.0033 | 9.8786 | 0.1859 | 0.4437 | 0.0078 | 2471 | 35 | 2424 | 173 | 2367 | 50 | 2.0 |
| 4 | 463 | 348 | 0.75 | 204.8 | 0.1334 | 0.0030 | 6.4059 | 0.1326 | 0.3482 | 0.0062 | 2143 | 39 | 2033 | 126 | 1926 | 40 | 5.4 |
| 5 | 601 | 237 | 0.39 | 7.0 | 0.0546 | 0.0089 | 0.0760 | 0.0121 | 0.0101 | 0.0004 | 394 | 365 | 74 | 12 | 65 | 3 | -12.9 |
| 6 | 318 | 154 | 0.48 | 51.2 | 0.0777 | 0.0040 | 1.4397 | 0.0715 | 0.1345 | 0.0030 | 1138 | 103 | 906 | 70 | 813 | 19 | -10.2 |
| 7 | 426 | 316 | 0.74 | 68.6 | 0.0862 | 0.0039 | 1.4763 | 0.0641 | 0.1243 | 0.0027 | 1342 | 88 | 921 | 63 | 755 | 17 | -18.0 |
| 8 | 538 | 653 | 1.21 | 255.3 | 0.1348 | 0.0032 | 6.3552 | 0.1407 | 0.3419 | 0.0062 | 2162 | 42 | 2026 | 134 | 1896 | 40 | 6.7 |
| 9 | 1090 | 521 | 0.48 | 136.6 | 0.0997 | 0.0034 | 1.3423 | 0.0427 | 0.0977 | 0.0019 | 1618 | 63 | 864 | 42 | 601 | 12 | -30.5 |
| 10 | 188 | 81 | 0.43 | 26.8 | 0.0701 | 0.0054 | 1.1846 | 0.0889 | 0.1226 | 0.0033 | 930 | 159 | 793 | 87 | 746 | 21 | -6.0 |
| 11 | 423 | 166 | 0.39 | 66.9 | 0.0680 | 0.0029 | 1.2939 | 0.0536 | 0.1379 | 0.0028 | 870 | 89 | 843 | 53 | 833 | 18 | -1.2 |
| 12 | 427 | 254 | 0.59 | 63.9 | 0.0772 | 0.0039 | 1.3259 | 0.0648 | 0.1245 | 0.0028 | 1127 | 101 | 857 | 64 | 757 | 18 | -11.7 |
| 13 | 375 | 168 | 0.45 | 64.9 | 0.0812 | 0.0038 | 1.6100 | 0.0722 | 0.1439 | 0.0031 | 1226 | 92 | 974 | 71 | 866 | 20 | -11.1 |
| 14 | 1128 | 333 | 0.30 | 96.8 | 0.0912 | 0.0031 | 0.8908 | 0.0281 | 0.0708 | 0.0014 | 1451 | 64 | 647 | 28 | 441 | 9 | -31.8 |
| 15 | 511 | 174 | 0.34 | 167.1 | 0.1191 | 0.0031 | 4.7034 | 0.1153 | 0.2866 | 0.0053 | 1942 | 47 | 1768 | 111 | 1624 | 34 | 9.9 |
| 16 | 87 | 30 | 0.34 | 16.2 | 0.0713 | 0.0057 | 1.6182 | 0.1248 | 0.1647 | 0.0047 | 965 | 162 | 977 | 119 | 983 | 30 | 0.6 |
| 17 | 1900 | 124 | 0.07 | 340.6 | 0.1224 | 0.0027 | 2.6427 | 0.0538 | 0.1566 | 0.0028 | 1992 | 40 | 1313 | 53 | 938 | 18 | -28.6 |
| 18 | 418 | 186 | 0.45 | 65.4 | 0.0702 | 0.0043 | 1.3026 | 0.0766 | 0.1345 | 0.0032 | 935 | 125 | 847 | 75 | 814 | 21 | -3.9 |
| 19 | 873 | 706 | 0.81 | 121.7 | 0.1974 | 0.0065 | 2.8022 | 0.0846 | 0.1030 | 0.0022 | 2805 | 54 | 1356 | 82 | 632 | 14 | -53.4 |
| 20 | 298 | 165 | 0.55 | 13.8 | 0.0757 | 0.0065 | 0.3884 | 0.0320 | 0.0372 | 0.0011 | 1087 | 172 | 333 | 32 | 236 | 7 | -29.3 |

| Table S2 (continued) | | | | | | | | | | | | | | | | | |
| --- | --- | --- | --- | --- | --- | --- | --- | --- | --- | --- | --- | --- | --- | --- | --- | --- | --- |
| Spot | U (ppm) | Th (ppm) | Th/U | *Pb^a^ (ppm) | ^207^Pb/^206^Pb | 1σ | ^207^Pb/^235^U | 1σ | ^206^Pb/^238^U | 1σ | t_207/206_ (Ma) | 1σ | t_207/235_ (Ma) | 1σ | t_206/238_ (Ma) | 1σ | concord^b^ (%) |
| 07CL13 |  |  |  |  |  |  |  |  |  |  |  |  |  |  |  |  |  |
| 1 | 206 | 459 | 2.23 | 6.6 | 0.0710 | 0.0053 | 0.1793 | 0.0129 | 0.0183 | 0.0004 | 958 | 153 | 167 | 13 | 117 | 3 | -30.1 |
| 2 | 105 | 191 | 1.82 | 3.3 | 0.0878 | 0.0152 | 0.2067 | 0.0342 | 0.0171 | 0.0009 | 1377 | 333 | 191 | 34 | 109 | 6 | -42.7 |
| 3 | 1301 | 2378 | 1.83 | 32.7 | 0.0470 | 0.0024 | 0.1027 | 0.0050 | 0.0158 | 0.0003 | 51 | 122 | 99 | 5 | 101 | 2 | 2.1 |
| 4 | 464 | 356 | 0.77 | 23.6 | 0.1291 | 0.0106 | 0.5849 | 0.0448 | 0.0329 | 0.0011 | 2086 | 144 | 468 | 45 | 208 | 7 | -55.4 |
| 5 | 242 | 176 | 0.73 | 8.5 | 0.1731 | 0.0140 | 0.5006 | 0.0371 | 0.0210 | 0.0007 | 2588 | 135 | 412 | 37 | 134 | 5 | -67.5 |
| 6 | 582 | 816 | 1.40 | 16.1 | 0.0738 | 0.0081 | 0.1824 | 0.0192 | 0.0179 | 0.0006 | 1037 | 220 | 170 | 19 | 114 | 4 | -32.7 |
| 7 | 323 | 176 | 0.54 | 8.5 | 0.1423 | 0.0114 | 0.3445 | 0.0257 | 0.0176 | 0.0005 | 2256 | 138 | 301 | 26 | 112 | 3 | -62.7 |
| 8 | 1518 | 103 | 0.07 | 473.1 | 0.1517 | 0.0029 | 5.6279 | 0.0868 | 0.2691 | 0.0040 | 2365 | 33 | 1920 | 84 | 1536 | 26 | 23.1 |
| 9 | 528 | 459 | 0.87 | 198.6 | 0.1442 | 0.0038 | 5.5846 | 0.1308 | 0.2809 | 0.0048 | 2278 | 46 | 1914 | 125 | 1596 | 31 | 19.1 |
| 10 | 369 | 74 | 0.20 | 96.2 | 0.1289 | 0.0038 | 3.9846 | 0.1054 | 0.2242 | 0.0039 | 2083 | 52 | 1631 | 102 | 1304 | 25 | 27.7 |
| 11 | 1813 | 628 | 0.35 | 156.6 | 0.0761 | 0.0026 | 0.8265 | 0.0263 | 0.0788 | 0.0013 | 1097 | 69 | 612 | 26 | 489 | 9 | -20.1 |
|  |  |  |  |  |  |  |  |  |  |  |  |  |  |  |  |  |  |
| 05WD01 |  |  |  |  |  |  |  |  |  |  |  |  |  |  |  |  |  |
| 1.1 | 165 | 33 | 0.20 | 55.8 | 0.1749 | 0.0034 | 7.3940 | 0.1303 | 0.3067 | 0.0033 | 2605 | 32 | 2160 | 16 | 1724 | 16 | 20.6 |
| 2.1 | 105 | 47 | 0.45 | 63.0 | 0.1825 | 0.0026 | 11.8474 | 0.1446 | 0.4708 | 0.0041 | 2676 | 24 | 2592 | 11 | 2487 | 18 | 3.2 |
| 2.2 | 70 | 35 | 0.51 | 48.9 | 0.1848 | 0.0022 | 13.4089 | 0.1148 | 0.5261 | 0.0036 | 2697 | 19 | 2709 | 8 | 2725 | 15 | -0.4 |
| 3.1 | 165 | 54 | 0.33 | 76.0 | 0.1864 | 0.0021 | 9.0992 | 0.0710 | 0.3541 | 0.0022 | 2710 | 19 | 2348 | 7 | 1954 | 11 | 15.4 |
| 4.1 | 241 | 72 | 0.30 | 125.2 | 0.1632 | 0.0020 | 9.8940 | 0.0894 | 0.4396 | 0.0030 | 2489 | 20 | 2425 | 8 | 2349 | 13 | 2.6 |
| 5.1 | 132 | 62 | 0.47 | 75.2 | 0.1649 | 0.0020 | 10.5037 | 0.0967 | 0.4621 | 0.0032 | 2506 | 20 | 2480 | 9 | 2449 | 14 | 1.0 |
| 6.1 | 430 | 163 | 0.38 | 38.0 | 0.1180 | 0.0048 | 0.7778 | 0.0295 | 0.0478 | 0.0007 | 1926 | 71 | 584 | 17 | 301 | 4 | -48.5 |
| 7.1 | 635 | 214 | 0.34 | 163.1 | 0.1670 | 0.0023 | 4.7531 | 0.0519 | 0.2064 | 0.0015 | 2528 | 23 | 1777 | 9 | 1210 | 8 | 42.3 |
| 8.1 | 109 | 47 | 0.43 | 61.0 | 0.1789 | 0.0025 | 10.8673 | 0.1268 | 0.4407 | 0.0036 | 2642 | 23 | 2512 | 11 | 2354 | 16 | 5.2 |
| 9.1 | 117 | 31 | 0.27 | 70.5 | 0.1825 | 0.0022 | 11.6884 | 0.1044 | 0.4645 | 0.0032 | 2676 | 20 | 2580 | 8 | 2459 | 14 | 3.7 |
| 10.1 | 144 | 61 | 0.42 | 95.6 | 0.1808 | 0.0019 | 11.8030 | 0.0789 | 0.4734 | 0.0028 | 2660 | 18 | 2589 | 6 | 2499 | 12 | 2.7 |
| 11.1 | 194 | 121 | 0.62 | 122.0 | 0.1640 | 0.0018 | 9.9855 | 0.0742 | 0.4418 | 0.0027 | 2497 | 19 | 2433 | 7 | 2359 | 12 | 2.6 |
| 13.1 | 1022 | 763 | 0.75 | 408.7 | 0.1551 | 0.0018 | 6.7225 | 0.0550 | 0.3143 | 0.0020 | 2403 | 20 | 2076 | 7 | 1762 | 10 | 15.8 |
| 14.1 | 223 | 92 | 0.41 | 118.8 | 0.1618 | 0.0027 | 10.2784 | 0.1519 | 0.4607 | 0.0045 | 2475 | 27 | 2460 | 14 | 2443 | 20 | 0.6 |
| 15.1 | 1301 | 701 | 0.54 | 156.6 | 0.0692 | 0.0016 | 1.1025 | 0.0224 | 0.1155 | 0.0009 | 906 | 45 | 755 | 11 | 705 | 5 | -6.6 |

| Table S2 (continued) | | | | | | | | | | | | | | | | | |
| --- | --- | --- | --- | --- | --- | --- | --- | --- | --- | --- | --- | --- | --- | --- | --- | --- | --- |
| Spot | U (ppm) | Th (ppm) | Th/U | *Pb^a^ (ppm) | ^207^Pb/^206^Pb | 1σ | ^207^Pb/^235^U | 1σ | ^206^Pb/^238^U | 1σ | t_207/206_ (Ma) | 1σ | t_207/235_ (Ma) | 1σ | t_206/238_ (Ma) | 1σ | concord^b^ (%) |
| 17.1 | 177 | 841 | 4.74 | 11.1 | 0.0562 | 0.0026 | 0.2775 | 0.0125 | 0.0358 | 0.0004 | 459 | 101 | 249 | 10 | 227 | 3 | -8.8 |
| 18.1 | 236 | 122 | 0.52 | 128.3 | 0.1623 | 0.0019 | 9.8031 | 0.0863 | 0.4380 | 0.0029 | 2480 | 20 | 2416 | 8 | 2342 | 13 | 2.6 |
|  |  |  |  |  |  |  |  |  |  |  |  |  |  |  |  |  |  |
| 05WD15 |  |  |  |  |  |  |  |  |  |  |  |  |  |  |  |  |  |
| 1.1 | 1440 | 1481 | 1.03 | 271.2 | 0.0657 | 0.0012 | 1.1596 | 0.0135 | 0.1281 | 0.0007 | 796 | 20 | 782 | 5 | 777 | 4 | -0.6 |
| 2.1 | 379 | 31 | 0.08 | 91.9 | 0.0683 | 0.0021 | 1.1663 | 0.0276 | 0.1238 | 0.0008 | 878 | 23 | 785 | 8 | 753 | 5 | -4.1 |
| 3.1 | 360 | 228 | 0.63 | 158.4 | 0.1604 | 0.0017 | 6.8180 | 0.0401 | 0.3084 | 0.0017 | 2459 | 17 | 2088 | 5 | 1733 | 8 | 17.8 |
| 4.1 | 981 | 398 | 0.41 | 232.4 | 0.1545 | 0.0022 | 6.5056 | 0.0771 | 0.3055 | 0.0024 | 2396 | 24 | 2047 | 10 | 1718 | 12 | 17.0 |
| 5.1 | 275 | 7 | 0.03 | 11.7 | 0.0678 | 0.0019 | 0.3333 | 0.0085 | 0.0357 | 0.0003 | 861 | 56 | 292 | 7 | 226 | 2 | -22.6 |
| 6.1 | 757 | 177 | 0.23 | 214.0 | 0.1755 | 0.0020 | 4.7112 | 0.0346 | 0.1947 | 0.0011 | 2610 | 19 | 1769 | 6 | 1147 | 6 | 47.5 |
| 7.1 | 328 | 321 | 0.98 | 142.2 | 0.1747 | 0.0021 | 7.3166 | 0.0621 | 0.3038 | 0.0019 | 2603 | 20 | 2151 | 8 | 1710 | 10 | 21.0 |
| 8.1 | 52 | 50 | 0.95 | 1.7 | 0.0637 | 0.0038 | 0.1663 | 0.0213 | 0.0189 | 0.0006 | 733 | 126 | 156 | 19 | 121 | 4 | -22.4 |
| 9.1 | 597 | 85 | 0.14 | 193.8 | 0.1627 | 0.0026 | 6.7998 | 0.0900 | 0.3031 | 0.0025 | 2484 | 26 | 2086 | 12 | 1706 | 13 | 19.1 |
| 10.1 | 562 | 226 | 0.40 | 146.8 | 0.1635 | 0.0027 | 6.8804 | 0.0982 | 0.3053 | 0.0027 | 2492 | 28 | 2096 | 13 | 1717 | 13 | 18.9 |
| 11.1 | 949 | 40 | 0.04 | 17.0 | 0.0483 | 0.0009 | 0.1102 | 0.0018 | 0.0166 | 0.0001 | 113 | 43 | 106 | 2 | 106 | 1 | 0.0 |
| 12.1 | 890 | 4070 | 4.57 | 149.1 | 0.0644 | 0.0251 | 0.2892 | 0.0933 | 0.0326 | 0.0010 | 756 | 69 | 258 | 28 | 207 | 6 | -19.8 |
| 13.1 | 119 | 48 | 0.41 | 53.4 | 0.1729 | 0.0107 | 10.2014 | 0.1652 | 0.4280 | 0.0064 | 2586 | 99 | 2453 | 39 | 2297 | 24 | 5.4 |
| 14.1 | 1013 | 44 | 0.04 | 37.3 | 0.0669 | 0.0013 | 0.3196 | 0.0053 | 0.0347 | 0.0002 | 833 | 39 | 282 | 4 | 220 | 1 | -22.0 |
| 16.1 | 183 | 5 | 0.03 | 6.6 | 0.0588 | 0.0026 | 0.2602 | 0.0109 | 0.0321 | 0.0004 | 560 | 92 | 235 | 9 | 204 | 2 | -13.2 |
| 17.1 | 383 | 571 | 1.49 | 276.6 | 0.1773 | 0.0019 | 11.5442 | 0.0753 | 0.4722 | 0.0027 | 2628 | 18 | 2568 | 6 | 2493 | 12 | 2.3 |
|  |  |  |  |  |  |  |  |  |  |  |  |  |  |  |  |  |  |
| 05WD16 |  |  |  |  |  |  |  |  |  |  |  |  |  |  |  |  |  |
| 1.1 | 173 | 53 | 0.30 | 2.1 | 0.0716 | 0.0073 | 0.1208 | 0.0119 | 0.0122 | 0.0003 | 973 | 195 | 116 | 11 | 79 | 2 | -31.9 |
| 2.1 | 163 | 2 | 0.01 | 6.2 | 0.0513 | 0.0016 | 0.2407 | 0.0072 | 0.0340 | 0.0003 | 255 | 72 | 219 | 6 | 216 | 2 | -1.4 |
| 3.1 | 708 | 800 | 1.13 | 30.6 | 0.0658 | 0.0016 | 0.2886 | 0.0063 | 0.0318 | 0.0003 | 798 | 50 | 257 | 5 | 202 | 2 | -21.4 |
| 4.1 | 134 | 219 | 1.64 | 22.8 | 0.0720 | 0.0019 | 1.1576 | 0.0281 | 0.1166 | 0.0011 | 985 | 52 | 781 | 13 | 711 | 6 | -9.0 |
| 5.1 | 202 | 462 | 2.28 | 5.0 | 0.0510 | 0.0046 | 0.0911 | 0.0080 | 0.0130 | 0.0003 | 243 | 194 | 89 | 7 | 83 | 2 | -6.7 |

| Table S2 (continued) | | | | | | | | | | | | | | | | | |
| --- | --- | --- | --- | --- | --- | --- | --- | --- | --- | --- | --- | --- | --- | --- | --- | --- | --- |
| Spot | U (ppm) | Th (ppm) | Th/U | *Pb^a^ (ppm) | ^207^Pb/^206^Pb | 1σ | ^207^Pb/^235^U | 1σ | ^206^Pb/^238^U | 1σ | t_207/206_ (Ma) | 1σ | t_207/235_ (Ma) | 1σ | t_206/238_ (Ma) | 1σ | concord^b^ (%) |
| 6.1 | 167 | 345 | 2.06 | 3.9 | 0.0559 | 0.0034 | 0.0996 | 0.0058 | 0.0129 | 0.0002 | 449 | 129 | 96 | 5 | 83 | 1 | -13.5 |
| 8.1 | 29 | 32 | 1.13 | 5.2 | 0.0670 | 0.0017 | 1.1269 | 0.0261 | 0.1220 | 0.0010 | 837 | 51 | 766 | 12 | 742 | 6 | -3.1 |
| 9.1 | 184 | 338 | 1.84 | 4.4 | 0.0544 | 0.0028 | 0.0982 | 0.0048 | 0.0131 | 0.0002 | 386 | 110 | 95 | 4 | 84 | 1 | -11.6 |
| 10.1 | 759 | 413 | 0.54 | 29.7 | 0.0613 | 0.0014 | 0.2636 | 0.0055 | 0.0312 | 0.0002 | 649 | 49 | 238 | 4 | 198 | 1 | -16.8 |
| 11.1 | 120 | 124 | 1.04 | 16.5 | 0.0687 | 0.0030 | 0.9017 | 0.0374 | 0.0951 | 0.0013 | 891 | 87 | 653 | 20 | 586 | 8 | -10.3 |
| 12.1 | 267 | 147 | 0.55 | 104.3 | 0.1509 | 0.0017 | 6.3079 | 0.0500 | 0.3031 | 0.0018 | 2356 | 20 | 2020 | 7 | 1707 | 9 | 16.6 |
| 13.1 | 144 | 218 | 1.52 | 3.1 | 0.0582 | 0.0032 | 0.1051 | 0.0056 | 0.0131 | 0.0002 | 536 | 118 | 102 | 5 | 84 | 1 | -17.6 |
| 14.1 | 438 | 152 | 0.35 | 186.0 | 0.1656 | 0.0022 | 9.7300 | 0.1026 | 0.4262 | 0.0032 | 2514 | 22 | 2410 | 10 | 2288 | 14 | 4.3 |
| 15.1 | 3359 | 928 | 0.28 | 1342.4 | 0.0702 | 0.0129 | 1.1969 | 0.1845 | 0.1238 | 0.0020 | 933 | 33 | 799 | 18 | 752 | 11 | -5.9 |
| 16.1 | 143 | 62 | 0.43 | 81.7 | 0.1665 | 0.0019 | 9.8834 | 0.0784 | 0.4306 | 0.0027 | 2522 | 19 | 2424 | 7 | 2309 | 12 | 4.0 |
|  |  |  |  |  |  |  |  |  |  |  |  |  |  |  |  |  |  |
| 06SW09 |  |  |  |  |  |  |  |  |  |  |  |  |  |  |  |  |  |
| 1 | 264 | 38 | 0.14 |  | 0.0531 | 0.0012 | 0.2744 | 0.0054 | 0.0375 | 0.0003 | 334 | 49 | 246 | 5 | 237 | 2 | -3.7 |
| 2 | 135 | 44 | 0.32 |  | 0.0642 | 0.0019 | 0.9075 | 0.0254 | 0.1026 | 0.0010 | 747 | 63 | 656 | 25 | 629 | 6 | -4.1 |
| 3 | 169 | 208 | 1.23 |  | 0.0655 | 0.0011 | 1.0181 | 0.0157 | 0.1127 | 0.0008 | 791 | 37 | 713 | 16 | 689 | 5 | -3.4 |
| 4 | 111 | 92 | 0.83 |  | 0.0668 | 0.0013 | 1.2111 | 0.0205 | 0.1316 | 0.0010 | 831 | 39 | 806 | 21 | 797 | 7 | -1.1 |
| 5 | 62 | 74 | 1.21 |  | 0.0653 | 0.0017 | 1.0690 | 0.0258 | 0.1187 | 0.0011 | 784 | 54 | 738 | 26 | 723 | 7 | -2.0 |
| 6 | 255 | 296 | 1.16 |  | 0.0654 | 0.0013 | 1.1595 | 0.0216 | 0.1285 | 0.0010 | 788 | 43 | 782 | 22 | 780 | 7 | -0.3 |
| 7 | 373 | 618 | 1.66 |  | 0.0664 | 0.0011 | 0.9322 | 0.0136 | 0.1018 | 0.0007 | 820 | 36 | 669 | 14 | 625 | 5 | -6.6 |
| 8 | 585 | 762 | 1.30 |  | 0.0657 | 0.0010 | 0.9227 | 0.0119 | 0.1019 | 0.0007 | 797 | 33 | 664 | 12 | 625 | 5 | -5.9 |
| 9 | 130 | 47 | 0.36 |  | 0.0664 | 0.0026 | 0.7487 | 0.0270 | 0.0818 | 0.0008 | 817 | 83 | 567 | 27 | 507 | 5 | -10.6 |
| 10 | 116 | 27 | 0.23 |  | 0.0574 | 0.0022 | 0.3050 | 0.0109 | 0.0385 | 0.0004 | 507 | 83 | 270 | 11 | 244 | 3 | -9.6 |
| 11 | 220 | 120 | 0.55 |  | 0.0658 | 0.0013 | 1.1412 | 0.0210 | 0.1258 | 0.0010 | 800 | 43 | 773 | 21 | 764 | 7 | -1.2 |
| 12 | 126 | 75 | 0.60 |  | 0.0595 | 0.0021 | 0.4199 | 0.0143 | 0.0512 | 0.0005 | 585 | 77 | 356 | 14 | 322 | 3 | -9.6 |
| 13 | 246 | 311 | 1.26 |  | 0.0679 | 0.0016 | 0.7222 | 0.0140 | 0.0772 | 0.0006 | 865 | 49 | 552 | 14 | 479 | 4 | -13.2 |
| 14 | 46 | 54 | 1.17 |  | 0.0631 | 0.0020 | 1.0063 | 0.0305 | 0.1156 | 0.0012 | 713 | 68 | 707 | 30 | 705 | 8 | -0.3 |
| 15 | 405 | 532 | 1.31 |  | 0.0646 | 0.0013 | 0.9291 | 0.0164 | 0.1043 | 0.0008 | 762 | 41 | 667 | 17 | 639 | 5 | -4.2 |

| Table S2 (continued) | | | | | | | | | | | | | | | | | |
| --- | --- | --- | --- | --- | --- | --- | --- | --- | --- | --- | --- | --- | --- | --- | --- | --- | --- |
| Spot | U (ppm) | Th (ppm) | Th/U | *Pb^a^ (ppm) | ^207^Pb/^206^Pb | 1σ | ^207^Pb/^235^U | 1σ | ^206^Pb/^238^U | 1σ | t_207/206_ (Ma) | 1σ | t_207/235_ (Ma) | 1σ | t_206/238_ (Ma) | 1σ | concord^b^ (%) |
| 17 | 338 | 437 | 1.29 |  | 0.0656 | 0.0009 | 1.1544 | 0.0121 | 0.1275 | 0.0009 | 795 | 27 | 779 | 12 | 774 | 6 | -0.6 |
| 18 | 69 | 52 | 0.75 |  | 0.0674 | 0.0015 | 0.9243 | 0.0190 | 0.0995 | 0.0008 | 850 | 47 | 665 | 19 | 611 | 5 | -8.1 |
| 19 | 82 | 74 | 0.91 |  | 0.0652 | 0.0013 | 1.1262 | 0.0204 | 0.1253 | 0.0010 | 780 | 42 | 766 | 21 | 761 | 6 | -0.7 |
| 20 | 126 | 115 | 0.92 |  | 0.0676 | 0.0013 | 0.9015 | 0.0151 | 0.0967 | 0.0007 | 857 | 39 | 653 | 15 | 595 | 5 | -8.9 |
|  |  |  |  |  |  |  |  |  |  |  |  |  |  |  |  |  |  |
| 06SW11 |  |  |  |  |  |  |  |  |  |  |  |  |  |  |  |  |  |
| 1 | 127 | 90 | 0.20 | 17.7 | 0.0642 | 0.0047 | 0.8185 | 0.0577 | 0.0924 | 0.0019 | 750 | 153 | 607 | 57 | 570 | 12 | -6.1 |
| 2 | 766 | 472 | 0.24 | 114.3 | 0.0666 | 0.0018 | 0.9034 | 0.0236 | 0.0984 | 0.0010 | 825 | 58 | 654 | 24 | 605 | 6 | -7.5 |
| 3 | 94 | 57 | 0.33 | 19.0 | 0.0660 | 0.0043 | 0.9078 | 0.0572 | 0.0998 | 0.0018 | 805 | 136 | 656 | 56 | 613 | 12 | -6.6 |
| 4 | 526 | 206 | 0.28 | 57.0 | 0.0636 | 0.0016 | 0.6778 | 0.0163 | 0.0773 | 0.0007 | 727 | 55 | 525 | 16 | 480 | 4 | -8.6 |
| 5 | 237 | 195 | 0.36 | 69.7 | 0.1130 | 0.0028 | 3.9414 | 0.0915 | 0.2529 | 0.0030 | 1849 | 44 | 1622 | 89 | 1453 | 19 | 14.0 |
| 6 | 152 | 185 | 0.15 | 26.8 | 0.0658 | 0.0023 | 0.9363 | 0.0313 | 0.1032 | 0.0012 | 800 | 73 | 671 | 31 | 633 | 7 | -5.7 |
| 7 | 438 | 316 | 0.25 | 78.9 | 0.0655 | 0.0010 | 1.1414 | 0.0155 | 0.1265 | 0.0009 | 789 | 33 | 773 | 16 | 768 | 6 | -0.6 |
| 8 | 141 | 68 | 0.47 | 32.0 | 0.0924 | 0.0016 | 2.0738 | 0.0321 | 0.1629 | 0.0014 | 1475 | 32 | 1140 | 32 | 973 | 9 | -14.6 |
| 9 | 353 | 322 | 0.18 | 56.4 | 0.0646 | 0.0028 | 0.6060 | 0.0252 | 0.0681 | 0.0008 | 760 | 92 | 481 | 25 | 424 | 5 | -11.9 |
| 10 | 154 | 82 | 0.18 | 15.0 | 0.0636 | 0.0040 | 0.6753 | 0.0407 | 0.0770 | 0.0013 | 730 | 133 | 524 | 41 | 478 | 8 | -8.8 |
| 11 | 133 | 167 | 0.15 | 25.4 | 0.0658 | 0.0043 | 1.1397 | 0.0721 | 0.1256 | 0.0024 | 801 | 137 | 772 | 71 | 762 | 15 | -1.3 |
| 12 | 269 | 190 | 0.18 | 33.9 | 0.0645 | 0.0055 | 0.6381 | 0.0525 | 0.0717 | 0.0014 | 759 | 181 | 501 | 52 | 446 | 9 | -11.0 |
| 13 | 106 | 83 | 0.63 | 52.1 | 0.1216 | 0.0033 | 5.7673 | 0.1493 | 0.3439 | 0.0045 | 1980 | 48 | 1942 | 141 | 1905 | 29 | 2.0 |
| 14 | 466 | 227 | 0.27 | 61.4 | 0.0660 | 0.0035 | 0.9149 | 0.0458 | 0.1005 | 0.0013 | 806 | 112 | 660 | 45 | 618 | 8 | -6.4 |
| 15 | 111 | 46 | 0.24 | 11.0 | 0.0604 | 0.0052 | 0.7029 | 0.0590 | 0.0844 | 0.0019 | 618 | 186 | 541 | 58 | 522 | 12 | -3.5 |
| 16 | 652 | 916 | 0.12 | 112.9 | 0.0646 | 0.0013 | 0.8510 | 0.0148 | 0.0956 | 0.0007 | 761 | 41 | 625 | 15 | 588 | 5 | -5.9 |
| 17 | 172 | 59 | 0.24 | 14.2 | 0.0615 | 0.0072 | 0.5400 | 0.0616 | 0.0637 | 0.0019 | 658 | 251 | 438 | 61 | 398 | 12 | -9.1 |
| 18 | 396 | 179 | 0.18 | 33.1 | 0.0646 | 0.0031 | 0.5058 | 0.0232 | 0.0568 | 0.0008 | 762 | 101 | 416 | 23 | 356 | 5 | -14.4 |
| 19 | 655 | 487 | 0.22 | 107.3 | 0.0652 | 0.0013 | 1.0323 | 0.0183 | 0.1148 | 0.0009 | 782 | 41 | 720 | 18 | 700 | 6 | -2.8 |
| 20 | 203 | 89 | 0.21 | 19.0 | 0.0656 | 0.0042 | 0.6327 | 0.0386 | 0.0700 | 0.0011 | 794 | 133 | 498 | 38 | 436 | 7 | -12.4 |

^a^ *Pb means radiogenic Pb.

^b^ concord denotes the concordance between three U-Pb apparent ages, which are calculated following 100×(t_206/238_/t_207/235_-1) and 100×(t_207/206_/t_207/235_-1) for zircons younger and older than 1000 Ma, respectively.

Table S3. LA-MC-ICPMS zircon Lu-Hf isotope data for Cenozoic continental basalts

| Spot | ^176^Yb/^177^Hf | ^176^Lu/^177^Hf | ^176^Hf/^177^Hf | 2σ | U-Pb Age (Ma) | ε_Hf_(t) | 2σ | T_DM_ (Ma) | 2σ | Age Spot^a^ | |
| --- | --- | --- | --- | --- | --- | --- | --- | --- | --- | --- | --- |
| 07CL03 |  |  |  |  |  |  |  |  |  |  | |
| 1 | 0.021568 | 0.000687 | 0.282357 | 0.000037 | 781 | 2.2 | 0.7 | 1256 | 51 | 1 | |
| 2 | 0.023339 | 0.000736 | 0.282459 | 0.000025 | 795 | 6.1 | 0.5 | 1115 | 35 | 2 | |
| 3 | 0.016250 | 0.000496 | 0.282387 | 0.000015 | 783 | 3.4 | 0.3 | 1207 | 21 | 3 | |
| 4 | 0.014357 | 0.000477 | 0.282257 | 0.000035 | 787 | -1.1 | 0.6 | 1386 | 48 | 4 | |
| 5 | 0.017380 | 0.000538 | 0.282481 | 0.000026 | 788 | 6.8 | 0.5 | 1077 | 36 | 5 | |
| 6 | 0.024656 | 0.000762 | 0.282432 | 0.000031 | 803 | 5.3 | 0.6 | 1153 | 43 | 6 | |
| 7 | 0.018572 | 0.000512 | 0.282416 | 0.000015 | 787 | 4.5 | 0.3 | 1167 | 21 | 7 | |
| 8 | 0.031119 | 0.000933 | 0.282429 | 0.000025 | 796 | 4.9 | 0.5 | 1162 | 36 | 8 | |
| 10 | 0.023575 | 0.000728 | 0.282435 | 0.000016 | 781 | 4.9 | 0.3 | 1148 | 23 | 10 | |
| 11 | 0.023044 | 0.000654 | 0.282423 | 0.000018 | 764 | 4.2 | 0.3 | 1162 | 25 | 11 | |
| 12 | 0.021449 | 0.000628 | 0.282345 | 0.000037 | 741 | 0.9 | 0.7 | 1270 | 51 | 12 | |
| 13 | 0.021621 | 0.000632 | 0.282458 | 0.000060 | 809 | 6.4 | 1.1 | 1112 | 84 | 13 | |
| 14 | 0.033306 | 0.001015 | 0.282458 | 0.000025 | 760 | 5.2 | 0.4 | 1123 | 35 | 14 | |
| 15 | 0.024543 | 0.000750 | 0.282256 | 0.000052 | 735 | -2.4 | 0.9 | 1398 | 72 | 15 | |
| 16 | 0.013489 | 0.000371 | 0.282366 | 0.000015 | 718 | 1.3 | 0.3 | 1232 | 20 | 16 | |
| 17 | 0.019804 | 0.000518 | 0.282359 | 0.000016 | 750 | 1.7 | 0.3 | 1246 | 22 | 17 | |
| 18 | 0.018303 | 0.000561 | 0.282446 | 0.000016 | 714 | 4.0 | 0.3 | 1127 | 23 | 18 | |
| 19 | 0.023041 | 0.000711 | 0.282506 | 0.000028 | 753 | 6.9 | 0.5 | 1048 | 39 | 19 | |
| 20 | 0.016636 | 0.000483 | 0.282420 | 0.000015 | 768 | 4.2 | 0.3 | 1161 | 21 | 20 | |
| 07CL05 |  |  |  |  |  |  |  |  |  |  | |
| 1 | 0.109807 | 0.003165 | 0.282478 | 0.000016 | 534 | 0.3 | 0.3 | 1161 | 25 | 1 | |
| 2 | 0.023439 | 0.000592 | 0.281398 | 0.000015 | 2254 | 0.9 | 0.3 | 2564 | 21 | 2 | |
| 3 | 0.002475 | 0.000060 | 0.281434 | 0.000015 | 1937 | -4.2 | 0.3 | 2481 | 19 | 3 | |
| 4 | 0.020118 | 0.000664 | 0.282445 | 0.000027 | 901 | 8.0 | 0.5 | 1132 | 37 | 4 | |
| 5 | 0.020223 | 0.000531 | 0.281327 | 0.000013 | 2437 | 2.6 | 0.2 | 2655 | 18 | 5 | |
| 6 | 0.030019 | 0.000830 | 0.281260 | 0.000028 | 2429 | -0.4 | 0.5 | 2767 | 38 | 6 | |
| 7 | 0.107534 | 0.002497 | 0.281513 | 0.000031 | 2423 | 5.7 | 0.6 | 2534 | 45 | 7 | |
| 8 | 0.120245 | 0.002911 | 0.282448 | 0.000030 | 334 | -4.8 | 0.5 | 1198 | 44 | 8 | |
| 9 | 0.021155 | 0.000616 | 0.281324 | 0.000039 | 2211 | -2.7 | 0.7 | 2666 | 53 | 9 | |
| 10 | 0.002794 | 0.000066 | 0.281371 | 0.000037 | 2071 | -3.4 | 0.7 | 2566 | 50 | 10 | |
| 11 | 0.040595 | 0.001031 | 0.281315 | 0.000034 | 2367 | -0.2 | 0.6 | 2707 | 46 | 11 | |
| 12 | 0.081593 | 0.001878 | 0.282042 | 0.000035 | 851 | -8.1 | 0.6 | 1745 | 50 | 12 | |
| 13 | 0.044152 | 0.001369 | 0.281431 | 0.000039 | 2365 | 3.4 | 0.7 | 2571 | 53 | 13 | |
| 14 | 0.016796 | 0.000441 | 0.281126 | 0.000029 | 2330 | -6.8 | 0.5 | 2919 | 39 | 14 | |
| 07CL07 |  |  |  |  |  |  |  |  |  |  | |
| 1 | 0.064473 | 0.001928 | 0.282473 | 0.000044 | 893 | 8.0 | 0.8 | 1130 | 63 | 1 | |
| 3 | 0.045027 | 0.001269 | 0.282358 | 0.000032 | 1102 | 8.8 | 0.6 | 1273 | 46 | 3 | |
| 7 | 0.103959 | 0.003006 | 0.282027 | 0.000042 | 1526 | 4.5 | 0.8 | 1821 | 62 | 7 | |
| 8 | 0.022934 | 0.000746 | 0.281555 | 0.000027 | 1823 | -3.4 | 0.5 | 2361 | 37 | 8 | |
| 9 | 0.011088 | 0.000301 | 0.282221 | 0.000036 | 781 | -2.4 | 0.7 | 1428 | 49 | 9 | |
| 10 | 0.012684 | 0.000470 | 0.282354 | 0.000098 | 946 | -5.8 | 1.8 | 1252 | 135 | 10 | |
| 11 | 0.047569 | 0.001447 | 0.281368 | 0.000027 | 2049 | -5.9 | 0.5 | 2663 | 38 | 11 | |
| 16 | 0.036954 | 0.001090 | 0.282364 | 0.000036 | 1032 | 7.7 | 0.7 | 1258 | 51 | 16 | |
| 17 | 0.067135 | 0.001970 | 0.282021 | 0.000050 | 1550 | 5.8 | 0.9 | 1780 | 71 | 17 | |
| 18 | 0.079853 | 0.002482 | 0.282201 | 0.000038 | 961 | -0.6 | 0.7 | 1544 | 56 | 18 | |
| 19 | 0.137848 | 0.004743 | 0.282400 | 0.000088 | 479 | -4.1 | 1.6 | 1338 | 136 | 19 | |
| 07CL08 |  |  |  |  |  |  |  |  |  |  | |
| 1 | 0.024001 | 0.000869 | 0.282475 | 0.000019 | 834 | 7.4 | 0.3 | 1096 | 27 | 1 | |
| 2 | 0.013015 | 0.000447 | 0.282443 | 0.000024 | 759 | 4.9 | 0.4 | 1128 | 34 | 2 | |
| 3 | 0.022976 | 0.000738 | 0.281288 | 0.000029 | 2471 | 1.7 | 0.5 | 2723 | 39 | 3 | |
|  |  |  |  |  |  |  |  |  |  |  | |
| Table S3 (continued) | | | | | | | | | | |  |
| Spot | ^176^Yb/^177^Hf | ^176^Lu/^177^Hf | ^176^Hf/^177^Hf | 2σ | U-Pb Age (Ma) | ε_Hf_(t) | 2σ | T_DM_ (Ma) | 2σ | Age Spot^a^ | |
| 4 | 0.060868 | 0.001816 | 0.281575 | 0.000031 | 2143 | 3.0 | 0.6 | 2400 | 44 | 4 | |
| 5 | 0.049295 | 0.001712 | 0.282378 | 0.000059 | 65 | -12.6 | 1.1 | 1260 | 84 | 5 | |
| 6 | 0.064729 | 0.002598 | 0.282318 | 0.000054 | 1138 | 7.2 | 1.0 | 1378 | 79 | 6 | |
| 8 | 0.031367 | 0.001106 | 0.281700 | 0.000140 | 2162 | 8.8 | 2.5 | 2184 | 194 | 8 | |
| 10 | 0.044654 | 0.001613 | 0.282372 | 0.000081 | 930 | 5.4 | 1.5 | 1265 | 115 | 10 | |
| 11 | 0.077936 | 0.003188 | 0.282369 | 0.000119 | 870 | 3.1 | 2.1 | 1325 | 177 | 11 | |
| 12 | 0.078689 | 0.003131 | 0.282380 | 0.000098 | 1127 | 8.8 | 1.8 | 1306 | 145 | 12 | |
| 15 | 0.050953 | 0.001558 | 0.281599 | 0.000053 | 1942 | -0.2 | 1.0 | 2350 | 74 | 15 | |
| 16 | 0.013062 | 0.000402 | 0.282376 | 0.000050 | 983 | 7.5 | 0.9 | 1219 | 68 | 16 | |
| 17 | 0.025272 | 0.000774 | 0.281613 | 0.000049 | 1992 | 2.4 | 0.9 | 2284 | 66 | 17 | |
| 20 | 0.042055 | 0.001463 | 0.282387 | 0.000057 | 1087 | 9.4 | 1.0 | 1238 | 82 | 20 | |
| 07CL13 |  |  |  |  |  |  |  |  |  |  | |
| 1 | 0.026581 | 0.000732 | 0.282253 | 0.000016 | 958 | 2.3 | 0.3 | 1401 | 22 | 1 | |
| 6 | 0.092951 | 0.002587 | 0.282200 | 0.000050 | 1037 | 0.9 | 0.9 | 1550 | 73 | 6 | |
| 7 | 0.042081 | 0.001264 | 0.282488 | 0.000037 | 112 | -7.7 | 0.7 | 1089 | 53 | 7 | |
| 8 | 0.023489 | 0.000685 | 0.281273 | 0.000025 | 2365 | -1.2 | 0.4 | 2740 | 34 | 8 | |
| 9 | 0.008853 | 0.000240 | 0.281290 | 0.000035 | 2278 | -1.8 | 0.6 | 2686 | 47 | 9 | |
| 11 | 0.053153 | 0.001665 | 0.282435 | 0.000097 | 489 | -1.7 | 1.8 | 1176 | 139 | 11 | |
| 05HF15 |  |  |  |  |  |  |  |  |  |  | |
| 1 | 0.074126 | 0.002277 | 0.281140 | 0.000028 | 2597 | -3.5 | 0.5 | 3044 | 39 | 1.1 | |
| 3 | 0.011515 | 0.000472 | 0.281514 | 0.000070 | 1926 | -2.2 | 1.3 | 2400 | 94 | 4.2 | |
| 4 | 0.014282 | 0.000600 | 0.281569 | 0.000057 | 1430 | -11.4 | 1.0 | 2332 | 77 | 4.1 | |
| 5 | 0.050100 | 0.001622 | 0.281221 | 0.000050 | 2190 | -8.3 | 0.9 | 2879 | 68 | 11.1 | |
| 6 | 0.004707 | 0.000154 | 0.281234 | 0.000034 | 2276 | -3.7 | 0.6 | 2754 | 46 | 9.1 | |
| 8 | 0.006516 | 0.000221 | 0.281211 | 0.000038 | 2740 | 6.0 | 0.7 | 2790 | 50 | 5.1 | |
| 05HF18 |  |  |  |  |  |  |  |  |  |  | |
| 1 | 0.018350 | 0.000532 | 0.281526 | 0.000029 | 1844 | -3.7 | 0.5 | 2388 | 39 | 10.1 | |
| 2 | 0.030495 | 0.000892 | 0.281501 | 0.000031 | 1850 | -4.9 | 0.6 | 2444 | 42 | 8.1 | |
| 3 | 0.034486 | 0.001054 | 0.282474 | 0.000043 | 729 | 5.0 | 0.8 | 1103 | 60 | 9.1 | |
| 4 | 0.012008 | 0.000349 | 0.281951 | 0.000033 | 602 | -15.9 | 0.6 | 1800 | 45 | 11.1 | |
| 5 | 0.099709 | 0.002972 | 0.282125 | 0.000040 | 777 | -7.3 | 0.7 | 1676 | 59 | 7.1 | |
| 6 | 0.040732 | 0.001342 | 0.282034 | 0.000030 | 507 | -15.4 | 0.5 | 1731 | 42 | 1.1 | |
| 7 | 0.031621 | 0.001097 | 0.282112 | 0.000037 | 771 | -6.9 | 0.7 | 1611 | 52 | 2.1 | |
| 8 | 0.014469 | 0.000428 | 0.281647 | 0.000029 | 1931 | 2.7 | 0.5 | 2217 | 39 | 3.1 | |
| 9 | 0.001085 | 0.000034 | 0.282383 | 0.000042 | 200 | -9.4 | 0.8 | 1198 | 58 | 5.1 | |
| 12 | 0.056417 | 0.001885 | 0.282382 | 0.000111 | 450 | -4.5 | 2.0 | 1260 | 159 | 4.1 | |
| 05NS04 |  |  |  |  |  |  |  |  |  |  | |
| 1 | 0.010299 | 0.000326 | 0.281398 | 0.000015 | 1874 | -7.3 | 0.3 | 2547 | 20 | 1.1 | |
| 2 | 0.024550 | 0.000636 | 0.281320 | 0.000019 | 2032 | -6.9 | 0.3 | 2673 | 26 | 5.1 | |
| 3 | 0.005658 | 0.000183 | 0.281282 | 0.000016 | 2034 | -7.6 | 0.3 | 2692 | 21 | 4.1 | |
| 5 | 0.037346 | 0.001293 | 0.282087 | 0.000026 | 688 | -9.7 | 0.5 | 1655 | 36 | 7.1 | |
| 6 | 0.047071 | 0.001445 | 0.281287 | 0.000057 | 2047 | -8.8 | 1.0 | 2775 | 79 | 9.1 | |
| 7 | 0.049756 | 0.001680 | 0.282131 | 0.000030 | 1311 | 4.9 | 0.5 | 1609 | 42 | 8.1 | |
| 05WD15 |  |  |  |  |  |  |  |  |  |  | |
| 1 | 0.012164 | 0.000529 | 0.281318 | 0.000021 | 2003 | -7.5 | 0.4 | 2668 | 28 | 3.1S | |
| 4 | 0.011084 | 0.000555 | 0.281371 | 0.000018 | 2560 | 6.9 | 0.3 | 2598 | 24 | 5.1S | |
| 6 | 0.016837 | 0.000797 | 0.281269 | 0.000018 | 2551 | 2.7 | 0.3 | 2754 | 24 | 8.1S | |
| 9 | 0.022866 | 0.001061 | 0.281251 | 0.000020 | 2680 | 4.5 | 0.4 | 2796 | 27 | 10.1S | |
| 11 | 0.021647 | 0.001009 | 0.281332 | 0.000021 | 2610 | 5.9 | 0.4 | 2682 | 29 | 6.1L | |
| 12 | 0.024048 | 0.001060 | 0.281257 | 0.000028 | 2586 | 2.6 | 0.5 | 2789 | 39 | 13.1L | |
| 17 | 0.021854 | 0.001068 | 0.281290 | 0.000023 | 2603 | 4.1 | 0.4 | 2744 | 31 | 7.1L | |
| 19 | 0.016681 | 0.000820 | 0.281378 | 0.000019 | 2459 | 4.5 | 0.3 | 2607 | 26 | 3.1L | |
|  |  |  |  |  |  |  |  |  |  |  | |
| Table S3 (continued) | | | | | | | | | | |  |
| Spot | ^176^Yb/^177^Hf | ^176^Lu/^177^Hf | ^176^Hf/^177^Hf | 2σ | U-Pb Age (Ma) | ε_Hf_(t) | 2σ | T_DM_ (Ma) | 2σ | Age Spot^a^ | |
| 05WD31 |  |  |  |  |  |  |  |  |  |  | |
| 1 | 0.171939 | 0.005014 | 0.282405 | 0.000018 | 1214 | 9.9 | 0.3 | 1341 | 29 | 1 | |
| 2 | 0.079303 | 0.002442 | 0.281946 | 0.000037 | 832 | -12.2 | 0.7 | 1910 | 54 | 2 | |
| 3 | 0.008657 | 0.000296 | 0.281808 | 0.000059 | 1039 | -11.4 | 1.1 | 1992 | 79 | 3 | |
| 4 | 0.006508 | 0.000206 | 0.281342 | 0.000030 | 1802 | -10.7 | 0.5 | 2613 | 39 | 4 | |
| 5 | 0.023144 | 0.000723 | 0.281008 | 0.000022 | 2250 | -13.2 | 0.4 | 3100 | 30 | 5 | |
| 7 | 0.034581 | 0.001043 | 0.280893 | 0.000031 | 2351 | -15.5 | 0.6 | 3280 | 42 | 7 | |
| 8 | 0.096596 | 0.003280 | 0.282106 | 0.000025 | 1229 | 1.0 | 0.4 | 1719 | 37 | 8 | |
| 9 | 0.011775 | 0.000445 | 0.281157 | 0.000035 | 2344 | -5.4 | 0.6 | 2878 | 47 | 9 | |
| 10 | 0.034589 | 0.001303 | 0.281352 | 0.000037 | 2607 | 6.0 | 0.7 | 2676 | 51 | 10 | |
| 11 | 0.015884 | 0.000498 | 0.280944 | 0.000021 | 2488 | -9.8 | 0.4 | 3166 | 28 | 11 | |
| 12 | 0.065446 | 0.002032 | 0.282489 | 0.000039 | 102 | -7.9 | 0.7 | 1111 | 56 | 12 | |
| 13 | 0.014806 | 0.000474 | 0.280987 | 0.000023 | 2459 | -8.9 | 0.4 | 3107 | 30 | 13 | |
| 05WD36 |  |  |  |  |  |  |  |  |  |  | |
| 1 | 0.011335 | 0.000430 | 0.281237 | 0.000018 | 2639 | 4.2 | 0.3 | 2770 | 24 | 1 | |
| 2 | 0.022518 | 0.000777 | 0.281373 | 0.000028 | 2287 | 0.5 | 0.5 | 2611 | 38 | 2 | |
| 3 | 0.029363 | 0.000922 | 0.280960 | 0.000018 | 2237 | -15.5 | 0.3 | 3181 | 24 | 3 | |
| 5 | 0.018599 | 0.000684 | 0.281262 | 0.000017 | 2676 | 5.5 | 0.3 | 2754 | 23 | 5 | |
| 6 | 0.026040 | 0.000891 | 0.281277 | 0.000020 | 2626 | 4.5 | 0.4 | 2749 | 28 | 6 | |
| 7 | 0.045416 | 0.001460 | 0.282453 | 0.000029 | 235 | -6.3 | 0.5 | 1144 | 41 | 7 | |
| 8 | 0.030961 | 0.000978 | 0.281327 | 0.000030 | 2600 | 5.5 | 0.5 | 2687 | 41 | 8 | |
| 9 | 0.028533 | 0.000952 | 0.281282 | 0.000023 | 2635 | 4.8 | 0.4 | 2747 | 32 | 9 | |
| 10 | 0.024309 | 0.000943 | 0.281290 | 0.000024 | 2686 | 6.3 | 0.4 | 2734 | 33 | 10 | |
| 11 | 0.043637 | 0.001492 | 0.281237 | 0.000035 | 2431 | -2.3 | 0.6 | 2847 | 48 | 11 | |
| 12 | 0.023797 | 0.000871 | 0.281296 | 0.000060 | 2484 | 2.0 | 1.1 | 2721 | 82 | 12 | |
| 13 | 0.031148 | 0.000921 | 0.281147 | 0.000019 | 1945 | -15.4 | 0.3 | 2927 | 25 | 13 | |
| 14 | 0.021940 | 0.000787 | 0.281365 | 0.000036 | 2476 | 4.4 | 0.7 | 2622 | 50 | 14 | |
| 15 | 0.016159 | 0.000578 | 0.281053 | 0.000025 | 2062 | -15.6 | 0.5 | 3027 | 34 | 15 | |
| 16 | 0.017576 | 0.000603 | 0.281372 | 0.000042 | 2469 | 4.8 | 0.8 | 2600 | 56 | 16 | |
| 17 | 0.056831 | 0.001918 | 0.282080 | 0.000040 | 1147 | -0.6 | 0.7 | 1692 | 56 | 17 | |
| 19 | 0.015413 | 0.000591 | 0.281312 | 0.000042 | 2497 | 3.4 | 0.8 | 2680 | 57 | 19 | |
| 20 | 0.063472 | 0.002327 | 0.281090 | 0.000041 | 2410 | -9.3 | 0.7 | 3119 | 58 | 20 | |
| 21 | 0.026433 | 0.001077 | 0.281352 | 0.000036 | 2450 | 2.9 | 0.6 | 2660 | 49 | 21 | |
| 22 | 0.034385 | 0.001182 | 0.281274 | 0.000029 | 2304 | -3.3 | 0.5 | 2774 | 40 | 22 | |
| 23 | 0.017876 | 0.000728 | 0.281206 | 0.000042 | 2523 | -0.1 | 0.8 | 2833 | 56 | 23 | |
| 24 | 0.021532 | 0.000696 | 0.281198 | 0.000029 | 2161 | -8.4 | 0.5 | 2841 | 39 | 24 | |
| 25 | 0.041650 | 0.001695 | 0.281705 | 0.000031 | 1665 | -2.6 | 0.6 | 2210 | 44 | 25 | |
| 06SW05 |  |  |  |  |  |  |  |  |  |  | |
| 1 | 0.178541 | 0.002998 | 0.281261 | 0.000031 | 2383 | -4.9 | 0.6 | 2932 | 44 | 2.1 | |
| 2 | 0.022979 | 0.000485 | 0.281338 | 0.000028 | 2464 | 3.7 | 0.5 | 2638 | 38 | 1.1 | |
| 4 | 0.029236 | 0.000595 | 0.281278 | 0.000025 | 2458 | 1.2 | 0.5 | 2727 | 34 | 3.1 | |
| 5 | 0.102753 | 0.001975 | 0.281400 | 0.000028 | 2063 | -5.2 | 0.5 | 2656 | 39 | 4.1 | |
| 7 | 0.080739 | 0.002869 | 0.281284 | 0.000041 | 2340 | -4.8 | 0.7 | 2888 | 59 | 10.1 | |
| 8 | 0.091056 | 0.002447 | 0.282497 | 0.000038 | 119 | -7.3 | 0.7 | 1112 | 55 | 6.1 | |
| 9 | 0.033138 | 0.000960 | 0.282447 | 0.000032 | 218 | -6.9 | 0.6 | 1138 | 45 | 8.1 | |
| 10 | 0.165441 | 0.003040 | 0.281557 | 0.000032 | 2310 | 4.0 | 0.6 | 2508 | 46 | 5.1 | |
| 06SW07 |  |  |  |  |  |  |  |  |  |  | |
| 1 | 0.015022 | 0.000450 | 0.281314 | 0.000028 | 2493 | 3.6 | 0.5 | 2668 | 38 | 1.1 | |
| 2 | 0.063891 | 0.002157 | 0.282381 | 0.000040 | 478 | -4.0 | 0.7 | 1270 | 57 | 5.1 | |
| 4 | 0.046024 | 0.001587 | 0.282388 | 0.000063 | 852 | 4.3 | 1.1 | 1241 | 90 | 3.1 | |
| 5 | 0.038574 | 0.001330 | 0.282109 | 0.000048 | 509 | -12.7 | 0.9 | 1626 | 68 | 6.1 | |
|  |  |  |  |  |  |  |  |  |  |  | |
|  |  |  |  |  |  |  |  |  |  |  | |
| Table S3 (continued) | | | | | | | | | | |  |
| Spot | ^176^Yb/^177^Hf | ^176^Lu/^177^Hf | ^176^Hf/^177^Hf | 2σ | U-Pb Age (Ma) | ε_Hf_(t) | 2σ | T_DM_ (Ma) | 2σ | Age Spot^a^ | |
| 06SW09 |  |  |  |  |  |  |  |  |  |  | |
| 2 | 0.062313 | 0.001326 | 0.281862 | 0.000041 | 629 | -18.9 | 0.7 | 1970 | 57 | 2 | |
| 3 | 0.156735 | 0.003169 | 0.281876 | 0.000027 | 689 | -18.0 | 0.5 | 2051 | 39 | 3 | |
| 4 | 0.063611 | 0.001098 | 0.281934 | 0.000028 | 797 | -12.7 | 0.5 | 1860 | 38 | 4 | |
| 5 | 0.083889 | 0.001364 | 0.281900 | 0.000029 | 723 | -15.6 | 0.5 | 1919 | 41 | 5 | |
| 6 | 0.152606 | 0.002719 | 0.282025 | 0.000034 | 780 | -10.6 | 0.6 | 1810 | 50 | 6 | |
| 7 | 0.164972 | 0.002774 | 0.282123 | 0.000031 | 820 | -6.4 | 0.6 | 1670 | 45 | 7 | |
| 8 | 0.242329 | 0.003901 | 0.282036 | 0.000031 | 797 | -10.5 | 0.6 | 1854 | 47 | 8 | |
| 9 | 0.066154 | 0.001402 | 0.282522 | 0.000030 | 814 | 8.4 | 0.5 | 1045 | 43 | 9 | |
| 10 | 0.080404 | 0.001395 | 0.282075 | 0.000034 | 507 | -14.0 | 0.6 | 1676 | 47 | 10 | |
| 11 | 0.114674 | 0.002039 | 0.282035 | 0.000036 | 764 | -10.3 | 0.7 | 1763 | 52 | 11 | |
| 12 | 0.043699 | 0.000728 | 0.282357 | 0.000039 | 585 | -2.1 | 0.7 | 1256 | 54 | 12 | |
| 13 | 0.057777 | 0.000944 | 0.281940 | 0.000024 | 865 | -10.9 | 0.4 | 1844 | 33 | 13 | |
| 14 | 0.080436 | 0.001313 | 0.281909 | 0.000031 | 705 | -15.6 | 0.6 | 1904 | 43 | 14 | |
| 15 | 0.197859 | 0.004210 | 0.282045 | 0.000037 | 762 | -11.0 | 0.7 | 1857 | 56 | 15 | |
| 06SW11 |  |  |  |  |  |  |  |  |  |  | |
| 1 | 0.073841 | 0.001979 | 0.281954 | 0.000032 | 750 | -13.4 | 0.6 | 1875 | 46 | 1 | |
| 2 | 0.121500 | 0.002518 | 0.281968 | 0.000024 | 825 | -11.6 | 0.4 | 1882 | 34 | 2 | |
| 3 | 0.055591 | 0.001331 | 0.282029 | 0.000027 | 805 | -9.2 | 0.5 | 1737 | 38 | 3 | |
| 4 | 0.127856 | 0.002629 | 0.281983 | 0.000023 | 727 | -13.2 | 0.4 | 1866 | 33 | 4 | |
| 6 | 0.101191 | 0.002057 | 0.282016 | 0.000029 | 800 | -10.2 | 0.5 | 1790 | 42 | 6 | |
| 7 | 0.114059 | 0.002292 | 0.281924 | 0.000026 | 768 | -14.2 | 0.5 | 1933 | 37 | 7 | |
| 8 | 0.090511 | 0.002112 | 0.281800 | 0.000029 | 1475 | -3.7 | 0.5 | 2100 | 41 | 8 | |
| 9 | 0.103637 | 0.003028 | 0.281996 | 0.000035 | 760 | -12.2 | 0.6 | 1868 | 51 | 9 | |
| 10 | 0.089240 | 0.001835 | 0.282399 | 0.000032 | 730 | 2.0 | 0.6 | 1233 | 45 | 10 | |
| 11 | 0.067635 | 0.001644 | 0.281993 | 0.000033 | 762 | -11.6 | 0.6 | 1803 | 47 | 11 | |
| 12 | 0.082905 | 0.002607 | 0.282081 | 0.000042 | 759 | -9.0 | 0.8 | 1723 | 62 | 12 | |
| 13 | 0.019485 | 0.000436 | 0.281304 | 0.000029 | 1980 | -8.4 | 0.5 | 2681 | 38 | 13 | |
| 14 | 0.080252 | 0.002340 | 0.282074 | 0.000027 | 806 | -8.2 | 0.5 | 1720 | 39 | 14 | |
| 15 | 0.031216 | 0.000806 | 0.282510 | 0.000029 | 522 | 1.9 | 0.5 | 1045 | 41 | 15 | |
| 16 | 0.087294 | 0.002273 | 0.281978 | 0.000034 | 761 | -12.5 | 0.6 | 1855 | 48 | 16 | |
| 17 | 0.027368 | 0.000837 | 0.282538 | 0.000029 | 398 | 0.3 | 0.5 | 1007 | 41 | 17 | |
| 18 | 0.102928 | 0.002760 | 0.282045 | 0.000034 | 762 | -10.3 | 0.6 | 1783 | 49 | 18 | |
| 19 | 0.147714 | 0.002676 | 0.281979 | 0.000032 | 782 | -12.2 | 0.6 | 1875 | 46 | 19 | |
| 20 | 0.024861 | 0.000518 | 0.282514 | 0.000030 | 794 | 8.1 | 0.5 | 1031 | 42 | 20 | |
| 06SW21a |  |  |  |  |  |  |  |  |  |  | |
| 1 | 0.026235 | 0.000624 | 0.282623 | 0.000023 | 441 | 4.2 | 0.4 | 883 | 32 | 2.1 | |
| 2 | 0.025104 | 0.000449 | 0.281117 | 0.000022 | 1988 | -14.8 | 0.4 | 2932 | 29 | 1.1 | |
| 3 | 0.108004 | 0.003171 | 0.282645 | 0.000097 | 250 | 0.5 | 1.7 | 914 | 145 | 5.1 | |
| 4 | 0.048858 | 0.001130 | 0.282585 | 0.000029 | 461 | 3.2 | 0.5 | 949 | 41 | 4.1 | |
| 6 | 0.052597 | 0.000859 | 0.281181 | 0.000025 | 2009 | -12.7 | 0.5 | 2877 | 34 | 3.1 | |
| 7 | 0.035705 | 0.000703 | 0.281200 | 0.000026 | 2010 | -11.8 | 0.5 | 2840 | 35 | 6.1 | |
| 8 | 0.059721 | 0.001054 | 0.281117 | 0.000021 | 2092 | -13.4 | 0.4 | 2978 | 28 | 6.2 | |
| 10 | 0.054369 | 0.001335 | 0.282568 | 0.000031 | 456 | 2.4 | 0.6 | 978 | 44 | 10.1 | |
| 12 | 0.067896 | 0.002066 | 0.282575 | 0.000034 | 460 | 2.5 | 0.6 | 986 | 49 | 9.1 | |
| 06SW21b |  |  |  |  |  |  |  |  |  |  | |
| 2 | 0.017428 | 0.000340 | 0.281502 | 0.000023 | 1797 | -5.3 | 0.4 | 2407 | 31 | 1.1 | |
| 4 | 0.130391 | 0.003320 | 0.282396 | 0.000039 | 436 | -4.7 | 0.7 | 1290 | 59 | 6.1 | |
| 6 | 0.046044 | 0.001206 | 0.281353 | 0.000045 | 2520 | 4.3 | 0.8 | 2667 | 61 | 2.1 | |
| 7 | 0.203133 | 0.004912 | 0.281520 | 0.000039 | 1841 | -9.4 | 0.7 | 2700 | 59 | 5.1 | |
| 8 | 0.074465 | 0.001477 | 0.281589 | 0.000043 | 1864 | -2.2 | 0.8 | 2360 | 60 | 4.1 | |
| 9 | 0.035315 | 0.000735 | 0.281620 | 0.000042 | 1857 | -0.3 | 0.7 | 2271 | 57 | 4.2 | |
|  |  |  |  |  |  |  |  |  |  |  | |
| Table S3 (continued) | | | | | | | | | | |  |
| Spot | ^176^Yb/^177^Hf | ^176^Lu/^177^Hf | ^176^Hf/^177^Hf | 2σ | U-Pb Age (Ma) | ε_Hf_(t) | 2σ | T_DM_ (Ma) | 2σ | Age Spot^a^ | |
| 10 | 0.079303 | 0.001849 | 0.281651 | 0.000022 | 1901 | 0.4 | 0.4 | 2296 | 32 | 3.1 | |
| 11 | 0.264013 | 0.006724 | 0.281494 | 0.000122 | 1877 | -11.9 | 2.2 | 2893 | 196 | 9.1 | |
| 12 | 0.112545 | 0.002797 | 0.281378 | 0.000076 | 1873 | -11.1 | 1.4 | 2747 | 109 | 7.1 | |
| 13 | 0.071379 | 0.001295 | 0.281652 | 0.000023 | 1904 | 1.2 | 0.4 | 2261 | 32 | 8.1 | |
| 06SW25 |  |  |  |  |  |  |  |  |  |  | |
| 4 | 0.036391 | 0.000719 | 0.282020 | 0.000022 | 777 | -9.9 | 0.4 | 1723 | 30 | 7.1 | |
| 5 | 0.022007 | 0.000444 | 0.281484 | 0.000022 | 1790 | -6.2 | 0.4 | 2438 | 29 | 3.1 | |
| 13 | 0.020882 | 0.000388 | 0.281091 | 0.000019 | 2048 | -14.3 | 0.3 | 2963 | 26 | 9.1 | |
| 06SW28 |  |  |  |  |  |  |  |  |  |  | |
| 1 | 0.094051 | 0.002122 | 0.281394 | 0.000027 | 2258 | -1.5 | 0.5 | 2675 | 38 | 1.1 | |
| 2 | 0.208209 | 0.005497 | 0.281750 | 0.000144 | 1840 | -1.9 | 2.6 | 2390 | 224 | 2.1 | |
| 3 | 0.174138 | 0.004718 | 0.281673 | 0.000067 | 1745 | -5.6 | 1.2 | 2453 | 102 | 3.1 | |
| 4 | 0.183069 | 0.004826 | 0.281751 | 0.000057 | 2122 | 4.4 | 1.0 | 2342 | 88 | 4.1 | |
| 06SW45 |  |  |  |  |  |  |  |  |  |  | |
| 1 | 0.014276 | 0.000315 | 0.281175 | 0.000033 | 2786 | 5.6 | 0.6 | 2844 | 44 | 9.1 | |
| 2 | 0.044899 | 0.001323 | 0.281190 | 0.000035 | 2644 | 1.0 | 0.6 | 2899 | 47 | 10.1 | |
| 7 | 0.046759 | 0.001485 | 0.281150 | 0.000040 | 2774 | 2.2 | 0.7 | 2966 | 54 | 7.1 | |
| 8 | 0.057347 | 0.001731 | 0.281450 | 0.000053 | 2146 | -1.3 | 1.0 | 2570 | 74 | 8.1 | |
| 11 | 0.062166 | 0.001855 | 0.281363 | 0.000042 | 2167 | -4.2 | 0.8 | 2700 | 58 | 6.1 | |
| 12 | 0.023355 | 0.000868 | 0.282592 | 0.000037 | 455 | 3.4 | 0.7 | 932 | 52 | 2.1 | |
| 13 | 0.045786 | 0.001622 | 0.281089 | 0.000053 | 2701 | -1.8 | 0.9 | 3061 | 73 | 3.1 | |
| 15 | 0.030826 | 0.000967 | 0.281199 | 0.000036 | 2648 | 2.1 | 0.6 | 2861 | 49 | 1.1 | |
| 06SW48 |  |  |  |  |  |  |  |  |  |  | |
| 1 | 0.067588 | 0.001719 | 0.282155 | 0.000030 | 303 | -15.5 | 0.5 | 1577 | 43 | 1.1 | |
| 2 | 0.057995 | 0.001378 | 0.281374 | 0.000039 | 2115 | -4.2 | 0.7 | 2651 | 53 | 2.1 | |
| 3 | 0.039999 | 0.001206 | 0.281315 | 0.000045 | 2173 | -4.8 | 0.8 | 2719 | 61 | 3.1 | |
| 6 | 0.112062 | 0.003416 | 0.281380 | 0.000044 | 1914 | -11.0 | 0.8 | 2792 | 64 | 4.1 | |
| 7 | 0.046443 | 0.001149 | 0.281279 | 0.000033 | 2064 | -8.4 | 0.6 | 2765 | 45 | 5.1 | |
| 8 | 0.049191 | 0.000981 | 0.281280 | 0.000031 | 2085 | -7.6 | 0.6 | 2751 | 42 | 10.1 | |
| 11 | 0.031322 | 0.000939 | 0.281366 | 0.000031 | 2087 | -4.4 | 0.6 | 2631 | 42 | 7.1 | |
| 12 | 0.052064 | 0.001064 | 0.281296 | 0.000040 | 2143 | -5.9 | 0.7 | 2736 | 54 | 6.1 | |
| 14 | 0.091558 | 0.002425 | 0.281404 | 0.000030 | 2192 | -3.0 | 0.5 | 2683 | 42 | 8.1 | |
| 20 | 0.033246 | 0.000915 | 0.281349 | 0.000038 | 2105 | -4.6 | 0.7 | 2652 | 52 | 9.1 | |

Note: The initial Hf isotope ratios are calculated at the age that was directly dated by either LA-ICPMS on the same volume or SHRIMP on the same property of spots. ε_Hf_(t) = [((^176^Hf/^177^Hf)_m_ – (^176^Lu/^177^Hf)_m_ × (e^λt^ – 1))/((^176^Hf/^177^Hf)_CHUR t=0_ – (^176^Lu/^177^Hf)_CHUR_ × (e^λt^ – 1)) – 1] × 10000. T_DM_ = 1/λ × [1 + ((^176^Hf/^177^Hf)_S_ – (^176^Hf/^177^Hf)_DM_)/( (^176^Lu/^177^Hf)_S_ – (^176^Lu/^177^Hf)_DM_)]. Abbreviations: S = sample, CHUR = chondritic reservoir, DM = depleted mantle.

^a^suffix “L” and “S” in 05WD15 represent LA-ICPMS and SHRIMP, respectively.

Table S4. LA-ICPMS zircon trace element data for Cenozoic continental basalts from east-central China

| Sample | | 07CL03 | | | | | | | | | | | | | | | | | | | | | | | | | | | | | | | | | | | | | | | | | | | | | | | | | | | | | | | | | | | | | | | | | | | | | | | | | | | | | | | | | | | | | | | | | | | | | | | | | | | | | | | | | | | | | | | | | | | | | | | | | | | | | | | | | | | | | | | | | | | | | |  | | | | |
| --- | --- | --- | --- | --- | --- | --- | --- | --- | --- | --- | --- | --- | --- | --- | --- | --- | --- | --- | --- | --- | --- | --- | --- | --- | --- | --- | --- | --- | --- | --- | --- | --- | --- | --- | --- | --- | --- | --- | --- | --- | --- | --- | --- | --- | --- | --- | --- | --- | --- | --- | --- | --- | --- | --- | --- | --- | --- | --- | --- | --- | --- | --- | --- | --- | --- | --- | --- | --- | --- | --- | --- | --- | --- | --- | --- | --- | --- | --- | --- | --- | --- | --- | --- | --- | --- | --- | --- | --- | --- | --- | --- | --- | --- | --- | --- | --- | --- | --- | --- | --- | --- | --- | --- | --- | --- | --- | --- | --- | --- | --- | --- | --- | --- | --- | --- | --- | --- | --- | --- | --- | --- | --- | --- | --- | --- | --- | --- | --- | --- | --- | --- | --- | --- | --- | --- | --- | --- | --- | --- | --- | --- | --- | --- | --- | --- | --- | --- | --- |
| Spot | | 1 | | | | | | 2 | | | | | | | 3 | | | | | | | | | 4 | | | | | | | | | 5 | | | | | | | | | 6 | | | | | | | | | 7 | | | | | | | | 8 | | | | | | | 9 | | | | | | | | | 10 | | | | | | | | | 11 | | | | | | | 12 | | | | | | | | | 13 | | | | | | | | | 14 | | | | | | | | 15 | | | | | | | | | 16 | | | | | | | | | 17 | | | | | | | | |  | | | | |
| La | | 0.07 | | | | | | 2.89 | | | | | | | 6.95 | | | | | | | | | 0.20 | | | | | | | | | 4.83 | | | | | | | | | 2.24 | | | | | | | | | 7.65 | | | | | | | | 0.16 | | | | | | | 0.38 | | | | | | | | | 0.34 | | | | | | | | | 1.27 | | | | | | | 14.35 | | | | | | | | | 2.68 | | | | | | | | | 0.44 | | | | | | | | 10.45 | | | | | | | | | 0.79 | | | | | | | | | 0.80 | | | | | | | | |  | | | | |
| Ce | | 10.32 | | | | | | 15.73 | | | | | | | 21.52 | | | | | | | | | 4.77 | | | | | | | | | 16.52 | | | | | | | | | 16.95 | | | | | | | | | 27.00 | | | | | | | | 10.96 | | | | | | | 11.60 | | | | | | | | | 6.67 | | | | | | | | | 11.54 | | | | | | | 40.54 | | | | | | | | | 21.27 | | | | | | | | | 14.38 | | | | | | | | 38.46 | | | | | | | | | 10.50 | | | | | | | | | 14.21 | | | | | | | | |  | | | | |
| Pr | | 0.12 | | | | | | 0.83 | | | | | | | 1.75 | | | | | | | | | 0.07 | | | | | | | | | 1.50 | | | | | | | | | 1.45 | | | | | | | | | 2.19 | | | | | | | | 0.07 | | | | | | | 0.19 | | | | | | | | | 0.15 | | | | | | | | | 0.80 | | | | | | | 3.69 | | | | | | | | | 1.20 | | | | | | | | | 0.21 | | | | | | | | 3.93 | | | | | | | | | 0.52 | | | | | | | | | 0.61 | | | | | | | | |  | | | | |
| Nd | | 2.18 | | | | | | 4.20 | | | | | | | 8.16 | | | | | | | | | 0.73 | | | | | | | | | 6.95 | | | | | | | | | 8.36 | | | | | | | | | 10.12 | | | | | | | | 1.09 | | | | | | | 2.33 | | | | | | | | | 1.26 | | | | | | | | | 5.29 | | | | | | | 15.96 | | | | | | | | | 9.49 | | | | | | | | | 1.86 | | | | | | | | 18.19 | | | | | | | | | 3.15 | | | | | | | | | 5.67 | | | | | | | | |  | | | | |
| Sm | | 3.64 | | | | | | 2.29 | | | | | | | 2.19 | | | | | | | | | 0.84 | | | | | | | | | 2.33 | | | | | | | | | 3.08 | | | | | | | | | 3.48 | | | | | | | | 1.90 | | | | | | | 3.29 | | | | | | | | | 1.57 | | | | | | | | | 2.18 | | | | | | | 4.37 | | | | | | | | | 7.75 | | | | | | | | | 2.79 | | | | | | | | 5.23 | | | | | | | | | 1.96 | | | | | | | | | 5.66 | | | | | | | | |  | | | | |
| Eu | | 2.07 | | | | | | 1.00 | | | | | | | 1.16 | | | | | | | | | 0.66 | | | | | | | | | 1.21 | | | | | | | | | 1.35 | | | | | | | | | 1.37 | | | | | | | | 1.03 | | | | | | | 1.79 | | | | | | | | | 1.13 | | | | | | | | | 1.29 | | | | | | | 1.73 | | | | | | | | | 4.45 | | | | | | | | | 1.51 | | | | | | | | 2.23 | | | | | | | | | 1.06 | | | | | | | | | 3.14 | | | | | | | | |  | | | | |
| Gd | | 13.3 | | | | | | 8.4 | | | | | | | 6.6 | | | | | | | | | 4.8 | | | | | | | | | 6.4 | | | | | | | | | 8.5 | | | | | | | | | 9.7 | | | | | | | | 8.5 | | | | | | | 12.4 | | | | | | | | | 6.5 | | | | | | | | | 6.8 | | | | | | | 11.0 | | | | | | | | | 22.2 | | | | | | | | | 11.7 | | | | | | | | 13.6 | | | | | | | | | 7.4 | | | | | | | | | 17.4 | | | | | | | | |  | | | | |
| Tb | | 3.78 | | | | | | 2.57 | | | | | | | 2.39 | | | | | | | | | 1.335 | | | | | | | | | 1.82 | | | | | | | | | 2.43 | | | | | | | | | 3.06 | | | | | | | | 2.86 | | | | | | | 3.73 | | | | | | | | | 2.14 | | | | | | | | | 2.11 | | | | | | | 3.31 | | | | | | | | | 5.74 | | | | | | | | | 3.74 | | | | | | | | 3.68 | | | | | | | | | 2.25 | | | | | | | | | 4.64 | | | | | | | | |  | | | | |
| Dy | | 40.2 | | | | | | 30.2 | | | | | | | 24.8 | | | | | | | | | 17.2 | | | | | | | | | 21.7 | | | | | | | | | 28.6 | | | | | | | | | 33.1 | | | | | | | | 33.3 | | | | | | | 39.7 | | | | | | | | | 26.9 | | | | | | | | | 24.7 | | | | | | | 36.8 | | | | | | | | | 54.7 | | | | | | | | | 42.4 | | | | | | | | 41.5 | | | | | | | | | 25.3 | | | | | | | | | 45.9 | | | | | | | | |  | | | | |
| Ho | | 13.9 | | | | | | 11.4 | | | | | | | 9.8 | | | | | | | | | 6.9 | | | | | | | | | 8.2 | | | | | | | | | 11.1 | | | | | | | | | 12.7 | | | | | | | | 12.8 | | | | | | | 14.2 | | | | | | | | | 10.5 | | | | | | | | | 10.0 | | | | | | | 13.7 | | | | | | | | | 17.8 | | | | | | | | | 16.3 | | | | | | | | 14.3 | | | | | | | | | 9.5 | | | | | | | | | 15.3 | | | | | | | | |  | | | | |
| Er | | 59.3 | | | | | | 53.8 | | | | | | | 46.2 | | | | | | | | | 32.9 | | | | | | | | | 38.6 | | | | | | | | | 52.2 | | | | | | | | | 58.6 | | | | | | | | 59.2 | | | | | | | 62.0 | | | | | | | | | 49.4 | | | | | | | | | 47.5 | | | | | | | 61.9 | | | | | | | | | 73.8 | | | | | | | | | 73.8 | | | | | | | | 63.3 | | | | | | | | | 43.3 | | | | | | | | | 62.6 | | | | | | | | |  | | | | |
| Tm | | 13.1 | | | | | | 12.2 | | | | | | | 11.0 | | | | | | | | | 7.8 | | | | | | | | | 9.3 | | | | | | | | | 12.3 | | | | | | | | | 13.2 | | | | | | | | 13.5 | | | | | | | 13.6 | | | | | | | | | 11.6 | | | | | | | | | 11.3 | | | | | | | 14.2 | | | | | | | | | 15.2 | | | | | | | | | 16.6 | | | | | | | | 14.0 | | | | | | | | | 9.7 | | | | | | | | | 13.6 | | | | | | | | |  | | | | |
| Yb | | 134 | | | | | | 131 | | | | | | | 125 | | | | | | | | | 89 | | | | | | | | | 102 | | | | | | | | | 133 | | | | | | | | | 145 | | | | | | | | 145 | | | | | | | 141 | | | | | | | | | 131 | | | | | | | | | 130 | | | | | | | 157 | | | | | | | | | 158 | | | | | | | | | 179 | | | | | | | | 152 | | | | | | | | | 109 | | | | | | | | | 143 | | | | | | | | |  | | | | |
| Lu | | 28 | | | | | | 28 | | | | | | | 27 | | | | | | | | | 19 | | | | | | | | | 22 | | | | | | | | | 29 | | | | | | | | | 30 | | | | | | | | 31 | | | | | | | 28 | | | | | | | | | 28 | | | | | | | | | 27 | | | | | | | 32 | | | | | | | | | 30 | | | | | | | | | 37 | | | | | | | | 32 | | | | | | | | | 22 | | | | | | | | | 27 | | | | | | | | |  | | | | |
| Pb | | 24 | | | | | | 32 | | | | | | | 25 | | | | | | | | | 16 | | | | | | | | | 20 | | | | | | | | | 28 | | | | | | | | | 31 | | | | | | | | 32 | | | | | | | 32 | | | | | | | | | 22 | | | | | | | | | 27 | | | | | | | 37 | | | | | | | | | 25 | | | | | | | | | 39 | | | | | | | | 30 | | | | | | | | | 21 | | | | | | | | | 22 | | | | | | | | |  | | | | |
| Th | | 34 | | | | | | 29 | | | | | | | 31 | | | | | | | | | 11 | | | | | | | | | 16 | | | | | | | | | 23 | | | | | | | | | 31 | | | | | | | | 32 | | | | | | | 35 | | | | | | | | | 20 | | | | | | | | | 24 | | | | | | | 47 | | | | | | | | | 36 | | | | | | | | | 47 | | | | | | | | 37 | | | | | | | | | 21 | | | | | | | | | 30 | | | | | | | | |  | | | | |
| U | | 37 | | | | | | 50 | | | | | | | 39 | | | | | | | | | 24 | | | | | | | | | 31 | | | | | | | | | 43 | | | | | | | | | 50 | | | | | | | | 50 | | | | | | | 1 | | | | | | | | | 36 | | | | | | | | | 44 | | | | | | | 60 | | | | | | | | | 36 | | | | | | | | | 63 | | | | | | | | 50 | | | | | | | | | 37 | | | | | | | | | 35 | | | | | | | | |  | | | | |
| Y | | 423 | | | | | | 360 | | | | | | | 308 | | | | | | | | | 220 | | | | | | | | | 256 | | | | | | | | | 349 | | | | | | | | | 398 | | | | | | | | 398 | | | | | | | 439 | | | | | | | | | 329 | | | | | | | | | 318 | | | | | | | 427 | | | | | | | | | 540 | | | | | | | | | 507 | | | | | | | | 450 | | | | | | | | | 293 | | | | | | | | | 459 | | | | | | | | |  | | | | |
| Hf | | 8549 | | | | | | 8702 | | | | | | | 7438 | | | | | | | | | 7745 | | | | | | | | | 7382 | | | | | | | | | 7797 | | | | | | | | | 8425 | | | | | | | | 8466 | | | | | | | 7931 | | | | | | | | | 6949 | | | | | | | | | 7138 | | | | | | | 8253 | | | | | | | | | 6729 | | | | | | | | | 8750 | | | | | | | | 8728 | | | | | | | | | 8223 | | | | | | | | | 7083 | | | | | | | | |  | | | | |
| Nb | | 0.33 | | | | | | 0.44 | | | | | | | 0.30 | | | | | | | | | 0.36 | | | | | | | | | 0.29 | | | | | | | | | 0.38 | | | | | | | | | 0.42 | | | | | | | | 0.55 | | | | | | | 0.59 | | | | | | | | | 0.40 | | | | | | | | | 0.54 | | | | | | | 0.56 | | | | | | | | | 0.56 | | | | | | | | | 0.56 | | | | | | | | 0.36 | | | | | | | | | 0.32 | | | | | | | | | 0.46 | | | | | | | | |  | | | | |
| Ta | | 0.11 | | | | | | 0.15 | | | | | | | 0.12 | | | | | | | | | 0.10 | | | | | | | | | 0.10 | | | | | | | | | 0.11 | | | | | | | | | 0.16 | | | | | | | | 0.16 | | | | | | | 0.14 | | | | | | | | | 0.13 | | | | | | | | | 0.14 | | | | | | | 0.18 | | | | | | | | | 0.11 | | | | | | | | | 0.20 | | | | | | | | 0.12 | | | | | | | | | 0.14 | | | | | | | | | 0.11 | | | | | | | | |  | | | | |
| P | |  | | | | | |  | | | | | | |  | | | | | | | | |  | | | | | | | | |  | | | | | | | | |  | | | | | | | | |  | | | | | | | |  | | | | | | |  | | | | | | | | |  | | | | | | | | |  | | | | | | |  | | | | | | | | |  | | | | | | | | |  | | | | | | | |  | | | | | | | | |  | | | | | | | | |  | | | | | | | | |  | | | | |
| LREE | | 13 | | | | | | 24 | | | | | | | 38 | | | | | | | | | 6 | | | | | | | | | 30 | | | | | | | | | 29 | | | | | | | | | 47 | | | | | | | | 12 | | | | | | | 14 | | | | | | | | | 8 | | | | | | | | | 19 | | | | | | | 75 | | | | | | | | | 35 | | | | | | | | | 17 | | | | | | | | 71 | | | | | | | | | 15 | | | | | | | | | 21 | | | | | | | | |  | | | | |
| MREE | | 63 | | | | | | 44 | | | | | | | 37 | | | | | | | | | 25 | | | | | | | | | 33 | | | | | | | | | 44 | | | | | | | | | 51 | | | | | | | | 48 | | | | | | | 61 | | | | | | | | | 38 | | | | | | | | | 37 | | | | | | | 57 | | | | | | | | | 95 | | | | | | | | | 62 | | | | | | | | 66 | | | | | | | | | 38 | | | | | | | | | 77 | | | | | | | | |  | | | | |
| HREE | | 248 | | | | | | 236 | | | | | | | 219 | | | | | | | | | 156 | | | | | | | | | 181 | | | | | | | | | 237 | | | | | | | | | 260 | | | | | | | | 261 | | | | | | | 259 | | | | | | | | | 230 | | | | | | | | | 226 | | | | | | | 279 | | | | | | | | | 294 | | | | | | | | | 323 | | | | | | | | 275 | | | | | | | | | 194 | | | | | | | | | 262 | | | | | | | | |  | | | | |
| (Yb/La)_n_ | | 2751.3 | | | | | | 63.2 | | | | | | | 25.1 | | | | | | | | | 623.2 | | | | | | | | | 29.5 | | | | | | | | | 82.8 | | | | | | | | | 26.5 | | | | | | | | 1229.7 | | | | | | | 515.2 | | | | | | | | | 533.8 | | | | | | | | | 142.7 | | | | | | | 15.3 | | | | | | | | | 82.1 | | | | | | | | | 573.3 | | | | | | | | 20.2 | | | | | | | | | 193.1 | | | | | | | | | 248.7 | | | | | | | | |  | | | | |
| (Sm/La)_n_ | | 82.9 | | | | | | 1.2 | | | | | | | 0.5 | | | | | | | | | 6.5 | | | | | | | | | 0.7 | | | | | | | | | 2.1 | | | | | | | | | 0.7 | | | | | | | | 17.9 | | | | | | | 13.3 | | | | | | | | | 7.1 | | | | | | | | | 2.7 | | | | | | | 0.5 | | | | | | | | | 4.5 | | | | | | | | | 9.9 | | | | | | | | 0.8 | | | | | | | | | 3.9 | | | | | | | | | 10.9 | | | | | | | | |  | | | | |
| (Lu/Gd)_n_ | | 16.9 | | | | | | 26.7 | | | | | | | 32.7 | | | | | | | | | 32.9 | | | | | | | | | 28.5 | | | | | | | | | 27.4 | | | | | | | | | 25.0 | | | | | | | | 29.0 | | | | | | | 18.6 | | | | | | | | | 34.4 | | | | | | | | | 32.2 | | | | | | | 23.8 | | | | | | | | | 10.9 | | | | | | | | | 25.6 | | | | | | | | 19.1 | | | | | | | | | 24.6 | | | | | | | | | 12.7 | | | | | | | | |  | | | | |
| Eu/Eu* | | 0.81 | | | | | | 0.62 | | | | | | | 0.86 | | | | | | | | | 0.79 | | | | | | | | | 0.90 | | | | | | | | | 0.76 | | | | | | | | | 0.67 | | | | | | | | 0.66 | | | | | | | 0.76 | | | | | | | | | 0.93 | | | | | | | | | 0.94 | | | | | | | 0.73 | | | | | | | | | 0.97 | | | | | | | | | 0.69 | | | | | | | | 0.77 | | | | | | | | | 0.75 | | | | | | | | | 0.89 | | | | | | | | |  | | | | |
| Ce/Ce* | | 21.61 | | | | | | 2.46 | | | | | | | 1.47 | | | | | | | | | 10.16 | | | | | | | | | 1.49 | | | | | | | | | 2.24 | | | | | | | | | 1.59 | | | | | | | | 24.53 | | | | | | | 10.59 | | | | | | | | | 7.33 | | | | | | | | | 2.73 | | | | | | | 1.33 | | | | | | | | | 2.90 | | | | | | | | | 11.54 | | | | | | | | 1.47 | | | | | | | | | 3.91 | | | | | | | | | 4.75 | | | | | | | | |  | | | | |
| U/Yb | | 0.278 | | | | | | 0.384 | | | | | | | 0.312 | | | | | | | | | 0.274 | | | | | | | | | 0.303 | | | | | | | | | 0.326 | | | | | | | | | 0.343 | | | | | | | | 0.347 | | | | | | | 0.009 | | | | | | | | | 0.275 | | | | | | | | | 0.340 | | | | | | | 0.383 | | | | | | | | | 0.227 | | | | | | | | | 0.354 | | | | | | | | 0.327 | | | | | | | | | 0.339 | | | | | | | | | 0.241 | | | | | | | | |  | | | | |
| Nb/Yb | | 0.002 | | | | | | 0.003 | | | | | | | 0.002 | | | | | | | | | 0.004 | | | | | | | | | 0.003 | | | | | | | | | 0.003 | | | | | | | | | 0.003 | | | | | | | | 0.004 | | | | | | | 0.004 | | | | | | | | | 0.003 | | | | | | | | | 0.004 | | | | | | | 0.004 | | | | | | | | | 0.004 | | | | | | | | | 0.003 | | | | | | | | 0.002 | | | | | | | | | 0.003 | | | | | | | | | 0.003 | | | | | | | | |  | | | | |
|  | |  | | | | | |  | | | | | | |  | | | | | | | | |  | | | | | | | | |  | | | | | | | | |  | | | | | | | | |  | | | | | | | |  | | | | | | |  | | | | | | | | |  | | | | | | | | |  | | | | | | |  | | | | | | | | |  | | | | | | | | |  | | | | | | | |  | | | | | | | | |  | | | | | | | | |  | | | | | | | | |  | | | | |
| Table S4 (continued) | | | | | | | | | | | | | | | | | | | | | | | | | | | | | | | | | | | | | | | | | | | | | | | | | | | | | | | | | | | | | | | | | | | | | | | | | | | | | | | | | | | | | | | | | | | | | | | | | | | | | | | | | | | | | | | | | | | | | | | | | | | | | | | | | | | | | | | | | | | | | | | | | |  | | |
| Sample | | 07CL03 | | | | | | | | | | | | | | | | | | | | |  | | | | | | | | 07CL05 | | | | | | | | | | | | | | | | | | | | | | | | | | | | | | | | | | | | | | | | | | | | | | | | | | | | | | | | | | | | | | | | | | | | | | | | | | | | | | | | | | | | | | | | | | | | | | | | | | | | | | | | | | | | | | | | | | |  | | |
| Spot | | 18 | | | | 19 | | | | | | | | 20 | | | | | | | | |  | | | | | | | | 1 | | | | | | | | | 2 | | | | | | | | 3 | | | | | | | | | 4 | | | | | | | 5 | | | | | | | | 6 | | | | | | | | | 7 | | | | | | | 8 | | | | | | | | | 9 | | | | | | | | | 10 | | | | | | | | 11 | | | | | | | 12 | | | | | | | | 13 | | | | | | | | 14 | | | | | | | | |  | | |
| La | | 0.46 | | | | 0.53 | | | | | | | | 3.04 | | | | | | | | |  | | | | | | | | 8.37 | | | | | | | | | 0.14 | | | | | | | | 1.00 | | | | | | | | | 0.04 | | | | | | | 22.65 | | | | | | | | 10.14 | | | | | | | | | 93.07 | | | | | | | 5.70 | | | | | | | | | 0.26 | | | | | | | | | 9.04 | | | | | | | | 0.18 | | | | | | | 0.56 | | | | | | | | 75.81 | | | | | | | | 0.21 | | | | | | | | |  | | |
| Ce | | 8.60 | | | | 11.58 | | | | | | | | 11.38 | | | | | | | | |  | | | | | | | | 64.97 | | | | | | | | | 13.27 | | | | | | | | 5.14 | | | | | | | | | 5.13 | | | | | | | 94.38 | | | | | | | | 52.34 | | | | | | | | | 234.54 | | | | | | | 127.04 | | | | | | | | | 5.13 | | | | | | | | | 27.14 | | | | | | | | 13.92 | | | | | | | 20.07 | | | | | | | | 161.52 | | | | | | | | 11.28 | | | | | | | | |  | | |
| Pr | | 0.22 | | | | 0.37 | | | | | | | | 0.83 | | | | | | | | |  | | | | | | | | 3.07 | | | | | | | | | 0.29 | | | | | | | | 0.44 | | | | | | | | | 0.03 | | | | | | | 6.74 | | | | | | | | 3.77 | | | | | | | | | 26.52 | | | | | | | 7.45 | | | | | | | | | 0.19 | | | | | | | | | 3.79 | | | | | | | | 0.28 | | | | | | | 1.06 | | | | | | | | 16.55 | | | | | | | | 0.18 | | | | | | | | |  | | |
| Nd | | 1.45 | | | | 3.04 | | | | | | | | 3.97 | | | | | | | | |  | | | | | | | | 17.66 | | | | | | | | | 3.34 | | | | | | | | 2.94 | | | | | | | | | 0.77 | | | | | | | 31.16 | | | | | | | | 18.02 | | | | | | | | | 116.32 | | | | | | | 50.18 | | | | | | | | | 1.53 | | | | | | | | | 19.82 | | | | | | | | 3.68 | | | | | | | 11.75 | | | | | | | | 69.89 | | | | | | | | 2.65 | | | | | | | | |  | | |
| Sm | | 1.56 | | | | 2.91 | | | | | | | | 1.53 | | | | | | | | |  | | | | | | | | 11.96 | | | | | | | | | 4.39 | | | | | | | | 2.95 | | | | | | | | | 1.18 | | | | | | | 8.61 | | | | | | | | 6.44 | | | | | | | | | 26.71 | | | | | | | 33.58 | | | | | | | | | 3.34 | | | | | | | | | 10.35 | | | | | | | | 5.38 | | | | | | | 15.50 | | | | | | | | 14.53 | | | | | | | | 4.08 | | | | | | | | |  | | |
| Eu | | 0.89 | | | | 1.40 | | | | | | | | 0.92 | | | | | | | | |  | | | | | | | | 1.25 | | | | | | | | | 0.92 | | | | | | | | 0.57 | | | | | | | | | 0.73 | | | | | | | 1.13 | | | | | | | | 1.69 | | | | | | | | | 1.11 | | | | | | | 4.19 | | | | | | | | | 0.64 | | | | | | | | | 2.09 | | | | | | | | 1.13 | | | | | | | 2.67 | | | | | | | | 1.46 | | | | | | | | 0.59 | | | | | | | | |  | | |
| Gd | | 6.9 | | | | 10.3 | | | | | | | | 5.4 | | | | | | | | |  | | | | | | | | 50.9 | | | | | | | | | 16.1 | | | | | | | | 14.3 | | | | | | | | | 4.8 | | | | | | | 13.9 | | | | | | | | 15.9 | | | | | | | | | 65.4 | | | | | | | 87.6 | | | | | | | | | 14.1 | | | | | | | | | 18.2 | | | | | | | | 26.2 | | | | | | | 61.5 | | | | | | | | 19.9 | | | | | | | | 13.9 | | | | | | | | |  | | |
| Tb | | 2.17 | | | | 3.15 | | | | | | | | 1.676 | | | | | | | | |  | | | | | | | | 17.86 | | | | | | | | | 4.52 | | | | | | | | 4.12 | | | | | | | | | 1.75 | | | | | | | 3.33 | | | | | | | | 5.21 | | | | | | | | | 20.23 | | | | | | | 30.02 | | | | | | | | | 4.3 | | | | | | | | | 4.88 | | | | | | | | 8.38 | | | | | | | 20.04 | | | | | | | | 5.37 | | | | | | | | 3.8 | | | | | | | | |  | | |
| Dy | | 24.9 | | | | 35.6 | | | | | | | | 19.0 | | | | | | | | |  | | | | | | | | 219.2 | | | | | | | | | 46.6 | | | | | | | | 31.3 | | | | | | | | | 20.1 | | | | | | | 35.0 | | | | | | | | 58.9 | | | | | | | | | 233.7 | | | | | | | 351.8 | | | | | | | | | 39.6 | | | | | | | | | 30.3 | | | | | | | | 91.7 | | | | | | | 227.1 | | | | | | | | 56.1 | | | | | | | | 40.3 | | | | | | | | |  | | |
| Ho | | 9.4 | | | | 13.2 | | | | | | | | 7.5 | | | | | | | | |  | | | | | | | | 84.4 | | | | | | | | | 15.5 | | | | | | | | 7.2 | | | | | | | | | 8.0 | | | | | | | 12.7 | | | | | | | | 22.3 | | | | | | | | | 85.8 | | | | | | | 121.7 | | | | | | | | | 14.1 | | | | | | | | | 5.3 | | | | | | | | 34.0 | | | | | | | 76.9 | | | | | | | | 21.0 | | | | | | | | 13.8 | | | | | | | | |  | | |
| Er | | 43.6 | | | | 60.2 | | | | | | | | 35.9 | | | | | | | | |  | | | | | | | | 389.1 | | | | | | | | | 68.3 | | | | | | | | 24.3 | | | | | | | | | 38.5 | | | | | | | 59.6 | | | | | | | | 101.7 | | | | | | | | | 366.8 | | | | | | | 508.8 | | | | | | | | | 61.1 | | | | | | | | | 15.9 | | | | | | | | 143.5 | | | | | | | 308.2 | | | | | | | | 105.0 | | | | | | | | 60.3 | | | | | | | | |  | | |
| Tm | | 10.2 | | | | 13.7 | | | | | | | | 8.7 | | | | | | | | |  | | | | | | | | 85.4 | | | | | | | | | 15.3 | | | | | | | | 4.3 | | | | | | | | | 9.0 | | | | | | | 14.2 | | | | | | | | 23.4 | | | | | | | | | 74.1 | | | | | | | 104.8 | | | | | | | | | 13.4 | | | | | | | | | 3.2 | | | | | | | | 30.2 | | | | | | | 58.6 | | | | | | | | 25.2 | | | | | | | | 13.2 | | | | | | | | |  | | |
| Yb | | 115 | | | | 148 | | | | | | | | 100 | | | | | | | | |  | | | | | | | | 855 | | | | | | | | | 160 | | | | | | | | 40 | | | | | | | | | 100 | | | | | | | 153 | | | | | | | | 248 | | | | | | | | | 695 | | | | | | | 970 | | | | | | | | | 139 | | | | | | | | | 30 | | | | | | | | 306 | | | | | | | 521 | | | | | | | | 295 | | | | | | | | 133 | | | | | | | | |  | | |
| Lu | | 24 | | | | 31 | | | | | | | | 21 | | | | | | | | |  | | | | | | | | 165 | | | | | | | | | 30 | | | | | | | | 7 | | | | | | | | | 22 | | | | | | | 31 | | | | | | | | 50 | | | | | | | | | 121 | | | | | | | 166 | | | | | | | | | 25 | | | | | | | | | 6 | | | | | | | | 53 | | | | | | | 88 | | | | | | | | 59 | | | | | | | | 25 | | | | | | | | |  | | |
| Pb | | 27 | | | | 28 | | | | | | | | 17 | | | | | | | | |  | | | | | | | | 50 | | | | | | | | | 531 | | | | | | | | 621 | | | | | | | | | 21 | | | | | | | 768 | | | | | | | | 807 | | | | | | | | | 1077 | | | | | | | 250 | | | | | | | | | 442 | | | | | | | | | 398 | | | | | | | | 824 | | | | | | | 178 | | | | | | | | 974 | | | | | | | | 1057 | | | | | | | | |  | | |
| Th | | 27 | | | | 31 | | | | | | | | 14 | | | | | | | | |  | | | | | | | | 1234 | | | | | | | | | 107 | | | | | | | | 24 | | | | | | | | | 15 | | | | | | | 234 | | | | | | | | 173 | | | | | | | | | 355 | | | | | | | 758 | | | | | | | | | 63 | | | | | | | | | 63 | | | | | | | | 217 | | | | | | | 110 | | | | | | | | 318 | | | | | | | | 301 | | | | | | | | |  | | |
| U | | 47 | | | | 47 | | | | | | | | 28 | | | | | | | | |  | | | | | | | | 1079 | | | | | | | | | 272 | | | | | | | | 464 | | | | | | | | | 29 | | | | | | | 333 | | | | | | | | 407 | | | | | | | | | 534 | | | | | | | 942 | | | | | | | | | 264 | | | | | | | | | 290 | | | | | | | | 462 | | | | | | | 263 | | | | | | | | 514 | | | | | | | | 597 | | | | | | | | |  | | |
| Y | | 298 | | | | 416 | | | | | | | | 240 | | | | | | | | |  | | | | | | | | 2557 | | | | | | | | | 485 | | | | | | | | 216 | | | | | | | | | 252 | | | | | | | 420 | | | | | | | | 709 | | | | | | | | | 2389 | | | | | | | 3465 | | | | | | | | | 418 | | | | | | | | | 159 | | | | | | | | 995 | | | | | | | 2108 | | | | | | | | 669 | | | | | | | | 427 | | | | | | | | |  | | |
| Hf | | 7899 | | | | 8282 | | | | | | | | 7032 | | | | | | | | |  | | | | | | | | 9925 | | | | | | | | | 8601 | | | | | | | | 11163 | | | | | | | | | 7468 | | | | | | | 8994 | | | | | | | | 9329 | | | | | | | | | 7881 | | | | | | | 9132 | | | | | | | | | 9505 | | | | | | | | | 10802 | | | | | | | | 9500 | | | | | | | 8307 | | | | | | | | 9017 | | | | | | | | 9032 | | | | | | | | |  | | |
| Nb | | 0.42 | | | | 0.42 | | | | | | | | 0.28 | | | | | | | | |  | | | | | | | | 10.15 | | | | | | | | | 0.96 | | | | | | | | 0.85 | | | | | | | | | 0.31 | | | | | | | 1.72 | | | | | | | | 1.65 | | | | | | | | | 8.30 | | | | | | | 23.55 | | | | | | | | | 0.57 | | | | | | | | | 0.56 | | | | | | | | 0.80 | | | | | | | 4.51 | | | | | | | | 1.19 | | | | | | | | 0.99 | | | | | | | | |  | | |
| Ta | | 0.11 | | | | 0.12 | | | | | | | | 0.09 | | | | | | | | |  | | | | | | | | 4.33 | | | | | | | | | 0.45 | | | | | | | | 0.47 | | | | | | | | | 0.09 | | | | | | | 0.53 | | | | | | | | 0.34 | | | | | | | | | 1.79 | | | | | | | 5.69 | | | | | | | | | 0.24 | | | | | | | | | 0.34 | | | | | | | | 0.51 | | | | | | | 1.61 | | | | | | | | 0.59 | | | | | | | | 0.54 | | | | | | | | |  | | |
| P | |  | | | |  | | | | | | | |  | | | | | | | | |  | | | | | | | |  | | | | | | | | |  | | | | | | | |  | | | | | | | | |  | | | | | | |  | | | | | | | |  | | | | | | | | |  | | | | | | |  | | | | | | | | |  | | | | | | | | |  | | | | | | | |  | | | | | | |  | | | | | | | |  | | | | | | | |  | | | | | | | | |  | | |
| LREE | | 11 | | | | 16 | | | | | | | | 19 | | | | | | | | |  | | | | | | | | 94 | | | | | | | | | 17 | | | | | | | | 10 | | | | | | | | | 6 | | | | | | | 155 | | | | | | | | 84 | | | | | | | | | 470 | | | | | | | 190 | | | | | | | | | 7 | | | | | | | | | 60 | | | | | | | | 18 | | | | | | | 33 | | | | | | | | 324 | | | | | | | | 14 | | | | | | | | |  | | |
| MREE | | 36 | | | | 53 | | | | | | | | 28 | | | | | | | | |  | | | | | | | | 301 | | | | | | | | | 73 | | | | | | | | 53 | | | | | | | | | 28 | | | | | | | 62 | | | | | | | | 88 | | | | | | | | | 347 | | | | | | | 507 | | | | | | | | | 62 | | | | | | | | | 66 | | | | | | | | 133 | | | | | | | 327 | | | | | | | | 97 | | | | | | | | 63 | | | | | | | | |  | | |
| HREE | | 201 | | | | 266 | | | | | | | | 173 | | | | | | | | |  | | | | | | | | 1579 | | | | | | | | | 289 | | | | | | | | 83 | | | | | | | | | 178 | | | | | | | 271 | | | | | | | | 446 | | | | | | | | | 1342 | | | | | | | 1871 | | | | | | | | | 253 | | | | | | | | | 60 | | | | | | | | 567 | | | | | | | 1054 | | | | | | | | 505 | | | | | | | | 245 | | | | | | | | |  | | |
| (Yb/La)_n_ | | 347.8 | | | | 386.5 | | | | | | | | 45.7 | | | | | | | | |  | | | | | | | | 142.4 | | | | | | | | | 1592.3 | | | | | | | | 56.1 | | | | | | | | | 3968.5 | | | | | | | 9.4 | | | | | | | | 34.1 | | | | | | | | | 10.4 | | | | | | | 237.2 | | | | | | | | | 746.7 | | | | | | | | | 4.7 | | | | | | | | 2368.6 | | | | | | | 1298.1 | | | | | | | | 5.4 | | | | | | | | 906.7 | | | | | | | | |  | | |
| (Sm/La)_n_ | | 5.3 | | | | 8.5 | | | | | | | | 0.8 | | | | | | | | |  | | | | | | | | 2.2 | | | | | | | | | 48.6 | | | | | | | | 4.6 | | | | | | | | | 52.2 | | | | | | | 0.6 | | | | | | | | 1.0 | | | | | | | | | 0.4 | | | | | | | 9.1 | | | | | | | | | 19.9 | | | | | | | | | 1.8 | | | | | | | | 46.3 | | | | | | | 42.9 | | | | | | | | 0.3 | | | | | | | | 30.8 | | | | | | | | |  | | |
| (Lu/Gd)_n_ | | 28.1 | | | | 24.3 | | | | | | | | 32.2 | | | | | | | | |  | | | | | | | | 26.2 | | | | | | | | | 15.2 | | | | | | | | 4.0 | | | | | | | | | 38.0 | | | | | | | 18.1 | | | | | | | | 25.4 | | | | | | | | | 14.9 | | | | | | | 15.3 | | | | | | | | | 14.6 | | | | | | | | | 2.5 | | | | | | | | 16.5 | | | | | | | 11.6 | | | | | | | | 24.0 | | | | | | | | 14.3 | | | | | | | | |  | | |
| Eu/Eu* | | 0.71 | | | | 0.70 | | | | | | | | 0.88 | | | | | | | | |  | | | | | | | | 0.13 | | | | | | | | | 0.30 | | | | | | | | 0.22 | | | | | | | | | 0.82 | | | | | | | 0.31 | | | | | | | | 0.49 | | | | | | | | | 0.08 | | | | | | | 0.22 | | | | | | | | | 0.24 | | | | | | | | | 0.46 | | | | | | | | 0.24 | | | | | | | 0.23 | | | | | | | | 0.26 | | | | | | | | 0.22 | | | | | | | | |  | | |
| Ce/Ce* | | 6.69 | | | | 6.20 | | | | | | | | 1.73 | | | | | | | | |  | | | | | | | | 3.14 | | | | | | | | | 12.08 | | | | | | | | 1.89 | | | | | | | | | 37.89 | | | | | | | 1.85 | | | | | | | | 2.07 | | | | | | | | | 1.14 | | | | | | | 4.05 | | | | | | | | | 5.49 | | | | | | | | | 1.14 | | | | | | | | 12.27 | | | | | | | 4.85 | | | | | | | | 1.07 | | | | | | | | 13.31 | | | | | | | | |  | | |
| U/Yb | | 0.410 | | | | 0.317 | | | | | | | | 0.277 | | | | | | | | |  | | | | | | | | 1.262 | | | | | | | | | 1.699 | | | | | | | | 11.533 | | | | | | | | | 0.288 | | | | | | | 2.171 | | | | | | | | 1.639 | | | | | | | | | 0.768 | | | | | | | 0.971 | | | | | | | | | 1.897 | | | | | | | | | 9.552 | | | | | | | | 1.512 | | | | | | | 0.505 | | | | | | | | 1.743 | | | | | | | | 4.478 | | | | | | | | |  | | |
| Nb/Yb | | 0.004 | | | | 0.003 | | | | | | | | 0.003 | | | | | | | | |  | | | | | | | | 0.012 | | | | | | | | | 0.006 | | | | | | | | 0.021 | | | | | | | | | 0.003 | | | | | | | 0.011 | | | | | | | | 0.007 | | | | | | | | | 0.012 | | | | | | | 0.024 | | | | | | | | | 0.004 | | | | | | | | | 0.018 | | | | | | | | 0.003 | | | | | | | 0.009 | | | | | | | | 0.004 | | | | | | | | 0.007 | | | | | | | | |  | | |
|  | |  | | | |  | | | | | | | |  | | | | | | | | |  | | | | | | | |  | | | | | | | | |  | | | | | | | |  | | | | | | | | |  | | | | | | |  | | | | | | | |  | | | | | | | | |  | | | | | | |  | | | | | | | | |  | | | | | | | | |  | | | | | | | |  | | | | | | |  | | | | | | | |  | | | | | | | |  | | | | | | | | | | | |
| Table S4 (continued) | | | | | | | | | | | | | | | | | | | | | | | | | | | | | | | | | | | | | | | | | | | | | | | | | | | | | | | | | | | | | | | | | | | | | | | | | | | | | | | | | | | | | | | | | | | | | | | | | | | | | | | | | | | | | | | | | | | | | | | | | | | | | | | | | | | | | | | | | | | | | | | |  | | | | |
| Sample | | 07CL07 | | | | | | | | | | | | | | | | | | | | | | | | | | | | | | | | | | | | | | | | | | | | | | | | | | | | | | | | | | | | | | | | | | | | | | | | | | | | | | | | | | | | | | | | | | | | | | | | | | | | | | | | | | | | | | | | | | | | | | | | | | | | | | | | | | | | | | | | | | | | | |  | | | | |
| Spot | | 1 | | | | | | 2 | | | | | | | 3 | | | | | | | | | 4 | | | | | | | | | 5 | | | | | | | | | 6 | | | | | | | | | 7 | | | | | | | | 8 | | | | | | | 9 | | | | | | | | | 10 | | | | | | | | | 11 | | | | | | | 12 | | | | | | | | | 13 | | | | | | | | | 14 | | | | | | | | 15 | | | | | | | | | 16 | | | | | | | | | 17 | | | | | | | | |  | | | | |
| La | | 0.22 | | | | | | 1.34 | | | | | | | 0.19 | | | | | | | | | 0.17 | | | | | | | | | 0.17 | | | | | | | | | 0.34 | | | | | | | | | 34.81 | | | | | | | | 0.14 | | | | | | | 0.38 | | | | | | | | | 0.23 | | | | | | | | | 0.17 | | | | | | | 0.22 | | | | | | | | | 0.17 | | | | | | | | | 0.17 | | | | | | | | 0.21 | | | | | | | | | 45.08 | | | | | | | | | 0.15 | | | | | | | | |  | | | | |
| Ce | | 25.81 | | | | | | 22.22 | | | | | | | 20.57 | | | | | | | | | 2.58 | | | | | | | | | 14.80 | | | | | | | | | 71.30 | | | | | | | | | 99.33 | | | | | | | | 1.07 | | | | | | | 17.81 | | | | | | | | | 0.74 | | | | | | | | | 2.32 | | | | | | | 24.40 | | | | | | | | | 17.65 | | | | | | | | | 14.89 | | | | | | | | 14.54 | | | | | | | | | 107.63 | | | | | | | | | 49.86 | | | | | | | | |  | | | | |
| Pr | | 0.19 | | | | | | 0.57 | | | | | | | 0.30 | | | | | | | | | 0.17 | | | | | | | | | 0.17 | | | | | | | | | 0.56 | | | | | | | | | 7.91 | | | | | | | | 0.14 | | | | | | | 0.47 | | | | | | | | | 0.10 | | | | | | | | | 0.14 | | | | | | | 0.54 | | | | | | | | | 0.32 | | | | | | | | | 0.32 | | | | | | | | 1.25 | | | | | | | | | 10.07 | | | | | | | | | 0.53 | | | | | | | | |  | | | | |
| Nd | | 4.27 | | | | | | 3.15 | | | | | | | 4.07 | | | | | | | | | 0.54 | | | | | | | | | 3.12 | | | | | | | | | 10.67 | | | | | | | | | 32.19 | | | | | | | | 0.91 | | | | | | | 6.11 | | | | | | | | | 0.39 | | | | | | | | | 0.56 | | | | | | | 6.34 | | | | | | | | | 5.16 | | | | | | | | | 4.88 | | | | | | | | 9.87 | | | | | | | | | 42.17 | | | | | | | | | 8.04 | | | | | | | | |  | | | | |
| Sm | | 6.88 | | | | | | 4.32 | | | | | | | 5.84 | | | | | | | | | 0.88 | | | | | | | | | 4.75 | | | | | | | | | 22.60 | | | | | | | | | 10.07 | | | | | | | | 3.72 | | | | | | | 5.21 | | | | | | | | | 1.08 | | | | | | | | | 1.25 | | | | | | | 10.50 | | | | | | | | | 7.36 | | | | | | | | | 7.30 | | | | | | | | 7.79 | | | | | | | | | 12.92 | | | | | | | | | 13.96 | | | | | | | | |  | | | | |
| Eu | | 2.34 | | | | | | 0.29 | | | | | | | 2.70 | | | | | | | | | 0.31 | | | | | | | | | 1.98 | | | | | | | | | 8.70 | | | | | | | | | 1.98 | | | | | | | | 0.64 | | | | | | | 1.09 | | | | | | | | | 0.21 | | | | | | | | | 0.37 | | | | | | | 5.18 | | | | | | | | | 3.15 | | | | | | | | | 2.31 | | | | | | | | 2.45 | | | | | | | | | 3.17 | | | | | | | | | 6.49 | | | | | | | | |  | | | | |
| Gd | | 31.8 | | | | | | 26.3 | | | | | | | 29.5 | | | | | | | | | 2.9 | | | | | | | | | 23.2 | | | | | | | | | 88.6 | | | | | | | | | 32.9 | | | | | | | | 16.6 | | | | | | | 13.7 | | | | | | | | | 5.9 | | | | | | | | | 7.6 | | | | | | | 65.0 | | | | | | | | | 35.7 | | | | | | | | | 27.7 | | | | | | | | 32.8 | | | | | | | | | 31.1 | | | | | | | | | 57.7 | | | | | | | | |  | | | | |
| Tb | | 10.57 | | | | | | 12.73 | | | | | | | 9.84 | | | | | | | | | 1.14 | | | | | | | | | 6.68 | | | | | | | | | 26.07 | | | | | | | | | 13.28 | | | | | | | | 5.29 | | | | | | | 3.3 | | | | | | | | | 2.91 | | | | | | | | | 4 | | | | | | | 21.3 | | | | | | | | | 12.31 | | | | | | | | | 8.54 | | | | | | | | 9.69 | | | | | | | | | 8.96 | | | | | | | | | 16.85 | | | | | | | | |  | | | | |
| Dy | | 120.2 | | | | | | 190.0 | | | | | | | 122.0 | | | | | | | | | 16.6 | | | | | | | | | 74.1 | | | | | | | | | 248.1 | | | | | | | | | 171.9 | | | | | | | | 55.6 | | | | | | | 30.4 | | | | | | | | | 36.2 | | | | | | | | | 49.9 | | | | | | | 232.6 | | | | | | | | | 135.2 | | | | | | | | | 95.0 | | | | | | | | 106.9 | | | | | | | | | 98.8 | | | | | | | | | 167.3 | | | | | | | | |  | | | | |
| Ho | | 46.1 | | | | | | 85.2 | | | | | | | 48.4 | | | | | | | | | 5.6 | | | | | | | | | 26.6 | | | | | | | | | 75.0 | | | | | | | | | 69.7 | | | | | | | | 20.7 | | | | | | | 9.6 | | | | | | | | | 10.6 | | | | | | | | | 20.2 | | | | | | | 82.5 | | | | | | | | | 49.1 | | | | | | | | | 33.9 | | | | | | | | 39.4 | | | | | | | | | 36.0 | | | | | | | | | 54.8 | | | | | | | | |  | | | | |
| Er | | 210.3 | | | | | | 437.9 | | | | | | | 214.8 | | | | | | | | | 23.2 | | | | | | | | | 116.4 | | | | | | | | | 284.9 | | | | | | | | | 333.2 | | | | | | | | 95.3 | | | | | | | 38.4 | | | | | | | | | 37.6 | | | | | | | | | 101.8 | | | | | | | 350.2 | | | | | | | | | 204.6 | | | | | | | | | 151.7 | | | | | | | | 167.9 | | | | | | | | | 156.8 | | | | | | | | | 218.6 | | | | | | | | |  | | | | |
| Tm | | 44.4 | | | | | | 103.6 | | | | | | | 44.9 | | | | | | | | | 4.5 | | | | | | | | | 24.8 | | | | | | | | | 56.1 | | | | | | | | | 75.0 | | | | | | | | 23.0 | | | | | | | 7.7 | | | | | | | | | 7.0 | | | | | | | | | 26.5 | | | | | | | 66.4 | | | | | | | | | 42.0 | | | | | | | | | 30.8 | | | | | | | | 33.1 | | | | | | | | | 31.9 | | | | | | | | | 42.4 | | | | | | | | |  | | | | |
| Yb | | 428 | | | | | | 1096 | | | | | | | 427 | | | | | | | | | 39 | | | | | | | | | 240 | | | | | | | | | 488 | | | | | | | | | 754 | | | | | | | | 258 | | | | | | | 72 | | | | | | | | | 62 | | | | | | | | | 312 | | | | | | | 616 | | | | | | | | | 408 | | | | | | | | | 302 | | | | | | | | 331 | | | | | | | | | 315 | | | | | | | | | 397 | | | | | | | | |  | | | | |
| Lu | | 83 | | | | | | 209 | | | | | | | 82 | | | | | | | | | 6 | | | | | | | | | 46 | | | | | | | | | 92 | | | | | | | | | 147 | | | | | | | | 53 | | | | | | | 13 | | | | | | | | | 10 | | | | | | | | | 64 | | | | | | | 113 | | | | | | | | | 74 | | | | | | | | | 56 | | | | | | | | 62 | | | | | | | | | 60 | | | | | | | | | 73 | | | | | | | | |  | | | | |
| Pb | | 21 | | | | | | 1211 | | | | | | | 17 | | | | | | | | | 300 | | | | | | | | | 12 | | | | | | | | | 65 | | | | | | | | | 1352 | | | | | | | | 444 | | | | | | | 10 | | | | | | | | | 99 | | | | | | | | | 797 | | | | | | | 50 | | | | | | | | | 17 | | | | | | | | | 10 | | | | | | | | 13 | | | | | | | | | 10 | | | | | | | | | 440 | | | | | | | | |  | | | | |
| Th | | 306 | | | | | | 1532 | | | | | | | 257 | | | | | | | | | 93 | | | | | | | | | 110 | | | | | | | | | 602 | | | | | | | | | 747 | | | | | | | | 57 | | | | | | | 146 | | | | | | | | | 12 | | | | | | | | | 21 | | | | | | | 416 | | | | | | | | | 248 | | | | | | | | | 119 | | | | | | | | 131 | | | | | | | | | 134 | | | | | | | | | 462 | | | | | | | | |  | | | | |
| U | | 194 | | | | | | 4492 | | | | | | | 151 | | | | | | | | | 202 | | | | | | | | | 83 | | | | | | | | | 365 | | | | | | | | | 1608 | | | | | | | | 384 | | | | | | | 79 | | | | | | | | | 435 | | | | | | | | | 567 | | | | | | | 201 | | | | | | | | | 134 | | | | | | | | | 90 | | | | | | | | 91 | | | | | | | | | 108 | | | | | | | | | 406 | | | | | | | | |  | | | | |
| Y | | 1463 | | | | | | 2648 | | | | | | | 1534 | | | | | | | | | 172 | | | | | | | | | 848 | | | | | | | | | 2244 | | | | | | | | | 2181 | | | | | | | | 601 | | | | | | | 297 | | | | | | | | | 360 | | | | | | | | | 624 | | | | | | | 2535 | | | | | | | | | 1482 | | | | | | | | | 1067 | | | | | | | | 1199 | | | | | | | | | 1099 | | | | | | | | | 1622 | | | | | | | | |  | | | | |
| Hf | | 8502 | | | | | | 13789 | | | | | | | 7641 | | | | | | | | | 8491 | | | | | | | | | 9792 | | | | | | | | | 9450 | | | | | | | | | 10078 | | | | | | | | 12011 | | | | | | | 6866 | | | | | | | | | 11039 | | | | | | | | | 11402 | | | | | | | 7699 | | | | | | | | | 7275 | | | | | | | | | 8069 | | | | | | | | 7544 | | | | | | | | | 9006 | | | | | | | | | 8529 | | | | | | | | |  | | | | |
| Nb | | 2.18 | | | | | | 39.52 | | | | | | | 2.33 | | | | | | | | | 0.61 | | | | | | | | | 0.87 | | | | | | | | | 2.60 | | | | | | | | | 6.06 | | | | | | | | 0.87 | | | | | | | 0.62 | | | | | | | | | 2.97 | | | | | | | | | 0.70 | | | | | | | 3.49 | | | | | | | | | 1.44 | | | | | | | | | 0.68 | | | | | | | | 1.51 | | | | | | | | | 1.89 | | | | | | | | | 0.97 | | | | | | | | |  | | | | |
| Ta | | 0.79 | | | | | | 23.26 | | | | | | | 0.69 | | | | | | | | | 0.15 | | | | | | | | | 0.37 | | | | | | | | | 1.12 | | | | | | | | | 3.54 | | | | | | | | 0.38 | | | | | | | 0.16 | | | | | | | | | 2.66 | | | | | | | | | 0.67 | | | | | | | 0.74 | | | | | | | | | 0.54 | | | | | | | | | 0.42 | | | | | | | | 0.40 | | | | | | | | | 0.62 | | | | | | | | | 0.35 | | | | | | | | |  | | | | |
| P | |  | | | | | |  | | | | | | |  | | | | | | | | |  | | | | | | | | |  | | | | | | | | |  | | | | | | | | |  | | | | | | | |  | | | | | | |  | | | | | | | | |  | | | | | | | | |  | | | | | | |  | | | | | | | | |  | | | | | | | | |  | | | | | | | |  | | | | | | | | |  | | | | | | | | |  | | | | | | | | |  | | | | |
| LREE | | 30 | | | | | | 27 | | | | | | | 25 | | | | | | | | | 3 | | | | | | | | | 18 | | | | | | | | | 83 | | | | | | | | | 174 | | | | | | | | 2 | | | | | | | 25 | | | | | | | | | 1 | | | | | | | | | 3 | | | | | | | 32 | | | | | | | | | 23 | | | | | | | | | 20 | | | | | | | | 26 | | | | | | | | | 205 | | | | | | | | | 59 | | | | | | | | |  | | | | |
| MREE | | 172 | | | | | | 234 | | | | | | | 170 | | | | | | | | | 22 | | | | | | | | | 111 | | | | | | | | | 394 | | | | | | | | | 230 | | | | | | | | 82 | | | | | | | 54 | | | | | | | | | 46 | | | | | | | | | 63 | | | | | | | 335 | | | | | | | | | 194 | | | | | | | | | 141 | | | | | | | | 160 | | | | | | | | | 155 | | | | | | | | | 262 | | | | | | | | |  | | | | |
| HREE | | 811 | | | | | | 1932 | | | | | | | 818 | | | | | | | | | 78 | | | | | | | | | 454 | | | | | | | | | 996 | | | | | | | | | 1380 | | | | | | | | 450 | | | | | | | 141 | | | | | | | | | 127 | | | | | | | | | 525 | | | | | | | 1228 | | | | | | | | | 777 | | | | | | | | | 574 | | | | | | | | 634 | | | | | | | | | 599 | | | | | | | | | 786 | | | | | | | | |  | | | | |
| (Yb/La)_n_ | | 2709.2 | | | | | | 1140.6 | | | | | | | 3133.5 | | | | | | | | | 317.2 | | | | | | | | | 1969.0 | | | | | | | | | 2001.7 | | | | | | | | | 30.2 | | | | | | | | 2517.8 | | | | | | | 265.5 | | | | | | | | | 379.7 | | | | | | | | | 2527.0 | | | | | | | 3901.8 | | | | | | | | | 3344.2 | | | | | | | | | 2446.8 | | | | | | | | 2200.6 | | | | | | | | | 9.7 | | | | | | | | | 3818.6 | | | | | | | | |  | | | | |
| (Sm/La)_n_ | | 48.4 | | | | | | 5.0 | | | | | | | 47.6 | | | | | | | | | 8.0 | | | | | | | | | 43.3 | | | | | | | | | 103.0 | | | | | | | | | 0.4 | | | | | | | | 40.3 | | | | | | | 21.2 | | | | | | | | | 7.3 | | | | | | | | | 11.3 | | | | | | | 73.9 | | | | | | | | | 67.1 | | | | | | | | | 65.7 | | | | | | | | 57.5 | | | | | | | | | 0.4 | | | | | | | | | 149.1 | | | | | | | | |  | | | | |
| (Lu/Gd)_n_ | | 21.1 | | | | | | 64.4 | | | | | | | 22.6 | | | | | | | | | 17.6 | | | | | | | | | 16.0 | | | | | | | | | 8.4 | | | | | | | | | 36.3 | | | | | | | | 25.9 | | | | | | | 7.9 | | | | | | | | | 13.6 | | | | | | | | | 68.1 | | | | | | | 14.1 | | | | | | | | | 16.7 | | | | | | | | | 16.3 | | | | | | | | 15.3 | | | | | | | | | 15.6 | | | | | | | | | 10.3 | | | | | | | | |  | | | | |
| Eu/Eu* | | 0.40 | | | | | | 0.06 | | | | | | | 0.51 | | | | | | | | | 0.53 | | | | | | | | | 0.47 | | | | | | | | | 0.52 | | | | | | | | | 0.30 | | | | | | | | 0.21 | | | | | | | 0.37 | | | | | | | | | 0.20 | | | | | | | | | 0.28 | | | | | | | 0.46 | | | | | | | | | 0.49 | | | | | | | | | 0.44 | | | | | | | | 0.40 | | | | | | | | | 0.46 | | | | | | | | | 0.60 | | | | | | | | |  | | | | |
| Ce/Ce* | | 28.80 | | | | | | 6.23 | | | | | | | 16.98 | | | | | | | | | 3.34 | | | | | | | | | 18.98 | | | | | | | | | 31.79 | | | | | | | | | 1.41 | | | | | | | | 1.72 | | | | | | | 8.88 | | | | | | | | | 1.21 | | | | | | | | | 3.41 | | | | | | | 12.06 | | | | | | | | | 14.12 | | | | | | | | | 11.79 | | | | | | | | 3.38 | | | | | | | | | 1.19 | | | | | | | | | 26.32 | | | | | | | | |  | | | | |
| U/Yb | | 0.453 | | | | | | 4.097 | | | | | | | 0.354 | | | | | | | | | 5.216 | | | | | | | | | 0.347 | | | | | | | | | 0.748 | | | | | | | | | 2.132 | | | | | | | | 1.486 | | | | | | | 1.098 | | | | | | | | | 7.009 | | | | | | | | | 1.818 | | | | | | | 0.326 | | | | | | | | | 0.328 | | | | | | | | | 0.298 | | | | | | | | 0.274 | | | | | | | | | 0.344 | | | | | | | | | 1.022 | | | | | | | | |  | | | | |
| Nb/Yb | | 0.005 | | | | | | 0.036 | | | | | | | 0.005 | | | | | | | | | 0.016 | | | | | | | | | 0.004 | | | | | | | | | 0.005 | | | | | | | | | 0.008 | | | | | | | | 0.003 | | | | | | | 0.009 | | | | | | | | | 0.048 | | | | | | | | | 0.002 | | | | | | | 0.006 | | | | | | | | | 0.004 | | | | | | | | | 0.002 | | | | | | | | 0.005 | | | | | | | | | 0.006 | | | | | | | | | 0.002 | | | | | | | | |  | | | | |
|  | | | | | | | | | | | | | | | | | | | | | | | | | | | | | | | | | | | | | | | | | | | | | | | | | | | | | | | | | | | | | | | | | | | | | | | | | | | | | | | | | | | | | | | | | | | | | | | | | | | | | | | | | | | | | | | | | | | | | | | | | | | | | | | | | | | | | | | | | | | | | | | | |  | | | |
|  | | | | | | | | | | | | | | | | | | | | | | | | | | | | | | | | | | | | | | | | | | | | | | | | | | | | | | | | | | | | | | | | | | | | | | | | | | | | | | | | | | | | | | | | | | | | | | | | | | | | | | | | | | | | | | | | | | | | | | | | | | | | | | | | | | | | | | | | | | | | | | | | |  | | | |
| Table S4 (continued) | | | | | | | | | | | | | | | | | | | | | | | | | | | | | | | | | | | | | | | | | | | | | | | | | | | | | | | | | | | | | | | | | | | | | | | | | | | | | | | | | | | | | | | | | | | | | | | | | | | | | | | | | | | | | | | | | | | | | | | | | | | | | | | | | | | | | | | | | | | | | | | | |  | | | |
| Sample | | 07CL07 | | | | | | | | | | | | | | | | | | | | | | | |  | | | | | | | | 07CL08 | | | | | | | | | | | | | | | | | | | | | | | | | | | | | | | | | | | | | | | | | | | | | | | | | | | | | | | | | | | | | | | | | | | | | | | | | | | | | | | | | | | | | | | | | | | | | | | | | | | | | | | | | | | | | | |  | | | |
| Spot | | 18 | | | | | | | | | 19 | | | | | | 20 | | | | | | | | |  | | | | | | | | 1 | | | | | | | | | 2 | | | | | | | | | 3 | | | | | | | | | 4 | | | | | | | | 5 | | | | | | | 6 | | | | | | | | | 7 | | | | | | | | 8 | | | | | | | | | 9 | | | | | | | | | 10 | | | | | | | 11 | | | | | | | | | | 12 | | | | | | | | 13 | | | | | | | | |  | | | |
| La | | 741.90 | | | | | | | | | 15.24 | | | | | | 68.50 | | | | | | | | |  | | | | | | | | 0.18 | | | | | | | | | 0.06 | | | | | | | | | 0.12 | | | | | | | | | 2.47 | | | | | | | | 0.10 | | | | | | | 0.22 | | | | | | | | | 1.16 | | | | | | | | 110.48 | | | | | | | | | 1.75 | | | | | | | | | 0.39 | | | | | | | 1.98 | | | | | | | | | | 0.40 | | | | | | | | 0.24 | | | | | | | | |  | | | |
| Ce | | 1635.76 | | | | | | | | | 41.58 | | | | | | 165.15 | | | | | | | | |  | | | | | | | | 12.76 | | | | | | | | | 6.07 | | | | | | | | | 18.59 | | | | | | | | | 96.50 | | | | | | | | 12.99 | | | | | | | 8.07 | | | | | | | | | 27.44 | | | | | | | | 319.83 | | | | | | | | | 26.80 | | | | | | | | | 8.42 | | | | | | | 15.72 | | | | | | | | | | 10.90 | | | | | | | | 8.83 | | | | | | | | |  | | | |
| Pr | | 209.71 | | | | | | | | | 5.38 | | | | | | 19.23 | | | | | | | | |  | | | | | | | | 0.12 | | | | | | | | | 0.06 | | | | | | | | | 0.11 | | | | | | | | | 1.42 | | | | | | | | 0.18 | | | | | | | 0.20 | | | | | | | | | 0.87 | | | | | | | | 37.54 | | | | | | | | | 1.24 | | | | | | | | | 0.15 | | | | | | | 0.47 | | | | | | | | | | 0.18 | | | | | | | | 0.16 | | | | | | | | |  | | | |
| Nd | | 867.21 | | | | | | | | | 27.44 | | | | | | 71.86 | | | | | | | | |  | | | | | | | | 1.40 | | | | | | | | | 0.57 | | | | | | | | | 1.67 | | | | | | | | | 9.73 | | | | | | | | 1.84 | | | | | | | 1.90 | | | | | | | | | 7.89 | | | | | | | | 168.54 | | | | | | | | | 8.81 | | | | | | | | | 1.77 | | | | | | | 3.60 | | | | | | | | | | 2.72 | | | | | | | | 1.63 | | | | | | | | |  | | | |
| Sm | | 146.94 | | | | | | | | | 14.53 | | | | | | 17.65 | | | | | | | | |  | | | | | | | | 2.89 | | | | | | | | | 1.17 | | | | | | | | | 3.53 | | | | | | | | | 7.67 | | | | | | | | 5.26 | | | | | | | 2.55 | | | | | | | | | 6.63 | | | | | | | | 41.58 | | | | | | | | | 5.66 | | | | | | | | | 2.85 | | | | | | | 1.96 | | | | | | | | | | 4.56 | | | | | | | | 2.02 | | | | | | | | |  | | | |
| Eu | | 10.93 | | | | | | | | | 1.45 | | | | | | 0.67 | | | | | | | | |  | | | | | | | | 1.16 | | | | | | | | | 0.73 | | | | | | | | | 1.42 | | | | | | | | | 1.48 | | | | | | | | 1.05 | | | | | | | 0.78 | | | | | | | | | 1.05 | | | | | | | | 2.85 | | | | | | | | | 1.05 | | | | | | | | | 0.93 | | | | | | | 0.50 | | | | | | | | | | 0.98 | | | | | | | | 0.53 | | | | | | | | |  | | | |
| Gd | | 101.8 | | | | | | | | | 41.7 | | | | | | 28.8 | | | | | | | | |  | | | | | | | | 12.5 | | | | | | | | | 5.5 | | | | | | | | | 15.5 | | | | | | | | | 28.3 | | | | | | | | 26.8 | | | | | | | 13.0 | | | | | | | | | 34.8 | | | | | | | | 49.1 | | | | | | | | | 27.3 | | | | | | | | | 22.1 | | | | | | | 14.3 | | | | | | | | | | 23.2 | | | | | | | | 13.2 | | | | | | | | |  | | | |
| Tb | | 16.91 | | | | | | | | | 14.17 | | | | | | 7.14 | | | | | | | | |  | | | | | | | | 4.02 | | | | | | | | | 1.78 | | | | | | | | | 5.16 | | | | | | | | | 10.55 | | | | | | | | 10.59 | | | | | | | 5.79 | | | | | | | | | 11.24 | | | | | | | | 11.47 | | | | | | | | | 11.23 | | | | | | | | | 7.97 | | | | | | | 5.69 | | | | | | | | | | 8.71 | | | | | | | | 4.55 | | | | | | | | |  | | | |
| Dy | | 153.2 | | | | | | | | | 174.0 | | | | | | 85.0 | | | | | | | | |  | | | | | | | | 47.4 | | | | | | | | | 20.9 | | | | | | | | | 59.2 | | | | | | | | | 128.9 | | | | | | | | 142.0 | | | | | | | 94.0 | | | | | | | | | 144.1 | | | | | | | | 115.5 | | | | | | | | | 148.8 | | | | | | | | | 100.1 | | | | | | | 87.8 | | | | | | | | | | 106.8 | | | | | | | | 67.8 | | | | | | | | |  | | | |
| Ho | | 59.6 | | | | | | | | | 74.3 | | | | | | 32.7 | | | | | | | | |  | | | | | | | | 18.0 | | | | | | | | | 7.6 | | | | | | | | | 21.5 | | | | | | | | | 50.9 | | | | | | | | 54.3 | | | | | | | 34.4 | | | | | | | | | 55.1 | | | | | | | | 39.7 | | | | | | | | | 67.8 | | | | | | | | | 41.5 | | | | | | | 40.5 | | | | | | | | | | 48.6 | | | | | | | | 31.5 | | | | | | | | |  | | | |
| Er | | 314.2 | | | | | | | | | 412.8 | | | | | | 148.4 | | | | | | | | |  | | | | | | | | 83.4 | | | | | | | | | 35.1 | | | | | | | | | 99.0 | | | | | | | | | 247.4 | | | | | | | | 240.1 | | | | | | | 183.8 | | | | | | | | | 236.6 | | | | | | | | 171.7 | | | | | | | | | 358.5 | | | | | | | | | 187.4 | | | | | | | 218.3 | | | | | | | | | | 250.5 | | | | | | | | 162.6 | | | | | | | | |  | | | |
| Tm | | 79.6 | | | | | | | | | 101.7 | | | | | | 32.3 | | | | | | | | |  | | | | | | | | 19.5 | | | | | | | | | 8.1 | | | | | | | | | 22.1 | | | | | | | | | 57.5 | | | | | | | | 53.1 | | | | | | | 47.4 | | | | | | | | | 51.3 | | | | | | | | 36.7 | | | | | | | | | 89.5 | | | | | | | | | 41.3 | | | | | | | 54.6 | | | | | | | | | | 61.0 | | | | | | | | 42.0 | | | | | | | | |  | | | |
| Yb | | 851 | | | | | | | | | 1156 | | | | | | 304 | | | | | | | | |  | | | | | | | | 204 | | | | | | | | | 86 | | | | | | | | | 236 | | | | | | | | | 597 | | | | | | | | 509 | | | | | | | 521 | | | | | | | | | 498 | | | | | | | | 368 | | | | | | | | | 999 | | | | | | | | | 399 | | | | | | | 607 | | | | | | | | | | 668 | | | | | | | | 480 | | | | | | | | |  | | | |
| Lu | | 166 | | | | | | | | | 249 | | | | | | 58 | | | | | | | | |  | | | | | | | | 43 | | | | | | | | | 19 | | | | | | | | | 47 | | | | | | | | | 111 | | | | | | | | 96 | | | | | | | 114 | | | | | | | | | 89 | | | | | | | | 66 | | | | | | | | | 210 | | | | | | | | | 78 | | | | | | | 136 | | | | | | | | | | 147 | | | | | | | | 105 | | | | | | | | |  | | | |
| Pb | | 1276 | | | | | | | | | 1290 | | | | | | 37 | | | | | | | | |  | | | | | | | | 39 | | | | | | | | | 14 | | | | | | | | | 811 | | | | | | | | | 794 | | | | | | | | 27 | | | | | | | 198 | | | | | | | | | 256 | | | | | | | | 947 | | | | | | | | | 517 | | | | | | | | | 105 | | | | | | | 263 | | | | | | | | | | 247 | | | | | | | | 251 | | | | | | | | |  | | | |
| Th | | 1246 | | | | | | | | | 8966 | | | | | | 181 | | | | | | | | |  | | | | | | | | 68 | | | | | | | | | 24 | | | | | | | | | 369 | | | | | | | | | 463 | | | | | | | | 601 | | | | | | | 318 | | | | | | | | | 426 | | | | | | | | 538 | | | | | | | | | 1090 | | | | | | | | | 188 | | | | | | | 423 | | | | | | | | | | 427 | | | | | | | | 375 | | | | | | | | |  | | | |
| U | | 2557 | | | | | | | | | 8942 | | | | | | 267 | | | | | | | | |  | | | | | | | | 45 | | | | | | | | | 14 | | | | | | | | | 172 | | | | | | | | | 348 | | | | | | | | 237 | | | | | | | 154 | | | | | | | | | 316 | | | | | | | | 653 | | | | | | | | | 521 | | | | | | | | | 81 | | | | | | | 166 | | | | | | | | | | 254 | | | | | | | | 168 | | | | | | | | |  | | | |
| Y | | 1887 | | | | | | | | | 2360 | | | | | | 989 | | | | | | | | |  | | | | | | | | 562 | | | | | | | | | 234 | | | | | | | | | 673 | | | | | | | | | 1623 | | | | | | | | 1620 | | | | | | | 1144 | | | | | | | | | 1589 | | | | | | | | 1178 | | | | | | | | | 2196 | | | | | | | | | 1177 | | | | | | | 1321 | | | | | | | | | | 1543 | | | | | | | | 1021 | | | | | | | | |  | | | |
| Hf | | 11868 | | | | | | | | | 11743 | | | | | | 10168 | | | | | | | | |  | | | | | | | | 9457 | | | | | | | | | 8841 | | | | | | | | | 9152 | | | | | | | | | 9288 | | | | | | | | 10323 | | | | | | | 9360 | | | | | | | | | 10287 | | | | | | | | 9976 | | | | | | | | | 10620 | | | | | | | | | 9706 | | | | | | | 9353 | | | | | | | | | | 8899 | | | | | | | | 10107 | | | | | | | | |  | | | |
| Nb | | 60.67 | | | | | | | | | 32.82 | | | | | | 2.94 | | | | | | | | |  | | | | | | | | 0.53 | | | | | | | | | 0.31 | | | | | | | | | 1.27 | | | | | | | | | 11.90 | | | | | | | | 1.80 | | | | | | | 1.67 | | | | | | | | | 6.05 | | | | | | | | 8.28 | | | | | | | | | 7.03 | | | | | | | | | 1.06 | | | | | | | 2.96 | | | | | | | | | | 1.19 | | | | | | | | 2.00 | | | | | | | | |  | | | |
| Ta | | 22.08 | | | | | | | | | 21.19 | | | | | | 1.02 | | | | | | | | |  | | | | | | | | 0.23 | | | | | | | | | 0.09 | | | | | | | | | 0.44 | | | | | | | | | 2.54 | | | | | | | | 1.26 | | | | | | | 0.39 | | | | | | | | | 1.82 | | | | | | | | 2.44 | | | | | | | | | 2.49 | | | | | | | | | 0.55 | | | | | | | 0.70 | | | | | | | | | | 0.32 | | | | | | | | 0.48 | | | | | | | | |  | | | |
| P | |  | | | | | | | | |  | | | | | |  | | | | | | | | |  | | | | | | | |  | | | | | | | | |  | | | | | | | | |  | | | | | | | | |  | | | | | | | |  | | | | | | |  | | | | | | | | |  | | | | | | | |  | | | | | | | | |  | | | | | | | | |  | | | | | | |  | | | | | | | | | |  | | | | | | | |  | | | | | | | | |  | | | |
| LREE | | 3455 | | | | | | | | | 90 | | | | | | 325 | | | | | | | | |  | | | | | | | | 14 | | | | | | | | | 7 | | | | | | | | | 20 | | | | | | | | | 110 | | | | | | | | 15 | | | | | | | 10 | | | | | | | | | 37 | | | | | | | | 636 | | | | | | | | | 39 | | | | | | | | | 11 | | | | | | | 22 | | | | | | | | | | 14 | | | | | | | | 11 | | | | | | | | |  | | | |
| MREE | | 430 | | | | | | | | | 246 | | | | | | 139 | | | | | | | | |  | | | | | | | | 68 | | | | | | | | | 30 | | | | | | | | | 85 | | | | | | | | | 177 | | | | | | | | 186 | | | | | | | 116 | | | | | | | | | 198 | | | | | | | | 220 | | | | | | | | | 194 | | | | | | | | | 134 | | | | | | | 110 | | | | | | | | | | 144 | | | | | | | | 88 | | | | | | | | |  | | | |
| HREE | | 1471 | | | | | | | | | 1994 | | | | | | 576 | | | | | | | | |  | | | | | | | | 368 | | | | | | | | | 156 | | | | | | | | | 426 | | | | | | | | | 1063 | | | | | | | | 953 | | | | | | | 901 | | | | | | | | | 930 | | | | | | | | 683 | | | | | | | | | 1725 | | | | | | | | | 748 | | | | | | | 1056 | | | | | | | | | | 1175 | | | | | | | | 821 | | | | | | | | |  | | | |
| (Yb/La)_n_ | | 1.6 | | | | | | | | | 105.7 | | | | | | 6.2 | | | | | | | | |  | | | | | | | | 1560.3 | | | | | | | | | 1908.2 | | | | | | | | | 2789.9 | | | | | | | | | 336.7 | | | | | | | | 6825.0 | | | | | | | 3303.8 | | | | | | | | | 598.8 | | | | | | | | 4.6 | | | | | | | | | 795.8 | | | | | | | | | 1428.0 | | | | | | | 427.5 | | | | | | | | | | 2328.1 | | | | | | | | 2787.7 | | | | | | | | |  | | | |
| (Sm/La)_n_ | | 0.3 | | | | | | | | | 1.5 | | | | | | 0.4 | | | | | | | | |  | | | | | | | | 24.6 | | | | | | | | | 28.8 | | | | | | | | | 46.3 | | | | | | | | | 4.8 | | | | | | | | 78.3 | | | | | | | 18.0 | | | | | | | | | 8.9 | | | | | | | | 0.6 | | | | | | | | | 5.0 | | | | | | | | | 11.3 | | | | | | | 1.5 | | | | | | | | | | 17.7 | | | | | | | | 13.0 | | | | | | | | |  | | | |
| (Lu/Gd)_n_ | | 13.2 | | | | | | | | | 48.4 | | | | | | 16.4 | | | | | | | | |  | | | | | | | | 27.9 | | | | | | | | | 27.5 | | | | | | | | | 24.7 | | | | | | | | | 31.8 | | | | | | | | 29.1 | | | | | | | 70.8 | | | | | | | | | 20.7 | | | | | | | | 10.9 | | | | | | | | | 62.2 | | | | | | | | | 28.5 | | | | | | | 76.8 | | | | | | | | | | 51.4 | | | | | | | | 64.3 | | | | | | | | |  | | | |
| Eu/Eu* | | 0.26 | | | | | | | | | 0.17 | | | | | | 0.09 | | | | | | | | |  | | | | | | | | 0.50 | | | | | | | | | 0.73 | | | | | | | | | 0.50 | | | | | | | | | 0.27 | | | | | | | | 0.22 | | | | | | | 0.34 | | | | | | | | | 0.17 | | | | | | | | 0.19 | | | | | | | | | 0.21 | | | | | | | | | 0.25 | | | | | | | 0.21 | | | | | | | | | | 0.24 | | | | | | | | 0.24 | | | | | | | | |  | | | |
| Ce/Ce* | | 1.00 | | | | | | | | | 1.12 | | | | | | 1.10 | | | | | | | | |  | | | | | | | | 20.85 | | | | | | | | | 23.48 | | | | | | | | | 36.46 | | | | | | | | | 12.43 | | | | | | | | 18.19 | | | | | | | 8.69 | | | | | | | | | 6.38 | | | | | | | | 1.21 | | | | | | | | | 4.29 | | | | | | | | | 8.53 | | | | | | | 3.86 | | | | | | | | | | 9.94 | | | | | | | | 10.87 | | | | | | | | |  | | | |
| U/Yb | | 3.003 | | | | | | | | | 7.737 | | | | | | 0.877 | | | | | | | | |  | | | | | | | | 0.221 | | | | | | | | | 0.158 | | | | | | | | | 0.728 | | | | | | | | | 0.584 | | | | | | | | 0.466 | | | | | | | 0.296 | | | | | | | | | 0.635 | | | | | | | | 1.773 | | | | | | | | | 0.522 | | | | | | | | | 0.202 | | | | | | | 0.273 | | | | | | | | | | 0.380 | | | | | | | | 0.350 | | | | | | | | |  | | | |
| Nb/Yb | | 0.071 | | | | | | | | | 0.028 | | | | | | 0.010 | | | | | | | | |  | | | | | | | | 0.003 | | | | | | | | | 0.004 | | | | | | | | | 0.005 | | | | | | | | | 0.020 | | | | | | | | 0.004 | | | | | | | 0.003 | | | | | | | | | 0.012 | | | | | | | | 0.022 | | | | | | | | | 0.007 | | | | | | | | | 0.003 | | | | | | | 0.005 | | | | | | | | | | 0.002 | | | | | | | | 0.004 | | | | | | | | |  | | | |
| Table S4 (continued) | | | | | | | | | | | | | | | | | | | | | | | | | | | | | | | | | | | | | | | | | | | | | | | | | | | | | | | | | | | | | | | | | | | | | | | | | | | | | | | | | | | | | | | | | | | | | | | | | | | | | | | | | | | | | | | | | | | | | | | | | | | | | | | | | | | | | | | | | | | | | | | | | | |  | |
| Sample | 07CL08 | | | | | | | | | | | | | | | | | | | | | | | | | | | | | | | | | | | | | | | | | | | | | | | | | | | | |  | | | | | | 07CL13 | | | | | | | | | | | | | | | | | | | | | | | | | | | | | | | | | | | | | | | | | | | | | | | | | | | | | | | | | | | | | | | | | | | | | | | | | | | | | | | | | | | | | | |  | |
| Spot | 14 | | | 15 | | | | | | | | 16 | | | | | | | | 17 | | | | | | | | 18 | | | | | | | | 19 | | | | | | | | | 20 | | | | | | | | |  | | | | | | 1 | | | | | | | 2 | | | | | | | 3 | | | | | | | | 4 | | | | | | | | 5 | | | | | | | | 6 | | | | | | | | | 7 | | | | | | | | | 8 | | | | | | | | | 9 | | | | | | | 10 | | | | | | 11 | | | | | | | | |  | |
| La | 0.89 | | | 0.41 | | | | | | | | 0.11 | | | | | | | | 5.70 | | | | | | | | 0.12 | | | | | | | | 3.24 | | | | | | | | | 0.22 | | | | | | | | |  | | | | | | 132.75 | | | | | | | 1.30 | | | | | | | 0.11 | | | | | | | | 5.68 | | | | | | | | 1.70 | | | | | | | | 4.01 | | | | | | | | | 7.10 | | | | | | | | | 47.11 | | | | | | | | | 104.79 | | | | | | | 2.86 | | | | | | 5.60 | | | | | | | | |  | |
| Ce | 13.50 | | | 4.52 | | | | | | | | 2.52 | | | | | | | | 35.28 | | | | | | | | 9.97 | | | | | | | | 39.33 | | | | | | | | | 15.54 | | | | | | | | |  | | | | | | 273.75 | | | | | | | 24.37 | | | | | | | 42.30 | | | | | | | | 44.67 | | | | | | | | 16.87 | | | | | | | | 40.28 | | | | | | | | | 27.88 | | | | | | | | | 444.84 | | | | | | | | | 341.35 | | | | | | | 26.22 | | | | | | 49.28 | | | | | | | | |  | |
| Pr | 0.52 | | | 0.93 | | | | | | | | 0.07 | | | | | | | | 8.27 | | | | | | | | 0.09 | | | | | | | | 5.83 | | | | | | | | | 0.14 | | | | | | | | |  | | | | | | 28.50 | | | | | | | 1.17 | | | | | | | 1.07 | | | | | | | | 4.88 | | | | | | | | 0.85 | | | | | | | | 1.16 | | | | | | | | | 2.97 | | | | | | | | | 46.87 | | | | | | | | | 42.39 | | | | | | | 2.66 | | | | | | 6.31 | | | | | | | | |  | |
| Nd | 3.53 | | | 10.82 | | | | | | | | 0.27 | | | | | | | | 64.35 | | | | | | | | 1.63 | | | | | | | | 47.67 | | | | | | | | | 1.33 | | | | | | | | |  | | | | | | 114.23 | | | | | | | 10.68 | | | | | | | 13.91 | | | | | | | | 23.22 | | | | | | | | 5.51 | | | | | | | | 14.40 | | | | | | | | | 15.73 | | | | | | | | | 270.36 | | | | | | | | | 214.41 | | | | | | | 15.58 | | | | | | 44.26 | | | | | | | | |  | |
| Sm | 3.90 | | | 16.59 | | | | | | | | 0.55 | | | | | | | | 70.03 | | | | | | | | 2.69 | | | | | | | | 58.05 | | | | | | | | | 2.33 | | | | | | | | |  | | | | | | 27.15 | | | | | | | 7.91 | | | | | | | 15.38 | | | | | | | | 16.69 | | | | | | | | 9.54 | | | | | | | | 20.95 | | | | | | | | | 9.39 | | | | | | | | | 124.77 | | | | | | | | | 75.12 | | | | | | | 9.08 | | | | | | 51.80 | | | | | | | | |  | |
| Eu | 0.77 | | | 4.38 | | | | | | | | 0.29 | | | | | | | | 23.23 | | | | | | | | 0.65 | | | | | | | | 22.90 | | | | | | | | | 0.48 | | | | | | | | |  | | | | | | 2.18 | | | | | | | 0.86 | | | | | | | 1.40 | | | | | | | | 3.59 | | | | | | | | 1.57 | | | | | | | | 1.42 | | | | | | | | | 1.17 | | | | | | | | | 37.37 | | | | | | | | | 16.31 | | | | | | | 2.15 | | | | | | 20.72 | | | | | | | | |  | |
| Gd | 15.2 | | | 62.7 | | | | | | | | 2.7 | | | | | | | | 132.1 | | | | | | | | 19.3 | | | | | | | | 184.9 | | | | | | | | | 14.3 | | | | | | | | |  | | | | | | 46.6 | | | | | | | 25.0 | | | | | | | 41.4 | | | | | | | | 46.7 | | | | | | | | 40.3 | | | | | | | | 87.2 | | | | | | | | | 31.5 | | | | | | | | | 154.3 | | | | | | | | | 106.2 | | | | | | | 29.1 | | | | | | 152.6 | | | | | | | | |  | |
| Tb | 6.96 | | | 21.34 | | | | | | | | 0.88 | | | | | | | | 35.57 | | | | | | | | 6.86 | | | | | | | | 60.38 | | | | | | | | | 5.73 | | | | | | | | |  | | | | | | 10.84 | | | | | | | 6.19 | | | | | | | 11.35 | | | | | | | | 16.36 | | | | | | | | 13.64 | | | | | | | | 27.64 | | | | | | | | | 9.73 | | | | | | | | | 29.04 | | | | | | | | | 24.55 | | | | | | | 10.63 | | | | | | 35.63 | | | | | | | | |  | |
| Dy | 103.3 | | | 224.5 | | | | | | | | 10.7 | | | | | | | | 309.5 | | | | | | | | 104.5 | | | | | | | | 509.2 | | | | | | | | | 77.9 | | | | | | | | |  | | | | | | 98.2 | | | | | | | 59.4 | | | | | | | 110.0 | | | | | | | | 183.7 | | | | | | | | 147.2 | | | | | | | | 287.3 | | | | | | | | | 106.8 | | | | | | | | | 207.2 | | | | | | | | | 191.1 | | | | | | | 118.9 | | | | | | 274.5 | | | | | | | | |  | |
| Ho | 52.5 | | | 76.9 | | | | | | | | 4.5 | | | | | | | | 83.2 | | | | | | | | 46.7 | | | | | | | | 118.4 | | | | | | | | | 32.2 | | | | | | | | |  | | | | | | 30.3 | | | | | | | 18.0 | | | | | | | 35.7 | | | | | | | | 61.4 | | | | | | | | 52.2 | | | | | | | | 98.2 | | | | | | | | | 36.9 | | | | | | | | | 49.8 | | | | | | | | | 47.8 | | | | | | | 43.8 | | | | | | 70.3 | | | | | | | | |  | |
| Er | 292.4 | | | 287.6 | | | | | | | | 24.6 | | | | | | | | 313.6 | | | | | | | | 229.9 | | | | | | | | 390.2 | | | | | | | | | 159.7 | | | | | | | | |  | | | | | | 119.1 | | | | | | | 73.7 | | | | | | | 148.4 | | | | | | | | 276.1 | | | | | | | | 221.0 | | | | | | | | 393.3 | | | | | | | | | 156.7 | | | | | | | | | 162.9 | | | | | | | | | 177.3 | | | | | | | 197.1 | | | | | | 231.3 | | | | | | | | |  | |
| Tm | 77.2 | | | 54.6 | | | | | | | | 6.4 | | | | | | | | 63.7 | | | | | | | | 56.6 | | | | | | | | 72.3 | | | | | | | | | 36.2 | | | | | | | | |  | | | | | | 23.3 | | | | | | | 15.3 | | | | | | | 30.3 | | | | | | | | 58.1 | | | | | | | | 42.9 | | | | | | | | 73.8 | | | | | | | | | 32.3 | | | | | | | | | 28.2 | | | | | | | | | 34.9 | | | | | | | 41.5 | | | | | | 46.7 | | | | | | | | |  | |
| Yb | 920 | | | 464 | | | | | | | | 66 | | | | | | | | 578 | | | | | | | | 650 | | | | | | | | 666 | | | | | | | | | 372 | | | | | | | | |  | | | | | | 208 | | | | | | | 138 | | | | | | | 285 | | | | | | | | 516 | | | | | | | | 392 | | | | | | | | 642 | | | | | | | | | 302 | | | | | | | | | 246 | | | | | | | | | 319 | | | | | | | 409 | | | | | | 461 | | | | | | | | |  | |
| Lu | 200 | | | 80 | | | | | | | | 12 | | | | | | | | 92 | | | | | | | | 139 | | | | | | | | 112 | | | | | | | | | 73 | | | | | | | | |  | | | | | | 37 | | | | | | | 25 | | | | | | | 51 | | | | | | | | 86 | | | | | | | | 73 | | | | | | | | 115 | | | | | | | | | 57 | | | | | | | | | 46 | | | | | | | | | 55 | | | | | | | 76 | | | | | | 78 | | | | | | | | |  | |
| Pb | 375 | | | 679 | | | | | | | | 65 | | | | | | | | 1384 | | | | | | | | 256 | | | | | | | | 472 | | | | | | | | | 52 | | | | | | | | |  | | | | | | 22 | | | | | | | 11 | | | | | | | 110 | | | | | | | | 84 | | | | | | | | 30 | | | | | | | | 55 | | | | | | | | | 31 | | | | | | | | | 1959 | | | | | | | | | 759 | | | | | | | 392 | | | | | | 639 | | | | | | | | |  | |
| Th | 1128 | | | 511 | | | | | | | | 87 | | | | | | | | 1900 | | | | | | | | 418 | | | | | | | | 873 | | | | | | | | | 298 | | | | | | | | |  | | | | | | 459 | | | | | | | 191 | | | | | | | 2378 | | | | | | | | 356 | | | | | | | | 176 | | | | | | | | 816 | | | | | | | | | 176 | | | | | | | | | 103 | | | | | | | | | 459 | | | | | | | 74 | | | | | | 628 | | | | | | | | |  | |
| U | 333 | | | 174 | | | | | | | | 30 | | | | | | | | 124 | | | | | | | | 186 | | | | | | | | 706 | | | | | | | | | 165 | | | | | | | | |  | | | | | | 206 | | | | | | | 105 | | | | | | | 1301 | | | | | | | | 464 | | | | | | | | 242 | | | | | | | | 582 | | | | | | | | | 323 | | | | | | | | | 1518 | | | | | | | | | 528 | | | | | | | 369 | | | | | | 1813 | | | | | | | | |  | |
| Y | 1742 | | | 2220 | | | | | | | | 134 | | | | | | | | 2641 | | | | | | | | 1484 | | | | | | | | 3091 | | | | | | | | | 1008 | | | | | | | | |  | | | | | | 924 | | | | | | | 557 | | | | | | | 1105 | | | | | | | | 1872 | | | | | | | | 1477 | | | | | | | | 2736 | | | | | | | | | 1058 | | | | | | | | | 1710 | | | | | | | | | 1332 | | | | | | | 1345 | | | | | | 1855 | | | | | | | | |  | |
| Hf | 11759 | | | 10181 | | | | | | | | 5269 | | | | | | | | 14344 | | | | | | | | 9475 | | | | | | | | 8655 | | | | | | | | | 9665 | | | | | | | | |  | | | | | | 8528 | | | | | | | 9279 | | | | | | | 8426 | | | | | | | | 10426 | | | | | | | | 8704 | | | | | | | | 9030 | | | | | | | | | 8934 | | | | | | | | | 11536 | | | | | | | | | 11321 | | | | | | | 11238 | | | | | | 10515 | | | | | | | | |  | |
| Nb | 8.90 | | | 1.40 | | | | | | | | 0.67 | | | | | | | | 2.21 | | | | | | | | 1.48 | | | | | | | | 2.35 | | | | | | | | | 2.45 | | | | | | | | |  | | | | | | 1.18 | | | | | | | 0.75 | | | | | | | 2.63 | | | | | | | | 4.44 | | | | | | | | 2.47 | | | | | | | | 5.92 | | | | | | | | | 3.91 | | | | | | | | | 2.09 | | | | | | | | | 3.83 | | | | | | | 1.84 | | | | | | 2.08 | | | | | | | | |  | |
| Ta | 11.79 | | | 0.60 | | | | | | | | 0.27 | | | | | | | | 1.33 | | | | | | | | 0.49 | | | | | | | | 1.41 | | | | | | | | | 1.10 | | | | | | | | |  | | | | | | 0.51 | | | | | | | 0.18 | | | | | | | 1.30 | | | | | | | | 1.42 | | | | | | | | 1.52 | | | | | | | | 1.53 | | | | | | | | | 1.59 | | | | | | | | | 1.37 | | | | | | | | | 1.09 | | | | | | | 1.01 | | | | | | 1.21 | | | | | | | | |  | |
| P |  | | |  | | | | | | | |  | | | | | | | |  | | | | | | | |  | | | | | | | |  | | | | | | | | |  | | | | | | | | |  | | | | | |  | | | | | | |  | | | | | | |  | | | | | | | |  | | | | | | | |  | | | | | | | |  | | | | | | | | |  | | | | | | | | |  | | | | | | | | |  | | | | | | |  | | | | | |  | | | | | | | | |  | |
| LREE | 18 | | | 17 | | | | | | | | 3 | | | | | | | | 114 | | | | | | | | 12 | | | | | | | | 96 | | | | | | | | | 17 | | | | | | | | |  | | | | | | 549 | | | | | | | 38 | | | | | | | 57 | | | | | | | | 78 | | | | | | | | 25 | | | | | | | | 60 | | | | | | | | | 54 | | | | | | | | | 809 | | | | | | | | | 703 | | | | | | | 47 | | | | | | 105 | | | | | | | | |  | |
| MREE | 130 | | | 329 | | | | | | | | 15 | | | | | | | | 570 | | | | | | | | 134 | | | | | | | | 835 | | | | | | | | | 101 | | | | | | | | |  | | | | | | 185 | | | | | | | 99 | | | | | | | 179 | | | | | | | | 267 | | | | | | | | 212 | | | | | | | | 425 | | | | | | | | | 159 | | | | | | | | | 553 | | | | | | | | | 413 | | | | | | | 170 | | | | | | 535 | | | | | | | | |  | |
| HREE | 1541 | | | 963 | | | | | | | | 114 | | | | | | | | 1131 | | | | | | | | 1122 | | | | | | | | 1359 | | | | | | | | | 673 | | | | | | | | |  | | | | | | 417 | | | | | | | 269 | | | | | | | 551 | | | | | | | | 998 | | | | | | | | 781 | | | | | | | | 1323 | | | | | | | | | 586 | | | | | | | | | 533 | | | | | | | | | 634 | | | | | | | 768 | | | | | | 887 | | | | | | | | |  | |
| (Yb/La)_n_ | 1440.6 | | | 1576.8 | | | | | | | | 833.0 | | | | | | | | 141.5 | | | | | | | | 7550.7 | | | | | | | | 286.6 | | | | | | | | | 2358.0 | | | | | | | | |  | | | | | | 2.2 | | | | | | | 147.8 | | | | | | | 3515.2 | | | | | | | | 126.7 | | | | | | | | 321.3 | | | | | | | | 223.2 | | | | | | | | | 59.4 | | | | | | | | | 7.3 | | | | | | | | | 4.2 | | | | | | | 199.5 | | | | | | 114.8 | | | | | | | | |  | |
| (Sm/La)_n_ | 6.8 | | | 62.7 | | | | | | | | 7.7 | | | | | | | | 19.0 | | | | | | | | 34.7 | | | | | | | | 27.8 | | | | | | | | | 16.4 | | | | | | | | |  | | | | | | 0.3 | | | | | | | 9.4 | | | | | | | 210.8 | | | | | | | | 4.6 | | | | | | | | 8.7 | | | | | | | | 8.1 | | | | | | | | | 2.0 | | | | | | | | | 4.1 | | | | | | | | | 1.1 | | | | | | | 4.9 | | | | | | 14.3 | | | | | | | | |  | |
| (Lu/Gd)_n_ | 106.5 | | | 10.4 | | | | | | | | 37.2 | | | | | | | | 5.6 | | | | | | | | 58.1 | | | | | | | | 4.9 | | | | | | | | | 41.1 | | | | | | | | |  | | | | | | 6.4 | | | | | | | 8.0 | | | | | | | 10.1 | | | | | | | | 14.9 | | | | | | | | 14.6 | | | | | | | | 10.7 | | | | | | | | | 14.7 | | | | | | | | | 2.4 | | | | | | | | | 4.2 | | | | | | | 21.2 | | | | | | 4.1 | | | | | | | | |  | |
| Eu/Eu* | 0.27 | | | 0.37 | | | | | | | | 0.60 | | | | | | | | 0.73 | | | | | | | | 0.20 | | | | | | | | 0.62 | | | | | | | | | 0.20 | | | | | | | | |  | | | | | | 0.19 | | | | | | | 0.17 | | | | | | | 0.16 | | | | | | | | 0.37 | | | | | | | | 0.21 | | | | | | | | 0.09 | | | | | | | | | 0.19 | | | | | | | | | 0.82 | | | | | | | | | 0.56 | | | | | | | 0.37 | | | | | | 0.66 | | | | | | | | |  | |
| Ce/Ce* | 4.78 | | | 1.28 | | | | | | | | 6.98 | | | | | | | | 1.04 | | | | | | | | 22.09 | | | | | | | | 1.71 | | | | | | | | | 21.62 | | | | | | | | |  | | | | | | 1.04 | | | | | | | 4.47 | | | | | | | 11.77 | | | | | | | | 1.94 | | | | | | | | 3.42 | | | | | | | | 4.52 | | | | | | | | | 1.49 | | | | | | | | | 2.10 | | | | | | | | | 1.26 | | | | | | | 2.14 | | | | | | 1.79 | | | | | | | | |  | |
| U/Yb | 0.363 | | | 0.376 | | | | | | | | 0.454 | | | | | | | | 0.214 | | | | | | | | 0.287 | | | | | | | | 1.060 | | | | | | | | | 0.443 | | | | | | | | |  | | | | | | 0.993 | | | | | | | 0.759 | | | | | | | 4.567 | | | | | | | | 0.899 | | | | | | | | 0.617 | | | | | | | | 0.906 | | | | | | | | | 1.068 | | | | | | | | | 6.160 | | | | | | | | | 1.653 | | | | | | | 0.901 | | | | | | 3.930 | | | | | | | | |  | |
| Nb/Yb | 0.010 | | | 0.003 | | | | | | | | 0.010 | | | | | | | | 0.004 | | | | | | | | 0.002 | | | | | | | | 0.004 | | | | | | | | | 0.007 | | | | | | | | |  | | | | | | 0.006 | | | | | | | 0.005 | | | | | | | 0.009 | | | | | | | | 0.009 | | | | | | | | 0.006 | | | | | | | | 0.009 | | | | | | | | | 0.013 | | | | | | | | | 0.008 | | | | | | | | | 0.012 | | | | | | | 0.004 | | | | | | 0.005 | | | | | | | | |  | |
|  |  | | |  | | | | | | | |  | | | | | | | |  | | | | | | | |  | | | | | | | |  | | | | | | | | |  | | | | | | | | |  | | | | | |  | | | | | | |  | | | | | | |  | | | | | | | |  | | | | | | | |  | | | | | | | |  | | | | | | | | |  | | | | | | | | |  | | | | | | | | |  | | | | | | |  | | | | | |  | | | | | | | | | | |
| Table S4 (continued) | | | | | | | | | | | | | | | | | | | | | | | | | | | | | | | | | | | | | | | | | | | | | | | | | | | | | | | | | | | | | | | | | | | | | | | | | | | | | | | | | | | | | | | | | | | | | | | | | | | | | | | | | | | | | | | | | | | | | | | | | | | | | | | | | | | | | | | | | | |  | | | | | | | | | |
| Sample | 05WD31 | | | | | | | | | | | | | | | | | | | | | | | | | | | | | | | | | | | | | | | | | | | | | | | | | | | | | | | | | | | | | | | | | | | | | | | | | | | | | | | | | | | | | | | | | | | | | | | | | | | | | | | | | | | | | | |  | | | | | | | | | | 05WD36 | | | | | | | | | | | | | | | | |  | | | | | | | | | |
| Spot | 1 | | | | 3 | | | | | | | | 4 | | | | | | | | | 5 | | | | | | | | | | 6 | | | | | | | | | 7 | | | | | | | | | 8 | | | | | | | | 9 | | | | | | | | | | 10 | | | | | | | | | 11 | | | | | | | | | 12 | | | | | | | | | 13 | | | | | | | | | 14 | | | | | | | |  | | | | | | | | | | 1 | | | | | | | | | 2 | | | | | | | |  | | | | | | | | | |
| La | 188.38 | | | | 0.21 | | | | | | | | 0.13 | | | | | | | | | 0.14 | | | | | | | | | | 0.11 | | | | | | | | | 3.56 | | | | | | | | | 4.40 | | | | | | | | 48.31 | | | | | | | | | | 2.66 | | | | | | | | | 0.32 | | | | | | | | | 1.70 | | | | | | | | | 1.15 | | | | | | | | | 0.11 | | | | | | | |  | | | | | | | | | | 0.05 | | | | | | | | | 29.09 | | | | | | | |  | | | | | | | | | |
| Ce | 115.19 | | | | 13.28 | | | | | | | | 13.27 | | | | | | | | | 19.31 | | | | | | | | | | 11.89 | | | | | | | | | 24.83 | | | | | | | | | 34.90 | | | | | | | | 93.91 | | | | | | | | | | 24.08 | | | | | | | | | 22.58 | | | | | | | | | 18.12 | | | | | | | | | 25.80 | | | | | | | | | 42.14 | | | | | | | |  | | | | | | | | | | 6.65 | | | | | | | | | 208.21 | | | | | | | |  | | | | | | | | | |
| Pr | 49.69 | | | | 0.07 | | | | | | | | 0.08 | | | | | | | | | 0.09 | | | | | | | | | | 0.11 | | | | | | | | | 0.76 | | | | | | | | | 4.88 | | | | | | | | 8.25 | | | | | | | | | | 2.06 | | | | | | | | | 0.12 | | | | | | | | | 7.91 | | | | | | | | | 0.27 | | | | | | | | | 0.39 | | | | | | | |  | | | | | | | | | | 0.01 | | | | | | | | | 32.06 | | | | | | | |  | | | | | | | | | |
| Nd | 249.41 | | | | 0.58 | | | | | | | | 1.01 | | | | | | | | | 1.18 | | | | | | | | | | 1.09 | | | | | | | | | 3.45 | | | | | | | | | 31.83 | | | | | | | | 29.51 | | | | | | | | | | 22.99 | | | | | | | | | 1.04 | | | | | | | | | 40.27 | | | | | | | | | 2.55 | | | | | | | | | 5.68 | | | | | | | |  | | | | | | | | | | 0.69 | | | | | | | | | 277.98 | | | | | | | |  | | | | | | | | | |
| Sm | 112.42 | | | | 1.82 | | | | | | | | 1.96 | | | | | | | | | 2.28 | | | | | | | | | | 1.56 | | | | | | | | | 3.04 | | | | | | | | | 25.32 | | | | | | | | 4.42 | | | | | | | | | | 22.91 | | | | | | | | | 2.96 | | | | | | | | | 48.45 | | | | | | | | | 4.16 | | | | | | | | | 8.30 | | | | | | | |  | | | | | | | | | | 0.94 | | | | | | | | | 108.20 | | | | | | | |  | | | | | | | | | |
| Eu | 11.02 | | | | 0.58 | | | | | | | | 0.45 | | | | | | | | | 0.56 | | | | | | | | | | 0.46 | | | | | | | | | 0.46 | | | | | | | | | 14.98 | | | | | | | | 0.80 | | | | | | | | | | 12.26 | | | | | | | | | 1.58 | | | | | | | | | 7.70 | | | | | | | | | 0.41 | | | | | | | | | 5.17 | | | | | | | |  | | | | | | | | | | 0.32 | | | | | | | | | 31.62 | | | | | | | |  | | | | | | | | | |
| Gd | 226.2 | | | | 7.3 | | | | | | | | 6.8 | | | | | | | | | 14.0 | | | | | | | | | | 10.9 | | | | | | | | | 20.5 | | | | | | | | | 52.8 | | | | | | | | 7.7 | | | | | | | | | | 26.5 | | | | | | | | | 13.9 | | | | | | | | | 105.7 | | | | | | | | | 19.0 | | | | | | | | | 38.7 | | | | | | | |  | | | | | | | | | | 5.2 | | | | | | | | | 114.8 | | | | | | | |  | | | | | | | | | |
| Tb | 74.17 | | | | 2.5 | | | | | | | | 2.3 | | | | | | | | | 5.25 | | | | | | | | | | 3.58 | | | | | | | | | 8.57 | | | | | | | | | 21.5 | | | | | | | | 2.44 | | | | | | | | | | 5.4 | | | | | | | | | 4.96 | | | | | | | | | 32.84 | | | | | | | | | 5.55 | | | | | | | | | 11.97 | | | | | | | |  | | | | | | | | | | 1.78 | | | | | | | | | 20.2 | | | | | | | |  | | | | | | | | | |
| Dy | 752.7 | | | | 25.2 | | | | | | | | 23.6 | | | | | | | | | 65.8 | | | | | | | | | | 42.6 | | | | | | | | | 116.4 | | | | | | | | | 246.6 | | | | | | | | 28.3 | | | | | | | | | | 48.2 | | | | | | | | | 61.4 | | | | | | | | | 316.9 | | | | | | | | | 61.8 | | | | | | | | | 124.7 | | | | | | | |  | | | | | | | | | | 22.3 | | | | | | | | | 158.5 | | | | | | | |  | | | | | | | | | |
| Ho | 231.4 | | | | 8.5 | | | | | | | | 8.5 | | | | | | | | | 26.7 | | | | | | | | | | 14.9 | | | | | | | | | 45.4 | | | | | | | | | 83.7 | | | | | | | | 12.0 | | | | | | | | | | 17.9 | | | | | | | | | 22.8 | | | | | | | | | 91.5 | | | | | | | | | 21.2 | | | | | | | | | 45.1 | | | | | | | |  | | | | | | | | | | 9.4 | | | | | | | | | 46.3 | | | | | | | |  | | | | | | | | | |
| Er | 1025.3 | | | | 35.2 | | | | | | | | 35.3 | | | | | | | | | 121.3 | | | | | | | | | | 60.9 | | | | | | | | | 207.8 | | | | | | | | | 404.0 | | | | | | | | 58.6 | | | | | | | | | | 83.0 | | | | | | | | | 100.9 | | | | | | | | | 343.5 | | | | | | | | | 87.0 | | | | | | | | | 198.3 | | | | | | | |  | | | | | | | | | | 48.4 | | | | | | | | | 201.1 | | | | | | | |  | | | | | | | | | |
| Tm | 241.2 | | | | 6.9 | | | | | | | | 7.5 | | | | | | | | | 26.6 | | | | | | | | | | 11.9 | | | | | | | | | 43.4 | | | | | | | | | 102.9 | | | | | | | | 14.0 | | | | | | | | | | 20.9 | | | | | | | | | 21.1 | | | | | | | | | 63.6 | | | | | | | | | 17.0 | | | | | | | | | 40.2 | | | | | | | |  | | | | | | | | | | 12.1 | | | | | | | | | 48.1 | | | | | | | |  | | | | | | | | | |
| Yb | 2575 | | | | 67 | | | | | | | | 70 | | | | | | | | | 272 | | | | | | | | | | 110 | | | | | | | | | 418 | | | | | | | | | 1203 | | | | | | | | 149 | | | | | | | | | | 223 | | | | | | | | | 197 | | | | | | | | | 554 | | | | | | | | | 157 | | | | | | | | | 390 | | | | | | | |  | | | | | | | | | | 137 | | | | | | | | | 519 | | | | | | | |  | | | | | | | | | |
| Lu | 448 | | | | 12 | | | | | | | | 13 | | | | | | | | | 50 | | | | | | | | | | 21 | | | | | | | | | 75 | | | | | | | | | 237 | | | | | | | | 30 | | | | | | | | | | 48 | | | | | | | | | 36 | | | | | | | | | 92 | | | | | | | | | 27 | | | | | | | | | 74 | | | | | | | |  | | | | | | | | | | 29 | | | | | | | | | 96 | | | | | | | |  | | | | | | | | | |
| Pb | 604 | | | | 10 | | | | | | | | 44 | | | | | | | | | 726 | | | | | | | | | | 101 | | | | | | | | | 1280 | | | | | | | | | 234 | | | | | | | | 308 | | | | | | | | | | 356 | | | | | | | | | 301 | | | | | | | | | 34 | | | | | | | | | 875 | | | | | | | | | 17 | | | | | | | |  | | | | | | | | | | 292 | | | | | | | | | 658 | | | | | | | |  | | | | | | | | | |
| Th | 4908 | | | | 64 | | | | | | | | 38 | | | | | | | | | 293 | | | | | | | | | | 13 | | | | | | | | | 558 | | | | | | | | | 130 | | | | | | | | 144 | | | | | | | | | | 36 | | | | | | | | | 124 | | | | | | | | | 853 | | | | | | | | | 295 | | | | | | | | | 53 | | | | | | | |  | | | | | | | | | | 47 | | | | | | | | | 194 | | | | | | | |  | | | | | | | | | |
| U | 3096 | | | | 60 | | | | | | | | 58 | | | | | | | | | 808 | | | | | | | | | | 111 | | | | | | | | | 1137 | | | | | | | | | 3351 | | | | | | | | 357 | | | | | | | | | | 223 | | | | | | | | | 243 | | | | | | | | | 278 | | | | | | | | | 427 | | | | | | | | | 35 | | | | | | | |  | | | | | | | | | | 125 | | | | | | | | | 1262 | | | | | | | |  | | | | | | | | | |
| Y | 6790 | | | | 253 | | | | | | | | 242 | | | | | | | | | 806 | | | | | | | | | | 429 | | | | | | | | | 1343 | | | | | | | | | 3303 | | | | | | | | 386 | | | | | | | | | | 584 | | | | | | | | | 660 | | | | | | | | | 2420 | | | | | | | | | 648 | | | | | | | | | 1404 | | | | | | | |  | | | | | | | | | | 316 | | | | | | | | | 1258 | | | | | | | |  | | | | | | | | | |
| Hf | 16181 | | | | 9536 | | | | | | | | 9714 | | | | | | | | | 10826 | | | | | | | | | | 10240 | | | | | | | | | 11199 | | | | | | | | | 12922 | | | | | | | | 11266 | | | | | | | | | | 8642 | | | | | | | | | 11413 | | | | | | | | | 8590 | | | | | | | | | 10630 | | | | | | | | | 7372 | | | | | | | |  | | | | | | | | | | 11981 | | | | | | | | | 13969 | | | | | | | |  | | | | | | | | | |
| Nb | 320.38 | | | | 1.30 | | | | | | | | 1.48 | | | | | | | | | 2.86 | | | | | | | | | | 1.88 | | | | | | | | | 9.44 | | | | | | | | | 25.98 | | | | | | | | 3.27 | | | | | | | | | | 1.47 | | | | | | | | | 2.39 | | | | | | | | | 7.26 | | | | | | | | | 2.50 | | | | | | | | | 1.65 | | | | | | | |  | | | | | | | | | | 0.76 | | | | | | | | | 16.89 | | | | | | | |  | | | | | | | | | |
| Ta | 228.37 | | | | 0.63 | | | | | | | | 0.66 | | | | | | | | | 1.67 | | | | | | | | | | 0.59 | | | | | | | | | 3.39 | | | | | | | | | 3.93 | | | | | | | | 1.42 | | | | | | | | | | 0.27 | | | | | | | | | 0.93 | | | | | | | | | 0.43 | | | | | | | | | 0.85 | | | | | | | | | 0.12 | | | | | | | |  | | | | | | | | | | 0.38 | | | | | | | | | 12.94 | | | | | | | |  | | | | | | | | | |
| P |  | | | |  | | | | | | | |  | | | | | | | | |  | | | | | | | | | |  | | | | | | | | |  | | | | | | | | |  | | | | | | | |  | | | | | | | | | |  | | | | | | | | |  | | | | | | | | |  | | | | | | | | |  | | | | | | | | |  | | | | | | | |  | | | | | | | | | |  | | | | | | | | |  | | | | | | | |  | | | | | | | | | |
| LREE | 603 | | | | 14 | | | | | | | | 14 | | | | | | | | | 21 | | | | | | | | | | 13 | | | | | | | | | 33 | | | | | | | | | 76 | | | | | | | | 180 | | | | | | | | | | 52 | | | | | | | | | 24 | | | | | | | | | 68 | | | | | | | | | 30 | | | | | | | | | 48 | | | | | | | |  | | | | | | | | | | 7 | | | | | | | | | 547 | | | | | | | |  | | | | | | | | | |
| MREE | 1177 | | | | 37 | | | | | | | | 35 | | | | | | | | | 88 | | | | | | | | | | 59 | | | | | | | | | 149 | | | | | | | | | 361 | | | | | | | | 44 | | | | | | | | | | 115 | | | | | | | | | 85 | | | | | | | | | 512 | | | | | | | | | 91 | | | | | | | | | 189 | | | | | | | |  | | | | | | | | | | 30 | | | | | | | | | 433 | | | | | | | |  | | | | | | | | | |
| HREE | 4521 | | | | 129 | | | | | | | | 134 | | | | | | | | | 497 | | | | | | | | | | 219 | | | | | | | | | 789 | | | | | | | | | 2030 | | | | | | | | 263 | | | | | | | | | | 393 | | | | | | | | | 378 | | | | | | | | | 1145 | | | | | | | | | 309 | | | | | | | | | 748 | | | | | | | |  | | | | | | | | | | 237 | | | | | | | | | 911 | | | | | | | |  | | | | | | | | | |
| (Yb/La)_n_ | 19.1 | | | | 442.9 | | | | | | | | 728.5 | | | | | | | | | 2752.6 | | | | | | | | | | 1409.7 | | | | | | | | | 163.8 | | | | | | | | | 381.0 | | | | | | | | 4.3 | | | | | | | | | | 116.9 | | | | | | | | | 859.3 | | | | | | | | | 454.3 | | | | | | | | | 190.6 | | | | | | | | | 4766.9 | | | | | | | |  | | | | | | | | | | 4072.0 | | | | | | | | | 24.9 | | | | | | | |  | | | | | | | | | |
| (Sm/La)_n_ | 0.9 | | | | 13.4 | | | | | | | | 22.8 | | | | | | | | | 25.6 | | | | | | | | | | 22.2 | | | | | | | | | 1.3 | | | | | | | | | 8.9 | | | | | | | | 0.1 | | | | | | | | | | 13.3 | | | | | | | | | 14.3 | | | | | | | | | 44.1 | | | | | | | | | 5.6 | | | | | | | | | 112.8 | | | | | | | |  | | | | | | | | | | 31.0 | | | | | | | | | 5.8 | | | | | | | |  | | | | | | | | | |
| (Lu/Gd)_n_ | 16.0 | | | | 13.6 | | | | | | | | 15.7 | | | | | | | | | 28.8 | | | | | | | | | | 15.7 | | | | | | | | | 29.4 | | | | | | | | | 36.3 | | | | | | | | 31.4 | | | | | | | | | | 14.7 | | | | | | | | | 20.7 | | | | | | | | | 7.1 | | | | | | | | | 11.3 | | | | | | | | | 15.5 | | | | | | | |  | | | | | | | | | | 46.2 | | | | | | | | | 6.7 | | | | | | | |  | | | | | | | | | |
| Eu/Eu* | 0.21 | | | | 0.42 | | | | | | | | 0.34 | | | | | | | | | 0.23 | | | | | | | | | | 0.25 | | | | | | | | | 0.13 | | | | | | | | | 1.22 | | | | | | | | 0.42 | | | | | | | | | | 1.52 | | | | | | | | | 0.62 | | | | | | | | | 0.32 | | | | | | | | | 0.12 | | | | | | | | | 0.74 | | | | | | | |  | | | | | | | | | | 0.35 | | | | | | | | | 0.86 | | | | | | | |  | | | | | | | | | |
| Ce/Ce* | 0.29 | | | | 26.23 | | | | | | | | 30.67 | | | | | | | | | 40.42 | | | | | | | | | | 23.41 | | | | | | | | | 3.52 | | | | | | | | | 1.63 | | | | | | | | 1.06 | | | | | | | | | | 2.39 | | | | | | | | | 28.82 | | | | | | | | | 0.65 | | | | | | | | | 10.99 | | | | | | | | | 30.03 | | | | | | | |  | | | | | | | | | | 74.16 | | | | | | | | | 1.48 | | | | | | | |  | | | | | | | | | |
| U/Yb | 1.202 | | | | 0.894 | | | | | | | | 0.837 | | | | | | | | | 2.966 | | | | | | | | | | 1.007 | | | | | | | | | 2.719 | | | | | | | | | 2.786 | | | | | | | | 2.402 | | | | | | | | | | 0.999 | | | | | | | | | 1.232 | | | | | | | | | 0.502 | | | | | | | | | 2.719 | | | | | | | | | 0.090 | | | | | | | |  | | | | | | | | | | 0.912 | | | | | | | | | 2.430 | | | | | | | |  | | | | | | | | | |
| Nb/Yb | 0.124 | | | | 0.019 | | | | | | | | 0.021 | | | | | | | | | 0.010 | | | | | | | | | | 0.017 | | | | | | | | | 0.023 | | | | | | | | | 0.022 | | | | | | | | 0.022 | | | | | | | | | | 0.007 | | | | | | | | | 0.012 | | | | | | | | | 0.013 | | | | | | | | | 0.016 | | | | | | | | | 0.004 | | | | | | | |  | | | | | | | | | | 0.006 | | | | | | | | | 0.033 | | | | | | | |  | | | | | | | | | |
|  |  | | | |  | | | | | | | |  | | | | | | | | |  | | | | | | | | | |  | | | | | | | | |  | | | | | | | | |  | | | | | | | |  | | | | | | | | | |  | | | | | | | | |  | | | | | | | | |  | | | | | | | | |  | | | | | | | | |  | | | | | | | |  | | | | | | | | | |  | | | | | | | | |  | | | | | | | |  | | | | | | | | | |
| Table S4 (continued) | | | | | | | | | | | | | | | | | | | | | | | | | | | | | | | | | | | | | | | | | | | | | | | | | | | | | | | | | | | | | | | | | | | | | | | | | | | | | | | | | | | | | | | | | | | | | | | | | | | | | | | | | | | | | | | | | | | | | | | | | | | | | | | | | | | | | | | | | | | | | | |  | | | | | |
| Sample | 05WD36 | | | | | | | | | | | | | | | | | | | | | | | | | | | | | | | | | | | | | | | | | | | | | | | | | | | | | | | | | | | | | | | | | | | | | | | | | | | | | | | | | | | | | | | | | | | | | | | | | | | | | | | | | | | | | | | | | | | | | | | | | | | | | | | | | | | | | | | | | | | | | |  | | | | | |
| Spot | 3 | | | 4 | | | | | | | | 5 | | | | | | | | | 6 | | | | | | | | | 7 | | | | | | | | 8 | | | | | | | | 9 | | | | | | | | | 10 | | | | | | | | 11 | | | | | | | | 12 | | | | | | | 13 | | | | | | | | 14 | | | | | | | | 15 | | | | | | | 16 | | | | | | | | | 17 | | | | | | | | | 18 | | | | | | | | 19 | | | | | | | | 20 | | | | | | | |  | | | | | |
| La | 0.16 | | | 25.25 | | | | | | | | 3.59 | | | | | | | | | 1.21 | | | | | | | | | 0.17 | | | | | | | | 6.33 | | | | | | | | 0.07 | | | | | | | | | 0.19 | | | | | | | | 10.90 | | | | | | | | 25.54 | | | | | | | 17.14 | | | | | | | | 0.45 | | | | | | | | 2.15 | | | | | | | 0.23 | | | | | | | | | 0.10 | | | | | | | | | 0.33 | | | | | | | | 1.58 | | | | | | | | 0.15 | | | | | | | |  | | | | | |
| Ce | 12.49 | | | 147.98 | | | | | | | | 17.44 | | | | | | | | | 10.09 | | | | | | | | | 3.12 | | | | | | | | 17.49 | | | | | | | | 9.96 | | | | | | | | | 7.41 | | | | | | | | 116.64 | | | | | | | | 218.72 | | | | | | | 43.26 | | | | | | | | 21.38 | | | | | | | | 26.62 | | | | | | | 13.40 | | | | | | | | | 43.01 | | | | | | | | | 15.67 | | | | | | | | 19.10 | | | | | | | | 36.48 | | | | | | | |  | | | | | |
| Pr | 0.09 | | | 11.81 | | | | | | | | 2.54 | | | | | | | | | 0.54 | | | | | | | | | 0.15 | | | | | | | | 1.37 | | | | | | | | 0.05 | | | | | | | | | 0.14 | | | | | | | | 5.40 | | | | | | | | 30.98 | | | | | | | 3.33 | | | | | | | | 0.60 | | | | | | | | 0.41 | | | | | | | 0.12 | | | | | | | | | 0.12 | | | | | | | | | 0.60 | | | | | | | | 1.01 | | | | | | | | 0.32 | | | | | | | |  | | | | | |
| Nd | 1.00 | | | 63.54 | | | | | | | | 20.72 | | | | | | | | | 4.05 | | | | | | | | | 2.72 | | | | | | | | 6.62 | | | | | | | | 1.15 | | | | | | | | | 1.70 | | | | | | | | 55.41 | | | | | | | | 221.42 | | | | | | | 18.18 | | | | | | | | 8.01 | | | | | | | | 2.49 | | | | | | | 1.82 | | | | | | | | | 2.52 | | | | | | | | | 5.07 | | | | | | | | 12.78 | | | | | | | | 5.62 | | | | | | | |  | | | | | |
| Sm | 2.69 | | | 15.99 | | | | | | | | 17.97 | | | | | | | | | 3.62 | | | | | | | | | 4.27 | | | | | | | | 4.46 | | | | | | | | 2.74 | | | | | | | | | 2.83 | | | | | | | | 45.33 | | | | | | | | 82.02 | | | | | | | 11.43 | | | | | | | | 7.40 | | | | | | | | 2.41 | | | | | | | 3.20 | | | | | | | | | 7.32 | | | | | | | | | 4.83 | | | | | | | | 9.84 | | | | | | | | 8.49 | | | | | | | |  | | | | | |
| Eu | 0.24 | | | 6.52 | | | | | | | | 4.45 | | | | | | | | | 1.51 | | | | | | | | | 0.61 | | | | | | | | 4.25 | | | | | | | | 0.57 | | | | | | | | | 0.69 | | | | | | | | 35.39 | | | | | | | | 17.44 | | | | | | | 1.29 | | | | | | | | 3.27 | | | | | | | | 0.26 | | | | | | | 0.49 | | | | | | | | | 0.48 | | | | | | | | | 0.79 | | | | | | | | 3.76 | | | | | | | | 2.78 | | | | | | | |  | | | | | |
| Gd | 15.6 | | | 33.5 | | | | | | | | 34.1 | | | | | | | | | 14.5 | | | | | | | | | 20.8 | | | | | | | | 30.6 | | | | | | | | 14.6 | | | | | | | | | 13.0 | | | | | | | | 92.4 | | | | | | | | 91.2 | | | | | | | 42.2 | | | | | | | | 27.4 | | | | | | | | 10.6 | | | | | | | 15.6 | | | | | | | | | 36.8 | | | | | | | | | 21.2 | | | | | | | | 23.7 | | | | | | | | 42.4 | | | | | | | |  | | | | | |
| Tb | 6.79 | | | 9.46 | | | | | | | | 5.57 | | | | | | | | | 5.11 | | | | | | | | | 8.56 | | | | | | | | 11.19 | | | | | | | | 5.43 | | | | | | | | | 4.45 | | | | | | | | 15.09 | | | | | | | | 17.04 | | | | | | | 15.23 | | | | | | | | 8.23 | | | | | | | | 4.48 | | | | | | | 5.51 | | | | | | | | | 13.9 | | | | | | | | | 6.49 | | | | | | | | 6.04 | | | | | | | | 13.74 | | | | | | | |  | | | | | |
| Dy | 90.8 | | | 109.3 | | | | | | | | 43.4 | | | | | | | | | 59.7 | | | | | | | | | 98.1 | | | | | | | | 127.6 | | | | | | | | 67.6 | | | | | | | | | 54.0 | | | | | | | | 140.6 | | | | | | | | 138.4 | | | | | | | 170.5 | | | | | | | | 85.1 | | | | | | | | 54.8 | | | | | | | 64.0 | | | | | | | | | 185.1 | | | | | | | | | 62.5 | | | | | | | | 59.8 | | | | | | | | 159.3 | | | | | | | |  | | | | | |
| Ho | 37.1 | | | 40.3 | | | | | | | | 14.6 | | | | | | | | | 22.4 | | | | | | | | | 39.6 | | | | | | | | 46.8 | | | | | | | | 26.6 | | | | | | | | | 21.0 | | | | | | | | 47.6 | | | | | | | | 44.1 | | | | | | | 60.4 | | | | | | | | 30.4 | | | | | | | | 21.2 | | | | | | | 23.1 | | | | | | | | | 74.0 | | | | | | | | | 22.9 | | | | | | | | 21.8 | | | | | | | | 59.1 | | | | | | | |  | | | | | |
| Er | 173.7 | | | 193.0 | | | | | | | | 64.7 | | | | | | | | | 104.0 | | | | | | | | | 181.0 | | | | | | | | 198.9 | | | | | | | | 122.7 | | | | | | | | | 95.6 | | | | | | | | 197.6 | | | | | | | | 180.5 | | | | | | | 262.5 | | | | | | | | 127.0 | | | | | | | | 94.8 | | | | | | | 97.4 | | | | | | | | | 344.8 | | | | | | | | | 96.1 | | | | | | | | 91.8 | | | | | | | | 267.8 | | | | | | | |  | | | | | |
| Tm | 38.3 | | | 45.0 | | | | | | | | 16.7 | | | | | | | | | 22.9 | | | | | | | | | 38.9 | | | | | | | | 42.4 | | | | | | | | 27.0 | | | | | | | | | 21.2 | | | | | | | | 40.9 | | | | | | | | 37.0 | | | | | | | 54.2 | | | | | | | | 25.7 | | | | | | | | 19.8 | | | | | | | 20.8 | | | | | | | | | 74.0 | | | | | | | | | 20.0 | | | | | | | | 19.6 | | | | | | | | 58.5 | | | | | | | |  | | | | | |
| Yb | 368 | | | 507 | | | | | | | | 190 | | | | | | | | | 229 | | | | | | | | | 377 | | | | | | | | 402 | | | | | | | | 272 | | | | | | | | | 216 | | | | | | | | 391 | | | | | | | | 362 | | | | | | | 516 | | | | | | | | 239 | | | | | | | | 190 | | | | | | | 204 | | | | | | | | | 719 | | | | | | | | | 183 | | | | | | | | 194 | | | | | | | | 583 | | | | | | | |  | | | | | |
| Lu | 66 | | | 115 | | | | | | | | 41 | | | | | | | | | 48 | | | | | | | | | 74 | | | | | | | | 74 | | | | | | | | 52 | | | | | | | | | 46 | | | | | | | | 70 | | | | | | | | 69 | | | | | | | 89 | | | | | | | | 45 | | | | | | | | 37 | | | | | | | 38 | | | | | | | | | 132 | | | | | | | | | 35 | | | | | | | | 38 | | | | | | | | 114 | | | | | | | |  | | | | | |
| Pb | 1353 | | | 135 | | | | | | | | 563 | | | | | | | | | 486 | | | | | | | | | 38 | | | | | | | | 362 | | | | | | | | 263 | | | | | | | | | 241 | | | | | | | | 738 | | | | | | | | 580 | | | | | | | 51 | | | | | | | | 462 | | | | | | | | 364 | | | | | | | 403 | | | | | | | | | 140 | | | | | | | | | 333 | | | | | | | | 460 | | | | | | | | 2051 | | | | | | | |  | | | | | |
| Th | 217 | | | 571 | | | | | | | | 49 | | | | | | | | | 94 | | | | | | | | | 93 | | | | | | | | 94 | | | | | | | | 55 | | | | | | | | | 53 | | | | | | | | 375 | | | | | | | | 455 | | | | | | | 72 | | | | | | | | 121 | | | | | | | | 96 | | | | | | | 91 | | | | | | | | | 246 | | | | | | | | | 81 | | | | | | | | 89 | | | | | | | | 668 | | | | | | | |  | | | | | |
| U | 1019 | | | 1431 | | | | | | | | 371 | | | | | | | | | 237 | | | | | | | | | 203 | | | | | | | | 262 | | | | | | | | 105 | | | | | | | | | 94 | | | | | | | | 566 | | | | | | | | 452 | | | | | | | 57 | | | | | | | | 208 | | | | | | | | 364 | | | | | | | 184 | | | | | | | | | 302 | | | | | | | | | 148 | | | | | | | | 240 | | | | | | | | 1077 | | | | | | | |  | | | | | |
| Y | 1119 | | | 1340 | | | | | | | | 510 | | | | | | | | | 722 | | | | | | | | | 1143 | | | | | | | | 1383 | | | | | | | | 800 | | | | | | | | | 649 | | | | | | | | 1437 | | | | | | | | 1362 | | | | | | | 1736 | | | | | | | | 889 | | | | | | | | 652 | | | | | | | 685 | | | | | | | | | 2208 | | | | | | | | | 673 | | | | | | | | 649 | | | | | | | | 1902 | | | | | | | |  | | | | | |
| Hf | 11437 | | | 13599 | | | | | | | | 13977 | | | | | | | | | 8942 | | | | | | | | | 8057 | | | | | | | | 8580 | | | | | | | | 8609 | | | | | | | | | 9818 | | | | | | | | 9565 | | | | | | | | 9674 | | | | | | | 11906 | | | | | | | | 9872 | | | | | | | | 11399 | | | | | | | 10301 | | | | | | | | | 11968 | | | | | | | | | 9132 | | | | | | | | 10842 | | | | | | | | 9905 | | | | | | | |  | | | | | |
| Nb | 4.97 | | | 4.41 | | | | | | | | 1.18 | | | | | | | | | 1.87 | | | | | | | | | 1.45 | | | | | | | | 1.88 | | | | | | | | 1.08 | | | | | | | | | 0.64 | | | | | | | | 6.82 | | | | | | | | 3.63 | | | | | | | 3.87 | | | | | | | | 1.78 | | | | | | | | 2.00 | | | | | | | 1.45 | | | | | | | | | 7.48 | | | | | | | | | 1.81 | | | | | | | | 1.55 | | | | | | | | 5.18 | | | | | | | |  | | | | | |
| Ta | 3.81 | | | 1.17 | | | | | | | | 1.35 | | | | | | | | | 0.46 | | | | | | | | | 0.54 | | | | | | | | 0.65 | | | | | | | | 0.50 | | | | | | | | | 0.28 | | | | | | | | 1.59 | | | | | | | | 0.58 | | | | | | | 1.12 | | | | | | | | 0.64 | | | | | | | | 0.77 | | | | | | | 0.81 | | | | | | | | | 1.65 | | | | | | | | | 0.74 | | | | | | | | 0.73 | | | | | | | | 1.65 | | | | | | | |  | | | | | |
| P |  | | |  | | | | | | | |  | | | | | | | | |  | | | | | | | | |  | | | | | | | |  | | | | | | | |  | | | | | | | | |  | | | | | | | |  | | | | | | | |  | | | | | | |  | | | | | | | |  | | | | | | | |  | | | | | | |  | | | | | | | | |  | | | | | | | | |  | | | | | | | |  | | | | | | | |  | | | | | | | |  | | | | | |
| LREE | 14 | | | 249 | | | | | | | | 44 | | | | | | | | | 16 | | | | | | | | | 6 | | | | | | | | 32 | | | | | | | | 11 | | | | | | | | | 9 | | | | | | | | 188 | | | | | | | | 497 | | | | | | | 82 | | | | | | | | 30 | | | | | | | | 32 | | | | | | | 16 | | | | | | | | | 46 | | | | | | | | | 22 | | | | | | | | 34 | | | | | | | | 43 | | | | | | | |  | | | | | |
| MREE | 116 | | | 175 | | | | | | | | 105 | | | | | | | | | 84 | | | | | | | | | 132 | | | | | | | | 178 | | | | | | | | 91 | | | | | | | | | 75 | | | | | | | | 329 | | | | | | | | 346 | | | | | | | 241 | | | | | | | | 131 | | | | | | | | 73 | | | | | | | 89 | | | | | | | | | 244 | | | | | | | | | 96 | | | | | | | | 103 | | | | | | | | 227 | | | | | | | |  | | | | | |
| HREE | 684 | | | 900 | | | | | | | | 327 | | | | | | | | | 426 | | | | | | | | | 710 | | | | | | | | 764 | | | | | | | | 501 | | | | | | | | | 400 | | | | | | | | 747 | | | | | | | | 693 | | | | | | | 982 | | | | | | | | 467 | | | | | | | | 362 | | | | | | | 384 | | | | | | | | | 1343 | | | | | | | | | 358 | | | | | | | | 366 | | | | | | | | 1082 | | | | | | | |  | | | | | |
| (Yb/La)_n_ | 3270.8 | | | 28.0 | | | | | | | | 73.7 | | | | | | | | | 263.5 | | | | | | | | | 3089.5 | | | | | | | | 88.6 | | | | | | | | 5189.9 | | | | | | | | | 1588.0 | | | | | | | | 50.0 | | | | | | | | 19.8 | | | | | | | 41.9 | | | | | | | | 740.6 | | | | | | | | 123.1 | | | | | | | 1238.5 | | | | | | | | | 10122.0 | | | | | | | | | 774.8 | | | | | | | | 171.5 | | | | | | | | 5308.8 | | | | | | | |  | | | | | |
| (Sm/La)_n_ | 26.5 | | | 1.0 | | | | | | | | 7.8 | | | | | | | | | 4.6 | | | | | | | | | 38.9 | | | | | | | | 1.1 | | | | | | | | 58.1 | | | | | | | | | 23.1 | | | | | | | | 6.4 | | | | | | | | 5.0 | | | | | | | 1.0 | | | | | | | | 25.5 | | | | | | | | 1.7 | | | | | | | 21.6 | | | | | | | | | 114.5 | | | | | | | | | 22.7 | | | | | | | | 9.6 | | | | | | | | 86.0 | | | | | | | |  | | | | | |
| (Lu/Gd)_n_ | 34.5 | | | 27.8 | | | | | | | | 9.8 | | | | | | | | | 26.8 | | | | | | | | | 28.8 | | | | | | | | 19.6 | | | | | | | | 29.0 | | | | | | | | | 28.5 | | | | | | | | 6.2 | | | | | | | | 6.1 | | | | | | | 17.1 | | | | | | | | 13.4 | | | | | | | | 28.0 | | | | | | | 19.7 | | | | | | | | | 28.9 | | | | | | | | | 13.5 | | | | | | | | 13.0 | | | | | | | | 21.8 | | | | | | | |  | | | | | |
| Eu/Eu* | 0.09 | | | 0.84 | | | | | | | | 0.54 | | | | | | | | | 0.55 | | | | | | | | | 0.16 | | | | | | | | 0.82 | | | | | | | | 0.22 | | | | | | | | | 0.29 | | | | | | | | 1.64 | | | | | | | | 0.61 | | | | | | | 0.16 | | | | | | | | 0.62 | | | | | | | | 0.13 | | | | | | | 0.17 | | | | | | | | | 0.07 | | | | | | | | | 0.20 | | | | | | | | 0.72 | | | | | | | | 0.37 | | | | | | | |  | | | | | |
| Ce/Ce* | 25.03 | | | 2.09 | | | | | | | | 1.36 | | | | | | | | | 3.07 | | | | | | | | | 4.54 | | | | | | | | 1.39 | | | | | | | | 39.51 | | | | | | | | | 10.64 | | | | | | | | 3.71 | | | | | | | | 1.65 | | | | | | | 1.32 | | | | | | | | 8.51 | | | | | | | | 6.50 | | | | | | | 19.70 | | | | | | | | | 82.08 | | | | | | | | | 6.64 | | | | | | | | 3.61 | | | | | | | | 29.70 | | | | | | | |  | | | | | |
| U/Yb | 2.766 | | | 2.822 | | | | | | | | 1.958 | | | | | | | | | 1.034 | | | | | | | | | 0.539 | | | | | | | | 0.652 | | | | | | | | 0.386 | | | | | | | | | 0.436 | | | | | | | | 1.449 | | | | | | | | 1.249 | | | | | | | 0.110 | | | | | | | | 0.872 | | | | | | | | 1.918 | | | | | | | 0.899 | | | | | | | | | 0.420 | | | | | | | | | 0.807 | | | | | | | | 1.235 | | | | | | | | 1.849 | | | | | | | |  | | | | | |
| Nb/Yb | 0.013 | | | 0.009 | | | | | | | | 0.006 | | | | | | | | | 0.008 | | | | | | | | | 0.004 | | | | | | | | 0.005 | | | | | | | | 0.004 | | | | | | | | | 0.003 | | | | | | | | 0.017 | | | | | | | | 0.010 | | | | | | | 0.008 | | | | | | | | 0.007 | | | | | | | | 0.011 | | | | | | | 0.007 | | | | | | | | | 0.010 | | | | | | | | | 0.010 | | | | | | | | 0.008 | | | | | | | | 0.009 | | | | | | | |  | | | | | |
|  |  | | |  | | | | | | | |  | | | | | | | | |  | | | | | | | | |  | | | | | | | |  | | | | | | | |  | | | | | | | | |  | | | | | | | |  | | | | | | | |  | | | | | | |  | | | | | | | |  | | | | | | | |  | | | | | | |  | | | | | | | | |  | | | | | | | | |  | | | | | | | |  | | | | | | | |  | | | | | | | |  | | | | | |
| Table S4 (continued) | | | | | | | | | | | | | | | | | | | | | | | | | | | | | | | | | | | | | | | | | | | | | | | | | | | | | | | | | | | | | | | | | | | | | | | | | | | | | | | | | | | | | | | | | | | | | | | | | | | | | | | | | | | | | | | | | | | | | | | | | | | | | | | | | | | | | | | | | | | | | | | | | | | |  |
| Sample | | | 05WD36 | | | | | | | | | | | | | | | | | | | | | | | | | | | | | | | | | | | | | | | | |  | | | | | | | | | 06SW09 | | | | | | | | | | | | | | | | | | | | | | | | | | | | | | | | | | | | | | | | | | | | | | | | | | | | | | | | | | | | | | | | | | | | | | | | | | | | | | | | | | | | | | | | | | | | | | |  |
| Spot | | | 21 | | | | | | 22 | | | | | | | 23 | | | | | | | | | 24 | | | | | | | | | | 25 | | | | | | | | |  | | | | | | | | | 1 | | | | | | | | | 2 | | | | | | | | | 3 | | | | | | | | 4 | | | | | | | | 5 | | | | | | | | | 6 | | | | | | | | | 7 | | | | | | | | | | 8 | | | | | | | | 9 | | | | | | | | | | 10 | | | | | | | 11 | | | | | | | |  |
| La | | | 3.44 | | | | | | 123.92 | | | | | | | 0.76 | | | | | | | | | 0.84 | | | | | | | | | | 1.93 | | | | | | | | |  | | | | | | | | | 0.03 | | | | | | | | | 0.60 | | | | | | | | | 259.82 | | | | | | | | 29.56 | | | | | | | | 0.24 | | | | | | | | | 8.85 | | | | | | | | | 4.59 | | | | | | | | | | 18.72 | | | | | | | | 0.76 | | | | | | | | | | 6.60 | | | | | | | 3.96 | | | | | | | |  |
| Ce | | | 32.23 | | | | | | 423.47 | | | | | | | 21.61 | | | | | | | | | 8.60 | | | | | | | | | | 58.69 | | | | | | | | |  | | | | | | | | | 11.47 | | | | | | | | | 9.43 | | | | | | | | | 800.03 | | | | | | | | 92.39 | | | | | | | | 16.99 | | | | | | | | | 52.12 | | | | | | | | | 63.29 | | | | | | | | | | 127.83 | | | | | | | | 14.25 | | | | | | | | | | 18.65 | | | | | | | 25.02 | | | | | | | |  |
| Pr | | | 2.12 | | | | | | 39.47 | | | | | | | 0.75 | | | | | | | | | 0.28 | | | | | | | | | | 1.25 | | | | | | | | |  | | | | | | | | | 0.05 | | | | | | | | | 0.07 | | | | | | | | | 122.88 | | | | | | | | 11.68 | | | | | | | | 0.40 | | | | | | | | | 2.94 | | | | | | | | | 2.07 | | | | | | | | | | 5.80 | | | | | | | | 0.29 | | | | | | | | | | 2.13 | | | | | | | 1.35 | | | | | | | |  |
| Nd | | | 14.12 | | | | | | 266.17 | | | | | | | 7.02 | | | | | | | | | 1.96 | | | | | | | | | | 8.32 | | | | | | | | |  | | | | | | | | | 0.76 | | | | | | | | | 0.70 | | | | | | | | | 678.44 | | | | | | | | 63.31 | | | | | | | | 6.74 | | | | | | | | | 18.57 | | | | | | | | | 16.71 | | | | | | | | | | 32.78 | | | | | | | | 2.27 | | | | | | | | | | 16.13 | | | | | | | 8.21 | | | | | | | |  |
| Sm | | | 12.66 | | | | | | 85.11 | | | | | | | 6.62 | | | | | | | | | 2.79 | | | | | | | | | | 8.06 | | | | | | | | |  | | | | | | | | | 2.58 | | | | | | | | | 2.00 | | | | | | | | | 217.69 | | | | | | | | 23.48 | | | | | | | | 12.92 | | | | | | | | | 17.63 | | | | | | | | | 19.94 | | | | | | | | | | 17.84 | | | | | | | | 2.42 | | | | | | | | | | 12.41 | | | | | | | 6.01 | | | | | | | |  |
| Eu | | | 2.85 | | | | | | 20.36 | | | | | | | 3.01 | | | | | | | | | 0.38 | | | | | | | | | | 2.99 | | | | | | | | |  | | | | | | | | | 0.11 | | | | | | | | | 0.08 | | | | | | | | | 1.82 | | | | | | | | 1.06 | | | | | | | | 0.40 | | | | | | | | | 0.35 | | | | | | | | | 1.44 | | | | | | | | | | 0.85 | | | | | | | | 0.32 | | | | | | | | | | 2.53 | | | | | | | 0.32 | | | | | | | |  |
| Gd | | | 27.2 | | | | | | 108.4 | | | | | | | 25.6 | | | | | | | | | 19.4 | | | | | | | | | | 29.5 | | | | | | | | |  | | | | | | | | | 15.0 | | | | | | | | | 15.2 | | | | | | | | | 282.5 | | | | | | | | 57.8 | | | | | | | | 59.8 | | | | | | | | | 77.6 | | | | | | | | | 91.2 | | | | | | | | | | 78.4 | | | | | | | | 11.4 | | | | | | | | | | 38.0 | | | | | | | 29.0 | | | | | | | |  |
| Tb | | | 6.57 | | | | | | 21.5 | | | | | | | 7.35 | | | | | | | | | 9.02 | | | | | | | | | | 8.81 | | | | | | | | |  | | | | | | | | | 5.74 | | | | | | | | | 5.98 | | | | | | | | | 56.78 | | | | | | | | 17.03 | | | | | | | | 18.78 | | | | | | | | | 27.73 | | | | | | | | | 32.73 | | | | | | | | | | 29.67 | | | | | | | | 4.44 | | | | | | | | | | 9.93 | | | | | | | 10.55 | | | | | | | |  |
| Dy | | | 63.8 | | | | | | 186.7 | | | | | | | 79.9 | | | | | | | | | 123.8 | | | | | | | | | | 100.3 | | | | | | | | |  | | | | | | | | | 67.5 | | | | | | | | | 78.3 | | | | | | | | | 462.5 | | | | | | | | 184.0 | | | | | | | | 207.0 | | | | | | | | | 324.8 | | | | | | | | | 371.6 | | | | | | | | | | 352.0 | | | | | | | | 55.9 | | | | | | | | | | 98.3 | | | | | | | 127.2 | | | | | | | |  |
| Ho | | | 24.9 | | | | | | 58.9 | | | | | | | 28.3 | | | | | | | | | 47.5 | | | | | | | | | | 37.1 | | | | | | | | |  | | | | | | | | | 25.9 | | | | | | | | | 31.0 | | | | | | | | | 135.9 | | | | | | | | 66.9 | | | | | | | | 73.8 | | | | | | | | | 120.4 | | | | | | | | | 136.4 | | | | | | | | | | 130.5 | | | | | | | | 22.7 | | | | | | | | | | 32.5 | | | | | | | 47.8 | | | | | | | |  |
| Er | | | 115.2 | | | | | | 237.7 | | | | | | | 118.0 | | | | | | | | | 208.0 | | | | | | | | | | 180.5 | | | | | | | | |  | | | | | | | | | 112.1 | | | | | | | | | 135.0 | | | | | | | | | 479.3 | | | | | | | | 271.7 | | | | | | | | 291.6 | | | | | | | | | 487.9 | | | | | | | | | 570.8 | | | | | | | | | | 532.0 | | | | | | | | 104.7 | | | | | | | | | | 128.7 | | | | | | | 203.6 | | | | | | | |  |
| Tm | | | 25.7 | | | | | | 49.2 | | | | | | | 23.3 | | | | | | | | | 42.6 | | | | | | | | | | 41.7 | | | | | | | | |  | | | | | | | | | 24.3 | | | | | | | | | 29.3 | | | | | | | | | 84.8 | | | | | | | | 53.6 | | | | | | | | 56.8 | | | | | | | | | 95.8 | | | | | | | | | 118.6 | | | | | | | | | | 104.3 | | | | | | | | 24.2 | | | | | | | | | | 25.7 | | | | | | | 42.6 | | | | | | | |  |
| Yb | | | 276 | | | | | | 472 | | | | | | | 227 | | | | | | | | | 398 | | | | | | | | | | 452 | | | | | | | | |  | | | | | | | | | 242 | | | | | | | | | 279 | | | | | | | | | 683 | | | | | | | | 479 | | | | | | | | 504 | | | | | | | | | 835 | | | | | | | | | 1091 | | | | | | | | | | 887 | | | | | | | | 247 | | | | | | | | | | 246 | | | | | | | 395 | | | | | | | |  |
| Lu | | | 56 | | | | | | 88 | | | | | | | 45 | | | | | | | | | 71 | | | | | | | | | | 101 | | | | | | | | |  | | | | | | | | | 47 | | | | | | | | | 48 | | | | | | | | | 103 | | | | | | | | 79 | | | | | | | | 83 | | | | | | | | | 135 | | | | | | | | | 179 | | | | | | | | | | 136 | | | | | | | | 43 | | | | | | | | | | 45 | | | | | | | 67 | | | | | | | |  |
| Pb | | | 736 | | | | | | 816 | | | | | | | 254 | | | | | | | | | 2041 | | | | | | | | | | 95 | | | | | | | | |  | | | | | | | | | 65 | | | | | | | | | 58 | | | | | | | | | 110 | | | | | | | | 75 | | | | | | | | 42 | | | | | | | | | 157 | | | | | | | | | 246 | | | | | | | | | | 373 | | | | | | | | 53 | | | | | | | | | | 26 | | | | | | | 132 | | | | | | | |  |
| Th | | | 90 | | | | | | 753 | | | | | | | 43 | | | | | | | | | 166 | | | | | | | | | | 437 | | | | | | | | |  | | | | | | | | | 38 | | | | | | | | | 44 | | | | | | | | | 208 | | | | | | | | 92 | | | | | | | | 74 | | | | | | | | | 296 | | | | | | | | | 618 | | | | | | | | | | 762 | | | | | | | | 47 | | | | | | | | | | 27 | | | | | | | 120 | | | | | | | |  |
| U | | | 506 | | | | | | 1526 | | | | | | | 114 | | | | | | | | | 1736 | | | | | | | | | | 807 | | | | | | | | |  | | | | | | | | | 264 | | | | | | | | | 135 | | | | | | | | | 169 | | | | | | | | 111 | | | | | | | | 62 | | | | | | | | | 255 | | | | | | | | | 373 | | | | | | | | | | 585 | | | | | | | | 130 | | | | | | | | | | 116 | | | | | | | 220 | | | | | | | |  |
| Y | | | 801 | | | | | | 1805 | | | | | | | 837 | | | | | | | | | 1541 | | | | | | | | | | 1254 | | | | | | | | |  | | | | | | | | | 684 | | | | | | | | | 796 | | | | | | | | | 3181 | | | | | | | | 1673 | | | | | | | | 1824 | | | | | | | | | 3054 | | | | | | | | | 3659 | | | | | | | | | | 3413 | | | | | | | | 639 | | | | | | | | | | 822 | | | | | | | 1214 | | | | | | | |  |
| Hf | | | 12306 | | | | | | 10671 | | | | | | | 8714 | | | | | | | | | 12954 | | | | | | | | | | 11138 | | | | | | | | |  | | | | | | | | | 8331 | | | | | | | | | 9141 | | | | | | | | | 7504 | | | | | | | | 8081 | | | | | | | | 7067 | | | | | | | | | 7543 | | | | | | | | | 7876 | | | | | | | | | | 8255 | | | | | | | | 7288 | | | | | | | | | | 5358 | | | | | | | 8228 | | | | | | | |  |
| Nb | | | 1.62 | | | | | | 10.91 | | | | | | | 0.90 | | | | | | | | | 6.16 | | | | | | | | | | 3.25 | | | | | | | | |  | | | | | | | | | 7.42 | | | | | | | | | 7.98 | | | | | | | | | 11.19 | | | | | | | | 3.97 | | | | | | | | 1.67 | | | | | | | | | 13.24 | | | | | | | | | 7.67 | | | | | | | | | | 53.62 | | | | | | | | 11.04 | | | | | | | | | | 7.93 | | | | | | | 12.90 | | | | | | | |  |
| Ta | | | 1.72 | | | | | | 1.30 | | | | | | | 0.26 | | | | | | | | | 4.18 | | | | | | | | | | 0.70 | | | | | | | | |  | | | | | | | | | 2.25 | | | | | | | | | 1.96 | | | | | | | | | 1.42 | | | | | | | | 0.89 | | | | | | | | 0.41 | | | | | | | | | 1.46 | | | | | | | | | 1.53 | | | | | | | | | | 7.16 | | | | | | | | 2.61 | | | | | | | | | | 1.49 | | | | | | | 2.85 | | | | | | | |  |
| P | | |  | | | | | |  | | | | | | |  | | | | | | | | |  | | | | | | | | | |  | | | | | | | | |  | | | | | | | | | 135 | | | | | | | | | 150 | | | | | | | | | 1005 | | | | | | | | 940 | | | | | | | | 200 | | | | | | | | | 242 | | | | | | | | | 896 | | | | | | | | | | 426 | | | | | | | | 191 | | | | | | | | | | 1800 | | | | | | | 266 | | | | | | | |  |
| LREE | | | 52 | | | | | | 853 | | | | | | | 30 | | | | | | | | | 12 | | | | | | | | | | 70 | | | | | | | | |  | | | | | | | | | 12 | | | | | | | | | 11 | | | | | | | | | 1861 | | | | | | | | 197 | | | | | | | | 24 | | | | | | | | | 82 | | | | | | | | | 87 | | | | | | | | | | 185 | | | | | | | | 18 | | | | | | | | | | 44 | | | | | | | 39 | | | | | | | |  |
| MREE | | | 113 | | | | | | 422 | | | | | | | 123 | | | | | | | | | 155 | | | | | | | | | | 150 | | | | | | | | |  | | | | | | | | | 91 | | | | | | | | | 102 | | | | | | | | | 1021 | | | | | | | | 283 | | | | | | | | 299 | | | | | | | | | 448 | | | | | | | | | 517 | | | | | | | | | | 479 | | | | | | | | 74 | | | | | | | | | | 161 | | | | | | | 173 | | | | | | | |  |
| HREE | | | 498 | | | | | | 907 | | | | | | | 441 | | | | | | | | | 767 | | | | | | | | | | 813 | | | | | | | | |  | | | | | | | | | 451 | | | | | | | | | 523 | | | | | | | | | 1486 | | | | | | | | 950 | | | | | | | | 1010 | | | | | | | | | 1674 | | | | | | | | | 2095 | | | | | | | | | | 1790 | | | | | | | | 442 | | | | | | | | | | 477 | | | | | | | 756 | | | | | | | |  |
| (Yb/La)_n_ | | | 112.0 | | | | | | 5.3 | | | | | | | 415.7 | | | | | | | | | 660.2 | | | | | | | | | | 326.6 | | | | | | | | |  | | | | | | | | | 9903.6 | | | | | | | | | 652.9 | | | | | | | | | 3.7 | | | | | | | | 22.6 | | | | | | | | 2892.4 | | | | | | | | | 131.5 | | | | | | | | | 331.3 | | | | | | | | | | 66.1 | | | | | | | | 454.9 | | | | | | | | | | 51.9 | | | | | | | 139.0 | | | | | | | |  |
| (Sm/La)_n_ | | | 5.7 | | | | | | 1.1 | | | | | | | 13.5 | | | | | | | | | 5.1 | | | | | | | | | | 6.5 | | | | | | | | |  | | | | | | | | | 117.5 | | | | | | | | | 5.2 | | | | | | | | | 1.3 | | | | | | | | 1.2 | | | | | | | | 82.4 | | | | | | | | | 3.1 | | | | | | | | | 6.7 | | | | | | | | | | 1.5 | | | | | | | | 5.0 | | | | | | | | | | 2.9 | | | | | | | 2.4 | | | | | | | |  |
| (Lu/Gd)_n_ | | | 16.7 | | | | | | 6.6 | | | | | | | 14.2 | | | | | | | | | 29.9 | | | | | | | | | | 27.8 | | | | | | | | |  | | | | | | | | | 25.3 | | | | | | | | | 25.8 | | | | | | | | | 3.0 | | | | | | | | 11.1 | | | | | | | | 11.3 | | | | | | | | | 14.1 | | | | | | | | | 15.8 | | | | | | | | | | 14.1 | | | | | | | | 30.9 | | | | | | | | | | 9.5 | | | | | | | 18.7 | | | | | | | |  |
| Eu/Eu* | | | 0.46 | | | | | | 0.65 | | | | | | | 0.62 | | | | | | | | | 0.12 | | | | | | | | | | 0.53 | | | | | | | | |  | | | | | | | | | 0.04 | | | | | | | | | 0.03 | | | | | | | | | 0.02 | | | | | | | | 0.08 | | | | | | | | 0.04 | | | | | | | | | 0.02 | | | | | | | | | 0.09 | | | | | | | | | | 0.06 | | | | | | | | 0.15 | | | | | | | | | | 0.33 | | | | | | | 0.06 | | | | | | | |  |
| Ce/Ce* | | | 2.86 | | | | | | 1.47 | | | | | | | 6.36 | | | | | | | | | 4.33 | | | | | | | | | | 9.00 | | | | | | | | |  | | | | | | | | | 54.26 | | | | | | | | | 9.39 | | | | | | | | | 1.09 | | | | | | | | 1.22 | | | | | | | | 10.52 | | | | | | | | | 2.49 | | | | | | | | | 5.02 | | | | | | | | | | 2.98 | | | | | | | | 7.45 | | | | | | | | | | 1.21 | | | | | | | 2.64 | | | | | | | |  |
| U/Yb | | | 1.829 | | | | | | 3.232 | | | | | | | 0.503 | | | | | | | | | 4.365 | | | | | | | | | | 1.784 | | | | | | | | |  | | | | | | | | | 1.095 | | | | | | | | | 0.483 | | | | | | | | | 0.247 | | | | | | | | 0.231 | | | | | | | | 0.123 | | | | | | | | | 0.306 | | | | | | | | | 0.342 | | | | | | | | | | 0.659 | | | | | | | | 0.526 | | | | | | | | | | 0.472 | | | | | | | 0.557 | | | | | | | |  |
| Nb/Yb | | | 0.006 | | | | | | 0.023 | | | | | | | 0.004 | | | | | | | | | 0.015 | | | | | | | | | | 0.007 | | | | | | | | |  | | | | | | | | | 0.031 | | | | | | | | | 0.029 | | | | | | | | | 0.016 | | | | | | | | 0.008 | | | | | | | | 0.003 | | | | | | | | | 0.016 | | | | | | | | | 0.007 | | | | | | | | | | 0.060 | | | | | | | | 0.045 | | | | | | | | | | 0.032 | | | | | | | 0.033 | | | | | | | |  |
|  | | |  | | | | | |  | | | | | | |  | | | | | | | | |  | | | | | | | | | |  | | | | | | | | |  | | | | | | | | |  | | | | | | | | |  | | | | | | | | |  | | | | | | | |  | | | | | | | |  | | | | | | | | |  | | | | | | | | |  | | | | | | | | | |  | | | | | | | |  | | | | | | | | | |  | | | | | | |  | | | | | | | | |
| Table S4 (continued) | | | | | | | | | | | | | | | | | | | | | | | | | | | | | | | | | | | | | | | | | | | | | | | | | | | | | | | | | | | | | | | | | | | | | | | | | | | | | | | | | | | | | | | | | | | | | | | | | | | | | | | | | | | | | | | | | | | | | | | | | | | | | | | | | | | | | | | | | | | | | |  | | | | | | |
| Sample | | 06SW09 | | | | | | | | | | | | | | | | | | | | | | | | | | | | | | | | | | | | | | | | | | | | | | | | | | | | | | | | | | | | | | | | | | | | | | |  | | | | | | | | | | 06SW11 | | | | | | | | | | | | | | | | | | | | | | | | | | | | | | | | | | | | | | | | | | | | | | | | | | | | | | | | | | |  | | | | | | |
| Spot | | 12 | | | | | 13 | | | | | | | | | | | 14 | | | | | | | | | 15 | | | | | | | | | | 17 | | | | | | | | | | 18 | | | | | | | | | 19 | | | | | | | | | 20 | | | | | | | |  | | | | | | | | | | 1 | | | | | | | | | 2 | | | | | | | | | | | 3 | | | | | | | | | | 4 | | | | | | | | | | | 5 | | | | | | | | | | 6 | | | | | | | |  | | | | | | |
| La | | 0.11 | | | | | 20.42 | | | | | | | | | | | 0.09 | | | | | | | | | 4.72 | | | | | | | | | | 0.13 | | | | | | | | | | 9.47 | | | | | | | | | 0.03 | | | | | | | | | 0.83 | | | | | | | |  | | | | | | | | | | 12.35 | | | | | | | | | 3.75 | | | | | | | | | | | 0.29 | | | | | | | | | | 1.19 | | | | | | | | | | | 14.60 | | | | | | | | | | 59.19 | | | | | | | |  | | | | | | |
| Ce | | 15.81 | | | | | 74.78 | | | | | | | | | | | 16.35 | | | | | | | | | 71.26 | | | | | | | | | | 68.30 | | | | | | | | | | 43.53 | | | | | | | | | 21.22 | | | | | | | | | 29.44 | | | | | | | |  | | | | | | | | | | 50.96 | | | | | | | | | 36.68 | | | | | | | | | | | 8.57 | | | | | | | | | | 15.00 | | | | | | | | | | | 43.51 | | | | | | | | | | 161.74 | | | | | | | |  | | | | | | |
| Pr | | 0.12 | | | | | 7.51 | | | | | | | | | | | 0.22 | | | | | | | | | 2.50 | | | | | | | | | | 0.20 | | | | | | | | | | 4.16 | | | | | | | | | 0.11 | | | | | | | | | 0.20 | | | | | | | |  | | | | | | | | | | 6.67 | | | | | | | | | 2.33 | | | | | | | | | | | 0.21 | | | | | | | | | | 0.47 | | | | | | | | | | | 4.62 | | | | | | | | | | 19.91 | | | | | | | |  | | | | | | |
| Nd | | 2.09 | | | | | 47.93 | | | | | | | | | | | 4.11 | | | | | | | | | 14.84 | | | | | | | | | | 3.11 | | | | | | | | | | 23.87 | | | | | | | | | 2.10 | | | | | | | | | 2.55 | | | | | | | |  | | | | | | | | | | 40.12 | | | | | | | | | 14.97 | | | | | | | | | | | 1.53 | | | | | | | | | | 2.75 | | | | | | | | | | | 22.10 | | | | | | | | | | 102.38 | | | | | | | |  | | | | | | |
| Sm | | 4.23 | | | | | 33.38 | | | | | | | | | | | 7.28 | | | | | | | | | 12.62 | | | | | | | | | | 9.67 | | | | | | | | | | 11.46 | | | | | | | | | 6.91 | | | | | | | | | 6.44 | | | | | | | |  | | | | | | | | | | 18.22 | | | | | | | | | 9.75 | | | | | | | | | | | 2.39 | | | | | | | | | | 3.17 | | | | | | | | | | | 14.94 | | | | | | | | | | 34.87 | | | | | | | |  | | | | | | |
| Eu | | 1.06 | | | | | 0.69 | | | | | | | | | | | 0.30 | | | | | | | | | 0.68 | | | | | | | | | | 0.07 | | | | | | | | | | 0.45 | | | | | | | | | 0.07 | | | | | | | | | 0.10 | | | | | | | |  | | | | | | | | | | 1.91 | | | | | | | | | 0.42 | | | | | | | | | | | 0.32 | | | | | | | | | | 0.17 | | | | | | | | | | | 7.52 | | | | | | | | | | 2.31 | | | | | | | |  | | | | | | |
| Gd | | 23.9 | | | | | 101.3 | | | | | | | | | | | 34.3 | | | | | | | | | 56.9 | | | | | | | | | | 67.6 | | | | | | | | | | 33.1 | | | | | | | | | 42.5 | | | | | | | | | 41.7 | | | | | | | |  | | | | | | | | | | 45.9 | | | | | | | | | 45.1 | | | | | | | | | | | 14.0 | | | | | | | | | | 16.9 | | | | | | | | | | | 65.2 | | | | | | | | | | 73.2 | | | | | | | |  | | | | | | |
| Tb | | 8.85 | | | | | 32.27 | | | | | | | | | | | 11.2 | | | | | | | | | 21.08 | | | | | | | | | | 26.49 | | | | | | | | | | 9.98 | | | | | | | | | 15.55 | | | | | | | | | 15.36 | | | | | | | |  | | | | | | | | | | 13.81 | | | | | | | | | 18.79 | | | | | | | | | | | 5.28 | | | | | | | | | | 7.43 | | | | | | | | | | | 20.11 | | | | | | | | | | 21.02 | | | | | | | |  | | | | | | |
| Dy | | 106.6 | | | | | 344.5 | | | | | | | | | | | 121.8 | | | | | | | | | 248.2 | | | | | | | | | | 312.7 | | | | | | | | | | 109.8 | | | | | | | | | 179.6 | | | | | | | | | 177.6 | | | | | | | |  | | | | | | | | | | 153.8 | | | | | | | | | 247.1 | | | | | | | | | | | 67.6 | | | | | | | | | | 101.0 | | | | | | | | | | | 196.0 | | | | | | | | | | 222.4 | | | | | | | |  | | | | | | |
| Ho | | 42.2 | | | | | 118.6 | | | | | | | | | | | 43.7 | | | | | | | | | 90.5 | | | | | | | | | | 113.8 | | | | | | | | | | 38.9 | | | | | | | | | 65.1 | | | | | | | | | 64.4 | | | | | | | |  | | | | | | | | | | 55.1 | | | | | | | | | 102.1 | | | | | | | | | | | 27.6 | | | | | | | | | | 42.5 | | | | | | | | | | | 63.3 | | | | | | | | | | 79.1 | | | | | | | |  | | | | | | |
| Er | | 192.0 | | | | | 460.4 | | | | | | | | | | | 176.6 | | | | | | | | | 367.6 | | | | | | | | | | 445.7 | | | | | | | | | | 156.8 | | | | | | | | | 254.5 | | | | | | | | | 251.6 | | | | | | | |  | | | | | | | | | | 232.8 | | | | | | | | | 472.6 | | | | | | | | | | | 129.3 | | | | | | | | | | 201.3 | | | | | | | | | | | 244.1 | | | | | | | | | | 322.3 | | | | | | | |  | | | | | | |
| Tm | | 44.4 | | | | | 86.2 | | | | | | | | | | | 35.9 | | | | | | | | | 72.9 | | | | | | | | | | 85.5 | | | | | | | | | | 32.6 | | | | | | | | | 49.7 | | | | | | | | | 48.9 | | | | | | | |  | | | | | | | | | | 50.6 | | | | | | | | | 107.4 | | | | | | | | | | | 30.3 | | | | | | | | | | 49.3 | | | | | | | | | | | 50.0 | | | | | | | | | | 66.6 | | | | | | | |  | | | | | | |
| Yb | | 450 | | | | | 720 | | | | | | | | | | | 328 | | | | | | | | | 638 | | | | | | | | | | 713 | | | | | | | | | | 305 | | | | | | | | | 439 | | | | | | | | | 425 | | | | | | | |  | | | | | | | | | | 480 | | | | | | | | | 1053 | | | | | | | | | | | 306 | | | | | | | | | | 506 | | | | | | | | | | | 447 | | | | | | | | | | 613 | | | | | | | |  | | | | | | |
| Lu | | 81 | | | | | 114 | | | | | | | | | | | 55 | | | | | | | | | 98 | | | | | | | | | | 107 | | | | | | | | | | 48 | | | | | | | | | 67 | | | | | | | | | 63 | | | | | | | |  | | | | | | | | | | 77 | | | | | | | | | 178 | | | | | | | | | | | 55 | | | | | | | | | | 79 | | | | | | | | | | | 70 | | | | | | | | | | 102 | | | | | | | |  | | | | | | |
| Pb | | 36 | | | | | 127 | | | | | | | | | | | 34 | | | | | | | | | 216 | | | | | | | | | | 251 | | | | | | | | | | 40 | | | | | | | | | 58 | | | | | | | | | 69 | | | | | | | |  | | | | | | | | | | 67 | | | | | | | | | 430 | | | | | | | | | | | 73 | | | | | | | | | | 220 | | | | | | | | | | | 288 | | | | | | | | | | 96 | | | | | | | |  | | | | | | |
| Th | | 75 | | | | | 311 | | | | | | | | | | | 54 | | | | | | | | | 532 | | | | | | | | | | 437 | | | | | | | | | | 52 | | | | | | | | | 74 | | | | | | | | | 115 | | | | | | | |  | | | | | | | | | | 90 | | | | | | | | | 472 | | | | | | | | | | | 57 | | | | | | | | | | 206 | | | | | | | | | | | 195 | | | | | | | | | | 185 | | | | | | | |  | | | | | | |
| U | | 126 | | | | | 246 | | | | | | | | | | | 46 | | | | | | | | | 405 | | | | | | | | | | 338 | | | | | | | | | | 69 | | | | | | | | | 82 | | | | | | | | | 126 | | | | | | | |  | | | | | | | | | | 127 | | | | | | | | | 766 | | | | | | | | | | | 94 | | | | | | | | | | 526 | | | | | | | | | | | 237 | | | | | | | | | | 152 | | | | | | | |  | | | | | | |
| Y | | 1168 | | | | | 3030 | | | | | | | | | | | 1117 | | | | | | | | | 2329 | | | | | | | | | | 2837 | | | | | | | | | | 979 | | | | | | | | | 1594 | | | | | | | | | 1550 | | | | | | | |  | | | | | | | | | | 1477 | | | | | | | | | 2871 | | | | | | | | | | | 800 | | | | | | | | | | 1192 | | | | | | | | | | | 1605 | | | | | | | | | | 2083 | | | | | | | |  | | | | | | |
| Hf | | 7909 | | | | | 6774 | | | | | | | | | | | 6575 | | | | | | | | | 7612 | | | | | | | | | | 7151 | | | | | | | | | | 6233 | | | | | | | | | 6217 | | | | | | | | | 6575 | | | | | | | |  | | | | | | | | | | 8054 | | | | | | | | | 11553 | | | | | | | | | | | 7628 | | | | | | | | | | 10224 | | | | | | | | | | | 6550 | | | | | | | | | | 7093 | | | | | | | |  | | | | | | |
| Nb | | 10.07 | | | | | 9.11 | | | | | | | | | | | 1.85 | | | | | | | | | 31.32 | | | | | | | | | | 32.38 | | | | | | | | | | 4.75 | | | | | | | | | 3.67 | | | | | | | | | 9.28 | | | | | | | |  | | | | | | | | | | 3.89 | | | | | | | | | 37.38 | | | | | | | | | | | 4.09 | | | | | | | | | | 18.76 | | | | | | | | | | | 21.93 | | | | | | | | | | 3.84 | | | | | | | |  | | | | | | |
| Ta | | 0.83 | | | | | 1.25 | | | | | | | | | | | 0.39 | | | | | | | | | 4.42 | | | | | | | | | | 4.21 | | | | | | | | | | 0.75 | | | | | | | | | 0.64 | | | | | | | | | 1.55 | | | | | | | |  | | | | | | | | | | 0.84 | | | | | | | | | 7.16 | | | | | | | | | | | 2.03 | | | | | | | | | | 4.61 | | | | | | | | | | | 1.08 | | | | | | | | | | 0.75 | | | | | | | |  | | | | | | |
| P | | 577 | | | | | 245 | | | | | | | | | | | 167 | | | | | | | | | 204 | | | | | | | | | | 315 | | | | | | | | | | 756 | | | | | | | | | 165 | | | | | | | | | 255 | | | | | | | |  | | | | | | | | | | 1713 | | | | | | | | | 838 | | | | | | | | | | | 207 | | | | | | | | | | 386 | | | | | | | | | | | 354 | | | | | | | | | | 3654 | | | | | | | |  | | | | | | |
| LREE | | 18 | | | | | 151 | | | | | | | | | | | 21 | | | | | | | | | 93 | | | | | | | | | | 72 | | | | | | | | | | 81 | | | | | | | | | 23 | | | | | | | | | 33 | | | | | | | |  | | | | | | | | | | 110 | | | | | | | | | 58 | | | | | | | | | | | 11 | | | | | | | | | | 19 | | | | | | | | | | | 85 | | | | | | | | | | 343 | | | | | | | |  | | | | | | |
| MREE | | 145 | | | | | 512 | | | | | | | | | | | 175 | | | | | | | | | 340 | | | | | | | | | | 417 | | | | | | | | | | 165 | | | | | | | | | 245 | | | | | | | | | 241 | | | | | | | |  | | | | | | | | | | 234 | | | | | | | | | 321 | | | | | | | | | | | 90 | | | | | | | | | | 129 | | | | | | | | | | | 304 | | | | | | | | | | 354 | | | | | | | |  | | | | | | |
| HREE | | 810 | | | | | 1500 | | | | | | | | | | | 639 | | | | | | | | | 1266 | | | | | | | | | | 1464 | | | | | | | | | | 582 | | | | | | | | | 876 | | | | | | | | | 853 | | | | | | | |  | | | | | | | | | | 896 | | | | | | | | | 1913 | | | | | | | | | | | 548 | | | | | | | | | | 879 | | | | | | | | | | | 875 | | | | | | | | | | 1183 | | | | | | | |  | | | | | | |
| (Yb/La)_n_ | | 5916.3 | | | | | 49.2 | | | | | | | | | | | 5021.4 | | | | | | | | | 188.3 | | | | | | | | | | 7413.5 | | | | | | | | | | 45.0 | | | | | | | | | 19749.7 | | | | | | | | | 712.6 | | | | | | | |  | | | | | | | | | | 54.2 | | | | | | | | | 391.4 | | | | | | | | | | | 1495.9 | | | | | | | | | | 593.2 | | | | | | | | | | | 42.7 | | | | | | | | | | 14.4 | | | | | | | |  | | | | | | |
| (Sm/La)_n_ | | 61.8 | | | | | 2.5 | | | | | | | | | | | 123.9 | | | | | | | | | 4.1 | | | | | | | | | | 111.8 | | | | | | | | | | 1.9 | | | | | | | | | 345.3 | | | | | | | | | 12.0 | | | | | | | |  | | | | | | | | | | 2.3 | | | | | | | | | 4.0 | | | | | | | | | | | 13.0 | | | | | | | | | | 4.1 | | | | | | | | | | | 1.6 | | | | | | | | | | 0.9 | | | | | | | |  | | | | | | |
| (Lu/Gd)_n_ | | 27.5 | | | | | 9.1 | | | | | | | | | | | 12.9 | | | | | | | | | 13.9 | | | | | | | | | | 12.8 | | | | | | | | | | 11.8 | | | | | | | | | 12.8 | | | | | | | | | 12.2 | | | | | | | |  | | | | | | | | | | 13.5 | | | | | | | | | 31.9 | | | | | | | | | | | 31.9 | | | | | | | | | | 38.1 | | | | | | | | | | | 8.7 | | | | | | | | | | 11.3 | | | | | | | |  | | | | | | |
| Eu/Eu* | | 0.25 | | | | | 0.03 | | | | | | | | | | | 0.05 | | | | | | | | | 0.07 | | | | | | | | | | 0.01 | | | | | | | | | | 0.07 | | | | | | | | | 0.01 | | | | | | | | | 0.01 | | | | | | | |  | | | | | | | | | | 0.19 | | | | | | | | | 0.05 | | | | | | | | | | | 0.13 | | | | | | | | | | 0.06 | | | | | | | | | | | 0.62 | | | | | | | | | | 0.14 | | | | | | | |  | | | | | | |
| Ce/Ce* | | 30.58 | | | | | 1.48 | | | | | | | | | | | 20.10 | | | | | | | | | 5.04 | | | | | | | | | | 83.25 | | | | | | | | | | 1.70 | | | | | | | | | 52.11 | | | | | | | | | 17.05 | | | | | | | |  | | | | | | | | | | 1.36 | | | | | | | | | 2.97 | | | | | | | | | | | 8.13 | | | | | | | | | | 4.94 | | | | | | | | | | | 1.29 | | | | | | | | | | 1.15 | | | | | | | |  | | | | | | |
| U/Yb | | 0.280 | | | | | 0.342 | | | | | | | | | | | 0.140 | | | | | | | | | 0.634 | | | | | | | | | | 0.474 | | | | | | | | | | 0.228 | | | | | | | | | 0.186 | | | | | | | | | 0.296 | | | | | | | |  | | | | | | | | | | 0.264 | | | | | | | | | 0.727 | | | | | | | | | | | 0.308 | | | | | | | | | | 1.040 | | | | | | | | | | | 0.530 | | | | | | | | | | 0.247 | | | | | | | |  | | | | | | |
| Nb/Yb | | 0.022 | | | | | 0.013 | | | | | | | | | | | 0.006 | | | | | | | | | 0.049 | | | | | | | | | | 0.045 | | | | | | | | | | 0.016 | | | | | | | | | 0.008 | | | | | | | | | 0.022 | | | | | | | |  | | | | | | | | | | 0.008 | | | | | | | | | 0.036 | | | | | | | | | | | 0.013 | | | | | | | | | | 0.037 | | | | | | | | | | | 0.049 | | | | | | | | | | 0.006 | | | | | | | |  | | | | | | |
|  | |  | | | | |  | | | | | | | | | | |  | | | | | | | | |  | | | | | | | | | |  | | | | | | | | | |  | | | | | | | | |  | | | | | | | | |  | | | | | | | |  | | | | | | | | | |  | | | | | | | | |  | | | | | | | | | | |  | | | | | | | | | |  | | | | | | | | | | |  | | | | | | | | | |  | | | | | | | |  | | | | | | |
| Table S4 (continued) | | | | | | | | | | | | | | | | | | | | | | | | | | | | | | | | | | | | | | | | | | | | | | | | | | | | | | | | | | | | | | | | | | | | | | | | | | | | | | | | | | | | | | | | | | | | | | | | | | | | | | | | | | | | | | | | | | | | | | | | | | | | | | | | | | | | | | | | | | | | |  | | | | | | | |
| Sample | | 06SW11 | | | | | | | | | | | | | | | | | | | | | | | | | | | | | | | | | | | | | | | | | | | | | | | | | | | | | | | | | | | | | | | | | | | | | | | | | | | | | | | | | | | | | | | | | | | | | | | | | | | | | | | | | | | | | | | | | | | | | | | | | | | | | | | | | | | | | | | | | | |  | | | | | | | |
| Spot | | 7 | | | | | | | | 8 | | | | | | | | | 9 | | | | | | | | | | 10 | | | | | | | | | | 11 | | | | | | | | | | 12 | | | | | | | | | 13 | | | | | | | | | | | | 14 | | | | | | | | | | 15 | | | | | | | | | 16 | | | | | | | | | | 17 | | | | | | | | | 18 | | | | | | | | | | | | 19 | | | | | | | | | | 20 | | | | | | | | | | |  | | | | | | | |
| La | | 5.04 | | | | | | | | 5.14 | | | | | | | | | 0.33 | | | | | | | | | | 0.16 | | | | | | | | | | 0.53 | | | | | | | | | | 2.38 | | | | | | | | | 1.28 | | | | | | | | | | | | 15.46 | | | | | | | | | | 0.09 | | | | | | | | | 0.65 | | | | | | | | | | 0.06 | | | | | | | | | 3.98 | | | | | | | | | | | | 1.88 | | | | | | | | | | 0.27 | | | | | | | | | | |  | | | | | | | |
| Ce | | 31.16 | | | | | | | | 29.03 | | | | | | | | | 32.60 | | | | | | | | | | 2.17 | | | | | | | | | | 19.22 | | | | | | | | | | 26.07 | | | | | | | | | 30.78 | | | | | | | | | | | | 61.32 | | | | | | | | | | 9.08 | | | | | | | | | 95.04 | | | | | | | | | | 12.99 | | | | | | | | | 32.17 | | | | | | | | | | | | 41.84 | | | | | | | | | | 19.10 | | | | | | | | | | |  | | | | | | | |
| Pr | | 1.99 | | | | | | | | 1.16 | | | | | | | | | 0.23 | | | | | | | | | | 0.15 | | | | | | | | | | 0.75 | | | | | | | | | | 0.72 | | | | | | | | | 0.18 | | | | | | | | | | | | 7.32 | | | | | | | | | | 0.12 | | | | | | | | | 0.86 | | | | | | | | | | 0.05 | | | | | | | | | 1.28 | | | | | | | | | | | | 1.35 | | | | | | | | | | 0.20 | | | | | | | | | | |  | | | | | | | |
| Nd | | 12.04 | | | | | | | | 7.28 | | | | | | | | | 2.99 | | | | | | | | | | 3.22 | | | | | | | | | | 10.45 | | | | | | | | | | 5.87 | | | | | | | | | 1.48 | | | | | | | | | | | | 40.45 | | | | | | | | | | 0.75 | | | | | | | | | 7.94 | | | | | | | | | | 0.54 | | | | | | | | | 8.62 | | | | | | | | | | | | 9.60 | | | | | | | | | | 1.44 | | | | | | | | | | |  | | | | | | | |
| Sm | | 7.94 | | | | | | | | 6.40 | | | | | | | | | 5.75 | | | | | | | | | | 7.09 | | | | | | | | | | 18.90 | | | | | | | | | | 8.00 | | | | | | | | | 2.04 | | | | | | | | | | | | 14.72 | | | | | | | | | | 1.07 | | | | | | | | | 12.90 | | | | | | | | | | 1.41 | | | | | | | | | 8.69 | | | | | | | | | | | | 9.53 | | | | | | | | | | 1.39 | | | | | | | | | | |  | | | | | | | |
| Eu | | 0.36 | | | | | | | | 0.35 | | | | | | | | | 0.65 | | | | | | | | | | 0.20 | | | | | | | | | | 2.78 | | | | | | | | | | 0.95 | | | | | | | | | 0.40 | | | | | | | | | | | | 1.24 | | | | | | | | | | 0.29 | | | | | | | | | 0.89 | | | | | | | | | | 0.36 | | | | | | | | | 0.88 | | | | | | | | | | | | 0.56 | | | | | | | | | | 0.32 | | | | | | | | | | |  | | | | | | | |
| Gd | | 39.9 | | | | | | | | 25.4 | | | | | | | | | 34.1 | | | | | | | | | | 39.6 | | | | | | | | | | 84.3 | | | | | | | | | | 43.3 | | | | | | | | | 8.4 | | | | | | | | | | | | 35.9 | | | | | | | | | | 4.0 | | | | | | | | | 62.8 | | | | | | | | | | 7.2 | | | | | | | | | 41.5 | | | | | | | | | | | | 48.2 | | | | | | | | | | 7.4 | | | | | | | | | | |  | | | | | | | |
| Tb | | 16.23 | | | | | | | | 8.79 | | | | | | | | | 13.6 | | | | | | | | | | 14.05 | | | | | | | | | | 28.25 | | | | | | | | | | 16.13 | | | | | | | | | 3.12 | | | | | | | | | | | | 12.29 | | | | | | | | | | 1.48 | | | | | | | | | 23.72 | | | | | | | | | | 2.51 | | | | | | | | | 14.75 | | | | | | | | | | | | 19.51 | | | | | | | | | | 2.88 | | | | | | | | | | |  | | | | | | | |
| Dy | | 212.5 | | | | | | | | 99.8 | | | | | | | | | 170.5 | | | | | | | | | | 168.5 | | | | | | | | | | 306.7 | | | | | | | | | | 200.5 | | | | | | | | | 35.7 | | | | | | | | | | | | 154.1 | | | | | | | | | | 17.8 | | | | | | | | | 284.4 | | | | | | | | | | 32.8 | | | | | | | | | 174.7 | | | | | | | | | | | | 254.9 | | | | | | | | | | 35.3 | | | | | | | | | | |  | | | | | | | |
| Ho | | 88.0 | | | | | | | | 36.9 | | | | | | | | | 69.4 | | | | | | | | | | 64.6 | | | | | | | | | | 108.7 | | | | | | | | | | 76.7 | | | | | | | | | 12.5 | | | | | | | | | | | | 60.8 | | | | | | | | | | 7.3 | | | | | | | | | 107.6 | | | | | | | | | | 14.0 | | | | | | | | | 66.8 | | | | | | | | | | | | 102.5 | | | | | | | | | | 14.0 | | | | | | | | | | |  | | | | | | | |
| Er | | 407.2 | | | | | | | | 153.1 | | | | | | | | | 318.9 | | | | | | | | | | 278.0 | | | | | | | | | | 436.2 | | | | | | | | | | 324.5 | | | | | | | | | 55.3 | | | | | | | | | | | | 278.7 | | | | | | | | | | 35.9 | | | | | | | | | 466.2 | | | | | | | | | | 68.3 | | | | | | | | | 292.6 | | | | | | | | | | | | 456.4 | | | | | | | | | | 66.7 | | | | | | | | | | |  | | | | | | | |
| Tm | | 93.4 | | | | | | | | 33.0 | | | | | | | | | 72.3 | | | | | | | | | | 60.5 | | | | | | | | | | 92.3 | | | | | | | | | | 71.4 | | | | | | | | | 12.7 | | | | | | | | | | | | 66.3 | | | | | | | | | | 9.2 | | | | | | | | | 106.6 | | | | | | | | | | 18.3 | | | | | | | | | 61.9 | | | | | | | | | | | | 106.3 | | | | | | | | | | 17.5 | | | | | | | | | | |  | | | | | | | |
| Yb | | 911 | | | | | | | | 311 | | | | | | | | | 712 | | | | | | | | | | 571 | | | | | | | | | | 860 | | | | | | | | | | 672 | | | | | | | | | 128 | | | | | | | | | | | | 671 | | | | | | | | | | 107 | | | | | | | | | 1043 | | | | | | | | | | 208 | | | | | | | | | 585 | | | | | | | | | | | | 1048 | | | | | | | | | | 201 | | | | | | | | | | |  | | | | | | | |
| Lu | | 148 | | | | | | | | 50 | | | | | | | | | 116 | | | | | | | | | | 93 | | | | | | | | | | 134 | | | | | | | | | | 109 | | | | | | | | | 21 | | | | | | | | | | | | 106 | | | | | | | | | | 19 | | | | | | | | | 161 | | | | | | | | | | 37 | | | | | | | | | 108 | | | | | | | | | | | | 162 | | | | | | | | | | 32 | | | | | | | | | | |  | | | | | | | |
| Pb | | 298 | | | | | | | | 125 | | | | | | | | | 207 | | | | | | | | | | 58 | | | | | | | | | | 90 | | | | | | | | | | 126 | | | | | | | | | 198 | | | | | | | | | | | | 236 | | | | | | | | | | 44 | | | | | | | | | 387 | | | | | | | | | | 57 | | | | | | | | | 124 | | | | | | | | | | | | 402 | | | | | | | | | | 74 | | | | | | | | | | |  | | | | | | | |
| Th | | 316 | | | | | | | | 68 | | | | | | | | | 322 | | | | | | | | | | 82 | | | | | | | | | | 167 | | | | | | | | | | 190 | | | | | | | | | 83 | | | | | | | | | | | | 227 | | | | | | | | | | 46 | | | | | | | | | 916 | | | | | | | | | | 59 | | | | | | | | | 179 | | | | | | | | | | | | 487 | | | | | | | | | | 89 | | | | | | | | | | |  | | | | | | | |
| U | | 438 | | | | | | | | 141 | | | | | | | | | 353 | | | | | | | | | | 154 | | | | | | | | | | 133 | | | | | | | | | | 269 | | | | | | | | | 106 | | | | | | | | | | | | 466 | | | | | | | | | | 111 | | | | | | | | | 652 | | | | | | | | | | 172 | | | | | | | | | 396 | | | | | | | | | | | | 655 | | | | | | | | | | 203 | | | | | | | | | | |  | | | | | | | |
| Y | | 2473 | | | | | | | | 937 | | | | | | | | | 1924 | | | | | | | | | | 1708 | | | | | | | | | | 2817 | | | | | | | | | | 2034 | | | | | | | | | 370 | | | | | | | | | | | | 1678 | | | | | | | | | | 227 | | | | | | | | | 2877 | | | | | | | | | | 421 | | | | | | | | | 1840 | | | | | | | | | | | | 2779 | | | | | | | | | | 414 | | | | | | | | | | |  | | | | | | | |
| Hf | | 9853 | | | | | | | | 7839 | | | | | | | | | 9187 | | | | | | | | | | 8393 | | | | | | | | | | 6037 | | | | | | | | | | 8108 | | | | | | | | | 9191 | | | | | | | | | | | | 10137 | | | | | | | | | | 8012 | | | | | | | | | 7828 | | | | | | | | | | 8866 | | | | | | | | | 6711 | | | | | | | | | | | | 9532 | | | | | | | | | | 8434 | | | | | | | | | | |  | | | | | | | |
| Nb | | 14.75 | | | | | | | | 24.00 | | | | | | | | | 11.76 | | | | | | | | | | 1.31 | | | | | | | | | | 3.24 | | | | | | | | | | 8.69 | | | | | | | | | 2.21 | | | | | | | | | | | | 24.54 | | | | | | | | | | 1.19 | | | | | | | | | 24.08 | | | | | | | | | | 2.01 | | | | | | | | | 5.85 | | | | | | | | | | | | 37.15 | | | | | | | | | | 2.88 | | | | | | | | | | |  | | | | | | | |
| Ta | | 3.14 | | | | | | | | 4.69 | | | | | | | | | 2.76 | | | | | | | | | | 0.46 | | | | | | | | | | 0.57 | | | | | | | | | | 1.51 | | | | | | | | | 0.87 | | | | | | | | | | | | 4.55 | | | | | | | | | | 0.47 | | | | | | | | | 4.51 | | | | | | | | | | 0.76 | | | | | | | | | 1.03 | | | | | | | | | | | | 7.01 | | | | | | | | | | 0.82 | | | | | | | | | | |  | | | | | | | |
| P | | 1020 | | | | | | | | 206 | | | | | | | | | 337 | | | | | | | | | | 928 | | | | | | | | | | 358 | | | | | | | | | | 322 | | | | | | | | | 159 | | | | | | | | | | | | 2381 | | | | | | | | | | 107 | | | | | | | | | 573 | | | | | | | | | | 126 | | | | | | | | | 562 | | | | | | | | | | | | 799 | | | | | | | | | | 138 | | | | | | | | | | |  | | | | | | | |
| LREE | | 50 | | | | | | | | 43 | | | | | | | | | 36 | | | | | | | | | | 6 | | | | | | | | | | 31 | | | | | | | | | | 35 | | | | | | | | | 34 | | | | | | | | | | | | 125 | | | | | | | | | | 10 | | | | | | | | | 104 | | | | | | | | | | 14 | | | | | | | | | 46 | | | | | | | | | | | | 55 | | | | | | | | | | 21 | | | | | | | | | | |  | | | | | | | |
| MREE | | 277 | | | | | | | | 141 | | | | | | | | | 225 | | | | | | | | | | 229 | | | | | | | | | | 441 | | | | | | | | | | 269 | | | | | | | | | 50 | | | | | | | | | | | | 218 | | | | | | | | | | 25 | | | | | | | | | 385 | | | | | | | | | | 44 | | | | | | | | | 240 | | | | | | | | | | | | 333 | | | | | | | | | | 47 | | | | | | | | | | |  | | | | | | | |
| HREE | | 1648 | | | | | | | | 584 | | | | | | | | | 1289 | | | | | | | | | | 1067 | | | | | | | | | | 1631 | | | | | | | | | | 1254 | | | | | | | | | 230 | | | | | | | | | | | | 1182 | | | | | | | | | | 178 | | | | | | | | | 1883 | | | | | | | | | | 346 | | | | | | | | | 1115 | | | | | | | | | | | | 1876 | | | | | | | | | | 332 | | | | | | | | | | |  | | | | | | | |
| (Yb/La)_n_ | | 252.1 | | | | | | | | 84.5 | | | | | | | | | 2972.2 | | | | | | | | | | 5101.2 | | | | | | | | | | 2254.1 | | | | | | | | | | 393.7 | | | | | | | | | 139.5 | | | | | | | | | | | | 60.5 | | | | | | | | | | 1727.4 | | | | | | | | | 2249.8 | | | | | | | | | | 5080.2 | | | | | | | | | 204.9 | | | | | | | | | | | | 777.5 | | | | | | | | | | 1054.7 | | | | | | | | | | |  | | | | | | | |
| (Sm/La)_n_ | | 2.4 | | | | | | | | 1.9 | | | | | | | | | 26.7 | | | | | | | | | | 70.4 | | | | | | | | | | 55.0 | | | | | | | | | | 5.2 | | | | | | | | | 2.5 | | | | | | | | | | | | 1.5 | | | | | | | | | | 19.3 | | | | | | | | | 30.9 | | | | | | | | | | 38.3 | | | | | | | | | 3.4 | | | | | | | | | | | | 7.9 | | | | | | | | | | 8.1 | | | | | | | | | | |  | | | | | | | |
| (Lu/Gd)_n_ | | 30.1 | | | | | | | | 15.8 | | | | | | | | | 27.6 | | | | | | | | | | 19.1 | | | | | | | | | | 12.8 | | | | | | | | | | 20.4 | | | | | | | | | 20.5 | | | | | | | | | | | | 23.8 | | | | | | | | | | 39.3 | | | | | | | | | 20.7 | | | | | | | | | | 41.9 | | | | | | | | | 21.1 | | | | | | | | | | | | 27.3 | | | | | | | | | | 35.3 | | | | | | | | | | |  | | | | | | | |
| Eu/Eu* | | 0.05 | | | | | | | | 0.07 | | | | | | | | | 0.11 | | | | | | | | | | 0.03 | | | | | | | | | | 0.18 | | | | | | | | | | 0.12 | | | | | | | | | 0.26 | | | | | | | | | | | | 0.16 | | | | | | | | | | 0.37 | | | | | | | | | 0.08 | | | | | | | | | | 0.28 | | | | | | | | | 0.12 | | | | | | | | | | | | 0.06 | | | | | | | | | | 0.24 | | | | | | | | | | |  | | | | | | | |
| Ce/Ce* | | 2.41 | | | | | | | | 2.80 | | | | | | | | | 27.51 | | | | | | | | | | 3.23 | | | | | | | | | | 6.17 | | | | | | | | | | 4.82 | | | | | | | | | 13.89 | | | | | | | | | | | | 1.41 | | | | | | | | | | 18.37 | | | | | | | | | 26.39 | | | | | | | | | | 52.48 | | | | | | | | | 3.47 | | | | | | | | | | | | 6.17 | | | | | | | | | | 19.53 | | | | | | | | | | |  | | | | | | | |
| U/Yb | | 0.481 | | | | | | | | 0.454 | | | | | | | | | 0.495 | | | | | | | | | | 0.270 | | | | | | | | | | 0.154 | | | | | | | | | | 0.400 | | | | | | | | | 0.828 | | | | | | | | | | | | 0.694 | | | | | | | | | | 1.046 | | | | | | | | | 0.626 | | | | | | | | | | 0.830 | | | | | | | | | 0.677 | | | | | | | | | | | | 0.625 | | | | | | | | | | 1.007 | | | | | | | | | | |  | | | | | | | |
| Nb/Yb | | 0.016 | | | | | | | | 0.077 | | | | | | | | | 0.017 | | | | | | | | | | 0.002 | | | | | | | | | | 0.004 | | | | | | | | | | 0.013 | | | | | | | | | 0.017 | | | | | | | | | | | | 0.037 | | | | | | | | | | 0.011 | | | | | | | | | 0.023 | | | | | | | | | | 0.010 | | | | | | | | | 0.010 | | | | | | | | | | | | 0.035 | | | | | | | | | | 0.014 | | | | | | | | | | |  | | | | | | | |
| Note: Element concentrations are reported in ppm. Eu/Eu* = 2×Eu_n_/(Sm_n_+Gd_n_). Ce/Ce* = 2×Ce_n_/(La_n_+Pr_n_). Chondrite REE values are after Sun and McDonough (1989). | | | | | | | | | | | | | | | | | | | | | | | | | | | | | | | | | | | | | | | | | | | | | | | | | | | | | | | | | | | | | | | | | | | | | | | | | | | | | | | | | | | | | | | | | | | | | | | | | | | | | | | | | | | | | | | | | | | | | | | | | | | | | | | | | | | | | | | | | | | | |  | | | | | | | |

Table S5. Trace element compositions and Pb isotope compositions of basalts from studied area

| Sample | Nb (ppm) | La (ppm) | Nb/La | ^206^Pb/^204^Pb |
| --- | --- | --- | --- | --- |
| *Southeastern NCC* | | | | |
| 07CL01 | 29.7 | 23.2 | 1.28 | 17.179 |
| 07CL03 | 90.5 | 70.7 | 1.28 | 17.995 |
| 07CL04 | 89.8 | 71.8 | 1.25 | 17.944 |
| 07CL05 | 46.9 | 35.7 | 1.31 | 17.867 |
| 07CL07 | 24.2 | 22.9 | 1.05 | 16.752 |
| 07CL08 | 59.4 | 42.9 | 1.39 |  |
| 07CL09 | 88.1 | 67.6 | 1.30 | 17.981 |
| 07CL10 | 27.9 | 21.2 | 1.31 | 17.184 |
| 07CL11 | 31.6 | 23.6 | 1.34 | 17.143 |
| 07CL13 | 24.7 | 18.7 | 1.32 | 17.320 |
| 05HF02 | 31.8 | 19.7 | 1.61 | 17.480 |
| 05HF04 | 34.4 | 21.1 | 1.63 | 17.485 |
| 05HF08 | 37.6 | 23.0 | 1.63 | 17.504 |
| 05HF09 | 38.2 | 24.4 | 1.57 | 17.414 |
| 05HF10 | 63.0 | 48.2 | 1.31 | 18.077 |
| 05HF15 | 62.9 | 47.3 | 1.33 | 18.049 |
| 05HF17 | 73.0 | 46.2 | 1.58 | 17.724 |
| 05HF18 | 76.8 | 45.2 | 1.70 | 17.815 |
| 06SW37 | 59.0 | 41.5 | 1.42 | 17.419 |
| 06SW45 | 35.1 | 25.4 | 1.38 | 17.873 |
| 06SW47 | 36.1 | 25.1 | 1.44 | 17.872 |
| 06SW48 | 35.1 | 25.2 | 1.39 | 17.895 |
| 06SW49 | 33.8 | 24.0 | 1.41 | 17.851 |
| 06SW50 | 31.7 | 24.6 | 1.29 | 17.875 |
|  |  |  |  |  |
| *Northeastern SCB* | | | | |
| 05NS02 | 101 | 62.8 | 1.61 | 17.763 |
| 05NS04 | 102 | 65.0 | 1.57 | 17.773 |
| 05WD05 | 25.4 | 28.1 | 0.91 | 16.254 |
| 05WD06 | 59.8 | 46.0 | 1.30 | 17.012 |
| 05WD08 | 58.0 | 44.9 | 1.29 | 17.014 |
| 05WD09 | 47.6 | 39.7 | 1.20 | 16.744 |
| 05WD11 | 60.5 | 46.8 | 1.29 | 16.972 |
| 05WD15 | 60.5 | 47.6 | 1.27 | 16.970 |
| 05WD16 | 56.8 | 43.8 | 1.30 | 17.201 |
| 05WD18 | 46.6 | 38.4 | 1.21 | 17.389 |
| 05WD22 | 44.6 | 31.6 | 1.41 | 17.198 |
| 05WD31 | 68.5 | 46.2 | 1.48 | 17.668 |
| 05WD33 | 80.0 | 54.2 | 1.48 | 17.759 |
| 05WD36 | 29.0 | 24.1 | 1.20 | 16.697 |
| 05WD39 | 19.5 | 17.0 | 1.15 | 16.753 |
| 05WD41 | 34.6 | 29.5 | 1.18 | 16.710 |
| 05WD42 | 38.7 | 30.3 | 1.28 | 16.726 |
| 06SW19 | 70.7 | 50.4 | 1.40 | 17.865 |
| 06SW21 | 80.6 | 58.1 | 1.39 | 18.108 |
| 06SW23 | 79.3 | 56.2 | 1.41 | 18.029 |
| 06SW25 | 36.6 | 36.1 | 1.01 | 17.446 |
| 06SW26 | 47.1 | 31.9 | 1.48 | 17.134 |
| 06SW28 | 22.1 | 16.0 | 1.38 | 16.842 |
| 06SW30 | 37.0 | 27.1 | 1.37 | 17.284 |
| 06SW31 | 36.1 | 26.3 | 1.37 | 17.294 |
| 06SW33 | 23.9 | 18.4 | 1.30 | 16.933 |
|  |  |  |  |  |
| Table S5 (continued) | | | | |
| Sample | Nb (ppm) | La (ppm) | Nb/La | ^206^Pb/^204^Pb |
| 06SW34 | 32.3 | 28.3 | 1.14 | 17.584 |
| 06SW35 | 36.0 | 30.4 | 1.18 | 17.664 |
|  |  |  |  |  |
| *Sulu orogen* |  |  |  |  |
| 06SW03 | 105 | 77.3 | 1.36 | 17.915 |
| 06SW04 | 98 | 76.3 | 1.29 | 17.913 |
| 06SW05 | 115 | 88.0 | 1.31 | 17.876 |
| 06SW07 | 94 | 73.9 | 1.27 | 17.924 |
| 06SW08 | 124 | 73.8 | 1.68 | 17.846 |
| 06SW09 | 111 | 76.7 | 1.45 | 18.039 |
| 06SW11 | 120 | 84.6 | 1.42 | 17.995 |
| 06SW12 | 119 | 93.0 | 1.28 | 17.942 |
| 06SW13 | 123 | 91.8 | 1.34 | 17.925 |
| 06SW15 | 100 | 89.2 | 1.12 | 17.996 |

Note: Data are from Zhang et al. (2009); Wang et al. (2011) and Xu et al. (2012).

Table S6. Major and trace element compositions of magmatic zircon-bearing mafic igneous rocks

| Sample | JR31 12-68 | JR31 22-1 | JN-1 | 07JN38 | 05SD34 | 05SD37 | 05SD38 | 05SD40 |
| --- | --- | --- | --- | --- | --- | --- | --- | --- |
| Location^a^ | IMOR | IMOR | NCC | NCC | DSO | DSO | DSO | DSO |
| Rock type | gabbro | gabbro | gabbro | gabbro | gabbro | gabbro | gabbro | gabbro |
| *Major element (wt.%)* | | | | | | | | |
| SiO_2_ | 48.70 | 51.20 | 49.89 | 48.85 | 47.22 | 45.86 | 45.10 | 48.57 |
| TiO_2_ | 0.24 | 0.53 | 0.57 | 0.44 | 1.07 | 1.24 | 1.30 | 0.73 |
| Al_2_O_3_ | 15.70 | 16.50 | 13.26 | 12.59 | 9.15 | 11.55 | 10.61 | 10.05 |
| Fe_2_O_3_^T^ | 8.20 | 6.50 | 10.58 | 9.89 | 8.26 | 8.64 | 8.96 | 7.61 |
| MnO | 0.14 | 0.12 | 0.17 | 0.17 | 0.13 | 0.10 | 0.11 | 0.12 |
| MgO | 11.10 | 8.20 | 11.02 | 13.55 | 10.20 | 9.87 | 10.77 | 8.12 |
| CaO | 11.00 | 13.70 | 10.85 | 9.09 | 13.82 | 12.02 | 12.55 | 14.29 |
| Na_2_O | 2.20 | 2.70 | 2.30 | 1.94 | 1.29 | 1.52 | 0.83 | 1.60 |
| K_2_O | 0.11 | 0.08 | 0.89 | 0.53 | 3.50 | 4.11 | 4.21 | 3.39 |
| P_2_O_5_ | 0.03 | 0.04 | 0.13 | 0.08 | 3.02 | 2.74 | 3.19 | 3.42 |
| LOI | 2.30 | 0.70 | -0.03 | 2.38 | 1.12 | 1.47 | 2.23 | 1.23 |
| Total | 97.50 | 99.70 | 99.64 | 99.51 | 98.79 | 99.12 | 99.86 | 99.15 |
|  |  |  |  |  |  |  |  |  |
| *Trace element (ppm)* | | | | | | | | |
| Cs | 0.02 | 0.05 |  |  |  |  |  |  |
| Rb | 0.28 | 0.81 | 17.1 | 8.31 | 81.2 | 96.1 | 111 | 79.2 |
| Ba | 3.49 | 10.8 | 403 | 279 | 9484 | 15010 | 13528 | 8628 |
| Th |  |  | 0.86 | 0.42 | 14.5 | 14.9 | 22.5 | 9.83 |
| U |  |  | 0.26 | 0.14 | 2.54 | 2.59 | 3.43 | 1.56 |
| Nb | 0.20 | 0.42 | 2.28 | 2.13 | 12.6 | 15.4 | 18.1 | 11.6 |
| Ta |  |  | 0.16 | 0.11 | 0.65 | 0.71 | 0.81 | 0.49 |
| La | 1.18 | 1.35 | 8.99 | 6.49 | 166 | 149 | 176 | 181 |
| Ce | 3.97 | 4.63 | 21.4 | 14.3 | 349 | 318 | 363 | 375 |
| Pr | 0.61 | 0.83 | 3.05 | 1.98 | 40.9 | 38 | 42.2 | 43.9 |
| Pb |  |  | 2.36 | 2.89 | 18.1 | 26.5 | 14.7 | 25.0 |
| Nd | 3.31 | 4.55 | 13.6 | 8.71 | 182 | 166 | 183 | 189 |
| Sr | 177 | 191 | 657 | 496 | 2137 | 2603 | 3343 | 2915 |
| Zr | 25 | 37 | 46 | 32 | 137 | 123 | 174 | 153 |
| Hf |  |  | 1.08 | 0.99 | 3.71 | 3.41 | 3.99 | 4.04 |
| Sm | 1.24 | 1.74 | 3.13 | 2.42 | 27.7 | 25.5 | 26.9 | 28.5 |
| Eu | 0.66 | 0.78 | 1.06 | 0.82 | 6.20 | 5.88 | 6.31 | 6.53 |
| Gd | 1.80 | 2.39 | 2.88 | 2.18 | 16.4 | 15.4 | 16.6 | 16.9 |
| Tb | 0.37 | 0.49 | 0.45 | 0.33 | 1.85 | 1.65 | 1.75 | 1.83 |
| Dy | 2.35 | 3.14 | 2.47 | 2.05 | 7.56 | 6.59 | 7.02 | 7.60 |
| Ho | 0.52 | 0.69 | 0.47 | 0.41 | 1.22 | 1.00 | 1.05 | 1.19 |
| Y | 13.3 | 18.7 | 12.4 | 10.4 | 33.0 | 28.7 | 30.9 | 34.0 |
| Er | 1.38 | 1.86 | 1.21 | 1.11 | 2.94 | 2.36 | 2.49 | 2.82 |
| Yb | 1.28 | 1.84 | 1.05 | 1.07 | 2.04 | 1.55 | 1.64 | 2.01 |
| Lu | 0.19 | 0.26 | 0.17 | 0.16 | 0.28 | 0.23 | 0.24 | 0.28 |

| Table S6 (continued) | | | | | | | | |
| --- | --- | --- | --- | --- | --- | --- | --- | --- |
| Sample | 00SD47 | 00DP01 | 09JZY14 | T108 | 01SHC05 | 09ZJP08 | 09DSC04 | 09DB97 |
| Location^a^ | DSO | DSO | DSO | DA | DSO | DSO | DSO | DSO |
| Rock type | gabbro | gabbro | gabbro | gabbro | pyroxenite | pyroxenite | hornblendite | hornblendite |
| *Major element (wt.%)* | | | | | | | | |
| SiO_2_ | 51.49 | 50.02 | 47.81 | 49.97 | 49.97 | 50.11 | 45.83 | 45.79 |
| TiO_2_ | 0.60 | 0.98 | 1.54 | 0.59 | 0.48 | 0.46 | 1.03 | 1.76 |
| Al_2_O_3_ | 12.15 | 12.91 | 15.84 | 11.47 | 6.98 | 5.62 | 8.30 | 16.70 |
| Fe_2_O_3_^T^ | 7.40 | 8.32 | 10.28 | 8.62 | 11.28 | 10.65 | 12.78 | 11.23 |
| MnO | 0.18 | 0.15 | 0.16 | 0.18 | 0.17 | 0.22 | 0.24 | 0.18 |
| MgO | 7.23 | 11.28 | 6.84 | 10.47 | 17.58 | 14.34 | 14.53 | 5.47 |
| CaO | 16.41 | 7.42 | 7.57 | 13.29 | 9.10 | 14.63 | 10.85 | 8.41 |
| Na_2_O | 1.83 | 2.24 | 3.64 | 2.03 | 1.43 | 0.80 | 1.22 | 3.49 |
| K_2_O | 0.38 | 3.82 | 2.55 | 0.18 | 1.32 | 0.21 | 1.25 | 2.14 |
| P_2_O_5_ | 0.45 | 0.69 | 0.74 | 0.03 | 0.20 | 0.04 | 0.18 | 1.50 |
| LOI | 0.47 | 0.87 | 0.26 | 2.92 | 2.19 | 0.71 | 0.03 | 0.14 |
| Total | 98.59 | 98.70 | 97.20 | 99.75 | 100.70 | 97.80 | 96.24 | 96.82 |
|  |  |  |  |  |  |  |  |  |
| *Trace element (ppm)* | | | | | | | | |
| Cs |  |  |  | 0.41 |  |  |  |  |
| Rb | 7.90 | 93.6 | 71.1 | 4.16 | 32.0 | 2.26 | 28.7 | 44.8 |
| Ba | 283 | 3304 | 2483 | 29.5 | 500 | 131 | 293 | 1991 |
| Th | 2.89 | 6.05 | 1.75 | 0.15 | 2.10 | 0.21 | 0.64 | 0.99 |
| U | 0.55 | 1.18 | 0.208 | 0.054 | 0.90 | 0.05 | 0.13 | 0.30 |
| Nb | 4.92 | 15.3 | 17.4 | 0.39 | 4.00 | 0.91 | 9.30 | 12.1 |
| Ta | 0.46 | 0.81 | 0.56 | 0.03 | 0.50 | 0.05 | 0.39 | 0.44 |
| La | 44.6 | 70.3 | 75.9 | 1.05 | 23.64 | 7.84 | 34.4 | 57.3 |
| Ce | 97.6 | 137 | 148 | 3.28 | 42.9 | 22.0 | 93.1 | 134 |
| Pr | 11.6 | 16.5 | 17.5 | 0.57 | 5.39 | 3.41 | 12.5 | 18.5 |
| Pb | 6.58 | 20.2 | 9.00 | 0.48 | 18.1 | 1.65 | 2.75 | 7.49 |
| Nd | 49.6 | 63.6 | 68.1 | 2.97 | 18.8 | 17.4 | 51.2 | 80.0 |
| Sr | 551 | 1158 | 1179 | 90 | 279 | 220 | 82 | 1308 |
| Zr | 79 | 325 | 154 | 12 | 84 | 46 | 91 | 77 |
| Hf | 2.65 | 7.44 | 3.34 | 0.40 | 1.60 | 1.70 | 3.05 | 2.13 |
| Sm | 9.53 | 11.3 | 11.4 | 1.03 | 3.71 | 4.56 | 9.86 | 14.6 |
| Eu | 2.06 | 3.1 | 3.01 | 0.40 | 1.07 | 1.20 | 2.62 | 3.84 |
| Gd | 7.53 | 9.05 | 8.99 | 1.36 | 3.14 | 4.34 | 7.94 | 11.1 |
| Tb | 1.22 | 1.08 | 1.19 | 0.26 | 0.45 | 0.73 | 1.12 | 1.38 |
| Dy | 6.49 | 5.41 | 5.90 | 1.82 | 2.41 | 4.44 | 5.92 | 6.68 |
| Ho | 1.29 | 0.91 | 1.00 | 0.40 | 0.45 | 0.83 | 1.08 | 1.13 |
| Y | 35.2 | 25.8 | 28.4 | 11.3 | 14.5 | 23.3 | 30.7 | 31.0 |
| Er | 3.48 | 2.36 | 2.61 | 1.20 | 1.23 | 2.37 | 2.81 | 2.73 |
| Yb | 3.09 | 1.91 | 1.94 | 1.18 | 1.19 | 2.19 | 2.40 | 2.06 |
| Lu | 0.44 | 0.27 | 0.30 | 0.18 | 0.16 | 0.33 | 0.36 | 0.29 |

| Table S6 (continued) | | | | | | | | |
| --- | --- | --- | --- | --- | --- | --- | --- | --- |
| Sample | 09DSC02 | 09DB96 | 09ZJP06 | 09DB100 | 12LZ14 | ZK10-37 | HQ10-03 | C-1412-1 |
| Location^a^ | DSO | DSO | DSO | DSO | QO | QO | QO | KC |
| Rock type | hornblendite | hornblendite | hornblendite | hornblendite | basalt | basalt | basalt | dolerite |
| *Major element (wt.%)* | | | | | | | | |
| SiO_2_ | 45.62 | 48.81 | 44.68 | 45.11 | 44.72 | 45.82 | 44.27 | 49.39 |
| TiO_2_ | 1.62 | 1.43 | 0.99 | 1.54 | 2.47 | 2.54 | 2.80 | 0.80 |
| Al_2_O_3_ | 10.43 | 18.83 | 10.46 | 18.06 | 15.55 | 14.50 | 16.14 | 16.34 |
| Fe_2_O_3_^T^ | 12.73 | 8.84 | 14.14 | 10.69 | 10.46 | 11.00 | 11.98 | 12.62 |
| MnO | 0.21 | 0.12 | 0.21 | 0.13 | 0.15 | 0.16 | 0.07 | 0.22 |
| MgO | 11.96 | 3.91 | 10.73 | 5.65 | 8.86 | 9.86 | 4.84 | 7.59 |
| CaO | 10.44 | 6.47 | 11.19 | 10.78 | 9.17 | 8.56 | 4.62 | 11.67 |
| Na_2_O | 2.16 | 4.80 | 1.25 | 2.94 | 3.28 | 3.33 | 4.82 | 2.10 |
| K_2_O | 1.09 | 2.30 | 1.09 | 1.07 | 1.47 | 1.02 | 0.60 | 0.20 |
| P_2_O_5_ | 0.14 | 0.66 | 0.26 | 0.39 | 0.46 | 0.38 | 0.68 | 0.08 |
| LOI | 0.11 | 0.03 | 0.34 | 0.32 | 2.89 | 3.64 | 8.32 | 0.10 |
| Total | 96.52 | 96.20 | 95.34 | 96.68 | 99.52 | 100.81 | 99.14 | 101.00 |
|  |  |  |  |  |  |  |  |  |
| *Trace element (ppm)* | | | | | | | | |
| Cs |  |  |  |  |  |  |  |  |
| Rb | 12.9 | 50.3 | 43.8 | 27.7 | 11.7 | 6.90 | 6.23 | 6.00 |
| Ba | 403 | 2245 | 260 | 397 | 180 | 201 | 51 | 29 |
| Th | 2.03 | 3.06 | 0.59 | 0.70 | 1.76 | 2.66 | 3.00 | 0.21 |
| U | 0.28 | 0.28 | 0.12 | 0.13 | 0.76 | 1.01 | 1.19 | 0.04 |
| Nb | 11.2 | 10.0 | 1.61 | 5.20 | 32.2 | 44.5 | 52.8 | 2.34 |
| Ta | 0.51 | 0.33 | 0.1.0 | 0.25 | 1.95 | 2.54 | 3.12 |  |
| La | 43.5 | 69.3 | 9.45 | 18.2 | 21.0 | 29.1 | 33.5 | 2.49 |
| Ce | 108 | 126 | 25.4 | 45.6 | 45.0 | 57.2 | 64.9 | 6.55 |
| Pr | 15.0 | 14.6 | 3.91 | 6.63 | 5.51 | 6.98 | 7.58 | 0.91 |
| Pb | 3.58 | 8.44 | 3.29 | 5.44 | 2.00 | 3.16 | 2.26 | 0.37 |
| Nd | 64.5 | 55 | 19.4 | 29.9 | 23.3 | 28.6 | 30.9 | 4.56 |
| Sr | 364 | 1539 | 821 | 1135 | 1209 | 712 | 402 | 106 |
| Zr | 69 | 185 | 42.6 | 58 | 213 | 260 | 284 | 47 |
| Hf | 3.03 | 4.06 | 1.43 | 1.90 | 4.37 | 5.30 | 5.82 |  |
| Sm | 12.8 | 8.62 | 4.97 | 7.22 | 5.38 | 5.83 | 6.19 | 1.64 |
| Eu | 3.38 | 2.57 | 1.27 | 2.23 | 1.87 | 1.90 | 1.99 | 0.66 |
| Gd | 10.5 | 6.28 | 4.83 | 7.10 | 5.52 | 5.62 | 5.81 | 2.18 |
| Tb | 1.40 | 0.77 | 0.73 | 0.99 | 0.88 | 0.87 | 0.85 | 0.41 |
| Dy | 7.14 | 3.74 | 4.21 | 5.84 | 4.96 | 4.95 | 4.55 | 2.83 |
| Ho | 1.23 | 0.65 | 0.77 | 1.03 | 0.96 | 0.94 | 0.79 | 0.59 |
| Y | 34.6 | 18.3 | 21.2 | 28.1 | 26.2 | 26.3 | 22.1 | 13.0 |
| Er | 3.28 | 1.63 | 2.07 | 2.70 | 2.65 | 2.46 | 2.06 | 1.66 |
| Yb | 2.59 | 1.30 | 1.78 | 2.20 | 2.19 | 2.06 | 1.63 | 1.66 |
| Lu | 0.38 | 0.20 | 0.25 | 0.31 | 0.34 | 0.29 | 0.24 | 0.24 |

| Table S6 (continued) | | | | |
| --- | --- | --- | --- | --- |
| Sample | C-1412-5 | C-1412 | C-1412-12 | C-1412-13 |
| Location^a^ | KC | KC | KC | KC |
| Rock type | dolerite | dolerite | dolerite | dolerite |
| *Major element (wt.%)* | | | | |
| SiO_2_ | 49.91 | 47.83 | 49.25 | 49.05 |
| TiO_2_ | 1.12 | 1.82 | 0.79 | 0.95 |
| Al_2_O_3_ | 13.54 | 13.20 | 16.63 | 15.33 |
| Fe_2_O_3_^T^ | 15.05 | 18.66 | 11.89 | 13.50 |
| MnO | 0.21 | 0.27 | 0.19 | 0.22 |
| MgO | 7.40 | 5.59 | 7.61 | 7.44 |
| CaO | 11.53 | 9.64 | 12.17 | 12.01 |
| Na_2_O | 2.12 | 2.21 | 2.09 | 2.21 |
| K_2_O | 0.30 | 0.27 | 0.22 | 0.15 |
| P_2_O_5_ | 0.12 | 0.12 | 0.09 | 0.12 |
| LOI | 0.10 | 0.14 | 1.17 | 0.88 |
| Total | 101.30 | 99.75 | 102.11 | 101.86 |
|  |  |  |  |  |
| *Trace element (ppm)* | | | | |
| Cs |  |  |  |  |
| Rb | 13.1 | 7.59 | 11.2 | 3.29 |
| Ba | 37.8 | 67.8 | 35.5 | 32.2 |
| Th | 0.26 | 0.39 | 0.17 | 0.21 |
| U | 0.06 | 0.10 | 0.04 | 0.06 |
| Nb | 3.48 | 5.77 | 2.05 | 2.68 |
| Ta |  |  |  |  |
| La | 3.5 | 5.12 | 1.97 | 2.42 |
| Ce | 8.78 | 13.3 | 5.03 | 6.52 |
| Pr | 1.33 | 2.02 | 0.79 | 1.00 |
| Pb | 0.69 | 2.59 | 0.67 | 0.98 |
| Nd | 6.45 | 10.1 | 4.18 | 5.32 |
| Sr | 106 | 121 | 102 | 97.6 |
| Zr | 63 | 85 | 47 | 63 |
| Hf |  |  |  |  |
| Sm | 2.26 | 3.19 | 1.37 | 1.72 |
| Eu | 0.844 | 1.19 | 0.557 | 0.63 |
| Gd | 3.05 | 4.46 | 1.85 | 2.27 |
| Tb | 0.56 | 0.74 | 0.34 | 0.41 |
| Dy | 3.9 | 4.69 | 2.25 | 2.75 |
| Ho | 0.82 | 1.01 | 0.49 | 0.59 |
| Y | 18.1 | 27.9 | 11.9 | 14.9 |
| Er | 2.31 | 3.04 | 1.42 | 1.77 |
| Yb | 2.28 | 2.9 | 1.45 | 1.75 |
| Lu | 0.34 | 0.42 | 0.22 | 0.25 |
| ^a^Abbreviations: IMOR: Indian Mid-Ocean Ridge; NCC: North China Craton; DSOB: Dabie-Sulu orogenic belt, China; QO: Qinling orogen, China; DA: Dorud–Azna, Iran; KC: Karelian Craton.  Data sources: IMOR: Coogan et al. (2001); NCC: Huang et al. (2012), Yang et al. (2012); DSOB: Zhao et al. (2005, 2012), Dai et al. (2012, 2015a, 2015b), Zhang et al. (2012); QO: Ding et al. (2013), Li et al. (2013), Dai et al. (2014); DA: Shakerardakani et al. (2015); KC: Stepanova et al. (2014). | | | | |

| Table S7. Calculated temperatures for mantle melting to produce  Cenozoic continental basalts in east-central China   | Sample | SiO_2_ (wt.%) | MgO  (wt.%) | T (°C)  Albarede (1992) | T (°C)  Herzberg and Asimow (2008) | | --- | --- | --- | --- | --- | | 07CL03 | 42.40 | 7.79 | 1279 | 1187 | | 07CL05 | 43.04 | 11.09 | 1379 | 1341 | | 07CL07 | 47.58 | 9.27 | 1295 | 1266 | | 07CL08 | 42.91 | 9.69 | 1337 | 1285 | | 05NS04 | 39.21 | 8.76 | 1334 | 1241 | | 05WD15 | 46.96 | 6.03 | 1197 | 1055 | | 05WD16 | 45.34 | 8.88 | 1297 | 1247 | | 05WD31 | 43.04 | 9.40 | 1328 | 1272 | | 06SW05 | 41.82 | 8.54 | 1308 | 1229 | | 06SW07 | 41.62 | 10.14 | 1361 | 1304 | | 06SW09 | 41.31 | 7.31 | 1270 | 1156 | | 06SW11 | 40.66 | 8.97 | 1330 | 1251 | | 06SW21 | 40.57 | 12.10 | 1428 | 1376 | | 06SW25 | 47.31 | 6.21 | 1201 | 1071 | | 06SW28 | 49.86 | 6.15 | 1189 | 1066 | | 06SW45 | 47.62 | 6.56 | 1211 | 1101 | | 06SW48 | 48.15 | 6.74 | 1215 | 1115 | | 05HF15 | 42.68 | 11.61 | 1397 | 1359 | | 05HF18 | 43.76 | 10.83 | 1366 | 1331 | | Note: The major element composition of basalts are from Zhang et al. (2009), Wang et al. (2011) and Xu et al. (2012). | | | | | |
| --- | --- | --- | --- | --- | --- | --- | --- | --- | --- | --- | --- | --- | --- | --- | --- | --- | --- | --- | --- | --- | --- | --- | --- | --- | --- | --- | --- | --- | --- | --- | --- | --- | --- | --- | --- | --- | --- | --- | --- | --- | --- | --- | --- | --- | --- | --- | --- | --- | --- | --- | --- | --- | --- | --- | --- | --- | --- | --- | --- | --- | --- | --- | --- | --- | --- | --- | --- | --- | --- | --- | --- | --- | --- | --- | --- | --- | --- | --- | --- | --- | --- | --- | --- | --- | --- | --- | --- | --- | --- | --- | --- | --- | --- | --- | --- | --- | --- | --- | --- | --- | --- | --- | --- | --- | --- |

**References for Tables S5, S6 and S7**

Albarede, F., 1992. How deep do common basaltic magmas form and differentiate? Journal of Geophysical Research 97, 10997–11009.

Coogan, L.A., MacLeod, C.J., Dick, H.J.B., Edwards, S.J., Kvassnes, A., Natland, J.H., Robinson, P.T., Thompson, G., O'Hara, M.J., 2001. Whole-rock geochemistry of gabbros from the Southwest Indian Ridge: constraints on geochemical fractionations between the upper and lower oceanic crust and magma chamber processes at (very) slow-spreading ridges. Chemical Geology 178, 1-22.

Dai, L.-Q., Zhao, Z.-F., Zheng, Y.-F., Zhang, J., 2012. The nature of orogenic lithospheric mantle: Geochemical constraints from postcollisional mafic–ultramafic rocks in the Dabie orogen. Chemical Geology 334, 99-121.

Dai, L.-Q., Zhao, Z.-F., Zheng, Y.-F., 2014. Geochemical insights into the role of metasomatic hornblendite in generating alkali basalts. Geochemistry, Geophysics, Geosystems 15, 3762-3779.

Dai, L.-Q., Zhao, Z.-F., Zheng, Y.-F., 2015a. Tectonic development from oceanic subduction to continental collision: Geochemical evidence from postcollisional mafic rocks in the Hong'an–Dabie orogens. Gondwana Research 27, 1236-1254.

Dai, L.-Q., Zhao, Z.-F., Zheng, Y.-F., Zhang, J., 2015b. Source and magma mixing processes in continental subduction factory: Geochemical evidence from postcollisional mafic igneous rocks in the Dabie orogen. Geochemistry, Geophysics, Geosystems 16, 659-680.

Ding, Y., Yu, XH., Mo, XX., Li, XW., Huang, XF., Wei, P., He, WY., 2013. Geochronology, geochemistry and petrogenesis of the Hongqiang basalts from Northeast Qinghai-Tibetan Plateau. Earth Science Fronties 20, 180-191 (in Chinese with English abstract).

Herzberg, C., Asimow, P.D., 2008. Petrology of some oceanic island basalts: PRIMELT2.XLS software for primary magma calculation. Geochemistry Geophysics Geosystems 9, Q09001.

Huang, X.-L., Zhong, J.-W., Xu, Y.-G., 2012. Two tales of the continental lithospheric mantle prior to the destruction of the North China Craton: Insights from Early Cretaceous mafic intrusions in western Shandong, East China. Geochimica et Cosmochimica Acta 96, 193-214.

Li, X.-W., Mo, X.-X., Yu, X.-H., Ding, Y., Huang, X.-F., Wei, P., He, W.-Y., 2013. Geochronological, geochemical and Sr–Nd–Hf isotopic constraints on the origin of the Cretaceous intraplate volcanism in West Qinling, Central China: Implications for asthenosphere–lithosphere interaction. Lithos 177, 381-401.

Shakerardakani, F., Neubauer, F., Masoudi, F., Mehrabi, B., Liu, X., Dong, Y., Mohajjel, M., Monfaredi, B., Friedl, G., 2015. Panafrican basement and Mesozoic gabbro in the Zagros orogenic belt in the Dorud–Azna region (NW Iran): Laser-ablation ICP–MS zircon ages and geochemistry. Tectonophysics 647–648, 146-171.

Stepanova, A.V., Samsonov, A.V., Salnikova, E.B., Puchtel, I.S., Larionova, Y.O., Larionov, A.N., Stepanov, V.S., Shapovalov, Y.B., Egorova, S.V., 2014. Palaeoproterozoic Continental MORB-type Tholeiites in the Karelian Craton: Petrology, Geochronology, and Tectonic Setting. Journal of Petrology 55, 1719-1751.

Wang, Y., Zhao, Z.-F., Zheng, Y.-F., Zhang, J.-J., 2011. Geochemical constraints on the nature of mantle source for Cenozoic continental basalts in east-central China. Lithos 125, 940-955.

Xu, Z., Zhao, Z.-F., Zheng, Y.-F., 2012. Slab–mantle interaction for thinning of cratonic lithospheric mantle in North China: Geochemical evidence from Cenozoic continental basalts in central Shandong. Lithos 146–147, 202-217.

Yang, Q.-L., Zhao, Z.-F., Zheng, Y.-F., 2012. Modification of subcontinental lithospheric mantle above continental subduction zone: Constraints from geochemistry of Mesozoic gabbroic rocks in southeastern North China. Lithos 146–147, 164-182.

Zhang, J.-J., Zheng, Y.-F., Zhao, Z.-F., 2009. Geochemical evidence for interaction between oceanic crust and lithospheric mantle in the origin of Cenozoic continental basalts in east-central China. Lithos 110, 305-326.

Zhang, J., Zhao, Z.-F., Zheng, Y.-F., Liu, X.-M., Xie, L.-W., 2012. Zircon Hf–O isotope and whole-rock geochemical constraints on origin of postcollisional mafic to felsic dykes in the Sulu orogen. Lithos 136–139, 225-245.

Zhao, Z.-F., Zheng, Y.-F., Wei, C.-S., Wu, Y.-B., Chen, F.-K., Jahn, B.-m., 2005. Zircon U–Pb age, element and C–O isotope geochemistry of post-collisional mafic-ultramafic rocks from the Dabie orogen in east-central China. Lithos 83, 1-28.

Zhao, Z.-F., Zheng, Y.-F., Zhang, J., Dai, L.-Q., Li, Q., Liu, X., 2012. Syn-exhumation magmatism during continental collision: Evidence from alkaline intrusives of Triassic age in the Sulu orogen. Chemical Geology 328, 70-88.

1. ^*^ Corresponding author. E-mail address: zxu85@ustc.edu.cn [↑](#footnote-ref-2)
